# TIGER: Toolbox for integrating genome-scale metabolic models, expression data, and transcriptional regulatory networks

**DOI:** 10.1186/1752-0509-5-147

**Published:** 2011-09-23

**Authors:** Paul A Jensen, Kyla A Lutz, Jason A Papin

**Affiliations:** 1 Department of Biomedical Engineering, University of Virginia, Charlottesville, VA 22908, USA

## Abstract

**Background:**

Several methods have been developed for analyzing genome-scale models of metabolism and transcriptional regulation. Many of these methods, such as Flux Balance Analysis, use constrained optimization to predict relationships between metabolic flux and the genes that encode and regulate enzyme activity. Recently, mixed integer programming has been used to encode these gene-protein-reaction (GPR) relationships into a single optimization problem, but these techniques are often of limited generality and lack a tool for automating the conversion of rules to a coupled regulatory/metabolic model.

**Results:**

We present TIGER, a Toolbox for Integrating Genome-scale Metabolism, Expression, and Regulation. TIGER converts a series of generalized, Boolean or multilevel rules into a set of mixed integer inequalities. The package also includes implementations of existing algorithms to integrate high-throughput expression data with genome-scale models of metabolism and transcriptional regulation. We demonstrate how TIGER automates the coupling of a genome-scale metabolic model with GPR logic and models of transcriptional regulation, thereby serving as a platform for algorithm development and large-scale metabolic analysis. Additionally, we demonstrate how TIGER's algorithms can be used to identify inconsistencies and improve existing models of transcriptional regulation with examples from the reconstructed transcriptional regulatory network of *Saccharomyces cerevisiae*.

**Conclusion:**

The TIGER package provides a consistent platform for algorithm development and extending existing genome-scale metabolic models with regulatory networks and high-throughput data.

## Background

Constraint-Based Reconstruction and Analysis (COBRA) methods have allowed the study of metabolism on a genome-wide scale [1]. These models have been used to understand the interplay between environmental and genetic perturbations and the metabolic capabilities of an organism. Applications of COBRA methods have led to increased understanding in the fields of bioprocess optimization [2], pathogenicity [3], symbiosis [4], biofuel production [5], and human disease [6].

### The Gene-Protein-Reaction relationship

COBRA models often contain two sets of biological information, a matrix of stoichiometric data for metabolic reactions, and a mapping between gene-encoding enzymes and the reactions they catalyze (the gene-protein-reaction, or GPR, relationship). Predicting the metabolic capabilities of a COBRA model is possible with Flux Balance Analysis (FBA), a two-stage mathematical technique based on the observation that metabolic networks often display optimal dynamics [7]. In the first stage of FBA, genes in the modeled organism are classified as either "on" or "off" to create an *in silico *genetic state. Turning genes "off" can be used to simulate significant reductions in expression levels or complete knockouts. The GPR for each reaction, represented as a binary rule, determines if a sufficient collection of proteins (isozymes, enzymatic subunits, etc.) is present for the reaction to carry flux. All reactions with satisfied GPR rules are collected into a stoichiometric matrix. The second stage of FBA uses linear programming to calculate a thermodynamically-feasible, mass-balanced flux distribution that maximizes the flux through an objective reaction. The objective reactions used in FBA vary among organisms, ranging from ATP maintenance to biomass production [8]. By assuming that the fluxes through a metabolic network have evolved to maximize an objective, FBA eliminates the need for detailed kinetic information for each of the thousands of reactions in a complete metabolic reconstruction.

The GPR rules do not always describe a one-to-one mapping between genes and reactions (where one gene encodes a complete enzyme that independently catalyzes one reaction). For example, a COBRA reconstruction for the yeast *Saccharomyces cerevisiae *[9] contains 1266 metabolic reactions; 231 (18.3%) of these reactions have complex GPR associations. The most complex GPR in this model involves the products of 18 open reading frames. The entire set of GPR rules contains 340 instances of isozyme-like behavior (two proteins both able to fully catalyze a reaction) and 279 different complexes of protein subunits.

Because of the complexity of the GPR mappings, early extensions to FBA were reaction, rather than gene, centric. For example, the OptKnock [2] algorithm removed reactions from a FBA model to design a strain of *E. coli *with optimal production of a metabolic byproduct. Ideally, OptKnock would operate by removing genes, not reactions, since it is not straightforward to independently remove reactions from a biological system without genetic manipulations. An optimization using genes as decision variables would require a method for encoding the GPR logic into a set of linear inequalities. This encoding was developed as SR-FBA [10] using a mixed integer optimization approach for GPR logic and other Boolean regulatory rules. An SR-FBA-based approach was later used to develop OptORF, a method to design microbial strains through gene knockouts and overexpression [11]. Other gene-centric, FBA-related algorithms have been developed, each using a variation of the SR-FBA method [12-14]. However, a general software platform for coupling GPR rules of arbitrary complexity with a COBRA model using mixed integer programming was not available. Such a tool would speed the development of new algorithms by removing the need for researchers to re-implement this complex process.

### Transcriptional regulatory networks

The accuracy of COBRA models has been improved through the addition of transcriptional regulatory networks (TRNs) [15,16]. The TRN is a set of rules that relate the expression states of metabolic genes to various genetic and environmental cues. Because of the paucity of kinetic details available to describe these relationships, genome-scale models often represent gene expression and environmental cues in a binary, "on" or "off" format. This approach allows TRNs to be described with Boolean logic.

The first genome-scale TRNs were applied to models of *Escherichia coli *[15] and *Saccharomyces cerevisiae *[16] metabolism. The rules were written in standard Boolean format, where each Boolean variable is given by an explicit function of the other variables. This method creates two significant problems. First, the TRN uses the absence or presence of metabolites in the extracellular environment to calculate which genes (and, subsequently, reactions) will be active. However, certain metabolic pathways secrete byproducts into the extracellular space, thereby changing the environment. Studies with the *E. coli *and *S. cerevisiae *TRNs used an iterative approach [17] - applying the TRN to the metabolic network in a starting environment, determining which metabolites would be secreted, and then repeating the process in the new environment until the environment no longer changes between iterations. A more straightforward approach would be to solve the TRN and metabolic networks simultaneously by formulating both problems in a single optimization.

A second obstacle with TRN integration is that the explicit rule formulation used by previous studies [17] can over-constrain the metabolic model. (In explicit rules, each gene's state can be calculated unambiguously from the state of all other genes and metabolites.) Consider the following subnetwork of the iMH805 TRN for *S. cerevisiae *[16]:(1)

Transcription factor *mth1 *is repressed by *mig1 *and promotes expression of *rgt1*. Extracellular L-glutamine (gln-L) represses *rgt1 *expression. The original iMH805 study required this set of constraints be described with the following set of explicit rules [16]:

$$\begin{array}{r} \text{not MIG1}\Leftrightarrow mth1\;\\ \text{MTH1 and (not gln-L)}\Leftrightarrow rgt1 \end{array}$$

An implicit representation of (1) is

$$\begin{array}{lll} \text{MIG1} & \Rightarrow \text{not }mth1\; & \text{(1)}\\ \text{MTH1} & \Rightarrow rgt1\; & \text{(2)}\\ \text{g}\ln-\text{L} & \Rightarrow \text{not }rgt1\; & \text{(3)}\\ & \; & \text{(4)} \end{array}$$

The three transcription factors and one metabolite in these rules can be arranged in 2^4 ^= 16 possible states. As shown in Table 1, only four of the sixteen states are feasible for the explicit rules. The implicit formulation of the same system allows four new states and makes one of the explicit states infeasible. This example illustrates that two mathematical descriptions of the same biological process can lead to distinct model predictions. The model developer should be free to choose the rule formulation that best encompasses the underlying biology. However, implicit rules require simultaneous solution with a metabolic model and are often more difficult to parse into a mixed integer linear program. As a result, previous TRN integration studies have relied solely on explicit rules to describe regulatory interactions [17]. A software platform that can correctly parse both explicit and implicit rules would ease the development of large TRN models.

**Table 1:** Feasible states for explicit and implicit rules

State				Feasibility	
	
*mig1*	*mth1*	*rgt1*	gln-L	Explicit	Implicit
0	0	0	0		•
0	0	0	1		•
0	0	1	0		•
0	0	1	1		
0	1	0	0		
0	1	0	1	•	
0	1	1	0	•	•
0	1	1	1		
1	0	0	0	•	•
1	0	0	1	•	•
1	0	1	0		•
1	0	1	1		
1	1	0	0		
1	1	0	1		
1	1	1	0		
1	1	1	1		

Feasible states for explicit and implicit rules describing the transcriptional regulatory network (1). Rules are taken from the *S. cerevisiae *regulatory network model iMH805 [16]

## Objectives

Software suites have been developed to enable COBRA analyses. Packages such as CellNetAnalyzer [18], the BioMet Toolbox [19], and the COBRA Toolbox [20] implement several useful algorithms for studying COBRA models and TRNs. However, to date, no single software platform has been developed to 1.) convert COBRA models and TRNs into integrated optimization problems, 2.) analyze these integrated models with existing algorithms to incorporate high-throughput expression data, and 3.) allow users to easily develop new algorithms for the integrated models. To overcome these limitations, we present a Toolbox for Integrating Genome-scale metabolism, Expression, and Regulation (TIGER). TIGER automatically converts a list of implicit or explicit GPR and TRN rules into a set of linear inequalities; these equations are integrated with an existing COBRA model. The software allows rules to be written in a generalized Boolean format, enabling TRN logic to more accurately reflect the underlying biology. We demonstrate how this increased expressivity can overcome inconsistencies in existing TRN models. We will also show how TIGER simplifies the development of gene-centric extensions to FBA by improving three algorithms for integrating high-throughput expression data with a COBRA model.

## Implementation

**Figure 1:**
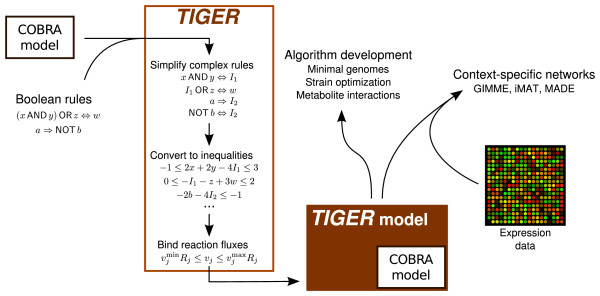
**TIGER platform overview**. TIGER converts Boolean rules to MILPs. Rules are first simplified by substitution and converted into a system of linear inequalities. The rules are optionally attached to a COBRA model by coupling indicators of reaction participation (*R_i_*) to the reaction flux *v_i_*. In addition to serving as a platform for developing new algorithms, TIGER models can be integrated with high-throughput expression data to generate context-specific models using variations on the GIMME, iMAT, and MADE algorithms.

The primary functions of TIGER are shown in Figure 1. TIGER converts a GPR and additional regulatory rules into an equivalent mixed integer linear program (MILP). The MILP constraints are added to a COBRA metabolic model to create a TIGER model that combines metabolism, GPR associations, and transcriptional regulation. This integrated model serves as a platform for applying many gene-centric extensions to FBA, including algorithms that incorporate "omics" data for model refinement. In this section, we describe how the rules parsed by TIGER are constructed, and how they are converted to an MILP. Sample files depicting a COBRA model, GPR, and TRN are provided in the "test/samples" directory of the TIGER distribution.

### Creating rules

**Figure 2:**
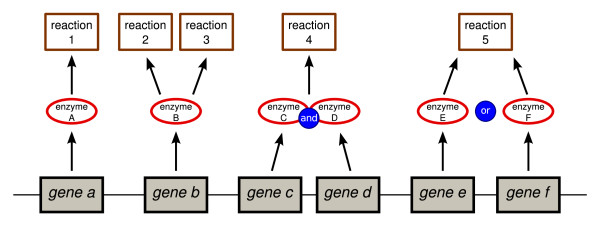
**The gene-protein-reaction relationship**. Boolean logic is used to describe gene-protein-reaction (GPR) relationships of varying complexity. Reactions can carry flux if and only if a correct combination of genes is expressed. Reaction 1 is catalyzed by a single gene product (reaction 1 ⇔ *gene a*). Reactions 2 and 3 are both catalyzed by the product of *gene b *(reaction 1 ⇔ *gene b*, reaction 2 ⇔ *gene b*). Genes *c *and *d *produce subunits of an enzymatic complex; both proteins are required for reaction 4 to carry flux (reaction 4 ⇔ *gene c *and*gene d*). Genes *e *and *f *encode isozymes. Either enzyme can catalyze reaction 5 (reaction 5 ⇔ *gene e *or *gene f *).

The GPR uses Boolean logic to describe the nonlinear relationship among genes, their protein products, and the reactions they catalyze. Examples of GPR relationships appear in Figure 2. The majority of metabolic reactions adhere to a one gene, one enzyme, one reaction relationship, as demonstrated by *gene a*, enzyme A, and reaction 1. If *gene a *is expressed, FBA assumes that reaction 1 can carry any physiologically feasible flux. Some enzymes, such as enzyme B, can catalyze two or more separate reactions. Other reactions, such as reaction 4, require two enzymes (C and D) to carry any flux. Enzymes C and D are commonly protein subunits of an enzymatic complex; if either is absent, no substrate conversion is possible. Genes *e *and *f *encode isozymes; either protein can independently catalyze reaction 5.

These GPR relationships can be described as a Boolean *expression *using the standard operators and and or. For example, a reaction that is catalyzed by either of two isozymes, the second of which is composed of two subunits, would have a GPR of the form "isozyme_1 _or (isozyme_2a _and isozyme_2b_)". Two expressions are joined with an implication operator (⇒ or ⇔ corresponding to "if" and "if and only if"), to form a *rule*. For GPR associations, rules are formed as "GPR ⇔ *reaction*", where *reaction *is an indicator variable that constrains the flux through a reaction to be zero when the GPR expression is false.

TIGER expressions allow additional features to describe logical relationships that are more complex than those typically found in GPRs. The not operator allows logical negation, which is often used to construct rules for transcriptional repression. Expressions can also contain *conditionals *that compare the numerical values of individual variables. If a gene *g *was known to be expressed when glucose uptake is greater than 10 flux units, this relationship could be represented by the rule "glc_ex *>*10 ⇒ *g*", where "glc_ex" is the glucose exchange reaction in the metabolic model. Any two expressions of arbitrary complexity can be combined as a rule and parsed by TIGER. The grammar used by TIGER for rules was designed to resemble logical operations in common programming languages and to be compatible with the GPRs of widely-used COBRA models. A complete description of the TIGER syntax appears in Additional File 1.

Some transcriptional regulators, such as the response of *crp *to cAMP in *E. coli*, display multiple levels of activity and cannot be easily described with Boolean logic [15]. Rather than require users to create several variables describing each state of activation, TIGER allows multilevel variables. If a transcription factor *t *activates target genes *g*_low _at low levels of expression and *g*_high _at high levels of expression, then this relationship could be described with the rules

(2) $$\left(t=1\right)\Rightarrow g_{1\text{ow}}$$

(3) $$\left(t=2\right)\Rightarrow g_{\text{high}}$$

where *t *= 0, 1, and 2 corresponds to no, low, and high expression. Logical operators have a different interpretation when applied to multilevel variables. If proteins *x *and *y *form a promoter complex for expression of gene *z*, then the corresponding rule for *z *expression would be "*x *and* y *⇒ *z*", since both *x *and *y *are required for *z *transcription. If *x *and *y *were multilevel, one would assume that *z *expression would be proportional to the promoter subunit in lower abundance, since this species would limit the amount of complete promoter complex that could be formed. Thus, the and operation often corresponds to a minimization:

(4) xandy≡ min{x,y}

The or operation would be used in situations where either factor can independently induce expression. In this situation, the species in higher abundance determines the target gene's transcription level. TIGER implements the multilevel or as a maximization:

(5) xory≡ max{x,y}

The not operator can have two interpretations when applied to multilevel variables:

(6) $$\text{not}\;x\equiv \left\{\begin{array}{c} x>0\;\text{pseudo - binary}\\ \bar{x}-x\;\text{inversion},x\in \left\{0,\ldots ,\bar{x}\right\} \end{array}\right)$$

where $x\in \left\{0,\ldots ,\bar{x}\right\}$. The first case (pseudo-binary) regards any nonzero value as true, regardless of the number of levels the variable may occupy. The second case (inversion) requires that the value of *x *and the quantity not*x *always sum to the maximum value that *x *can occupy. In this case, not*x *is a measure of how far *x *is from its upper bound. Users are able to select the pseudo-binary or inversion representation depending on which interpretation is a better approximation of the biological context. For example, consider a gene/repressor relationship *R *⇒ not* G*, where the repressor *R *can take on three biologically distinct levels - "off," "low," and "high". If both "low" and "high" levels of *R *prevent any expression of *G*, then the pseudo-binary not operator would be appropriate as *G *is off whenever *R *is not "off". However, if *G *also has the same three levels of expression, then the inversion interpretation of the not operator is more appropriate. This choice implies that

(7) R=off→G=high

(8) R=low→G=low

(9) R=high→G=off

Any variable in a TIGER model can be declared with multiple levels. Such declarations are made when rules are added to a model using the add_rule function in two ways: 1.) setting the default upper bound to all variables to any integer greater than one, or 2.) providing a list of variable names and a set of upper and lower bounds.

### Rule simplification

Simple Boolean rules can be represented by systems of linear inequalities of integer variables [21]. A general Boolean rule can be converted by the following procedure to a set of simple rules before conversion to an MILP.

We define an "atomic" expression as either a variable (*x*) or a negated variable (not* x*). If a not operator appears before an expression that is not atomic, TIGER applies DeMorgan's laws to move the negation onto atomic expressions (e.g., not (*x *and* y*) becomes the equivalent expression (not *x*) or (not* y*)). A simple rule then conforms to one of the following patterns

(10) x(⇒|⇔)z

(11) xand y(⇒|⇔)z

(12) xory(⇒|⇔)z

(13) x⟨op⟩y(⇒|⇔)z

where *x*, *y*, and *z *are atomic, and 〈*op*〉 is a conditional operator (≤, ≥, etc.). Non-simple rules are converted to simple rules through a series of recursive substitutions. For example, the rule

(14) (xor(not y))and z⇒w

is not simple, since the expression *x *or (not* y*) is not atomic. By defining an indicator variable *I*, which is true if and only if the expression *x *or (not* y*) is true, equation (14) can be written as two simple rules:

(15) xor(not y)⇔I

(16) Iand z⇒w

The bounds of *I *are determined by the bounds of *x *and *y*. If $x\in \left\{0,\ldots ,\bar{x}\right\}$ and y∈{0,…,ȳ}, then I∈{0,…,Ī}, where

(17) $$Ī=\left\{\begin{array}{cc} \max\left\{\bar{x},ȳ\right\} & \text{for }x\;\text{or}\;y\\ \min\left\{\bar{x},ȳ\right\} & \text{for }x\;\text{and}\;y \end{array}\right)$$

Thus, if *x *and *y *are binary, $\bar{x}=ȳ=1$, so *I *is binary as well.

TIGER applies the above substitutions recursively, creating indicator variables as necessary until all rules are simple. Each simple rule is converted to a set of linear inequalities that are added as constraints to the model structure. If a variable name already appears in the model, TIGER assumes that these variables represent the same quantity and thus allows new rules to be added to an existing model without recompiling previous rules. At the same time, TIGER creates variables to substitute for negated variables. For efficiency, TIGER ensures that only one negated variable is created for each original variable, regardless of the number of times the negated expression appears in the set of simple rules. Details of the conversion between simple rules and inequalities, along with methods for handling conditionals, are provided in Additional File 1.

### Reaction coupling

If the GPR expression for a reaction is not satisfied, the reaction is not allowed to carry flux. To enforce this relationship during an optimization, a set of discrete variables *R_i _*are defined, where *R_i _*= 0 if the GPR for reaction *i *is not satisfied, and *R_i _>*0 otherwise. To enforce the GPR's effect on flux, TIGER adds the constraint

(18) $$v_{i}^{\min}R_{i}\leq v_{i}\leq v_{i}^{\max}R_{i}$$

where *v_i _*is the flux through the *i*th reaction, with lower and upper bounds $v_{i}^{\min}$ and $v_{i}^{\max}$.

### Model structure

**Figure 3:**
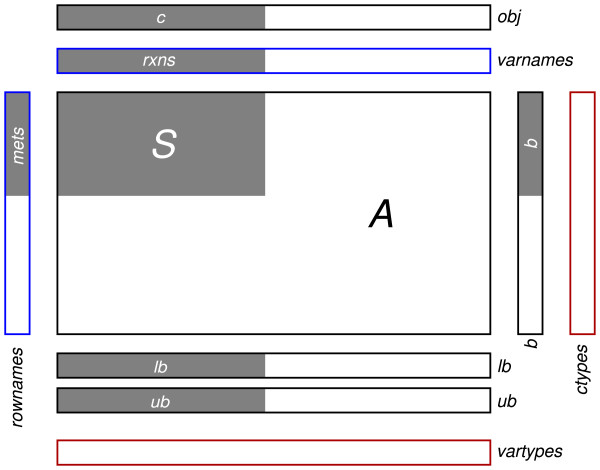
**Structure of TIGER models**. TIGER models are represented as Matlab structures. Boxes indicate size and orientation of the fields. Black text denotes TIGER field names. Gray areas contain data from the COBRA model, with white text indicating the relevant COBRA field names. Border color indicates data type: black → double-precision matrix, blue → cell array of strings, red → character array.

TIGER models are represented as Matlab structures. The layout of this structure is shown in Figure 3. The structure contains fields obj, A, b, lb, and ub that correspond to the values in the following MILP problem:

$$\begin{array}{c} \min obj^{\prime }x\\ \text{subject to}\\ \;\;Ax\left(\leq |=|\geq \right)b\\ \;\;lb\leq x\leq ub \end{array}$$

The type of (in)equality for each constraint in A is determined by the character vector ctype. The type of variable for each entry in *x *is specified by the field vartype, where 'c', 'b', and 'i' denote continuous, binary, and general integer variables. Reaction fluxes are continuous variables, while all other variables are either binary or integer depending on the corresponding upper bound. The fields rownames and varnames contain descriptive names of the constraints and variables, stored as cell arrays of strings. Functions in TIGER allow variables to be interchangeably referenced by their name, column index, or through Matlab's logical indexing features.

The format for TIGER models is designed for compatibility with the model structure for the COBRA Toolbox [20]. TIGER can use a COBRA Toolbox model as a starting point for converting a genome-scale reconstruction; therefore, any model in a file format supported by the COBRA Toolbox (SBML, Simpheny, etc.) can be converted to a TIGER model.

### Accessing the MILP solver

TIGER uses a custom Matlab class CMPI (Common Mathematical Programming Interface) to create and solve mathematical programming problems. CMPI defines a consistent structure for MILP (and mixed-integer quadratic programming, MIQP) problems, providing independence from the underlying MILP solver software. TIGER currently supports the CPLEX, Gurobi (via Gurobi MEX), and GLPK (via GLPK Mex) software packages, all of which are freely available for academic use. Porting TIGER to use a new solver requires modifying only the CMPI method solve_mip to specify the new interface. CMPI also provides a standardized method for configuring common solver parameters (maximum solution time, optimality and feasibility tolerances, etc.).

Previous work has indicated that the computation time of some FBA-related algorithms, such as Flux Variability Analysis [22], can be reduced by saving information about the problem structure between calls to the MILP solver [23]. CMPI provides a method, solve_multiple_milps, to preserve the solver state between successive calls to the CPLEX optimizer and reduce runtime in this manner. (Gurobi and GLPK currently do not support this feature in their Matlab interfaces.) If the CPLEX optimizer is not installed, CMPI will automatically make successive calls to the installed optimizer. While this removes the potential speed increase from using solver restarts, it allows TIGER code to remain solver independent and portable.

### Using TIGER

TIGER source code and installation instructions are available online at http://bme.virginia.edu/csbl/downloads/ or http://csbl.bitbucket.org/tiger The version of TIGER used for the examples in this study is included as Additional File 2. All functions in the toolbox are documented using Matlab's "help" facilities. Complete documentation and a step-by-step tutorial are also available on the TIGER website. The software includes a testing suite to verify the installation. These tests contain examples that build a TIGER structure from a simple COBRA model, add a set of TRN rules, call a MILP solver, and display the solution.

## Results and Discussion

### Refining integrated models for Saccharomyces cerevisiae

TIGER was used to couple the 1266 reactions in iND750 [9], a genome-scale model of *Saccharomyces cerevisiae *metabolism, with 750 metabolic genes. The resulting TIGER model contained 4498 constraints in 3214 variables. A model of *S. cerevisiae *transcriptional regulation, iMH805 [16], was added. The additional 805 rules contributed 1057 constraints and 562 variables to the TIGER model. The conversion took 53.66 s for iND750 and 20.31 s to add the TRN using an Intel 3.2 GHz i7-quad core processor running Linux.

As mentioned above, previous methods for integrating TRNs involve an iterative process, alternating between calculating gene states from a given environment and determining an environment based on metabolic byproducts [17]. However, the multiple layers of trascriptional regulation may require several iterations of this method to reach a stable gene state. The number of iterations to reach a stable state varies by environment and cannot easily be determined *a priori *[24]. In fact, some feedback mechanisms in TRNs may lead to a stable cycle of gene activation/inactivation rather than a single gene state. TIGER solves the TRN and FBA problems simultaneously, so the resulting gene state is always stable (or an optimal state inside a stable cycle).

Applying large-scale TRNs to COBRA models may result in infeasible models, i.e., models unable to produce any biomass. This is often due to a small number of rules that turn off reactions that are essential for biomass production. Previous work has developed techniques for finding which rules create the model infeasibility [13]. TIGER includes the function find_infeasible_rules to identify rules that prevent feasible solutions to the resulting MILP. Given a model and a set of rules that prevent a feasible solution, find_infeasible_rules creates a MILP that preserves the logic of the rules but allows each rule to be artificially satisfied. The objective of this MILP is to minimize the number of rules that must be artificially satisfied while finding a feasible solution for the model. (Details of this process are available in Additional File 1.)

Analysis by TIGER reported that the combined iND750/iMH805 metabolic and TRN network was unable to produce biomass under aerobic conditions in a glucose minimal media. Since *S. cerevisiae *is well-known to grow in this environment, we used TIGER's find_infeasible_rules function to identify the following three rules that prevented growth:

(19) $${\text{O}}_{2}\left[\text{e}\right]\;\text{or}\;\left(\text{not ROX1}\right)\Leftrightarrow hap1$$

(20) $${\text{O}}_{2}\left[\text{e}\right]\;\text{and}\;\text{HAP1}\Leftrightarrow rox1$$

(21) glucose[e]andHAP1and(not ROX1)⇔erg11

The product of gene *erg11*, lanosterol 14*α*-demethylase, is an essential enzyme for the production of ergosterol, a main sterol in *S. cerevisiae *[25]. This gene is essential in the iND750 metabolic model and must remain "on" during aerobic growth on glucose. However, the stable gene state for *erg11 *in the above rules is always "off" after three iterations, as described in Table 2. Because the iMH805 study used the results of the second iteration as the final gene state, this inaccuracy was unnoticed.

**Table 2:** Gene states for erg11 regulation

		Iteration				
			
	Start	1	2	3	4	Rule
O_2_[e]	1	1	1	1	1	
glucose [e]	1	1	1	1	1	
*hap1*	0	1	1	1	1	O_2_[e] or not ROX1
*rox1*	0	0	1	1	1	O_2_[e] and HAP1
*erg11*	1	1	1	0	0	O_2_[e] and HAP1 and (not ROX1)

*erg11*	1	1	1	1	1	O_2_[e] and HAP1 and (high_o2 or not ROX1)

To start, all transcription factors are assumed to be "off", and all metabolic genes are "on". The extracellular environment contains only glucose (glucose[e]), oxygen (O_2_[e]), and essential salts and minerals. Each iteration calculates the next gene state based on the current state of metabolites in the environment and transcription factors. After three iterations, expression of *erg11 *turns off, unless a modified rule is used that account for high oxygen uptake. The modified rule (shown below the double line) is more consistent with the results in Turi & Loper [25] Rules are taken from the *S. cerevisiae *regulatory network model iMH805 [16]

Rule (21) was originally derived from Turi & Loper [25]. Re-examination of this manuscript revealed that while ROX1 represses *erg11*, complete repression is only observed under low oxygen conditions. To incorporate these findings, we create an indicator "high o2" that is true if and only if the cell uptakes more than 10% of the maximum oxygen consumption rate. This relationship is expressed as

(22) high_02⇔EX_02(e)<-0.244

where "EX_o2(e)" is the iND750 name for the oxygen exchange reaction, and the maximum oxygen uptake rate for growth on glucose is 2.44 mol/(g dry cell weight)/h (negative flux through exchange reactions indicate uptake by the cell). Rule (21) for *erg11 *expression was re-written to only exhibit ROX1 repression under low oxygen conditions:

(23) glucose[e]and HAP1 and (high_o2 or not ROX1)⇔erg11

The set of refined rules (20,21,23) reproduces the correct growth phenotype in aerobic glucose conditions. This example demonstrates a three-step procedure for refining existing TRN models using TIGER: 1.) apply the existing TRN to a COBRA model, 2.) use the find_infeasible_rules function to identify rules that cause the model to differ from a known phenotype, and 3.) re-examine the evidence for these rules and make appropriate modifications. As shown in the previous example, new biological information can often be incorporated into existing rules using TIGER's support for complex logical expressions.

### Improved methods for expression data

Coupling the GPR with a metabolic model is a starting point for several algorithms designed to refine metabolic models by integrating high-throughput gene or protein expression data. The TIGER package contains implementations of three of these methods.

#### GIMME

GIMME was designed to generate context-specific metabolic networks by designating each reaction as "on" or "off" [26]. Given expression data and a minimum expression threshold, GIMME calculates a normalized gene "score" for each reaction by averaging the expression values of all genes that appear in the GPR for the reaction. Reactions with a score below a cutoff are turned off; only reactions scoring above the cutoff are allowed to carry flux. Because this thresholding does not guarantee a functioning model, an optimization problem is formed to minimize the number of "off" reactions that must carry flux when the model produces a minimum objective flux.

The GIMME algorithm is an excellent candidate for conversion to a gene-centric approach. Rather than average the gene expression values for each reaction, a simpler approach is to turn genes "on" or "off" if their expression is above or below a threshold. Using an integrated GPR/metabolic model, an optimization problem could re-activate "off" genes to allow the network to produce an objective flux. TIGER provides a gimme function that implements this gene-centric approach. Similar to the original algorithm, TIGER's GIMME uses the distance below the expression threshold as a weight when selecting genes for re-activation. In addition to removing discrepancies caused by the averaging of gene expression values over each reaction, TIGER's integrated approach allows GIMME to identify each gene as either "on" or "off" in the resulting network (GIMME originally reported only the state of each reaction).

**Figure 4:**
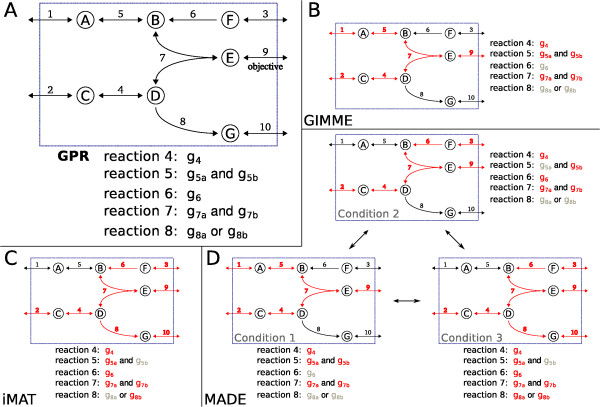
**Integrating expression data with a prototypic network model**. **A**. Prototypic network model. Production of metabolite E is the metabolic objective (reaction 9). Reactions 4 and 6 are catalyzed by a single enzyme. Reactions 5 and 7 require enzymes composed of two necessary subunits. Reaction 8 can be catalyzed by either of two isozymes. **B **- **D **display the results of integrating expression data in Tables 3 - 5. Red indicates that a gene (reaction) is expressed (active).

We applied GIMME to the prototypic network shown in Figure 4a. In this network, three metabolites, A, C, and F, are transported into the system and undergo chemical transformation into products E and G. The metabolic objective for this system is the production of E. The GPR associations are also shown in Figure 4a. Reactions 4 through 8 are gene associated. Reactions 5 and 7 both require two enzymatic subunits to carry flux, and reaction 8 can carry flux through either of two isozymes.

The expression data applied to this network are shown in Table 3. Using simple thresholding at a threshold expression level of 10 does not produce a functional model. As shown in Table 3 and Figure 4b, GIMME reactivates the genes *g*5a and *g*7b. Because reaction 7 is the only method of producing metabolite E (the metabolic objective), this reaction must be active and both subunits (*g*_7a _and *g*_7b_) must be expressed. Production of E also requires metabolite B and either reaction 5 or reaction 6. The expression data showed that both genes *g*_5a _and *g*_6 _were below the expression threshold; GIMME chose to activate *g*_5a _since its expression was closer to the threshold.

**Table 3:** Expression data for GIMME example

Gene	Expression	Simple thresholding	GIMME
*g*_4_	13	1	1
*g*_5a_	6	0	1
*g*_5b_	12	1	1
*g*_6_	2	0	0
*g*_7a_	18	1	1
*g*_7b_	6	0	1
*g*_8a_	2	0	0
*g*_8b_	4	0	0

**Figure 5:**
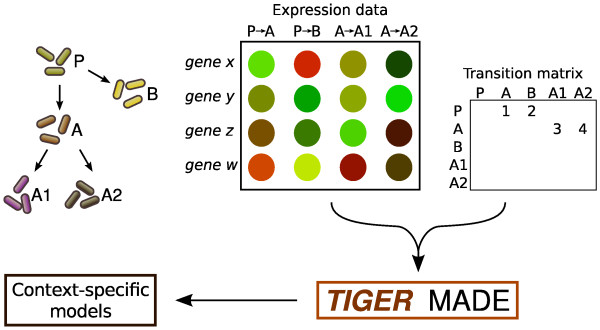
**Conceptual example using the enhanced MADE algorithm**. Workflow for integrating expression data from evolved bacterial cultures using TIGER's enhanced MADE algorithm. Parent strain *P *splits into strains *A *and *B*. Strain *A *further diverges into strains *A*1 and *A*2. Expression data indicates fold change between different strains for each gene. The interaction matrix indicates that the first column of fold changes represents a change from *P *to *A*, the second column from *P *to *B*, etc. The expression data and the interaction matrix are combined to create gene states for each bacterial strain.

Expression values for genes of the network shown in Figure 5a. "Simple thresholding" is the gene state after a simple threshold of 10 is applied to the expression values. "GIMME" is the gene state calculated by GIMME that produces a functioning model. All expression values are arbitrary units.

#### iMAT

Shlomi, *et al*. [27], introduced the iMAT algorithm to create tissue-specific models of mammalian metabolism. Briefly, reactions or genes were classified as having either low, regular, or high activity using information from several databases. An optimization problem was formulated such that

(24) $$v_{i}\left\{\begin{array}{c} \geq \varepsilon \;\text{if reaction }i\;\text{has high activity}\\ \leq \varepsilon \;\text{if reaction }i\;\text{has low activity} \end{array}\right)$$

for each reaction flux *v_i _*and some small flux *ε*. The optimization attempted to preserve the reaction classifications while enforcing that the resulting set of reaction fluxes be feasible (mass balanced). The mass-balance approach attempts to yield functional models in multicellular organisms that lack a clearly defined metabolic objective.

TIGER includes a gene-centric version of the iMAT algorithm. Because TIGER allows multilevel variables, each gene in the GPR is allowed to occupy a state of low (0), medium (1), or high (2) activity. The multilevel operators in the GPR then combine the gene expression values to create a reaction indicator with the same three levels. Rules are added to enforce the constraints in equation (24). Additionally, if a metabolic objective is available for the organism, the flux through the corresponding objective reaction can be constrained above a minimum value.

iMAT was used to integrate the activity data in Table 4 with the network model in Figure 4a. The results are shown in Figure 4c. Notice that the high activity of gene *g*_8b _requires that reactions 8 and 10 carry flux. Because production of metabolite G is not a part of the system's metabolic objective, reaction 8 would most likely not carry flux in the FBA flux distribution for this network; the activity seen in this side pathway is enforced by the iMAT constraints.

**Table 4:** Activity levels for iMAT example

Gene	Activity level
*g*_4_	medium
*g*_5a_	low
*g*_5b_	low
*g*_6_	high
*g*_7a_	high
*g*_7b_	high
*g*_8a_	medium
*g*_8b_	high

Classification of genes according to "low", "medium", or "high" activity for iMAT. Genes correspond to the network shown in Figure 5a. iMAT produces the integrated network shown in Figure 5c.

#### MADE

Metabolic Adjustment by Differential Expression (MADE) removes the need for a pre-defined "on"/"off" threshold when integrating expression data [12]. Instead, MADE uses the differential expression between two or more conditions to determine which genes or proteins are likely to be "on" or "off". If a gene increases significantly between conditions 1 and 2, MADE attempts to turn the gene "off" in condition 1 and "on" in condition 2. The expression data are mapped while ensuring model functionality in all conditions, and the statistical significance of the expression changes are used to prioritize discrepancies.

The TIGER implementation of MADE offers two improvements. First, genes are allowed to be multilevel instead of binary. The user is allowed to define a mapping between the multiple levels of gene expression and the flux constraints for the corresponding reactions. Second, TIGER MADE allows comparisons among states that do not appear as a linear sequence. The original MADE algorithm used a series of *n *conditions *C*_1_, *C*_2_, ..., *C_n_*, and *n *- 1 sets of expression data describing the changes *C*_1 _→ *C*_2_, *C*_2 _→ *C*_3_, ..., C_*n*-1 _→ *C_n_*. TIGER MADE allows any number of connections between the *n *states.

A conceptual example of TIGER MADE's capabilities is presented in Figure 5. The goal in this example is to use expression data from five bacterial strains to develop strain-specific metabolic models. Strains *A *and *B *were evolved from the same parent strain *P*. Strain *A *later gave rise to two additional strains, *A*1 and *A*2. A matrix containing the differential expression between each strain and its predecessor is calculated. A transition matrix *T *is defined as follows: if *T*(*i*, *j*) = *k*, then the *k*th column of the differential expression matrix was calculated between strains *i *and *j*. Values greater than one in this column indicate that expression is higher in strain *j *than in strain *i*. These two matrices are used by TIGER MADE to create a single optimization problem. The result is a functional gene state for each condition that maximizes the significant changes in gene expression between each strain and its parent.

Figure 4d shows the results of applying MADE on three network models simultaneously. Each resulting model is functional and captures the most significant differences described the fold changes listed in Table 5. Notice that despite significant changes in enzyme expression for reactions 4 and 7, these reactions remain "on" in all models because they are required to produce metabolite E, the metabolic objective.

**Table 5:** Expression data for MADE example

	Fold change						MADE states		
		
Gene	1 → 2	*P*	2 → 3	*P*	3 → 1	*P*	1	2	3
*g*_4_	2.1	0.68	3.0	0.08	0.7	0.15	1	1	1
*g*_5a_	0.5	0.45	8.0	0.44	0.8	0.22	1	0	1
*g*_5b_	1.1	0.07	0.1	0.49	2.2	0.03	1	1	0
*g*_6_	3.6	0.05	1.6	0.22	0.4	0.48	0	1	1
*g*_7a_	1.4	0.38	4.2	0.40	1.8	0.43	1	1	1
*g*_7b_	2.3	0.04	0.4	0.19	0.3	0.12	1	1	1
*g*_8a_	1.3	0.40	7.3	0.21	0.2	0.45	0	0	1
*g*_8b_	0.9	0.89	4.1	0.83	0.1	0.28	0	0	1

Fold change and *P*-values for the MADE example. Column "1 → 2" and indicates the fold change in expression between conditions 1 and 2; *P *is the *P*-value for this change. "MADE states" are the binary gene states returned by the algorithm. The states maximize the transitions in the expression data while retaining model functionality in each condition. Gene names correspond to the network in Figure 5a. Results are also depicted in Figure 5d.

Source code for all of the examples in Figure 4 are available in the file test/gimme_imat_made_examples.m of the TIGER distribution.

## Conclusions

We have presented TIGER, a software platform for converting generalized Boolean and multilevel rules to mixed-integer linear programs, and coupling these rules to genome-scale models of metabolism. The flexibility of TIGER's generalized rule format allows for a more accurate description of biological processes such as catalysis by isozymes and multi-meric proteins, metabolic flux control, and transcriptional regulation. These features were used to identify and correct inconsistencies within an existing TRN model of *Saccharomyces cerevisiae*. We have also demonstrated how TIGER can be used as a starting point for implementing and improving existing algorithms for genome-scale analysis.

In addition to adding implementations of other gene-centric algorithms to TIGER, we are exploring methods to improve the solution efficiency of the generated MILP. Possible strategies include exploiting indicator constraints, specially-ordered-sets (SOS), and other solver optimizations through CMPI.

## Availability and requirements

**Project name: **TIGER

**Project home page: **http://bme.virginia.edu/csbl/downloads or http://csbl.bitbucket.org/tiger

**Operating system: **Platform independent

**Programming language: **Matlab

**Other requirements: **Matlab v7.0 or higher, a Mixed-Integer Linear Programming solver (e.g. CPLEX, Gurobi, or GLPK)

**License: **MIT

**Non-academic use restrictions: **None

## List of abbreviations used

TIGER: Toolbox for Integrating Genome-scale metabolism, Expression, and Regulation; COBRA: COnstraint-Based Reconstruction and Analysis; FBA: Flux Balance Analysis; GPR: Gene-Protein-Reaction; TRN: Transcriptional Regulatory Network; MILP: Mixed-Integer Linear Program; SR-FBA: Steady-State Regulatory FBA; iND750: Genome-scale model of *Saccharomyces cerevisiae *metabolism; iMH805: Genome-scale transcriptional regulatory network for *Saccharomyces cerevisiae*.; CMPI: Common Mathematical Programming Interface; GIMME: Gene Inactivity Moderated by Metabolism and Expression; iMAT: Integrative Metabolic Analysis Tool; MADE: Metabolic Adjustment by Differential Expression.

## Authors' contributions

PAJ designed and wrote the TIGER software and performed the simulations. KAL prepared the TRN validation study and contributed TIGER functions. The project was conceived by PAJ and JP, and advised by JP. All authors wrote and reviewed the manuscript.

## Supplementary Material

Additional file 1 **Supplementary Methods**. Detailed grammar for TIGER rules and specifics on the rule to MILP conversion process. Click here for file

Additional file 2 **TIGER source code**. Source code, documentation, and tutorials are also available online at http://bme.virginia.edu/csbl/downloads/ or http://csbl.bitbucket.org/tiger. Click here for file
