# Supplementary material for: TIGER: Toolbox for integrating genome-scale metabolic models, expression data, and transcriptional regulatory networks
Source: BMC Syst Biol. 2011 Sep 23;5:147. doi: 10.1186/1752-0509-5-147 (PMC3224351; doi:10.1186/1752-0509-5-147)
Supplement: Additional file 1 — Supplementary Methods. Detailed grammar for TIGER rules and specifics on the rule to MILP conversion process. [file 1752-0509-5-147-S1.PDF]

Supplementary Methods

Complete TIGER Grammar

This section describes the syntax used for all rules that can be parsed by TIGER. The grammar was designed to resemble Boolean operations in common programming languages.

Expressions are composed of *atoms* and *numerics* joined by *operators*. Atoms are either

- Identifiers composed of a sequence of any characters except `~!&|()<>=` or whitespace. Identifiers are case sensitive and cannot overlap with an operator (see below).
- Quoted strings of any characters. Either single (`'`) or double (`"`) quotes may be used. Quotation marks appearing inside a quoted string may be escaped with a backslash character (\).

Numerics are integer or floating point numbers that appear in conditionals. Scientific notation is allowed in the forms '`1e-10`' or '`-2E5`'. Some metabolic models use gene identifiers that could be interpreted as numbers, e.g. the Entrez gene identifier. TIGER allows two solutions to this potential ambiguity. First, numbers in quotation marks are interpreted as atoms. Second, the `parse_string` function allows numeric parsing to be turned off for all identifiers.

Two atoms or numerics can also be compared using the comparison operators `=`, `>`, `<`, `>=`, `<=`, and `~=`. The expressions are called *conditionals* and correspond to a value of one (true) if the condition is satisfied, and a zero (false) otherwise.

Atoms and conditionals are joined by the Boolean `AND`, `OR`, and `NOT` operators to create *expressions* of arbitrary complexity. Two expressions are joined by the `=>` (if) or `<=>` (if and only if) operators to form a *rule*.

The TIGER operators obey the following order of precedence:

highest	`NOT`
	`=`, `>`, `<`, `>=`, `<=`, and `~=`
	`AND`, `OR`
lowest	`=>`, `<=>`

All operators are left-associative. Parentheses can be used to change the order of operations in rules. Without parenthesis, the following expressions are parsed as shown

```
    a AND b AND c   →   (a AND b) AND c
       a > 2 OR b   →   (a > 2) OR b
      NOT a AND b   →   (NOT a) AND b
       NOT a <= 3   →   (NOT a) <= 3
a AND NOT b OR c    →   (a AND (NOT b)) OR c
```

The `add_rule` function allows two different interpretations of the NOT operator when applied to a multilevel variable. The *binary* form considers any nonzero value of x to be true, while the *inverted* form requires that $x + \text{NOT } x = x^{\max}$. The differences are described in the following table for $x \in \{0, 1, 2, 3\}$:

x	NOT x, binary	NOT x, inverted
0	1	3
1	0	2
2	0	1
3	0	0

Notice that for binary variables, both forms are equivalent.

In order to increase the number of existing COBRA models that can be parsed by TIGER without modification, synonyms are allowed for the following operators:

<table>
<tr><th>Operator</th><th>Other allowed forms</th></tr>
<tr><td>AND</td><td>and, &, &&</td></tr>
<tr><td>OR</td><td>or, |, ||</td></tr>
<tr><td>NOT</td><td>not, ~, !</td></tr>
<tr><td>=></td><td>->, ==>, -->, if, IF</td></tr>
<tr><td><=></td><td><->, <==>, <-->, iff, IFF</td></tr>
<tr><td>=</td><td>==</td></tr>
<tr><td>~=</td><td>!=, <></td></tr>
</table>

The following formal grammar incorporates the above information and describes all rules that can be parsed and translated by the TIGER platform.

$$
\begin{aligned}
\textit{rule} &\rightarrow \textit{expr}\ (\Rightarrow \mid \Leftrightarrow)\ \textit{expr} \\
\textit{expr} &\rightarrow \text{NOT}\ \textit{expr} \\
&\mid\ \textit{expr}\ \text{AND}\ \textit{expr} \\
&\mid\ \textit{expr}\ \text{OR}\ \textit{expr} \\
&\mid\ \textit{cond} \\
&\mid\ \textit{id} \\
\textit{cond} &\rightarrow \textit{id}\ \textit{op}\ (\textit{id} \mid \textit{number}) \\
\textit{op} &\rightarrow (\leq \mid < \mid = \mid \sim= \mid > \mid \geq)
\end{aligned}
$$

```
number  →  [+-]?\d+\.?\d*([eE]+[+-]?\d+)?
id      →  [^~!&|()<>= ]+
        |  "([^"]|\")+"
        |  '([^']|\')+'
```

Converting Rules to Linear Inequalities

As described in the main text, TIGER allows generalized rules of the form

$$f(x, y, \ldots) \quad (\Rightarrow \mid \Leftrightarrow) \quad g(x, y, \ldots) \tag{1}$$

We define an atomic expression as either x or $\mathsf{NOT}\ x$, where x is a discrete variable. If $g(x, y, \ldots)$ is not atomic, we define an indicator variable I such that

$$f(x, y, \ldots)(\Rightarrow \mid \Leftrightarrow)\, I \tag{2}$$

$$g(x, y, \ldots) \Leftrightarrow I \tag{3}$$

Using the recursive substitution procedure outlined in the text, rules (2) and (3) can be reduced to one of the following forms:

$$x \quad (\Rightarrow \mid \Leftrightarrow) \quad I \tag{4}$$

$$x\ \mathsf{AND}\ y \quad (\Rightarrow \mid \Leftrightarrow) \quad I \tag{5}$$

$$x\ \mathsf{OR}\ y \quad (\Rightarrow \mid \Leftrightarrow) \quad I \tag{6}$$

$$x\, \langle op \rangle\, y \quad (\Rightarrow \mid \Leftrightarrow) \quad I \tag{7}$$

where x, y, and I are all atomic. We now describe how each of these cases is converted into a set of linear inequalities.

Binary variables

If x and y are binary, the following transformations are used:

$$
\begin{aligned}
x \Rightarrow I &\quad\rightarrow\quad I \geq x \\
x \Leftrightarrow I &\quad\rightarrow\quad I = x \\
x\ \mathsf{AND}\ y \Rightarrow I &\quad\rightarrow\quad 2x + 2y - 4I \leq 3 \\
x\ \mathsf{AND}\ y \Leftrightarrow I &\quad\rightarrow\quad \begin{cases} x\ \mathsf{AND}\ y \Rightarrow I \\ 2x + 2y - 4I \geq -1 \end{cases} \\
x\ \mathsf{OR}\ y \Rightarrow I &\quad\rightarrow\quad -x - y + 3I \geq 0 \\
x\ \mathsf{OR}\ y \Leftrightarrow I &\quad\rightarrow\quad \begin{cases} x\ \mathsf{OR}\ y \Rightarrow I \\ -x - y + 3I \leq 2 \end{cases}
\end{aligned}
$$

Multilevel or continuous variables

The following transformations are used when either x or y are positive integers or continuous real values. If both x and y are discrete ($x \in \{0, \ldots, x^{\max}\}$, $y \in \{0, \ldots, y^{\max}\}$), I will be discrete and valued in the set $\{0, \ldots, I^{\max}\}$. If either x or y is continuous with $x \in [x^{\min}, x^{\max}]$ and $y \in [y^{\min}, y^{\max}]$, then I is a continuous variables with bounds $[I^{\min}, I^{\max}]$.

The rules $x \Rightarrow I$ and $x \Leftrightarrow I$ follow the same transformations as in the binary case. The junction operators are interpreted as

$$\begin{aligned} x \text{ OR } y \Rightarrow I &\quad\rightarrow\quad I \geq \max\{x, y\} \\ x \text{ OR } y \Leftrightarrow I &\quad\rightarrow\quad I = \max\{x, y\} \\ x \text{ AND } y \Rightarrow I &\quad\rightarrow\quad I \geq \min\{x, y\} \\ x \text{ AND } y \Leftrightarrow I &\quad\rightarrow\quad I = \min\{x, y\} \end{aligned}$$

These transformations are implemented with the following inequalities:

$$x \text{ OR } y \Rightarrow I \quad\rightarrow\quad \begin{cases} I \geq x \\ I \geq y \end{cases}$$

$$x \text{ OR } y \Leftrightarrow I \quad\rightarrow\quad \begin{cases} x \text{ OR } y \Rightarrow I \\ x > y \Leftrightarrow I_{\text{aux}} \\ I \leq x + (x^{\max} - x^{\min})(1 - I_{\text{aux}}) \\ I \leq y + (y^{\max} - y^{\min}) I_{\text{aux}} \end{cases}$$

$$x \text{ AND } y \Rightarrow I \quad\rightarrow\quad \begin{cases} x > y \Leftrightarrow I_{\text{aux}} \\ x - (x^{\max} - x^{\min}) I_{\text{aux}} \leq I \\ y - (y^{\max} - y^{\min})(1 - I_{\text{aux}}) \leq I \end{cases}$$

$$x \text{ AND } y \Leftrightarrow I \quad\rightarrow\quad \begin{cases} x \text{ AND } y \Rightarrow I \\ I \leq x \\ I \leq y \end{cases}$$

where the axillary variable I_{aux} is binary. The bounds on I are

	$I^{\min}$	$I^{\max}$
x OR $y \Rightarrow I$	$\max\{x^{\min}, y^{\min}\}$	$\max\{x^{\max}, y^{\max}\}$
x OR $y \Leftrightarrow I$	$\max\{x^{\min}, y^{\min}\}$	$\max\{x^{\max}, y^{\max}\}$
x AND $y \Rightarrow I$	$\min\{x^{\min}, y^{\min}\}$	$\max\{x^{\max}, y^{\max}\}$
x AND $y \Leftrightarrow I$	$\min\{x^{\min}, y^{\min}\}$	$\min\{x^{\max}, y^{\max}\}$

Conditionals

Consider the simple rule

$$\sum_i \phi_i x_i \geq k \Rightarrow I \tag{8}$$

that is a linear inequality in terms of variables x_i with constant coefficients ϕ_i. We convert this expression to an equivalent set of rules

$$\sum_i \phi_i x_i + s = k \tag{9}$$

$$s \leq 0 \Rightarrow I \tag{10}$$

where s is a continuous *slack variable* that is strictly positive if and only if $\sum_i \phi_i x_i < k$. We can represent rule (10) with the inequality $I + s > 0$. Because MILP solvers do not allow strict inequalities, this constraint is implemented as $I + s \geq \epsilon$, where ϵ is a very small, positive number.

The upper and lower bounds, u and l, on the quantity $\sum_i \phi_i x_i$ are

$$u = \sum_i \max\{\phi_i x_i^{\max}, \phi_i x_i^{\min}\}$$
$$l = \sum_i \min\{\phi_i x_i^{\max}, \phi_i x_i^{\min}\}$$

Thus, the bounds $s \in [k-u, k-l]$ are sufficient to allow the slack variable s to always satisfy the constraint (9).

To represent the rule

$$\sum_i \phi_i x_i \geq k \Leftrightarrow I \tag{11}$$

we begin by following the above procedure and add constraints (9) and (10). Then, to enforce that $I \Rightarrow s \leq 0$, we require that

$$s \leq (k-l)(1-I) \tag{12}$$

This completes the "if and only if" implication.

Finally, we show that all conditionals parsed by TIGER can be represented in terms of rules (8) and (11) through the following substitutions:

$$\begin{aligned}
x > y &\quad\rightarrow\quad \textsf{NOT}\ (-x \geq -y) \\
x < y &\quad\rightarrow\quad \textsf{NOT}\ (x \geq y) \\
x \leq y &\quad\rightarrow\quad -x \geq -y \\
x = y &\quad\rightarrow\quad (x \geq y)\ \textsf{AND}\ (-x \geq -y) \\
x \neq y &\quad\rightarrow\quad \textsf{NOT}\ ((x \geq y)\ \textsf{AND}\ (-x \geq -y))
\end{aligned}$$

The difference operator

Some algorithms require the inclusion of the nonlinear absolute value operator, often to measure the difference between two variables, $|x - y|$. TIGER includes the `add_diff` function that adds a variable $d = |x - y|$. If x and y are binary, then

$$d = x\ \textsf{XOR}\ y = (x\ \textsf{OR}\ y)\ \textsf{AND}\ (\textsf{NOT}\ (x\ \textsf{AND}\ y)) \tag{13}$$

If either x or y is continuous or multilevel, then d is constructed as the maximum of $x - y$ and $y - x$. This is represented by the following constraints:

$$\begin{aligned}
f^+ &= x - y, \quad f^+ \in [x^{\min} - y^{\max}, x^{\max} - y^{\min}] \\
f^- &= y - x, \quad f^- \in [y^{\min} - x^{\max}, y^{\max} - x^{\min}] \\
d &= \max\{f^+, f^-\} \\
&= f^+\ \textsf{OR}\ f^-
\end{aligned}$$

Finding Infeasible Rules

Consider a feasible TIGER model M and a set of rules R that, when added to M, make the resulting problem infeasible. We can find a minimal set of rules in R that are inconsistent though the following procedure.

Each rule in R is of the form

$$p \Rightarrow q \tag{14}$$

or

$$p \Leftrightarrow q \tag{15}$$

We create a set S of *artificially satisfiable rules* by taking each rule in R and adding a set of Boolean variables as follows:

$$\begin{aligned} p \Rightarrow q \quad &\rightarrow \quad p \Rightarrow q \text{ OR } r_i \\ p \Leftrightarrow q \quad &\rightarrow \quad p \text{ OR } l_i \Leftrightarrow q \text{ OR } r_i \end{aligned}$$

These rules are artificially satisfiable since setting all variables l_i and r_i to true will satisfy every rule in R. If all l_i and r_i are set to false, then the original logic in R is preserved, i.e. $R = S|_{l_i, r_i = 0}$. A MILP is then formulated as

$$\begin{aligned} &\min \sum_i l_i + \sum_i r_i \\ &\text{subject to} \\ &\quad M, S \end{aligned}$$

Any rule containing a true l_i or r_i in the optimal solution of this problem is returned by TIGER as part of the set of infeasible rules. The `find_infeasible_rules` function also reports whether each infeasible rule was artificially satisfied on the left (l_i true) or right (r_i true) side.
