# Supplementary material for: TIGER: Toolbox for integrating genome-scale metabolic models, expression data, and transcriptional regulatory networks
Source: BMC Syst Biol. 2011 Sep 23;5:147. doi: 10.1186/1752-0509-5-147 (PMC3224351; doi:10.1186/1752-0509-5-147)
Supplement: Additional file 2 — TIGER source code. Source code, documentation, and tutorials are also available online at http://bme.virginia.edu/csbl/downloads/ or http://csbl.bitbucket.org/tiger. [file 1752-0509-5-147-S2.GZ › tiger/doc/tutorial.pdf]

TIGER Quick Start

Version 1.0

April 6, 2011

This tutorial provides a brief overview of some commands in the TIGER toolbox. Details on each command can be found by typing "`help` *command*" at the Matlab prompt. The tutorial begins by explaining how rules are written, parsed, and added to TIGER models. After describing how TIGER models are solved, we demonstrate TIGER's features for converting a COBRA model directly. Finally, we use TIGER for an advanced example – calculating a minimal genome for a model system.

Rules

TIGER provides an easy way to 1.) convert Boolean expressions and rules into constraint-based problems, and 2.) couple these constraints to metabolic models. The TIGER toolbox parses rules using the `parse_string` command.

```
>> e = parse_string('a or b => c');
>> e
   OR
    |--- a    ==>    c
    |
    +--- b
>> class(e)
ans =
expr
```

After being parsed, rules are stored in Matlab as an object of the custom `expr` class. When displayed at in the command window, `expr` objects are shown as tree structures. Rules are written as two Boolean expressions joined with either the `=>` (if) or `<=>` (if and only if) operators. The rule `a <=> b` states that if `a` is true then `b` is true, and if `a` is false then `b` is also false. The rule `a => b` implies that `b` is true when `a` is true, but `b` is not necessarily false when `a` is false.

Expressions are written using the standard Boolean operators `and`, `or`, and `not` (or their shorthand symbols, `&`, `|`, and `~`). Parentheses are used to group complex expressions.

```
>> parse_string('a and (b or c) => d')
   AND
    |--- a
    |
    +--- OR         ==>    d
          |--- b
          |
          +--- c
>> parse_string('(a and b) or c => d')
   OR
    |--- AND
    |     |--- a
    |     |         ==>    d
    |     +--- b
    |
    +--- c
```

The `not` operator binds more tightly than the other operators:

```
>> parse_string('not a and b => c')
   AND
    |-- ~a    ==>    c
    |
    +--- b
>> parse_string('not (a and b) => c')
  ~AND
    |--- a    ==>    c
    |
    +--- b
```

The **TIGER** structure

TIGER converts Boolean rules into an equivalent mixed-integer linear program (MILP) of the form

$$
\begin{aligned}
&\min\ obj'x \\
&\text{subject to} \\
&\qquad Ax\,(\leq \mid = \mid \geq)\,b \\
&\qquad lb \leq x \leq ub
\end{aligned}
$$

The MILP is stored as a Matlab structure. An empty structure (one without any rules added to it) is created with the `create_empty_tiger` command:

```
>> tiger = create_empty_tiger()
tiger =
```

```
          A: []
          b: []
         lb: []
         ub: []
        obj: []
   varnames:
   rownames:
     ctypes: ''
   vartypes: ''
        ind: []
   indtypes: ''
      param: [1x1 struct]
```

The fields `obj`, `A`, `b`, `lb`, and `ub` correspond directly to the variables in the above MILP. The cells `varnames` and `rownames` hold names for each variable and constraint in the model. The character array `ctypes` identifies the type of constraint ($\leq$, $=$, or $\geq$) for each row in A. The field *vartypes* describes each variable as continuous (`c`), binary (`b`), or a positive integer (`i`). The remaining fields are used internally by TIGER functions.

Adding rules

The function `add_rule` accepts a TIGER structure and an `expr` object. It returns a new structure that includes constraints that encode the Boolean logic of the added rule.

```
>> empty = create_empty_tiger();
>> e = parse_string('a and b <=> c');
>> tiger = add_rule(empty,e)
tiger =
          A: [2x3 double]
          b: [2x1 double]
         lb: [3x1 double]
         ub: [3x1 double]
        obj: [3x1 double]
   varnames: 3x1 cell
   rownames: 2x1 cell
     ctypes: [2x1 char]
   vartypes: [3x1 char]
        ind: [2x1 double]
   indtypes: [2x1 char]
      param: [1x1 struct]
```

The rule `'a and b <=> c'` was converted to two constraints, and the three variables `a`, `b`, and `c` were added to the structure.

```
>> tiger.varnames
```

```
ans =
    'a'
    'b'
    'c'
```

Another rule can be added to the structure with an additional call to `add_rule`. (Notice that `add_rule` can accept a rule as a string and will make an appropriate call to `parse_string`.)

```
>> tiger = add_rule(tiger,'a or (x and y) => c')
tiger =
           A: [5x6 double]
           b: [5x1 double]
          lb: [6x1 double]
          ub: [6x1 double]
         obj: [6x1 double]
    varnames: 6x1 cell
    rownames: 5x1 cell
      ctypes: [5x1 char]
    vartypes: [6x1 char]
         ind: [5x1 double]
    indtypes: [5x1 char]
       param: [1x1 struct]
>> tiger.varnames
ans =
    'a'
    'b'
    'c'
    'x'
    'y'
    'I0001'
```

The structure `tiger` now contains five constraints and six variables. Besides adding the variables `x` and `y` found in the new rule, an *indicator variable*, `I0001`, was added. Indicator variables are sometimes needed to represent more complex rules as a linear inequalities; they are added as necessary by TIGER.

Solving models

TIGER models can be solved using the `solve_tiger` command. This command calls an external MILP solver to find a feasible state for each variable in x that optimizes the objective $obj'x$. (If the `obj` vector is all zeros, the first feasible solution is returned.)

```
>> sol = solve_tiger(tiger)
sol =
         x: [6x1 double]
```

```
      val: 0
   output: [1x1 struct]
     flag: 2
```

The result of `solve_tiger` is a solution structure that contains the solution vector (`x`), an objective value (`val`), and messages from the external solver (`output` and `flag`). An easy method to display the values for each variable in the model is the `show_sol` function.

```
>> show_sol(tiger,sol,'all')
         a:  1
         b:  1
         c:  1
         x:  1
         y:  0
     I0001:  0
```

Before solving a model, the `set_var` function can be used to specify the value of variables. We can use `set_var` to find a feasible solution where the variable `a` is zero.

```
>> no_a = set_var(tiger,'a',0);
>> sol = solve_tiger(no_a);
>> show_sol(no_a,sol,'all')
         a:  0
         b:  0
         c:  0
         x:  1
         y:  0
     I0001:  0
```

Remember that TIGER functions, including `set_var`, do not modify the model structure in place. Instead, they return a modified model structure, which was assigned to `no_a` in the previous example.

Adding a COBRA model

TIGER was designed to simplify the process of adding Boolean constraints to models for the COBRA toolbox. Figure 1 shows a simple metabolic network. This system can be described by a COBRA Toolbox structure:

```
>> cobra
cobra =
          S: [4x5 double]
          c: [5x1 double]
         lb: [5x1 double]
```

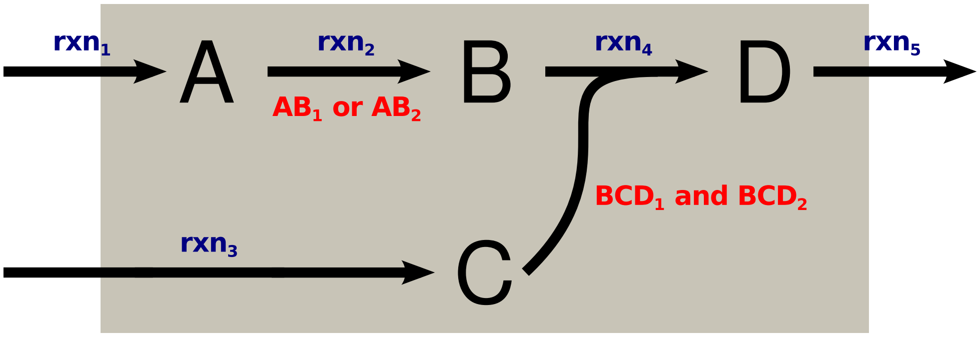

Figure 1: Simple metabolic network. Reaction rxn_2 is catalyzed by either isozyme AB_1 or AB_2. Reaction rxn_4 required both subunits BCD_1 and BCD_2 to carry flux.

```
         ub: [5x1 double]
          b: [4x1 double]
       rxns: 5x1 cell
       mets: 4x1 cell
      genes: 4x1 cell
    grRules: 5x1 cell
```

Converting this model to a TIGER model requires the following steps:

1. Adding the reaction stoichiometry to the A matrix.
2. Creating indicator variables for each reaction. These variables are true if and only if the gene-protein-reaction (GPR) rule for a reaction is true.
3. Adding constraints such that if a reaction's indicator variable is zero, no flux can be carried by the reaction.

We can convert the GPR expressions in the `grRules` field to reaction indicators and add them to a TIGER model with `add_rule`. The indicators could then be bound to the fluxes with TIGER's `bind_var` function. However, an easier approach is to use the `cobra_to_tiger` function; this function performs all of the above steps and returns a TIGER model structure.

```
>> tiger = cobra_to_tiger(cobra)
tiger =
           S: [4x5 double]
          lb: [11x1 double]
          ub: [11x1 double]
           b: [12x1 double]
        rxns: 5x1 cell
        mets: 4x1 cell
```

```
       genes: 4x1 cell
     grRules: 5x1 cell
       param: [1x1 struct]
    varnames: 11x1 cell
    rownames: 12x1 cell
           A: [12x11 double]
         obj: [11x1 double]
      ctypes: [12x1 char]
    vartypes: [11x1 char]
         gpr: 5x1 cell
         ind: [12x1 double]
    indtypes: [12x1 char]
>> tiger.varnames
ans =
    'rxn1'
    'rxn2'
    'rxn3'
    'rxn4'
    'rxn5'
    'AB1'
    'AB2'
    'BCD1'
    'BCD2'
    'RXN__rxn2'
    'RXN__rxn4'
```

The resulting TIGER model has variables for each reaction (`rxn1` - `rxn5`), the four genes from the GPR (`AB1`, `AB2`, `BCD1`, and `BCD2`), and indicator variables for the two reactions that have GPR associations (`RXN__rxn2` and `RXN__rxn4`).

The `show_tiger` function prints the constraints in a TIGER model in a readable form.

```
>> show_tiger(tiger)


----- Objective -----
z = rxn5

----- Constraints -----
A:  rxn1 - rxn2 = 0
B:  rxn2 - rxn4 = 0
C:  rxn3 - rxn4 = 0
D:  rxn4 - rxn5 = 0
ROW5:  -AB1 - AB2 + 3*RXN__rxn2 >= 0
ROW6:  -AB1 - AB2 + 3*RXN__rxn2 <= 2
ROW7:  2*BCD1 + 2*BCD2 - 4*RXN__rxn4 <= 3
ROW8:  2*BCD1 + 2*BCD2 - 4*RXN__rxn4 >= -1
ROW9:  rxn2 - 10*RXN__rxn2 <= 0
ROW10:  rxn4 - 10*RXN__rxn4 <= 0
```

```
ROW11:  rxn2 + 10*RXN__rxn2 >= 0
ROW12:  rxn4 + 10*RXN__rxn4 >= 0
```

Rows `A`, `B`, `C`, and `D` are the mass balance constraints from the original COBRA model. Rows 5-8 represent the logic for the GPR associations. The rows labeled `BIND` are the binding constraints that couple the reaction indicators to the reaction fluxes.

TIGER provides the `fba` function to run Flux Balance Analysis on a model structure. We will use Flux Balance Analysis to optimize the flux through the objective reaction `rxn5`.

```
>> sol = fba(tiger)
sol =
         x: [11x1 double]
       val: 10
    output: [1x1 struct]
      flag: 2
>> show_sol(tiger,sol)
      rxn1:  +10.000000
      rxn2:  +10.000000
      rxn3:  +10.000000
      rxn4:  +10.000000
      rxn5:  +10.000000
       AB1:  1
      BCD1:  1
      BCD2:  1
 RXN__rxn2:  1
 RXN__rxn4:  1
```

The results show that the maximum flux through `rxn_5` is 10 units.

Conditionals

Some biological constraints require more than simple Boolean logic to be expressed mathematically. TIGER allows conditional expressions as part of rules to increase their expressiveness. Consider again the example metabolic system in Figure 1. Imagine that expression of the enzyme `BCD1` was repressed by large amounts of metabolite `A` entering the system, i.e., if the flux of `rxn1` is too high, expression of `BCD1` turns off. We can include this constraint by adding it to our TIGER model as follows:

```
>> new_tiger = add_rule(tiger,'rxn1 > 7.5 => not BCD1');
>> fba(new_tiger)
ans =
         x: [14x1 double]
       val: 7.5000
```

```
    output: [1x1 struct]
      flag: 2
```

Because BCD1 is a required subunit of an essential enzyme, the optimal FBA solution only contains 7.5 units of flux through rxn5. Any more flux would increase the flux through rxn1 above the threshold and trigger BCD1 repression.

In general, conditional expressions can be used in rules anywhere a variable would be allowed, e.g. `(x > 1) or (y and (z = 0.3)) => not (w < 2)` is a valid rule. In fact, the rule `a => b` is equivalent to the rule `(a = 1) => (b = 1)`, although the first form is preferred and is more efficient.

Advanced example: a minimal genome

We will now use TIGER to calculate the minimal number of genes necessary for a functional metabolic model. This is accomplished by minimizing the number of genes that must be turned "on" while allowing a minimum level of objective flux through the network. We start by using the add_growth_constraint function to require that at least 30% of the maximum flux is always produced:

```
>> min_growth = add_growth_constraint(tiger,0.3);
```

Next we change the objective function, starting by zeroing all previous entries in min_growth.obj. Then we use the set_fieldval command to set the objective coefficient for each gene's variable to one.

```
>> min_growth.obj(:) = 0;
>> min_growth = set_fieldval(min_growth,'obj',min_growth.genes,1);
```

All that is left is to solve the model (by minimization) and display the results.

```
>> sol = solve_tiger(min_growth,'min')
sol =
         x: [11x1 double]
       val: 3
    output: [1x1 struct]
      flag: 2
>> show_sol(min_growth,sol)
      rxn1:  +3.000000
      rxn2:  +3.000000
      rxn3:  +3.000000
      rxn4:  +3.000000
      rxn5:  +3.000000
       AB1:  1
      BCD1:  1
```

```
      BCD2:  1
 RXN__rxn2:  1
 RXN__rxn4:  1
```

The results show that at least three genes are needed for a functioning model (`sol.val = 3`), and that the genes `AB1`, `BCD1`, and `BCD2` are sufficent for minimal growth.
