# Supplementary material for: TIGER: Toolbox for integrating genome-scale metabolic models, expression data, and transcriptional regulatory networks
Source: BMC Syst Biol. 2011 Sep 23;5:147. doi: 10.1186/1752-0509-5-147 (PMC3224351; doi:10.1186/1752-0509-5-147)
Supplement: Additional file 2 — TIGER source code. Source code, documentation, and tutorials are also available online at http://bme.virginia.edu/csbl/downloads/ or http://csbl.bitbucket.org/tiger. [file 1752-0509-5-147-S2.GZ › tiger/doc/m2html/index.html]

Matlab Index


# Matlab Index

## Matlab Functions by Category

**Model Creation and Modification**

- create\_empty\_tiger
- assert\_tiger
- parse\_string
- add\_rule
- set\_var
- set\_fieldval
- add\_row
- add\_column
- add\_diff
- bind\_var
- convert\_ids

**COBRA Model Conversion**

- cobra\_to\_tiger
- convert\_gpr
- bind\_mets

**Flux Balance Analysis**

- fba
- single\_gene\_ko
- add\_growth\_constraint

**Model Solution**

- solve\_tiger
- find\_infeasible\_rules

**Model Verification**

- show\_tiger
- show\_sol
- check\_tiger
- is\_valid\_rule

**Data Integration**

- made

## Matlab Directories

- tiger
- tiger/@cmpi
- tiger/cobra
- tiger/elf
- tiger/parsing
- tiger/test
- tiger/test/unit
- tiger/test/unit/tests
- tiger/test/yeast
- tiger/test/yeast/old
- tiger/tie
- tiger/util

## Matlab Files found in these Directories

|  |  |  |  |
| --- | --- | --- | --- |
| about\_tiger | count | make\_dnf | show\_mip |
| add\_column | create\_empty\_tiger | make\_expr | show\_padded |
| add\_diff | create\_table | make\_milp | show\_sol |
| add\_growth\_constraint | create\_yeast\_trn\_model | make\_rxnGeneMat | show\_tiger |
| add\_obj\_constraint | dare | make\_tiger\_doc | showif |
| add\_row | decompose\_gpr | map | single\_gene\_ko |
| add\_rule | diffadj | map\_genes\_to\_rxns | solve\_mip |
| apply\_aliases | eva | mapcols | solve\_multiple\_mips |
| argf | expand\_to | maprows | solve\_tiger |
| argmax | expr | max\_abs | splitstr |
| argmin | extract\_cobra | mea | stack |
| array2names | fba | minimal\_genome | statusbar |
| assert\_cell | fill\_to | minimal\_genome\_study | strbuffer |
| assert\_tiger | find\_associated\_rules | miqp\_type | struct2list |
| average\_by\_subsystem | find\_conditions | moma | test\_\_add\_diff |
| bind\_mets | find\_exchange\_rxns | near | test\_\_create\_table |
| bind\_var | find\_infeasible\_rules | open\_bounds | test\_\_diffadj |
| cellfilter | find\_like | parse | test\_\_fba |
| celliter | find\_optimal\_states | parse\_string | test\_\_find\_optimal\_states |
| cellzip | flatten | printbuffer | test\_\_gimme |
| cellzipn | fva | remove\_column | test\_\_imat |
| check\_field | gal\_test | remove\_null\_rules | test\_\_indicators |
| check\_mip | get\_cplex\_flag | remove\_rev\_cons | test\_\_made |
| check\_tiger | gimme | remove\_row | test\_\_miqp |
| check\_transition\_matrix | hash | remove\_rule | test\_\_multilevel |
| close\_bounds | iif | restore\_rev\_cons | test\_\_remove\_rule |
| cmpi | imat | run\_new\_rules | test\_\_solve\_multiple\_mips |
| cobra\_model | infeas\_study | run\_trn\_comparison | test\_\_tile\_mip |
| cobra\_to\_elf | init\_test | scale\_bounds | test\_tiger |
| cobra\_to\_tiger | int2bin | scanfile | textframe |
| convert\_gpr | is\_valid\_expr | set\_cplex\_opts | tile\_mip |
| convert\_grRules | is\_valid\_rule | set\_fieldval | token |
| convert\_ids | lex | set\_gurobi\_opts | tutorial\_model |
| convert\_indicators | load\_rules | set\_var | update\_rule |
| convert\_miqp | made | show\_exchange\_rxns | verify\_sol |
| convert\_rules | make\_c\_matrix | show\_made\_results |  |

---

Generated on Thu 11-Aug-2011 15:06:19 by **m2html** © 2005
