# Supplementary material for: TIGER: Toolbox for integrating genome-scale metabolic models, expression data, and transcriptional regulatory networks
Source: BMC Syst Biol. 2011 Sep 23;5:147. doi: 10.1186/1752-0509-5-147 (PMC3224351; doi:10.1186/1752-0509-5-147)
Supplement: Additional file 2 — TIGER source code. Source code, documentation, and tutorials are also available online at http://bme.virginia.edu/csbl/downloads/ or http://csbl.bitbucket.org/tiger. [file 1752-0509-5-147-S2.GZ › tiger/doc/m2html/tiger/add_diff.html]

Description of add\_diff


Home > tiger > add\_diff.m

# add\_diff

## PURPOSE

**Add difference variables toa TIGER model**

## SYNOPSIS

**function [tiger,diff\_vars] = add\_diff(tiger,var1,var2)**

## DESCRIPTION

```
 ADD_DIFF  Add difference variables toa  TIGER model

   [TIGER,DIFF_VARS] = ADD_DIFF(TIGER,VAR1,VAR2)

   Add a difference variable DIFF_VARS = |VAR1 - VAR2|.  If VAR1 and VAR2
   are binary, DIFF_VARS = VAR1 xor VAR2.  Otherwise, a multilevel OR is
   used.

   VAR1 and VAR2 are variable ids.  DIFF_VARS returns a cell of names for
   the difference variables created.  The format is 'diff__x_y' for
   variables 'x' and 'y'.
```

## CROSS-REFERENCE INFORMATION

This function calls:

- add\_column Add a column to a TIGER model structure
- add\_row Add a row to a TIGER model structure
- add\_rule Add rules to a TIGER model
- convert\_ids Create name, indices, and logical indices from an array
- cellzip Zip two cell arrays by a function
- count Count the number of nonzero elements in a vector
- map Generate a new list by applying a function

This function is called by:

- test\_\_add\_diff
- diffadj Formulate and solve the differential adjustment problem
- imat Integrative Metabolic Analysis Tool

## SOURCE CODE

```
0001 function [tiger,diff_vars] = add_diff(tiger,var1,var2)
0002 % ADD_DIFF  Add difference variables toa  TIGER model
0003 %
0004 %   [TIGER,DIFF_VARS] = ADD_DIFF(TIGER,VAR1,VAR2)
0005 %
0006 %   Add a difference variable DIFF_VARS = |VAR1 - VAR2|.  If VAR1 and VAR2
0007 %   are binary, DIFF_VARS = VAR1 xor VAR2.  Otherwise, a multilevel OR is
0008 %   used.
0009 %
0010 %   VAR1 and VAR2 are variable ids.  DIFF_VARS returns a cell of names for
0011 %   the difference variables created.  The format is 'diff__x_y' for
0012 %   variables 'x' and 'y'.
0013 
0014 assert(length(var1) == length(var2), ...
0015        'var1 and var2 must have same length');
0016 
0017 [names1,idx1] = convert_ids(tiger.varnames,var1);
0018 [names2,idx2] = convert_ids(tiger.varnames,var2);
0019 
0020 binary = tiger.vartypes(idx1) == 'b' & tiger.vartypes(idx2) == 'b';
0021 multi = ~binary;
0022 
0023 make_name = @(x,y) ['diff__' x '_' y];
0024 diff_vars = cellzip(make_name,names1,names2);
0025 
0026 if count(binary) > 0
0027     make_binary_rule = @(x,y) sprintf('(%s | %s) & ~(%s & %s) <=> %s', ...
0028                                       x,y,x,y,make_name(x,y));
0029 
0030     % add the binary rules as 'x XOR y <=> d'
0031     tiger = add_rule(tiger,cellzip(make_binary_rule,names1(binary), ...
0032                                                     names2(binary)));
0033 end
0034 
0035 N = count(multi);
0036 if N > 0
0037     idx1 = idx1(multi);
0038     idx2 = idx2(multi);
0039     fp = map(@(x) ['fp__' x],diff_vars(multi));
0040     fn = map(@(x) ['fm__' x],diff_vars(multi));
0041     
0042     fp_lb = tiger.lb(idx1) - tiger.ub(idx2);
0043     fp_ub = tiger.ub(idx1) - tiger.lb(idx2);
0044     fn_lb = tiger.lb(idx2) - tiger.ub(idx1);
0045     fn_ub = tiger.ub(idx2) - tiger.lb(idx1);
0046     
0047     d_lb = zeros(N,1);
0048     d_ub = max(tiger.ub(idx1),tiger.ub(idx2)) ...
0049             - min(tiger.lb(idx1),tiger.lb(idx2));
0050     
0051     [m,n] = size(tiger.A);
0052     tiger = add_column(tiger,[fp fn diff_vars(multi)],'c', ...
0053                        [fp_lb; fn_lb; d_lb],[fp_ub; fn_ub; d_ub]);
0054     
0055 
0056     tiger = add_row(tiger,[],repmat('=',2*N,1));
0057     for i = 1 : N
0058         % fp = x - y
0059         tiger.A(  m+i,[idx1(i) idx2(i) n+i]  ) = [ 1 -1 -1];
0060         % fn = y - x
0061         tiger.A(m+N+i,[idx1(i) idx2(i) n+N+i]) = [-1  1 -1];
0062     end
0063     or_rules = cellzip(@(x,y) [x ' OR ' y],fp,fn);
0064     rules = cellzip(@(x,y) [x ' <=> ' y],or_rules,diff_vars(multi));
0065     % fp OR fn <=> d
0066     tiger = add_rule(tiger,rules);
0067 end
0068 
0069 
0070     
0071
```

---

Generated on Thu 11-Aug-2011 15:06:22 by **m2html** © 2005
