# Supplementary material for: TIGER: Toolbox for integrating genome-scale metabolic models, expression data, and transcriptional regulatory networks
Source: BMC Syst Biol. 2011 Sep 23;5:147. doi: 10.1186/1752-0509-5-147 (PMC3224351; doi:10.1186/1752-0509-5-147)
Supplement: Additional file 2 — TIGER source code. Source code, documentation, and tutorials are also available online at http://bme.virginia.edu/csbl/downloads/ or http://csbl.bitbucket.org/tiger. [file 1752-0509-5-147-S2.GZ › tiger/doc/m2html/tiger/minimal_genome.html]

Description of minimal\_genome


Home > tiger > minimal\_genome.m

# minimal\_genome

## PURPOSE

**Calculate a minimal genome**

## SYNOPSIS

**function [genes,sol] = minimal\_genome(tiger,varargin)**

## DESCRIPTION

```
 MINIMAL_GENOME  Calculate a minimal genome

   [GENES,SOL] = MINIMAL_GENOME(TIGER,...params...)

   Computes GENES, a list of the minimum number of genes (in TIGER.genes)
   that are necessary for a functioning model.  The "params" are passed
   to ADD_GROWTH_CONSTRAINT to define the conditions for functionality.

   SOL is the solution structure from CMPI.
```

## CROSS-REFERENCE INFORMATION

This function calls:

- add\_growth\_constraint Add minimum growth constraint to a model.
- convert\_ids Create name, indices, and logical indices from an array
- fba Run Flux Balance Analysis on a TIGER model.
- set\_fieldval Set values in a TIGER structure field

This function is called by:


## SOURCE CODE

```
0001 function [genes,sol] = minimal_genome(tiger,varargin)
0002 % MINIMAL_GENOME  Calculate a minimal genome
0003 %
0004 %   [GENES,SOL] = MINIMAL_GENOME(TIGER,...params...)
0005 %
0006 %   Computes GENES, a list of the minimum number of genes (in TIGER.genes)
0007 %   that are necessary for a functioning model.  The "params" are passed
0008 %   to ADD_GROWTH_CONSTRAINT to define the conditions for functionality.
0009 %
0010 %   SOL is the solution structure from CMPI.
0011 
0012 tiger = add_growth_constraint(tiger,varargin{:});
0013 
0014 tiger.obj(:) = 0;
0015 tiger = set_fieldval(tiger,'obj',tiger.genes,-1);
0016 sol = fba(tiger);
0017 idx = convert_ids(tiger.varnames,tiger.genes,'index');
0018 if ~isempty(sol.x)
0019     genes = tiger.genes(logical(round(sol.x(idx))));
0020 else
0021     genes = [];
0022 end
0023
```

---

Generated on Thu 11-Aug-2011 15:06:22 by **m2html** © 2005
