# Supplementary material for: TIGER: Toolbox for integrating genome-scale metabolic models, expression data, and transcriptional regulatory networks
Source: BMC Syst Biol. 2011 Sep 23;5:147. doi: 10.1186/1752-0509-5-147 (PMC3224351; doi:10.1186/1752-0509-5-147)
Supplement: Additional file 2 — TIGER source code. Source code, documentation, and tutorials are also available online at http://bme.virginia.edu/csbl/downloads/ or http://csbl.bitbucket.org/tiger. [file 1752-0509-5-147-S2.GZ › tiger/doc/m2html/tiger/bind_mets.html]

Description of bind\_mets


Home > tiger > bind\_mets.m

# bind\_mets

## PURPOSE

**Bind metabolites to exchange reactions.**

## SYNOPSIS

**function [tiger] = bind\_mets(tiger)**

## DESCRIPTION

```
 BIND_METS  Bind metabolites to exchange reactions.

   [TIGER] = BIND_METS(TIGER)

   Searches for variable names that match metabolite names.  If found,
   adds constraints to the exchange reactions for the metabolite such 
   that if the metabolite is taken up, the variable must be 'on', and
   if no metabolite is taken up, the variable must be 'off'.
```

## CROSS-REFERENCE INFORMATION

This function calls:

- bind\_var Bind variables to a indicator variable

This function is called by:

- convert\_rules

## SOURCE CODE

```
0001 function [tiger] = bind_mets(tiger)
0002 % BIND_METS  Bind metabolites to exchange reactions.
0003 %
0004 %   [TIGER] = BIND_METS(TIGER)
0005 %
0006 %   Searches for variable names that match metabolite names.  If found,
0007 %   adds constraints to the exchange reactions for the metabolite such
0008 %   that if the metabolite is taken up, the variable must be 'on', and
0009 %   if no metabolite is taken up, the variable must be 'off'.
0010 
0011 m = size(tiger.S,1);
0012 to_bind = ismember(tiger.rownames(1:m),tiger.varnames);
0013 met_idxs = find(to_bind);
0014 met_inds = tiger.rownames(met_idxs);
0015 
0016 % find the excange reaction
0017 ex_idxs = zeros(size(met_idxs));
0018 colsums = sum(tiger.S ~= 0,1);
0019 for i = 1 : length(met_idxs)
0020     ex_idxs(i) = find(tiger.S(met_idxs(i),:) ~= 0 & colsums == 1);
0021 end
0022 
0023 tiger = bind_var(tiger,ex_idxs,met_inds,'iff',true);
0024
```

---

Generated on Thu 11-Aug-2011 15:06:22 by **m2html** © 2005
