# Supplementary material for: TIGER: Toolbox for integrating genome-scale metabolic models, expression data, and transcriptional regulatory networks
Source: BMC Syst Biol. 2011 Sep 23;5:147. doi: 10.1186/1752-0509-5-147 (PMC3224351; doi:10.1186/1752-0509-5-147)
Supplement: Additional file 2 — TIGER source code. Source code, documentation, and tutorials are also available online at http://bme.virginia.edu/csbl/downloads/ or http://csbl.bitbucket.org/tiger. [file 1752-0509-5-147-S2.GZ › tiger/doc/m2html/tiger/find_associated_rules.html]

Description of find\_associated\_rules


Home > tiger > find\_associated\_rules.m

# find\_associated\_rules

## PURPOSE

**Find rules associated with an atom**

## SYNOPSIS

**function [assoc] = find\_associated\_rules(exprs,start,max\_iter)**

## DESCRIPTION

```
 FIND_ASSOCIATED_RULES  Find rules associated with an atom

   [ASSOC] = FIND_ASSOCIATED_RULES(EXPRS,START,MAX_ITER)

   Retruns ASSOC, an array of indices for expressions EXPRS that may
   depend on the atoms in START.

   The function begins by finding all EXPRS that contain atoms in START.
   Any other atom that appears in one of these expressions is appended to
   START, and the process is continued for a maximum of MAX_ITER
   iterations (by default, MAX_ITER is one larger than the number of
   expressions in EXPRS, allowing all associated rules to be found).  If
   MAX_ITER = 1, only expressions that contain a member of START are
   returned.
```

## CROSS-REFERENCE INFORMATION

This function calls:

- assert\_cell Assert that variable is a cell array.
- count Count the number of nonzero elements in a vector
- map Generate a new list by applying a function

This function is called by:


## SOURCE CODE

```
0001 function [assoc] = find_associated_rules(exprs,start,max_iter)
0002 % FIND_ASSOCIATED_RULES  Find rules associated with an atom
0003 %
0004 %   [ASSOC] = FIND_ASSOCIATED_RULES(EXPRS,START,MAX_ITER)
0005 %
0006 %   Retruns ASSOC, an array of indices for expressions EXPRS that may
0007 %   depend on the atoms in START.
0008 %
0009 %   The function begins by finding all EXPRS that contain atoms in START.
0010 %   Any other atom that appears in one of these expressions is appended to
0011 %   START, and the process is continued for a maximum of MAX_ITER
0012 %   iterations (by default, MAX_ITER is one larger than the number of
0013 %   expressions in EXPRS, allowing all associated rules to be found).  If
0014 %   MAX_ITER = 1, only expressions that contain a member of START are
0015 %   returned.
0016 
0017 assert(~isempty(start),'a list of starting atoms must be given');
0018 
0019 if nargin < 3
0020     max_iter = length(exprs) + 1;
0021 end
0022 
0023 rules = exprs;
0024 atoms = map(@(x) x.atoms,rules);
0025 
0026 assoc = false(size(rules));
0027 
0028 prev_count = -1;
0029 current_imp = assert_cell(start);
0030 
0031 curr_iter = 0;
0032 while prev_count < count(assoc) && curr_iter < max_iter
0033     curr_iter = curr_iter + 1;
0034     
0035     prev_count = count(assoc);
0036     f = @(x) any(ismember(current_imp,x));
0037     to_add = cellfun(f,atoms);
0038     assoc = assoc | to_add;
0039     new_atoms = atoms(to_add);
0040     current_imp = [current_imp{:},new_atoms{:}];
0041 end
0042 
0043 assoc = find(assoc);
```

---

Generated on Thu 11-Aug-2011 15:06:22 by **m2html** © 2005
