# Supplementary material for: TIGER: Toolbox for integrating genome-scale metabolic models, expression data, and transcriptional regulatory networks
Source: BMC Syst Biol. 2011 Sep 23;5:147. doi: 10.1186/1752-0509-5-147 (PMC3224351; doi:10.1186/1752-0509-5-147)
Supplement: Additional file 2 — TIGER source code. Source code, documentation, and tutorials are also available online at http://bme.virginia.edu/csbl/downloads/ or http://csbl.bitbucket.org/tiger. [file 1752-0509-5-147-S2.GZ › tiger/doc/m2html/tiger/util/printbuffer.html]

Description of printbuffer


Home > tiger > util > printbuffer.m

# printbuffer

## PURPOSE

## SYNOPSIS

**This is a script file.**

## DESCRIPTION

## CROSS-REFERENCE INFORMATION

This function calls:

- printbuffer

This function is called by:

- show\_mip Show equations for a MIP structure
- printbuffer

## SUBFUNCTIONS

- function printf(obj,fmt,varargin)
- function start\_wrap(obj)
- function stop\_wrap(obj)
- function newline(obj,n)
- function output(obj,str)
- function show(obj,str)

## SOURCE CODE

```
0001 classdef printbuffer < handle
0002     
0003 properties
0004     wrap = false;
0005     width = 80;
0006     curr = 0;
0007     indent = '   '
0008 end
0009 
0010 methods
0011     function printf(obj,fmt,varargin)
0012         str = sprintf(fmt,varargin{:});
0013         l = length(str);
0014         if obj.wrap
0015             if l + obj.curr > obj.width
0016                 obj.newline;
0017                 obj.output(obj.indent);
0018                 obj.curr = l + length(obj.indent);
0019             else
0020                 obj.curr = obj.curr + l;
0021             end
0022         end
0023         obj.output(str);
0024     end
0025     
0026     function start_wrap(obj)
0027         obj.wrap = true;
0028         obj.curr = 0;
0029     end
0030     
0031     function stop_wrap(obj)
0032         obj.wrap = false;
0033     end
0034     
0035     function newline(obj,n)
0036         if nargin < 2
0037             n = 1;
0038         end
0039         obj.output(repmat('\n',1,n));
0040     end
0041     
0042     function output(obj,str)
0043         fprintf(str);
0044     end
0045     
0046     function show(obj,str)
0047         obj.output(str);
0048         obj.newline();
0049     end
0050 end
0051 
0052 end
0053
```

---

Generated on Thu 11-Aug-2011 15:06:22 by **m2html** © 2005
