# Supplementary material for: TIGER: Toolbox for integrating genome-scale metabolic models, expression data, and transcriptional regulatory networks
Source: BMC Syst Biol. 2011 Sep 23;5:147. doi: 10.1186/1752-0509-5-147 (PMC3224351; doi:10.1186/1752-0509-5-147)
Supplement: Additional file 2 — TIGER source code. Source code, documentation, and tutorials are also available online at http://bme.virginia.edu/csbl/downloads/ or http://csbl.bitbucket.org/tiger. [file 1752-0509-5-147-S2.GZ › tiger/doc/m2html/tiger/util/index.html]

Index for Directory tiger/util


|  |  |
| --- | --- |
| Master index | Index for tiger/util |

# Index for tiger/util

## Matlab files in this directory:

|  |  |
| --- | --- |
| argf | Return the index vector for a function |
| argmax | Return the arg-maximum of a function |
| argmin | Return the arg-minimum of a function |
| array2names | Create a cell of names from an array of numbers |
| assert\_cell | Assert that variable is a cell array. |
| cellfilter | Return a subset of a cell array |
| celliter | Iterate over elements in a cell |
| cellzip | Zip two cell arrays by a function |
| cellzipn | Zip an unlimited number of cell arrays by a function |
| count | Count the number of nonzero elements in a vector |
| create\_table | Format and display tabular data |
| expand\_to | Expand a vector to length N |
| fill\_to | Fill a short to empty vector |
| find\_like | Find matches in a cell of strings |
| flatten | Flatten a cell of cells into a single cell. |
| hash |  |
| iif | Inline operator form of the IF structure |
| int2bin | Convert an integer to an array of binary values |
| make\_tiger\_doc |  |
| map | Generate a new list by applying a function |
| mapcols | Apply a function to columns in a matrix |
| maprows | Apply a function to rows in a matrix |
| max\_abs | Maximum absolute value in a set of vectors |
| near | Test if two values are close to each other |
| printbuffer |  |
| scanfile | Apply TEXTSCAN to a filename |
| show\_padded | Show padded strings |
| showif | Conditionally display to the command window |
| splitstr | Perl-style string splitting |
| stack |  |
| statusbar |  |
| strbuffer |  |
| struct2list | Convert a structure to a parameter list |
| textframe |  |

---

Generated on Thu 11-Aug-2011 15:06:20 by **m2html** © 2005
