# Supplementary material for: TIGER: Toolbox for integrating genome-scale metabolic models, expression data, and transcriptional regulatory networks
Source: BMC Syst Biol. 2011 Sep 23;5:147. doi: 10.1186/1752-0509-5-147 (PMC3224351; doi:10.1186/1752-0509-5-147)
Supplement: Additional file 2 — TIGER source code. Source code, documentation, and tutorials are also available online at http://bme.virginia.edu/csbl/downloads/ or http://csbl.bitbucket.org/tiger. [file 1752-0509-5-147-S2.GZ › tiger/doc/m2html/tiger/util/int2bin.html]

Description of int2bin


Home > tiger > util > int2bin.m

# int2bin

## PURPOSE

**Convert an integer to an array of binary values**

## SYNOPSIS

**function [bin] = int2bin(num,n\_bits)**

## DESCRIPTION

```
 INT2BIN  Convert an integer to an array of binary values

   [BIN] = INT2BIN(NUM)
   [BIN] = INT2BIN(NUM,N_BITS)

   Returns a vector BIN that is a binary encoding of the integer NUM.
   If given, BIN has length N_BITS; otherwise, BIN is the length of the
   fewest number of bits necessary to encode NUM.

   Examples:
   >> int2bin(10)
   ans = 
       1  0  1  0
   >> int2bin(10,6)
   ans = 
       0  0  1  0  1  0
```

## CROSS-REFERENCE INFORMATION

This function calls:


This function is called by:

- make\_c\_matrix Make reaction/gene correlation (C) matrix
- find\_optimal\_states Find optimal binary states

## SOURCE CODE

```
0001 function [bin] = int2bin(num,n_bits)
0002 % INT2BIN  Convert an integer to an array of binary values
0003 %
0004 %   [BIN] = INT2BIN(NUM)
0005 %   [BIN] = INT2BIN(NUM,N_BITS)
0006 %
0007 %   Returns a vector BIN that is a binary encoding of the integer NUM.
0008 %   If given, BIN has length N_BITS; otherwise, BIN is the length of the
0009 %   fewest number of bits necessary to encode NUM.
0010 %
0011 %   Examples:
0012 %   >> int2bin(10)
0013 %   ans =
0014 %       1  0  1  0
0015 %   >> int2bin(10,6)
0016 %   ans =
0017 %       0  0  1  0  1  0
0018 
0019 N = floor(log2(num)) + 1;
0020 if nargin == 2
0021     N = max(N,n_bits);
0022 end
0023 
0024 bin = zeros(1,N);
0025 
0026 for i = N : -1 : 1
0027     k = 2^(i-1);
0028     if num >= k
0029         num = num - k;
0030         bin(N+1-i) = 1;
0031     end
0032 end
```

---

Generated on Thu 11-Aug-2011 15:06:22 by **m2html** © 2005
