# Supplementary material for: TIGER: Toolbox for integrating genome-scale metabolic models, expression data, and transcriptional regulatory networks
Source: BMC Syst Biol. 2011 Sep 23;5:147. doi: 10.1186/1752-0509-5-147 (PMC3224351; doi:10.1186/1752-0509-5-147)
Supplement: Additional file 2 — TIGER source code. Source code, documentation, and tutorials are also available online at http://bme.virginia.edu/csbl/downloads/ or http://csbl.bitbucket.org/tiger. [file 1752-0509-5-147-S2.GZ › tiger/doc/m2html/tiger/util/show_padded.html]

Description of show\_padded


Home > tiger > util > show\_padded.m

# show\_padded

## PURPOSE

**Show padded strings**

## SYNOPSIS

**function [str] = show\_padded(str1,str2,width,pad)**

## DESCRIPTION

```
 SHOW_PADDED  Show padded strings

   SHOW_PADDED(STR1,STR2,WIDTH,PAD)
   [STR] = SHOW_PADDED(...)

   Prints STR1 and STR2, separated by PAD, repeated enough times to give
   an overall length of WIDTH.

   If called with a return argument, the padded string is returned, not
   printed.
```

## CROSS-REFERENCE INFORMATION

This function calls:


This function is called by:

- test\_tiger Run unit tests on the TIGER package

## SOURCE CODE

```
0001 function [str] = show_padded(str1,str2,width,pad)
0002 % SHOW_PADDED  Show padded strings
0003 %
0004 %   SHOW_PADDED(STR1,STR2,WIDTH,PAD)
0005 %   [STR] = SHOW_PADDED(...)
0006 %
0007 %   Prints STR1 and STR2, separated by PAD, repeated enough times to give
0008 %   an overall length of WIDTH.
0009 %
0010 %   If called with a return argument, the padded string is returned, not
0011 %   printed.
0012 
0013 if nargin < 4 || isempty(pad)
0014     pad = '.';
0015 end
0016 if nargin < 3 || isempty(width)
0017     width = 70;
0018 end
0019 
0020 assert(nargin >= 2, 'SHOW_PADDED requires two arguments');
0021 
0022 pad_width = width - length(str1) - length(str2);
0023 padding = repmat(pad,1,pad_width);
0024 
0025 str = sprintf('%s%s%s',str1,padding,str2);
0026 if nargout == 0
0027     fprintf('%s\n',str);
0028 end
0029
```

---

Generated on Thu 11-Aug-2011 15:06:22 by **m2html** © 2005
