# Supplementary material for: TIGER: Toolbox for integrating genome-scale metabolic models, expression data, and transcriptional regulatory networks
Source: BMC Syst Biol. 2011 Sep 23;5:147. doi: 10.1186/1752-0509-5-147 (PMC3224351; doi:10.1186/1752-0509-5-147)
Supplement: Additional file 2 — TIGER source code. Source code, documentation, and tutorials are also available online at http://bme.virginia.edu/csbl/downloads/ or http://csbl.bitbucket.org/tiger. [file 1752-0509-5-147-S2.GZ › tiger/doc/m2html/tiger/util/textframe.html]

Description of textframe


Home > tiger > util > textframe.m

# textframe

## PURPOSE

## SYNOPSIS

**This is a script file.**

## DESCRIPTION

## CROSS-REFERENCE INFORMATION

This function calls:

- assert\_cell Assert that variable is a cell array.
- textframe

This function is called by:

- expr
- create\_table Format and display tabular data
- textframe

## SUBFUNCTIONS

- function [obj] = textframe(lines)
- function display(obj)
- function [widths] = get.widths(obj)
- function [width] = get.width(obj)
- function [height] = get.height(obj)
- function [block] = make\_block(obj,varargin)
- function [newtf] = hcat2(frame1,frame2,varargin)
- function [newtf] = hcat(varargin)
- function [newtf] = vcat(varargin)
- function [new] = copy(obj)
- function add\_line(obj,fmt,varargin)

## SOURCE CODE

```
0001 classdef textframe < handle
0002 % TEXTFRAME  Format and pad multi-line text blocks
0003 %
0004 %   TEXTFRAME formats a set of uneven-lengthed strings into blocks
0005 %   of text with vertical and horizontal alignment.  TEXTFRAMEs can
0006 %   be easily combined with other TEXTFRAMEs using the VCAT and HCAT
0007 %   methods.
0008     
0009 properties
0010     lines         % cells of text lines
0011     SPACER = ' '  % character used to pad lines during formatting
0012 end
0013 
0014 properties (Dependent)
0015     width   % width of the longest line
0016     widths  % width of each individual line
0017     height  % number of lines
0018 end
0019 
0020 methods
0021     function [obj] = textframe(lines)
0022         % TEXTFRAME  Construct a TEXTFRAME
0023         %
0024         %   [OBJ] = TEXTFRAME(LINES)
0025         %
0026         %   Create a TEXTFRAME object.  LINES is an optional cell
0027         %   array of strings.
0028         
0029         if nargin == 0
0030             lines = {};
0031         end
0032         lines = assert_cell(lines);
0033         obj.lines = lines(:);
0034     end
0035     
0036     function display(obj)
0037         % DISPLAY  Display a TEXTFRAME
0038         %
0039         %   Displays a TEXTFRAME using default height and width.
0040         
0041         display_block = repmat(' ',obj.height,obj.width);
0042         lines = obj.make_block();
0043         for i = 1 : obj.height
0044             display_block(i,:) = lines.lines{i};
0045         end
0046         disp(display_block);
0047     end
0048     
0049     % ------- dependent access methods -------
0050     function [widths] = get.widths(obj)
0051         widths = cellfun(@length,obj.lines);
0052     end
0053     
0054     function [width] = get.width(obj)
0055         width = max(obj.widths);
0056     end
0057     
0058     function [height] = get.height(obj)
0059         height = length(obj.lines);
0060     end
0061     
0062     
0063     function [block] = make_block(obj,varargin)
0064         % MAKE_BLOCK  Create a TEXTFRAME with alignment padding
0065         %
0066         %   [BLOCK] = MAKE_BLOCK(...params...)
0067         %
0068         %   Parameters
0069         %   'height'    Height (number of lines).
0070         %   'width'     Width (number of characters).
0071         %   'halign'    Horizontal alignment.  Lines should be aligned
0072         %               on the 'left' (default), 'right', or 'center' of
0073         %               the block.
0074         %   'valign'    Vertical alignment.  Lines should be aligned on
0075         %               the 'top' (default), 'bottom', or 'middle' of
0076         %               the block.
0077         
0078         p = inputParser;
0079         p.addParamValue('height',obj.height);
0080         p.addParamValue('width',obj.width);
0081         p.addParamValue('halign','left');
0082         p.addParamValue('valign','top');
0083         
0084         p.parse(varargin{:});
0085         width = p.Results.width;
0086         height = p.Results.height;
0087         halign = p.Results.halign;
0088         valign = p.Results.valign;
0089         
0090         if any(obj.widths > width)
0091             warning('Some lines will be clipped horizontally.');
0092         end
0093         if obj.height > height
0094             warning('Some lines will be clipped vertically.');
0095             block = obj.lines(1:height);
0096         else
0097             block = obj.lines;
0098             if obj.height < height
0099                 nulls = arrayfun(@(x) '',1:(height-obj.height), ...
0100                                  'Uniform',false)';
0101                 switch valign
0102                     case {'top','t'}
0103                         block = [block; nulls];
0104                     case {'bottom','b'}
0105                         block = [nulls; block];
0106                     case {'middle','m'}
0107                         cut = length(nulls)/2 + 0.1;
0108                         block = [nulls(1:floor(cut)); ...
0109                                  block; ...
0110                                  nulls(ceil(cut):end)];
0111                 end
0112             end
0113         end
0114         
0115         for i = 1 : height
0116             if length(block{i}) > width
0117                 block{i} = block{i}(1:width);
0118             elseif length(block{i}) < width
0119                 spacer = repmat(obj.SPACER,1,width - length(block{i}));
0120                 switch halign
0121                     case {'left','l'}
0122                         block{i} = [block{i} spacer];
0123                     case {'right','r'}
0124                         block{i} = [spacer block{i}];
0125                     case {'center','c'}
0126                         cut = length(spacer)/2 + 0.1;
0127                         block{i} = [spacer(1:floor(cut)), ...
0128                                     block{i}, ...
0129                                     spacer(ceil(cut):end)];
0130                 end
0131             end
0132         end
0133         
0134         block = textframe(block);
0135     end
0136     
0137     function [newtf] = hcat2(frame1,frame2,varargin)
0138         % HCAT2  Horizontally concatenate two TEXTFRAMES
0139         %
0140         %   [NEWTF] = HCAT2(FRAME1,FRAME2,...params...)
0141         %
0142         %   Horizontally pad and concatenate two TEXTFRAMEs and return
0143         %   the resulting textframe.  This is an auxiliary function
0144         %   called by HCAT.
0145         
0146         p = inputParser;
0147         p.addParamValue('height',max([frame1.height,frame2.height]));
0148         p.addParamValue('spacer', '');
0149         p.addParamValue('valign','top');
0150         
0151         p.parse(varargin{:});
0152         
0153         args = {'height',p.Results.height, ...
0154                 'valign',p.Results.valign};
0155         f1 = frame1.make_block(args{:});
0156         f2 = frame2.make_block(args{:});
0157         
0158         newtf = f1.copy;
0159         for i = 1 : f1.height
0160             newtf.lines{i} = [f1.lines{i} p.Results.spacer f2.lines{i}];
0161         end
0162     end
0163     
0164     function [newtf] = hcat(varargin)
0165         % HCAT  Horizontally concatenate a series of TEXTFRAMEs
0166         %
0167         %   [NEWTF] = HCAT(FRAME1,FRAME2,...,...params...)
0168         %
0169         %   Align and concatenate a series of TEXTFRAMEs.  Parameters
0170         %   are the same as for MAKE_BLOCK.
0171         
0172         % separate the textframes from the parameter list
0173         is_tf = cellfun(@(x) isa(x,'textframe'),varargin);
0174         tfs = varargin(is_tf);
0175         args = varargin(~is_tf);
0176         
0177         newtf = tfs{1}.copy;
0178         for i = 2 : length(tfs)
0179             newtf = newtf.hcat2(tfs{i},args{:});
0180         end
0181     end
0182     
0183     function [newtf] = vcat(varargin)
0184         % VCAT  Vertically concatenate a series of TEXTFRAMEs
0185         %
0186         %   [NEWTF] = VCAT(FRAME1,FRAME2,...)
0187         %
0188         %   Vertically concatenate a series of TEXTFRAME objects.
0189         %   No alignment is applied; lines are simply added together.
0190         
0191         newtf = varargin{1}.copy;
0192         for i = 2 : length(varargin)
0193             newtf.lines = [newtf.lines; varargin{i}.lines];
0194         end
0195     end
0196     
0197     function [new] = copy(obj)
0198         % COPY  Create a duplicate TEXTFRAME object.
0199         %
0200         %   COPY duplicates a TEXTFRAME object with a separate
0201         %   handle.  This avoids links from simple copies.
0202         
0203         new = textframe(obj.lines);
0204     end
0205             
0206     function add_line(obj,fmt,varargin)
0207         % ADD_LINE  Add a string to a textframe.
0208         %
0209         %   ADD_LINE(FMT,...)
0210         %
0211         %   Adds a line to the end of a TEXTFRAME.  FMT is a
0212         %   PRINTF-style format string, with optional arguments.
0213         
0214         obj.lines{end+1,1} = sprintf(fmt,varargin{:});
0215     end
0216         
0217 end
0218 
0219 end % classdef
0220 
0221             
0222
```

---

Generated on Thu 11-Aug-2011 15:06:22 by **m2html** © 2005
