# Supplementary material for: TIGER: Toolbox for integrating genome-scale metabolic models, expression data, and transcriptional regulatory networks
Source: BMC Syst Biol. 2011 Sep 23;5:147. doi: 10.1186/1752-0509-5-147 (PMC3224351; doi:10.1186/1752-0509-5-147)
Supplement: Additional file 2 — TIGER source code. Source code, documentation, and tutorials are also available online at http://bme.virginia.edu/csbl/downloads/ or http://csbl.bitbucket.org/tiger. [file 1752-0509-5-147-S2.GZ › tiger/doc/m2html/tiger/util/iif.html]

Description of iif


Home > tiger > util > iif.m

# iif

## PURPOSE

**Inline operator form of the IF structure**

## SYNOPSIS

**function [val] = iif(test,val1,val2,lazy)**

## DESCRIPTION

```
 IIF  Inline operator form of the IF structure

   [VAL] = IIF(TEST,VAL1,VAL2)

   If TEST is true, VAL = VAL1.  Otherwise, VAL = VAL2.  If no value is
   given for VAL2, the default is [].
```

## CROSS-REFERENCE INFORMATION

This function calls:


This function is called by:


## SOURCE CODE

```
0001 function [val] = iif(test,val1,val2,lazy)
0002 % IIF  Inline operator form of the IF structure
0003 %
0004 %   [VAL] = IIF(TEST,VAL1,VAL2)
0005 %
0006 %   If TEST is true, VAL = VAL1.  Otherwise, VAL = VAL2.  If no value is
0007 %   given for VAL2, the default is [].
0008 
0009 if nargin < 4
0010     lazy = false;
0011 end
0012 
0013 if nargin == 2
0014     val2 = [];
0015 end
0016 
0017 if nargin < 2
0018     error('at least two inputs required');
0019 end
0020 
0021 if test
0022     if lazy
0023         val = eval(val1);
0024     else
0025         val = val1;
0026     end
0027 else
0028     if lazy
0029         val = eval(val2);
0030     else
0031         val = val2;
0032     end
0033 end
```

---

Generated on Thu 11-Aug-2011 15:06:22 by **m2html** © 2005
