# Supplementary material for: TIGER: Toolbox for integrating genome-scale metabolic models, expression data, and transcriptional regulatory networks
Source: BMC Syst Biol. 2011 Sep 23;5:147. doi: 10.1186/1752-0509-5-147 (PMC3224351; doi:10.1186/1752-0509-5-147)
Supplement: Additional file 2 — TIGER source code. Source code, documentation, and tutorials are also available online at http://bme.virginia.edu/csbl/downloads/ or http://csbl.bitbucket.org/tiger. [file 1752-0509-5-147-S2.GZ › tiger/doc/m2html/tiger/util/cellfilter.html]

Description of cellfilter


Home > tiger > util > cellfilter.m

# cellfilter

## PURPOSE

**Return a subset of a cell array**

## SYNOPSIS

**function [filtered,locs,tf] = cellfilter(f,C)**

## DESCRIPTION

```
 CELLFILTER  Return a subset of a cell array

   [FILTERED,LOCS,TF] = CELLFILTER(F,C)

   Returns the elements of C for which F(C{i}) is true.  The locations of
   the elements are LOCS.  TF is a logical indexing array such that 
   FILTERED = C(TF) = C(LOCS).
```

## CROSS-REFERENCE INFORMATION

This function calls:


This function is called by:

- create\_yeast\_trn\_model
- find\_like Find matches in a cell of strings

## SOURCE CODE

```
0001 function [filtered,locs,tf] = cellfilter(f,C)
0002 % CELLFILTER  Return a subset of a cell array
0003 %
0004 %   [FILTERED,LOCS,TF] = CELLFILTER(F,C)
0005 %
0006 %   Returns the elements of C for which F(C{i}) is true.  The locations of
0007 %   the elements are LOCS.  TF is a logical indexing array such that
0008 %   FILTERED = C(TF) = C(LOCS).
0009 
0010 tf = cellfun(f,C);
0011 filtered = C(tf);
0012 
0013 if nargout >= 2
0014     locs = find(tf);
0015 end
0016
```

---

Generated on Thu 11-Aug-2011 15:06:22 by **m2html** © 2005
