# Supplementary material for: TIGER: Toolbox for integrating genome-scale metabolic models, expression data, and transcriptional regulatory networks
Source: BMC Syst Biol. 2011 Sep 23;5:147. doi: 10.1186/1752-0509-5-147 (PMC3224351; doi:10.1186/1752-0509-5-147)
Supplement: Additional file 2 — TIGER source code. Source code, documentation, and tutorials are also available online at http://bme.virginia.edu/csbl/downloads/ or http://csbl.bitbucket.org/tiger. [file 1752-0509-5-147-S2.GZ › tiger/doc/m2html/tiger/util/splitstr.html]

Description of splitstr


Home > tiger > util > splitstr.m

# splitstr

## PURPOSE

**Perl-style string splitting**

## SYNOPSIS

**function [parts] = splitstr(str,regex)**

## DESCRIPTION

```
 SPLITSTR  Perl-style string splitting

   [PARTS] = SPLITSTR(STR,REGEX) splits STR by the regular expression
   REGEX, returning the remaining sections as PARTS.
```

## CROSS-REFERENCE INFORMATION

This function calls:


This function is called by:


## SOURCE CODE

```
0001 function [parts] = splitstr(str,regex)
0002 % SPLITSTR  Perl-style string splitting
0003 %
0004 %   [PARTS] = SPLITSTR(STR,REGEX) splits STR by the regular expression
0005 %   REGEX, returning the remaining sections as PARTS.
0006 
0007 parts = regexp(str,regex,'split');
```

---

Generated on Thu 11-Aug-2011 15:06:22 by **m2html** © 2005
