# Supplementary material for: TIGER: Toolbox for integrating genome-scale metabolic models, expression data, and transcriptional regulatory networks
Source: BMC Syst Biol. 2011 Sep 23;5:147. doi: 10.1186/1752-0509-5-147 (PMC3224351; doi:10.1186/1752-0509-5-147)
Supplement: Additional file 2 — TIGER source code. Source code, documentation, and tutorials are also available online at http://bme.virginia.edu/csbl/downloads/ or http://csbl.bitbucket.org/tiger. [file 1752-0509-5-147-S2.GZ › tiger/doc/m2html/tiger/util/cellzip.html]

Description of cellzip


Home > tiger > util > cellzip.m

# cellzip

## PURPOSE

**Zip two cell arrays by a function**

## SYNOPSIS

**function [ziped] = cellzip(f,a,b)**

## DESCRIPTION

```
 CELLZIP  Zip two cell arrays by a function

   [ZIPED] = CELLZIP(F,A,B)

   Computes ZIPED{i} = F(A{i},B{i}) foreach i in A,B.
```

## CROSS-REFERENCE INFORMATION

This function calls:


This function is called by:

- add\_diff Add difference variables toa TIGER model
- bind\_var Bind variables to a indicator variable
- convert\_gpr Add the GPR rules as constraints to the model.
- imat Integrative Metabolic Analysis Tool

## SOURCE CODE

```
0001 function [ziped] = cellzip(f,a,b)
0002 % CELLZIP  Zip two cell arrays by a function
0003 %
0004 %   [ZIPED] = CELLZIP(F,A,B)
0005 %
0006 %   Computes ZIPED{i} = F(A{i},B{i}) foreach i in A,B.
0007 
0008 ziped = arrayfun(@(i) f(a{i},b{i}),1:length(a),'Uniform',false);
```

---

Generated on Thu 11-Aug-2011 15:06:22 by **m2html** © 2005
