# Supplementary material for: TIGER: Toolbox for integrating genome-scale metabolic models, expression data, and transcriptional regulatory networks
Source: BMC Syst Biol. 2011 Sep 23;5:147. doi: 10.1186/1752-0509-5-147 (PMC3224351; doi:10.1186/1752-0509-5-147)
Supplement: Additional file 2 — TIGER source code. Source code, documentation, and tutorials are also available online at http://bme.virginia.edu/csbl/downloads/ or http://csbl.bitbucket.org/tiger. [file 1752-0509-5-147-S2.GZ › tiger/doc/m2html/tiger/util/stack.html]

Description of stack


Home > tiger > util > stack.m

# stack

## PURPOSE

## SYNOPSIS

**This is a script file.**

## DESCRIPTION

## CROSS-REFERENCE INFORMATION

This function calls:

- stack

This function is called by:

- lex Return a list of TOKENS from a string
- stack

## SUBFUNCTIONS

- function [obj] = stack(array)
- function [item] = pop(obj)
- function push(obj,item)
- function [item] = peek(obj)
- function reverse(obj)
- function [N] = get.length(obj)
- function [N] = get.N(obj)
- function [tf] = get.is\_another(obj)
- function [tf] = get.is\_empty(obj)

## SOURCE CODE

```
0001 classdef stack < handle
0002 % STACK  First-in, last-out stacks.
0003 %
0004 %   STACK creates a polymorphic stack with first-in, last-out (FILO)
0005 %   behavior.
0006     
0007 properties (Dependent)
0008     is_another  % true is stack contains another value
0009     is_empty    % true if stack is empty
0010     length      % number of items on stack
0011 end
0012 properties (Dependent,Hidden)
0013     N   % number of items on stack
0014 end
0015 
0016 properties (SetAccess = private)
0017     values  % cell of stack values
0018 end
0019 
0020 methods
0021     function [obj] = stack(array)
0022         % STACK  Create a FILO stack.
0023         %
0024         %   [OBJ] = STACK(ARRAY)
0025         %
0026         %   Creates a new stack.  ARRAY is an optional cell of initial
0027         %   values for the stack.  (The last item in the cell is the
0028         %   first item to be removed with POP.
0029         
0030         if nargin == 0
0031             obj.values = {};
0032         elseif isa(array,'cell')
0033             obj.values = array;
0034         else
0035             N = length(array);
0036             obj.values = cell(1,N);
0037             for i = 1 : N
0038                 obj.values{i} = array(i);
0039             end
0040         end
0041     end
0042 
0043     function [item] = pop(obj)
0044         % POP  Remove the last object added to the stack.
0045         assert(obj.is_another, 'stack is empty');
0046         item = obj.values{end};
0047         obj.values = obj.values(1:end-1);
0048     end
0049 
0050     function push(obj,item)
0051         % PUSH  Add an item to the stack.
0052         obj.values{end+1} = item;
0053     end
0054 
0055     function [item] = peek(obj)
0056         % PEEK  Return the last item added to the stack,
0057         %       without removing it.
0058         assert(obj.is_another, 'stack is empty');
0059         item = obj.values{end};
0060     end
0061 
0062     function reverse(obj)
0063         % REVERSE  Reverse the order of items on the stack.
0064         obj.values = fliplr(obj.values);
0065     end
0066     
0067     % ------- dependent access methods -------
0068     function [N] = get.length(obj)
0069         N = length(obj.values);
0070     end
0071     function [N] = get.N(obj)
0072         N = obj.length;
0073     end
0074     function [tf] = get.is_another(obj)
0075         tf = obj.N > 0;
0076     end
0077     function [tf] = get.is_empty(obj)
0078         tf = ~obj.is_another;
0079     end
0080 end
0081 
0082 end % classdef
0083
```

---

Generated on Thu 11-Aug-2011 15:06:22 by **m2html** © 2005
