# Supplementary material for: TIGER: Toolbox for integrating genome-scale metabolic models, expression data, and transcriptional regulatory networks
Source: BMC Syst Biol. 2011 Sep 23;5:147. doi: 10.1186/1752-0509-5-147 (PMC3224351; doi:10.1186/1752-0509-5-147)
Supplement: Additional file 2 — TIGER source code. Source code, documentation, and tutorials are also available online at http://bme.virginia.edu/csbl/downloads/ or http://csbl.bitbucket.org/tiger. [file 1752-0509-5-147-S2.GZ › tiger/doc/m2html/tiger/util/decompose_gpr.html]

Description of decompose\_gpr


Home > tiger > util > decompose\_gpr.m

# decompose\_gpr

## PURPOSE

## SYNOPSIS

**function decompose\_gpr(cobra)**

## DESCRIPTION

## CROSS-REFERENCE INFORMATION

This function calls:

- count Count the number of nonzero elements in a vector

This function is called by:


## SOURCE CODE

```
0001 function decompose_gpr(cobra)
0002 
0003 byg = sum(cobra.rxnGeneMat,1);
0004 byr = sum(cobra.rxnGeneMat,2);
0005 
0006 [nr,ng] = size(cobra.rxnGeneMat);
0007 
0008 none = count(byr == 0);
0009 fprintf('No GPR:   %i / %i (%.1f%%)\n',none,nr,none/nr*100);
0010 
0011 one = count(byr == 1);
0012 fprintf('One ORF:  %i / %i (%.1f%%)\n',one,nr,one/nr*100);
0013 
0014 promis = count(byg > 1);
0015 fprintf('2+ rxns:  %i / %i (%.1f%%)\n',promis,ng,promis/ng*100);
0016 
0017 and_cnt = cellfun(@(x) length(regexp(x,'&')),cobra.rules);
0018  or_cnt = cellfun(@(x) length(regexp(x,'\|')),cobra.rules);
0019 
0020 fprintf('Isozymes:  %i\n',sum(or_cnt));
0021 fprintf('Subunits:  %i\n',sum(and_cnt));
```

---

Generated on Thu 04-Aug-2011 09:58:54 by **m2html** © 2005
