# Supplementary material for: TIGER: Toolbox for integrating genome-scale metabolic models, expression data, and transcriptional regulatory networks
Source: BMC Syst Biol. 2011 Sep 23;5:147. doi: 10.1186/1752-0509-5-147 (PMC3224351; doi:10.1186/1752-0509-5-147)
Supplement: Additional file 2 — TIGER source code. Source code, documentation, and tutorials are also available online at http://bme.virginia.edu/csbl/downloads/ or http://csbl.bitbucket.org/tiger. [file 1752-0509-5-147-S2.GZ › tiger/doc/m2html/tiger/util/cellzipn.html]

Description of cellzipn


Home > tiger > util > cellzipn.m

# cellzipn

## PURPOSE

**Zip an unlimited number of cell arrays by a function**

## SYNOPSIS

**function [ziped] = cellzipn(f,varargin)**

## DESCRIPTION

```
 CELLZIP  Zip an unlimited number of cell arrays by a function

   [ZIPED] = CELLZIP(F,...)

   Computes ZIPED{i} = F(...), iterating element by element for each
   input cell.
```

## CROSS-REFERENCE INFORMATION

This function calls:

- argmax Return the arg-maximum of a function
- map Generate a new list by applying a function

This function is called by:


## SOURCE CODE

```
0001 function [ziped] = cellzipn(f,varargin)
0002 % CELLZIP  Zip an unlimited number of cell arrays by a function
0003 %
0004 %   [ZIPED] = CELLZIP(F,...)
0005 %
0006 %   Computes ZIPED{i} = F(...), iterating element by element for each
0007 %   input cell.
0008 
0009 if isempty(varargin)
0010     ziped = [];
0011 else
0012     N = length(varargin{1});
0013     if argmax(size(varargin),1) == 1
0014         ziped = cell(N,1);
0015     else
0016         ziped = cell(1,N);
0017     end
0018     
0019     extract = @(c,i) map(@(x) x{i},c);
0020     for i = 1 : N
0021         ziped{i} = f(extract(varargin,i));
0022     end
0023 end
0024
```

---

Generated on Thu 11-Aug-2011 15:06:22 by **m2html** © 2005
