# Supplementary material for: TIGER: Toolbox for integrating genome-scale metabolic models, expression data, and transcriptional regulatory networks
Source: BMC Syst Biol. 2011 Sep 23;5:147. doi: 10.1186/1752-0509-5-147 (PMC3224351; doi:10.1186/1752-0509-5-147)
Supplement: Additional file 2 — TIGER source code. Source code, documentation, and tutorials are also available online at http://bme.virginia.edu/csbl/downloads/ or http://csbl.bitbucket.org/tiger. [file 1752-0509-5-147-S2.GZ › tiger/doc/m2html/tiger/util/count.html]

Description of count


Home > tiger > util > count.m

# count

## PURPOSE

**Count the number of nonzero elements in a vector**

## SYNOPSIS

**function [n] = count(v)**

## DESCRIPTION

```
 COUNT  Count the number of nonzero elements in a vector
```

## CROSS-REFERENCE INFORMATION

This function calls:


This function is called by:

- convert\_miqp Prepare a MIQP for solution
- add\_diff Add difference variables toa TIGER model
- average\_by\_subsystem Average gene or flux data by subsystem
- decompose\_gpr Show summary statistics on the GPR of a COBRA model
- find\_associated\_rules Find rules associated with an atom
- diffadj Formulate and solve the differential adjustment problem
- made Metabolic Adjustment by Differential Expression
- show\_made\_results Summarize results from the MADE algorithm

## SOURCE CODE

```
0001 function [n] = count(v)
0002 % COUNT  Count the number of nonzero elements in a vector
0003 
0004 n = length(find(v));
```

---

Generated on Thu 11-Aug-2011 15:06:22 by **m2html** © 2005
