# Supplementary material for: TIGER: Toolbox for integrating genome-scale metabolic models, expression data, and transcriptional regulatory networks
Source: BMC Syst Biol. 2011 Sep 23;5:147. doi: 10.1186/1752-0509-5-147 (PMC3224351; doi:10.1186/1752-0509-5-147)
Supplement: Additional file 2 — TIGER source code. Source code, documentation, and tutorials are also available online at http://bme.virginia.edu/csbl/downloads/ or http://csbl.bitbucket.org/tiger. [file 1752-0509-5-147-S2.GZ › tiger/doc/m2html/tiger/util/mapcols.html]

Description of mapcols


Home > tiger > util > mapcols.m

# mapcols

## PURPOSE

**Apply a function to columns in a matrix**

## SYNOPSIS

**function [mapped] = mapcols(f,M,nrows)**

## DESCRIPTION

```
 MAPCOLS  Apply a function to columns in a matrix

   [MAPPED] = MAPCOLS(F,M) applies the function F to each column in M:
       MAPPED(:,i) = F(M(:,i)) for each i

   MAPCOLS(F,M,NROWS) specifies the number of rows returned by F.
```

## CROSS-REFERENCE INFORMATION

This function calls:


This function is called by:


## SOURCE CODE

```
0001 function [mapped] = mapcols(f,M,nrows)
0002 % MAPCOLS  Apply a function to columns in a matrix
0003 %
0004 %   [MAPPED] = MAPCOLS(F,M) applies the function F to each column in M:
0005 %       MAPPED(:,i) = F(M(:,i)) for each i
0006 %
0007 %   MAPCOLS(F,M,NROWS) specifies the number of rows returned by F.
0008 
0009 if nargin < 3 || isempty(nrows)
0010     nrows = size(M,1);
0011 end
0012 
0013 ncols = size(M,2);
0014 mapped = zeros(nrows,ncols);
0015 for i = 1 : ncols
0016     mapped(:,i) = f(M(:,i));
0017 end
```

---

Generated on Thu 11-Aug-2011 15:06:22 by **m2html** © 2005
