# Supplementary material for: TIGER: Toolbox for integrating genome-scale metabolic models, expression data, and transcriptional regulatory networks
Source: BMC Syst Biol. 2011 Sep 23;5:147. doi: 10.1186/1752-0509-5-147 (PMC3224351; doi:10.1186/1752-0509-5-147)
Supplement: Additional file 2 — TIGER source code. Source code, documentation, and tutorials are also available online at http://bme.virginia.edu/csbl/downloads/ or http://csbl.bitbucket.org/tiger. [file 1752-0509-5-147-S2.GZ › tiger/doc/m2html/tiger/util/strbuffer.html]

Description of strbuffer


Home > tiger > util > strbuffer.m

# strbuffer

## PURPOSE

## SYNOPSIS

**This is a script file.**

## DESCRIPTION

## CROSS-REFERENCE INFORMATION

This function calls:

- strbuffer

This function is called by:

- lex Return a list of TOKENS from a string
- strbuffer

## SUBFUNCTIONS

- function [obj] = strbuffer(c)
- function append(obj,c)
- function [tf] = isempty(obj)
- function clear(obj)

## SOURCE CODE

```
0001 classdef strbuffer < handle
0002 % STRBUFFER  Extensible string buffer
0003 %
0004 %   Charater strings that can be easily extended.
0005 
0006 properties
0007     val  % string value
0008 end
0009 
0010 methods
0011     function [obj] = strbuffer(c)
0012         % STRBUFFER  Create a string buffer with an optional
0013         %            initial value.
0014         if nargin < 1
0015             obj.val = '';
0016         else
0017             obj.val = c;
0018         end
0019     end
0020 
0021     function append(obj,c)
0022         % APPEND  Add a string to the end of the buffer.
0023         obj.val = [obj.val c];
0024     end
0025 
0026     function [tf] = isempty(obj)
0027         % ISEMPTY  Overload behavior for builtin ISEMPTY function.
0028         tf = isempty(obj.val);
0029     end
0030 
0031     function clear(obj)
0032         % CLEAR  Empty the string buffer.
0033         obj.val = '';
0034     end
0035 end
0036 
0037 end % classdef
```

---

Generated on Thu 11-Aug-2011 15:06:22 by **m2html** © 2005
