# Supplementary material for: TIGER: Toolbox for integrating genome-scale metabolic models, expression data, and transcriptional regulatory networks
Source: BMC Syst Biol. 2011 Sep 23;5:147. doi: 10.1186/1752-0509-5-147 (PMC3224351; doi:10.1186/1752-0509-5-147)
Supplement: Additional file 2 — TIGER source code. Source code, documentation, and tutorials are also available online at http://bme.virginia.edu/csbl/downloads/ or http://csbl.bitbucket.org/tiger. [file 1752-0509-5-147-S2.GZ › tiger/doc/m2html/tiger/util/statusbar.html]

Description of statusbar


Home > tiger > util > statusbar.m

# statusbar

## PURPOSE

## SYNOPSIS

**This is a script file.**

## DESCRIPTION

## CROSS-REFERENCE INFORMATION

This function calls:

- statusbar

This function is called by:

- make\_c\_matrix Make reaction/gene correlation (C) matrix
- fva Flux Variability Analysis
- single\_gene\_ko Perform single gene knockout simulations
- statusbar

## SUBFUNCTIONS

- function [obj] = statusbar(Ntotal,display)
- function start(obj,msg)
- function update(obj,n)
- function [bar] = getbar(obj,n)
- function [bar] = get\_timebar(obj,n)
- function test(pause\_length)
- function [timestr] = make\_timestr(seconds)

## SOURCE CODE

```
0001 classdef statusbar < handle
0002     
0003 properties
0004     N
0005     width = 80;
0006     n = 0;
0007     
0008     show_N = true;
0009     show_percent = true;
0010     show_time = true;
0011     
0012     n_before_estimate = 3;
0013     
0014     start_tic = [];
0015     
0016     margin = '  ';
0017     barchar = '=';
0018     
0019     reprint = false;
0020     display = true;
0021     
0022     update_every = 1;
0023     last_update = 0;
0024 end
0025 
0026 methods
0027     function [obj] = statusbar(Ntotal,display)
0028         obj.N = Ntotal;
0029         
0030         if nargin == 2
0031             obj.display = display;
0032         end
0033     end
0034     
0035     function start(obj,msg)
0036         if ~obj.display
0037             return;
0038         end
0039         
0040         obj.start_tic = tic;
0041 
0042         if nargin < 2 || isempty(msg)
0043             fprintf('\n');
0044         else
0045             fprintf('\n%s:\n',msg);
0046         end
0047         
0048         obj.update(0);
0049     end
0050     
0051     function update(obj,n)
0052         if ~obj.display ...
0053               || ((n - obj.last_update) < obj.update_every && n > 0)
0054             return;
0055         end
0056 
0057         obj.last_update = n;
0058         
0059         bar = obj.getbar(n);
0060         
0061         if n > 0
0062             if ~obj.reprint
0063                 fprintf(repmat('\b',1,length(bar)));
0064             else
0065                 fprintf('\n');
0066             end
0067         end
0068         fprintf('%s',bar);
0069         
0070         if n >= obj.N
0071             fprintf('\n');
0072         end
0073     end
0074     
0075     function [bar] = getbar(obj,n)
0076         trailer = '';
0077         frac = n / obj.N;
0078         
0079         if obj.show_N
0080             Nlen = length(sprintf('%i',obj.N));
0081             trailer = sprintf(' %*i/%*i',Nlen,n,Nlen,obj.N);
0082         end
0083         if obj.show_percent
0084             trailer = sprintf('%s (%5.1f%%)',trailer,frac*100);
0085         end
0086         
0087         if obj.show_time
0088             timebar = obj.get_timebar(n);
0089         else
0090             timebar = '';
0091         end
0092         
0093         barwidth = obj.width - 2*length(obj.margin) - length(trailer) ...
0094                       - length(timebar) - 2;
0095         filled = ceil(frac*barwidth);
0096         if filled == barwidth && n == obj.N
0097             indbar = repmat(obj.barchar,1,barwidth);
0098         elseif filled == 0
0099             indbar = ['>' repmat(' ',1,barwidth - 1)];
0100         else
0101             indbar = [repmat(obj.barchar,1,filled-1) '>' ...
0102                       repmat(' ',1,barwidth - filled)];
0103         end
0104         
0105         if ~isempty(timebar)
0106             timebar = [timebar ' '];
0107         end
0108         bar = [obj.margin timebar '[' indbar ']' trailer obj.margin];
0109     end
0110     
0111     function [bar] = get_timebar(obj,n)
0112         if n < obj.n_before_estimate
0113             timestr = '--:--:--';
0114         else
0115             elapsed = toc(obj.start_tic);
0116             estimate = (obj.N - n) * elapsed / n + (n < obj.N);
0117             timestr = statusbar.make_timestr(estimate);
0118         end
0119         
0120         bar = sprintf('E%s R%s', ...
0121                       statusbar.make_timestr(toc(obj.start_tic)), ...
0122                       timestr);
0123     end
0124 end
0125 
0126 methods (Static)
0127     function test(pause_length)
0128         if nargin == 0
0129             pause_length = 0.1;
0130         end
0131         
0132         N = 100;
0133         s = statusbar(N);
0134         s.start('Testing statusbar');
0135         for i = 1 : N
0136             s.update(i);
0137             pause(pause_length);
0138         end
0139     end
0140     
0141     function [timestr] = make_timestr(seconds)
0142         timestr = datestr(datevec(num2str(seconds),'SS'),'HH:MM:SS');
0143     end
0144 end
0145 
0146 end
0147
```

---

Generated on Thu 11-Aug-2011 15:06:22 by **m2html** © 2005
