# Supplementary material for: TIGER: Toolbox for integrating genome-scale metabolic models, expression data, and transcriptional regulatory networks
Source: BMC Syst Biol. 2011 Sep 23;5:147. doi: 10.1186/1752-0509-5-147 (PMC3224351; doi:10.1186/1752-0509-5-147)
Supplement: Additional file 2 — TIGER source code. Source code, documentation, and tutorials are also available online at http://bme.virginia.edu/csbl/downloads/ or http://csbl.bitbucket.org/tiger. [file 1752-0509-5-147-S2.GZ › tiger/doc/m2html/tiger/util/struct2list.html]

Description of struct2list


Home > tiger > util > struct2list.m

# struct2list

## PURPOSE

**Convert a structure to a parameter list**

## SYNOPSIS

**function [list] = struct2list(s)**

## DESCRIPTION

```
 STRUCT2LIST  Convert a structure to a parameter list

   [LIST] = STRUCT2LIST(S)

   Example:
   >> S.A = 1;
   >> S.B = 'test';
   >> struct2list(S)
   ans = 
       'A'   [1]   'B'   'test'
```

## CROSS-REFERENCE INFORMATION

This function calls:


This function is called by:

- eva Enzyme variability analysis

## SOURCE CODE

```
0001 function [list] = struct2list(s)
0002 % STRUCT2LIST  Convert a structure to a parameter list
0003 %
0004 %   [LIST] = STRUCT2LIST(S)
0005 %
0006 %   Example:
0007 %   >> S.A = 1;
0008 %   >> S.B = 'test';
0009 %   >> struct2list(S)
0010 %   ans =
0011 %       'A'   [1]   'B'   'test'
0012 
0013 names = fieldnames(s);
0014 Nnames = length(names);
0015 
0016 list = cell(1,2*Nnames);
0017 for i = 1 : Nnames
0018     list{2*(i-1)+1} = names{i};
0019     list{2*i} = s.(names{i});
0020 end
0021
```

---

Generated on Thu 11-Aug-2011 15:06:22 by **m2html** © 2005
