# Supplementary material for: TIGER: Toolbox for integrating genome-scale metabolic models, expression data, and transcriptional regulatory networks
Source: BMC Syst Biol. 2011 Sep 23;5:147. doi: 10.1186/1752-0509-5-147 (PMC3224351; doi:10.1186/1752-0509-5-147)
Supplement: Additional file 2 — TIGER source code. Source code, documentation, and tutorials are also available online at http://bme.virginia.edu/csbl/downloads/ or http://csbl.bitbucket.org/tiger. [file 1752-0509-5-147-S2.GZ › tiger/doc/m2html/tiger/util/hash.html]

Description of hash


Home > tiger > util > hash.m

# hash

## PURPOSE

## SYNOPSIS

**This is a script file.**

## DESCRIPTION

## CROSS-REFERENCE INFORMATION

This function calls:

- hash

This function is called by:

- hash

## SUBFUNCTIONS

- function [h] = hash()
- function set(h,ks,vs)
- function [vs] = get(h,ks)
- function [vs] = subsref(h,s)
- function [str] = join(list,spacer,quote)
- function [tf,loc] = isin(A,S)

## SOURCE CODE

```
0001 classdef hash < handle
0002     
0003 properties
0004     keys
0005     vals
0006 end
0007 
0008 methods
0009     function [h] = hash()
0010         h.keys = {};
0011         h.vals = {};
0012     end
0013     
0014     function set(h,ks,vs)
0015         h.keys = [h.keys ks];
0016         h.vals = [h.vals vs];
0017     end
0018     
0019     function [vs] = get(h,ks)
0020         [tf,loc] = h.isin(ks,h.keys);
0021         if ~all(tf)
0022             error(['Undefined key(s): ' hash.join(ks(~tf),' ',true)]);
0023         else
0024             vs = h.vals(loc);
0025         end
0026     end
0027             
0028     function [vs] = subsref(h,s)
0029         switch s(1).type
0030             case '{}'
0031                 vs = h.get(s(1).subs);
0032                 if length(s) > 1
0033                     s = s(2:end);
0034                     vs = cellfun(@(x) subsref(x,s), vs, ...
0035                                  'UniformOutput', false);
0036                 end
0037                 if length(vs) == 1
0038                     vs = vs{1};
0039                 end
0040             case '.'
0041                 props = {'keys','vals'};
0042                 meths = methods(hash);
0043                 if ismember(s(1).subs,meths)
0044                     if length(s) > 1
0045                         vs = feval(s(1).subs,h,s(2).subs);
0046                     else
0047                         vs = feval(s(1).subs,h);
0048                     end
0049                 elseif ismember(s(1).subs,props)
0050                     vs = h.(s(1).subs);
0051                 else
0052                     error(['??? No appropriate method, property,' ...
0053                            ' or field %s for class hash'],s(1).subs);
0054                 end
0055             otherwise
0056                 error('Use hash{''key''} to refernce hashes');
0057         end
0058     end
0059 end
0060 
0061 methods (Static,Access = private)
0062     function [str] = join(list,spacer,quote)
0063         if nargin < 2,  spacer = ' '; end
0064         if nargin == 3 && quote
0065             list = cellfun(@(x) ['''' x ''''],list,'UniformOutput',false);
0066         end
0067         str = '';
0068         for i = 1 : length(list) - 1
0069             str = [str list{i} spacer];
0070         end
0071         str = [str list{end}];
0072     end
0073     
0074     function [tf,loc] = isin(A,S)
0075         tf = false(size(A));
0076         loc = zeros(size(A));
0077         for i = 1 : length(A)
0078             for j = 1 : length(S)
0079                 if isa(A{i},class(S{j})) && A{i} == S{j}
0080                     tf(i) = true;
0081                     loc(i) = j;
0082                     break;
0083                 end
0084             end
0085         end
0086     end
0087 end
0088 
0089 end % classdef
0090
```

---

Generated on Thu 11-Aug-2011 15:06:22 by **m2html** © 2005
