# Supplementary material for: TIGER: Toolbox for integrating genome-scale metabolic models, expression data, and transcriptional regulatory networks
Source: BMC Syst Biol. 2011 Sep 23;5:147. doi: 10.1186/1752-0509-5-147 (PMC3224351; doi:10.1186/1752-0509-5-147)
Supplement: Additional file 2 — TIGER source code. Source code, documentation, and tutorials are also available online at http://bme.virginia.edu/csbl/downloads/ or http://csbl.bitbucket.org/tiger. [file 1752-0509-5-147-S2.GZ › tiger/doc/m2html/tiger/util/array2names.html]

Description of array2names


Home > tiger > util > array2names.m

# array2names

## PURPOSE

**Create a cell of names from an array of numbers**

## SYNOPSIS

**function [names] = array2names(fmt,array,dim)**

## DESCRIPTION

```
 ARRAY2NAMES  Create a cell of names from an array of numbers

   [NAMES] = ARRAY2NAMES(FMT,ARRAY,DIM)

   Using a printf format string FMT that accepts a single integer, 
   create a cell of names that are sequentially numbered by the values
   in array.  If DIM = 1, the result is a column cell.  Otherwise, the
   result is a row cell.

   Example:
       array2names('var%i',1:3)
       ans = 
           'var1'
           'var2'
           'var3'
```

## CROSS-REFERENCE INFORMATION

This function calls:


This function is called by:

- add\_column Add a column to a TIGER model structure
- add\_row Add a row to a TIGER model structure
- add\_rule Add rules to a TIGER model
- cobra\_to\_tiger Convert a COBRA model to a TIGER model
- cobra\_to\_elf Create an ELF model from a COBRA structure
- find\_infeasible\_rules Determine which rules make a model infeasible.
- cobra\_model Test model in COBRA format
- diffadj Formulate and solve the differential adjustment problem
- create\_table Format and display tabular data

## SOURCE CODE

```
0001 function [names] = array2names(fmt,array,dim)
0002 % ARRAY2NAMES  Create a cell of names from an array of numbers
0003 %
0004 %   [NAMES] = ARRAY2NAMES(FMT,ARRAY,DIM)
0005 %
0006 %   Using a printf format string FMT that accepts a single integer,
0007 %   create a cell of names that are sequentially numbered by the values
0008 %   in array.  If DIM = 1, the result is a column cell.  Otherwise, the
0009 %   result is a row cell.
0010 %
0011 %   Example:
0012 %       array2names('var%i',1:3)
0013 %       ans =
0014 %           'var1'
0015 %           'var2'
0016 %           'var3'
0017 
0018 if nargin < 3 || isempty(dim)
0019     dim = 1;
0020 end
0021 
0022 names = arrayfun(@(x) sprintf(fmt,x),array,'Uniform',false);
0023 if dim == 1
0024     names = names(:);
0025 end
0026
```

---

Generated on Thu 11-Aug-2011 15:06:22 by **m2html** © 2005
