# Supplementary material for: TIGER: Toolbox for integrating genome-scale metabolic models, expression data, and transcriptional regulatory networks
Source: BMC Syst Biol. 2011 Sep 23;5:147. doi: 10.1186/1752-0509-5-147 (PMC3224351; doi:10.1186/1752-0509-5-147)
Supplement: Additional file 2 — TIGER source code. Source code, documentation, and tutorials are also available online at http://bme.virginia.edu/csbl/downloads/ or http://csbl.bitbucket.org/tiger. [file 1752-0509-5-147-S2.GZ › tiger/doc/m2html/tiger/util/showif.html]

Description of showif


Home > tiger > util > showif.m

# showif

## PURPOSE

**Conditionally display to the command window**

## SYNOPSIS

**function showif(tf,fmt,varargin)**

## DESCRIPTION

```
 SHOWIF  Conditionally display to the command window

   SHOWIF(TF,FMT,...) prints the PRINTF format string FMT if TF is true.
   It is silent otherwise.  Additional arguments are passed to FPRITNF.
```

## CROSS-REFERENCE INFORMATION

This function calls:


This function is called by:


## SOURCE CODE

```
0001 function showif(tf,fmt,varargin)
0002 % SHOWIF  Conditionally display to the command window
0003 %
0004 %   SHOWIF(TF,FMT,...) prints the PRINTF format string FMT if TF is true.
0005 %   It is silent otherwise.  Additional arguments are passed to FPRITNF.
0006 
0007 if tf
0008     fprintf(fmt,varargin{:});
0009 end
```

---

Generated on Thu 11-Aug-2011 15:06:22 by **m2html** © 2005
