# Supplementary material for: TIGER: Toolbox for integrating genome-scale metabolic models, expression data, and transcriptional regulatory networks
Source: BMC Syst Biol. 2011 Sep 23;5:147. doi: 10.1186/1752-0509-5-147 (PMC3224351; doi:10.1186/1752-0509-5-147)
Supplement: Additional file 2 — TIGER source code. Source code, documentation, and tutorials are also available online at http://bme.virginia.edu/csbl/downloads/ or http://csbl.bitbucket.org/tiger. [file 1752-0509-5-147-S2.GZ › tiger/doc/m2html/tiger/util/create_table.html]

Description of create\_table


Home > tiger > util > create\_table.m

# create\_table

## PURPOSE

**Format and display tabular data**

## SYNOPSIS

**function [table] = create\_table(data,varargin)**

## DESCRIPTION

```
 CREATE_TABLE  Format and display tabular data

   [TABLE] = CREATE_TABLE(DATA,...params...)

   Create and display a matrix or cell array DATA in tabular form.
   Parameters are:
       'spacer'    String placed between columns of data.
                   (Default is '  ')
       'numfmt'    If DATA is a matrix, this format string is used to
                   convert the entries to strings.  Default is '%f'.
       'rowfmt'    A cell array of format strings, one for each column in
                   DATA.  Must have the same number of columns as DATA.
                   This parameter overrides 'numfmt'.
       'columnlabels'  A cell array of string labels for each column.  If
                       empty, the column index is used.
       'rowlabels'     A cell array of string labels for each column.  If
                       empty, the row index is used.
```

## CROSS-REFERENCE INFORMATION

This function calls:

- array2names Create a cell of names from an array of numbers
- map Generate a new list by applying a function
- textframe

This function is called by:

- test\_\_create\_table
- show\_made\_results Summarize results from the MADE algorithm

## SOURCE CODE

```
0001 function [table] = create_table(data,varargin)
0002 % CREATE_TABLE  Format and display tabular data
0003 %
0004 %   [TABLE] = CREATE_TABLE(DATA,...params...)
0005 %
0006 %   Create and display a matrix or cell array DATA in tabular form.
0007 %   Parameters are:
0008 %       'spacer'    String placed between columns of data.
0009 %                   (Default is '  ')
0010 %       'numfmt'    If DATA is a matrix, this format string is used to
0011 %                   convert the entries to strings.  Default is '%f'.
0012 %       'rowfmt'    A cell array of format strings, one for each column in
0013 %                   DATA.  Must have the same number of columns as DATA.
0014 %                   This parameter overrides 'numfmt'.
0015 %       'columnlabels'  A cell array of string labels for each column.  If
0016 %                       empty, the column index is used.
0017 %       'rowlabels'     A cell array of string labels for each column.  If
0018 %                       empty, the row index is used.
0019 
0020 [m,n] = size(data);
0021 
0022 p = inputParser;
0023 p.addParamValue('spacer','  ');
0024 p.addParamValue('numfmt','%f');
0025 p.addParamValue('rowfmt',{});
0026 p.addParamValue('columnlabels',array2names('%i',1:n));
0027 p.addParamValue('rowlabels',array2names('%i',1:m));
0028 p.parse(varargin{:});
0029 
0030 spacer = p.Results.spacer;
0031 numfmt = p.Results.numfmt;
0032 rowfmt = p.Results.rowfmt;
0033 col_headings = p.Results.columnlabels;
0034 row_headings = p.Results.rowlabels;
0035 
0036 if ~isa(data,'cell')
0037     X = cell(m,n);
0038     for i = 1 : m
0039         for j = 1 : n
0040             if isempty(rowfmt)
0041                 X{i,j} = sprintf(numfmt,data(i,j));
0042             else
0043                 X{i,j} = sprintf(rowfmt{j},data(i,j));
0044             end
0045         end
0046     end
0047     data = X;
0048 end
0049 
0050 columns = cell(1,n);
0051 columns{1} = textframe(' ');
0052 for i = 1 : n
0053     columns{i+1} = textframe(col_headings{i});
0054 end
0055 for i = 1 : m
0056     columns{1}.add_line(row_headings{i});
0057     for j = 1 : n
0058         columns{j+1}.add_line(data{i,j});
0059     end
0060 end
0061 
0062 tfs = map(@(x) x.make_block('halign','right'),columns);
0063 
0064 table = hcat(tfs{:},'spacer',spacer);
0065 
0066
```

---

Generated on Thu 11-Aug-2011 15:06:22 by **m2html** © 2005
