# Supplementary material for: TIGER: Toolbox for integrating genome-scale metabolic models, expression data, and transcriptional regulatory networks
Source: BMC Syst Biol. 2011 Sep 23;5:147. doi: 10.1186/1752-0509-5-147 (PMC3224351; doi:10.1186/1752-0509-5-147)
Supplement: Additional file 2 — TIGER source code. Source code, documentation, and tutorials are also available online at http://bme.virginia.edu/csbl/downloads/ or http://csbl.bitbucket.org/tiger. [file 1752-0509-5-147-S2.GZ › tiger/doc/m2html/tiger/util/find_like.html]

Description of find\_like


Home > tiger > util > find\_like.m

# find\_like

## PURPOSE

**Find matches in a cell of strings**

## SYNOPSIS

**function [matches,locs,tf] = find\_like(regex,C)**

## DESCRIPTION

```
 FIND_LIKE  Find matches in a cell of strings

   [MATCHES,LOCS,TF] = FIND_LIKE(REGEX,C)

   Returns the elements of C that match the regular expression REGEX.
   The locations of the elements are LOCS.  TF is a logical indexing 
   array such that MATCHES = C(TF) = C(LOCS).
```

## CROSS-REFERENCE INFORMATION

This function calls:

- cellfilter Return a subset of a cell array

This function is called by:

- remove\_rev\_cons Remove reversibility constraints from an ELF model
- restore\_rev\_cons Restore reversibility constraints in an ELF model
- create\_yeast\_trn\_model
- load\_rules
- imat Integrative Metabolic Analysis Tool

## SOURCE CODE

```
0001 function [matches,locs,tf] = find_like(regex,C)
0002 % FIND_LIKE  Find matches in a cell of strings
0003 %
0004 %   [MATCHES,LOCS,TF] = FIND_LIKE(REGEX,C)
0005 %
0006 %   Returns the elements of C that match the regular expression REGEX.
0007 %   The locations of the elements are LOCS.  TF is a logical indexing
0008 %   array such that MATCHES = C(TF) = C(LOCS).
0009 
0010 f = @(x) ~isempty(regexp(x,regex,'once'));
0011 [matches,locs,tf] = cellfilter(f,C);
```

---

Generated on Thu 11-Aug-2011 15:06:22 by **m2html** © 2005
