# Supplementary material for: TIGER: Toolbox for integrating genome-scale metabolic models, expression data, and transcriptional regulatory networks
Source: BMC Syst Biol. 2011 Sep 23;5:147. doi: 10.1186/1752-0509-5-147 (PMC3224351; doi:10.1186/1752-0509-5-147)
Supplement: Additional file 2 — TIGER source code. Source code, documentation, and tutorials are also available online at http://bme.virginia.edu/csbl/downloads/ or http://csbl.bitbucket.org/tiger. [file 1752-0509-5-147-S2.GZ › tiger/doc/m2html/tiger/util/scanfile.html]

Description of scanfile


Home > tiger > util > scanfile.m

# scanfile

## PURPOSE

**Apply TEXTSCAN to a filename**

## SYNOPSIS

**function [data] = scanfile(filename,format,varargin)**

## DESCRIPTION

```
 SCANFILE  Apply TEXTSCAN to a filename

   [DATA] = SCANFILE(FILENAME,FORMAT,...params...)

   Uses TEXTSCAN to parse a file named FILENAME using the format string
   FORMAT.  Additional parameters are passed to TEXTSCAN.  Returns the
   TEXTSCAN structure.
```

## CROSS-REFERENCE INFORMATION

This function calls:


This function is called by:


## SOURCE CODE

```
0001 function [data] = scanfile(filename,format,varargin)
0002 % SCANFILE  Apply TEXTSCAN to a filename
0003 %
0004 %   [DATA] = SCANFILE(FILENAME,FORMAT,...params...)
0005 %
0006 %   Uses TEXTSCAN to parse a file named FILENAME using the format string
0007 %   FORMAT.  Additional parameters are passed to TEXTSCAN.  Returns the
0008 %   TEXTSCAN structure.
0009 
0010 fid = fopen(filename);
0011 data = textscan(fid,format,varargin{:});
0012 fclose(fid);
0013
```

---

Generated on Thu 11-Aug-2011 15:06:22 by **m2html** © 2005
