# Supplementary material for: TIGER: Toolbox for integrating genome-scale metabolic models, expression data, and transcriptional regulatory networks
Source: BMC Syst Biol. 2011 Sep 23;5:147. doi: 10.1186/1752-0509-5-147 (PMC3224351; doi:10.1186/1752-0509-5-147)
Supplement: Additional file 2 — TIGER source code. Source code, documentation, and tutorials are also available online at http://bme.virginia.edu/csbl/downloads/ or http://csbl.bitbucket.org/tiger. [file 1752-0509-5-147-S2.GZ › tiger/doc/m2html/tiger/util/celliter.html]

Description of celliter


Home > tiger > util > celliter.m

# celliter

## PURPOSE

**Iterate over elements in a cell**

## SYNOPSIS

**function celliter(f,C)**

## DESCRIPTION

```
 CELLITER  Iterate over elements in a cell

   CELLITER(F,C) iterates over each element in cell C, calling F(C{i}).
```

## CROSS-REFERENCE INFORMATION

This function calls:


This function is called by:

- tile\_mip Combine several MIPs into a single structure
- create\_yeast\_trn\_model
- load\_rules

## SOURCE CODE

```
0001 function celliter(f,C)
0002 % CELLITER  Iterate over elements in a cell
0003 %
0004 %   CELLITER(F,C) iterates over each element in cell C, calling F(C{i}).
0005 
0006 for i = 1 : length(C)
0007     f(C{i});
0008 end
0009
```

---

Generated on Thu 11-Aug-2011 15:06:22 by **m2html** © 2005
