# Supplementary material for: TIGER: Toolbox for integrating genome-scale metabolic models, expression data, and transcriptional regulatory networks
Source: BMC Syst Biol. 2011 Sep 23;5:147. doi: 10.1186/1752-0509-5-147 (PMC3224351; doi:10.1186/1752-0509-5-147)
Supplement: Additional file 2 — TIGER source code. Source code, documentation, and tutorials are also available online at http://bme.virginia.edu/csbl/downloads/ or http://csbl.bitbucket.org/tiger. [file 1752-0509-5-147-S2.GZ › tiger/doc/m2html/tiger/util/assert_cell.html]

Description of assert\_cell


Home > tiger > util > assert\_cell.m

# assert\_cell

## PURPOSE

**Assert that variable is a cell array.**

## SYNOPSIS

**function [c,was\_cell] = assert\_cell(str)**

## DESCRIPTION

```
 ASSERT_CELL  Assert that variable is a cell array.

   [C,WAS_CELL] = ASSERT_CELL(STR)
   
   Checks that STR is a cell array.  If it is not (i.e., if it is a
   single string), then converts it to a cell array of length one.

   WAS_CELL is true if STR was originially a cell.
```

## CROSS-REFERENCE INFORMATION

This function calls:


This function is called by:

- add\_rule Add rules to a TIGER model
- find\_associated\_rules Find rules associated with an atom
- parse\_string Parse a rule string into an EXPR object
- remove\_rule Remove rule(s) previously added to a TIGER model
- diffadj Formulate and solve the differential adjustment problem
- textframe

## SOURCE CODE

```
0001 function [c,was_cell] = assert_cell(str)
0002 % ASSERT_CELL  Assert that variable is a cell array.
0003 %
0004 %   [C,WAS_CELL] = ASSERT_CELL(STR)
0005 %
0006 %   Checks that STR is a cell array.  If it is not (i.e., if it is a
0007 %   single string), then converts it to a cell array of length one.
0008 %
0009 %   WAS_CELL is true if STR was originially a cell.
0010 
0011 was_cell = isa(str,'cell');
0012 
0013 if isempty(str) && ~isa(str,'char')
0014     c = {};
0015 elseif ~isa(str,'cell')
0016     c = {str};
0017 else
0018     c = str;
0019 end
```

---

Generated on Thu 11-Aug-2011 15:06:22 by **m2html** © 2005
