# Supplementary material for: TIGER: Toolbox for integrating genome-scale metabolic models, expression data, and transcriptional regulatory networks
Source: BMC Syst Biol. 2011 Sep 23;5:147. doi: 10.1186/1752-0509-5-147 (PMC3224351; doi:10.1186/1752-0509-5-147)
Supplement: Additional file 2 — TIGER source code. Source code, documentation, and tutorials are also available online at http://bme.virginia.edu/csbl/downloads/ or http://csbl.bitbucket.org/tiger. [file 1752-0509-5-147-S2.GZ › tiger/doc/m2html/tiger/util/argf.html]

Description of argf


Home > tiger > util > argf.m

# argf

## PURPOSE

**Return the index vector for a function**

## SYNOPSIS

**function [locs] = argf(f,x,N)**

## DESCRIPTION

```
 ARGF Return the index vector for a function

   [LOCS] = ARGF(F,X) returns [~,LOCS] = F(X).  If called as ARGF(F,X,N),
   a maximum of N arguments are returned.
```

## CROSS-REFERENCE INFORMATION

This function calls:


This function is called by:

- argmax Return the arg-maximum of a function
- argmin Return the arg-minimum of a function

## SOURCE CODE

```
0001 function [locs] = argf(f,x,N)
0002 % ARGF Return the index vector for a function
0003 %
0004 %   [LOCS] = ARGF(F,X) returns [~,LOCS] = F(X).  If called as ARGF(F,X,N),
0005 %   a maximum of N arguments are returned.
0006 
0007 if nargin < 3 || isempty(N)
0008     N = 1;
0009 end
0010 
0011 if isempty(x)
0012     locs = [];
0013 else
0014     [~,locs] = f(x);
0015     locs = locs(1:min([N length(locs)]));
0016 end
```

---

Generated on Thu 11-Aug-2011 15:06:22 by **m2html** © 2005
