# Supplementary material for: TIGER: Toolbox for integrating genome-scale metabolic models, expression data, and transcriptional regulatory networks
Source: BMC Syst Biol. 2011 Sep 23;5:147. doi: 10.1186/1752-0509-5-147 (PMC3224351; doi:10.1186/1752-0509-5-147)
Supplement: Additional file 2 — TIGER source code. Source code, documentation, and tutorials are also available online at http://bme.virginia.edu/csbl/downloads/ or http://csbl.bitbucket.org/tiger. [file 1752-0509-5-147-S2.GZ › tiger/doc/m2html/tiger/util/expand_to.html]

Description of expand\_to


Home > tiger > util > expand\_to.m

# expand\_to

## PURPOSE

**Expand a vector to length N**

## SYNOPSIS

**function [x] = expand\_to(x,N,dim)**

## DESCRIPTION

```
 EXPAND_TO  Expand a vector to length N

   [X] = EXPAND_TO(X,N,DIM)
   [X] = EXPAND_TO(X,[M N])

   Expands the vector X to length N by filling with zeros.  If a vector 
   sizes is given, X is expanded as a M by N matrix.

   If X is empty, a vector of zeros is created.  If DIM = 1 (default), 
   the result is a column vector.  If DIM = 2, the a row vector is
   returned.
```

## CROSS-REFERENCE INFORMATION

This function calls:


This function is called by:

- check\_mip Ensure that the sense, ind, and indtypes fields are filled
- add\_column Add a column to a TIGER model structure
- add\_row Add a row to a TIGER model structure

## SOURCE CODE

```
0001 function [x] = expand_to(x,N,dim)
0002 % EXPAND_TO  Expand a vector to length N
0003 %
0004 %   [X] = EXPAND_TO(X,N,DIM)
0005 %   [X] = EXPAND_TO(X,[M N])
0006 %
0007 %   Expands the vector X to length N by filling with zeros.  If a vector
0008 %   sizes is given, X is expanded as a M by N matrix.
0009 %
0010 %   If X is empty, a vector of zeros is created.  If DIM = 1 (default),
0011 %   the result is a column vector.  If DIM = 2, the a row vector is
0012 %   returned.
0013 
0014 if nargin < 4 || isempty(dim)
0015     dim = 1;
0016 end
0017     
0018 assert(nargin >= 2, 'at least two arguments required');
0019 
0020 if length(N) == 2
0021     dims = N;
0022 elseif dim == 1
0023     dims = [N,1];
0024 else
0025     dims = [1,N];
0026 end
0027 
0028 if any(size(x) == 0)
0029     x = zeros(dims);
0030 elseif ~all(size(x) == dims)
0031     x(dims(1),dims(2)) = 0;
0032 end
```

---

Generated on Thu 11-Aug-2011 15:06:22 by **m2html** © 2005
