# Supplementary material for: TIGER: Toolbox for integrating genome-scale metabolic models, expression data, and transcriptional regulatory networks
Source: BMC Syst Biol. 2011 Sep 23;5:147. doi: 10.1186/1752-0509-5-147 (PMC3224351; doi:10.1186/1752-0509-5-147)
Supplement: Additional file 2 — TIGER source code. Source code, documentation, and tutorials are also available online at http://bme.virginia.edu/csbl/downloads/ or http://csbl.bitbucket.org/tiger. [file 1752-0509-5-147-S2.GZ › tiger/doc/m2html/tiger/util/max_abs.html]

Description of max\_abs


Home > tiger > util > max\_abs.m

# max\_abs

## PURPOSE

**Maximum absolute value in a set of vectors**

## SYNOPSIS

**function [maxabs] = max\_abs(varargin)**

## DESCRIPTION

```
 MAX_ABS  Maximum absolute value in a set of vectors

   [MAXABS] = MAX_ABS(...)

   MAX_ABS(A,B,C,...) finds MAX(|A|,|B|,|C|,...), the single largest
   absolute value in any of the given vectors.

   Examples:
   >> max_abs([1 2 11],[-12 5 3])
   ans = 
       12
```

## CROSS-REFERENCE INFORMATION

This function calls:


This function is called by:


## SOURCE CODE

```
0001 function [maxabs] = max_abs(varargin)
0002 % MAX_ABS  Maximum absolute value in a set of vectors
0003 %
0004 %   [MAXABS] = MAX_ABS(...)
0005 %
0006 %   MAX_ABS(A,B,C,...) finds MAX(|A|,|B|,|C|,...), the single largest
0007 %   absolute value in any of the given vectors.
0008 %
0009 %   Examples:
0010 %   >> max_abs([1 2 11],[-12 5 3])
0011 %   ans =
0012 %       12
0013 
0014 cands = cellfun(@(x) max(abs(x)),varargin);
0015 maxabs = max(cands);
0016
```

---

Generated on Thu 11-Aug-2011 15:06:22 by **m2html** © 2005
