# Supplementary material for: TIGER: Toolbox for integrating genome-scale metabolic models, expression data, and transcriptional regulatory networks
Source: BMC Syst Biol. 2011 Sep 23;5:147. doi: 10.1186/1752-0509-5-147 (PMC3224351; doi:10.1186/1752-0509-5-147)
Supplement: Additional file 2 — TIGER source code. Source code, documentation, and tutorials are also available online at http://bme.virginia.edu/csbl/downloads/ or http://csbl.bitbucket.org/tiger. [file 1752-0509-5-147-S2.GZ › tiger/doc/m2html/tiger/util/maprows.html]

Description of maprows


Home > tiger > util > maprows.m

# maprows

## PURPOSE

**Apply a function to rows in a matrix**

## SYNOPSIS

**function [mapped] = maprows(f,M,ncols)**

## DESCRIPTION

```
 MAPROWS  Apply a function to rows in a matrix

   [MAPPED] = MAPROWS(F,M) applies the function F to each row in M:
       MAPPED(i,:) = F(M(i,:)) for each i

   MAPROWS(F,M,NCOLS) specifies the number of columns returned by F.
```

## CROSS-REFERENCE INFORMATION

This function calls:


This function is called by:


## SOURCE CODE

```
0001 function [mapped] = maprows(f,M,ncols)
0002 % MAPROWS  Apply a function to rows in a matrix
0003 %
0004 %   [MAPPED] = MAPROWS(F,M) applies the function F to each row in M:
0005 %       MAPPED(i,:) = F(M(i,:)) for each i
0006 %
0007 %   MAPROWS(F,M,NCOLS) specifies the number of columns returned by F.
0008 
0009 if nargin < 3 || isempty(ncols)
0010     ncols = size(M,2);
0011 end
0012 
0013 nrows = size(M,1);
0014 mapped = zeros(nrows,ncols);
0015 for i = 1 : nrows
0016     mapped(i,:) = f(M(i,:));
0017 end
```

---

Generated on Thu 11-Aug-2011 15:06:22 by **m2html** © 2005
