# Supplementary material for: TIGER: Toolbox for integrating genome-scale metabolic models, expression data, and transcriptional regulatory networks
Source: BMC Syst Biol. 2011 Sep 23;5:147. doi: 10.1186/1752-0509-5-147 (PMC3224351; doi:10.1186/1752-0509-5-147)
Supplement: Additional file 2 — TIGER source code. Source code, documentation, and tutorials are also available online at http://bme.virginia.edu/csbl/downloads/ or http://csbl.bitbucket.org/tiger. [file 1752-0509-5-147-S2.GZ › tiger/doc/m2html/tiger/util/map.html]

Description of map


Home > tiger > util > map.m

# map

## PURPOSE

**Generate a new list by applying a function**

## SYNOPSIS

**function [mapped] = map(f,C)**

## DESCRIPTION

```
 MAP  Generate a new list by applying a function

   [MAPPED] = MAP(F,C) applies function handle F to each element in cell
   C to create a new list MAPPED from the return values, i.e., 
   MAPPED{i} = F(C{i})

   MAP also works on arrays using ARRAYFUN.  For cells and arrays with
   uniform return values, ARRAYFUN and CELLFUN are faster.
```

## CROSS-REFERENCE INFORMATION

This function calls:


This function is called by:

- tile\_mip Combine several MIPs into a single structure
- add\_diff Add difference variables toa TIGER model
- add\_rule Add rules to a TIGER model
- convert\_grRules Parse grRules into rules for the COBRA toolbox
- convert\_gpr Add the GPR rules as constraints to the model.
- cobra\_to\_elf Create an ELF model from a COBRA structure
- find\_associated\_rules Find rules associated with an atom
- find\_infeasible\_rules Determine which rules make a model infeasible.
- make\_dnf Make lists of atoms in the Disjunctive Normal Form
- parse\_string Parse a rule string into an EXPR object
- remove\_rule Remove rule(s) previously added to a TIGER model
- show\_exchange\_rxns Show exchange reactions (and fluxes)
- load\_rules
- diffadj Formulate and solve the differential adjustment problem
- imat Integrative Metabolic Analysis Tool
- cellzipn Zip an unlimited number of cell arrays by a function
- create\_table Format and display tabular data

## SOURCE CODE

```
0001 function [mapped] = map(f,C)
0002 % MAP  Generate a new list by applying a function
0003 %
0004 %   [MAPPED] = MAP(F,C) applies function handle F to each element in cell
0005 %   C to create a new list MAPPED from the return values, i.e.,
0006 %   MAPPED{i} = F(C{i})
0007 %
0008 %   MAP also works on arrays using ARRAYFUN.  For cells and arrays with
0009 %   uniform return values, ARRAYFUN and CELLFUN are faster.
0010 
0011 if isa(C,'cell')
0012     mapped = cellfun(f,C,'Uniform',false);
0013 elseif isa(C,'double') || length(C) > 1
0014     mapped = arrayfun(f,C,'Uniform',false);
0015 else
0016     mapped = cellfun(f,{C},'Uniform',false);
0017 end
0018 
0019
```

---

Generated on Thu 11-Aug-2011 15:06:22 by **m2html** © 2005
