# Supplementary material for: TIGER: Toolbox for integrating genome-scale metabolic models, expression data, and transcriptional regulatory networks
Source: BMC Syst Biol. 2011 Sep 23;5:147. doi: 10.1186/1752-0509-5-147 (PMC3224351; doi:10.1186/1752-0509-5-147)
Supplement: Additional file 2 — TIGER source code. Source code, documentation, and tutorials are also available online at http://bme.virginia.edu/csbl/downloads/ or http://csbl.bitbucket.org/tiger. [file 1752-0509-5-147-S2.GZ › tiger/doc/m2html/tiger/util/flatten.html]

Description of flatten


Home > tiger > util > flatten.m

# flatten

## PURPOSE

**Flatten a cell of cells into a single cell.**

## SYNOPSIS

**function [list] = flatten(lists)**

## DESCRIPTION

```
 FLATTEN  Flatten a cell of cells into a single cell.

   >> A = FLATTEN({{1,2},3,{4}})
   A = 
       [1]  [2]  [3]  [4]
```

## CROSS-REFERENCE INFORMATION

This function calls:


This function is called by:


## SOURCE CODE

```
0001 function [list] = flatten(lists)
0002 % FLATTEN  Flatten a cell of cells into a single cell.
0003 %
0004 %   >> A = FLATTEN({{1,2},3,{4}})
0005 %   A =
0006 %       [1]  [2]  [3]  [4]
0007 
0008 list = [lists{:}];
```

---

Generated on Thu 11-Aug-2011 15:06:22 by **m2html** © 2005
