# Supplementary material for: TIGER: Toolbox for integrating genome-scale metabolic models, expression data, and transcriptional regulatory networks
Source: BMC Syst Biol. 2011 Sep 23;5:147. doi: 10.1186/1752-0509-5-147 (PMC3224351; doi:10.1186/1752-0509-5-147)
Supplement: Additional file 2 — TIGER source code. Source code, documentation, and tutorials are also available online at http://bme.virginia.edu/csbl/downloads/ or http://csbl.bitbucket.org/tiger. [file 1752-0509-5-147-S2.GZ › tiger/doc/m2html/tiger/util/argmin.html]

Description of argmin


Home > tiger > util > argmin.m

# argmin

## PURPOSE

**Return the arg-minimum of a function**

## SYNOPSIS

**function [locs] = argmin(x,N)**

## DESCRIPTION

```
 ARGMIN Return the arg-minimum of a function

   [LOCS] = ARGMIN(X) returns the indices of the minimum values in X.
   If called as ARGMIN(X,N), a maximum of N indices are returned.
```

## CROSS-REFERENCE INFORMATION

This function calls:

- argf Return the index vector for a function

This function is called by:


## SOURCE CODE

```
0001 function [locs] = argmin(x,N)
0002 % ARGMIN Return the arg-minimum of a function
0003 %
0004 %   [LOCS] = ARGMIN(X) returns the indices of the minimum values in X.
0005 %   If called as ARGMIN(X,N), a maximum of N indices are returned.
0006 
0007 if nargin < 2
0008     N = 1;
0009 end
0010 
0011 locs = argf(@min,x,N);
```

---

Generated on Thu 11-Aug-2011 15:06:22 by **m2html** © 2005
