# Supplementary material for: TIGER: Toolbox for integrating genome-scale metabolic models, expression data, and transcriptional regulatory networks
Source: BMC Syst Biol. 2011 Sep 23;5:147. doi: 10.1186/1752-0509-5-147 (PMC3224351; doi:10.1186/1752-0509-5-147)
Supplement: Additional file 2 — TIGER source code. Source code, documentation, and tutorials are also available online at http://bme.virginia.edu/csbl/downloads/ or http://csbl.bitbucket.org/tiger. [file 1752-0509-5-147-S2.GZ › tiger/doc/m2html/tiger/util/fill_to.html]

Description of fill\_to


Home > tiger > util > fill\_to.m

# fill\_to

## PURPOSE

**Fill a short to empty vector**

## SYNOPSIS

**function [filled] = fill\_to(x,N,default,dim)**

## DESCRIPTION

```
 FILL_TO  Fill a short to empty vector

   [FILLED] = FILL_TO(X,N,DEFAULT,DIM)

   If X has only one element, replicate it to have N elements.  If X is
   empty, use DEFAULT (DEFAULT = 0 if not specified).  Vector orientation
   is determined by DIM:  1 (default) -> column, 2 -> row.
```

## CROSS-REFERENCE INFORMATION

This function calls:


This function is called by:

- check\_mip Ensure that the sense, ind, and indtypes fields are filled
- add\_column Add a column to a TIGER model structure
- add\_row Add a row to a TIGER model structure
- set\_fieldval Set values in a TIGER structure field
- diffadj Formulate and solve the differential adjustment problem

## SOURCE CODE

```
0001 function [filled] = fill_to(x,N,default,dim)
0002 % FILL_TO  Fill a short to empty vector
0003 %
0004 %   [FILLED] = FILL_TO(X,N,DEFAULT,DIM)
0005 %
0006 %   If X has only one element, replicate it to have N elements.  If X is
0007 %   empty, use DEFAULT (DEFAULT = 0 if not specified).  Vector orientation
0008 %   is determined by DIM:  1 (default) -> column, 2 -> row.
0009 
0010 if nargin < 4 || isempty(dim)
0011     dim = 1;
0012 end
0013 assert(dim == 1 || dim == 2, 'DIM must be either 1 or 2.');
0014 
0015 if nargin < 3 || isempty(default)
0016     default = 0;
0017 end
0018 
0019 if isempty(x)
0020     filled = default;
0021 else
0022     filled = x;
0023 end
0024 
0025 if length(x) < N
0026     if dim == 1
0027         filled = repmat(filled,N,1);
0028     else
0029         filled = repmat(filled,1,N);
0030     end
0031 end
0032 
0033
```

---

Generated on Thu 11-Aug-2011 15:06:22 by **m2html** © 2005
