# Supplementary material for: TIGER: Toolbox for integrating genome-scale metabolic models, expression data, and transcriptional regulatory networks
Source: BMC Syst Biol. 2011 Sep 23;5:147. doi: 10.1186/1752-0509-5-147 (PMC3224351; doi:10.1186/1752-0509-5-147)
Supplement: Additional file 2 — TIGER source code. Source code, documentation, and tutorials are also available online at http://bme.virginia.edu/csbl/downloads/ or http://csbl.bitbucket.org/tiger. [file 1752-0509-5-147-S2.GZ › tiger/doc/m2html/tiger/util/near.html]

Description of near


Home > tiger > util > near.m

# near

## PURPOSE

**Test if two values are close to each other**

## SYNOPSIS

**function [tf] = near(x,y,tol)**

## DESCRIPTION

```
 NEAR  Test if two values are close to each other

   [TF] = NEAR(X,Y,TOL)

   Tests if |X - Y| <= TOL.  If TOL is not given, the default is 1e-5.
   If Y is not given, the default is 0.  X and Y can be single numbers,
   vectors, or matrices.
```

## CROSS-REFERENCE INFORMATION

This function calls:


This function is called by:

- test\_\_add\_diff
- test\_\_diffadj
- test\_\_fba
- test\_\_gimme
- test\_\_imat
- test\_\_indicators
- test\_\_miqp
- test\_\_multilevel
- test\_\_remove\_rule
- test\_\_solve\_multiple\_mips
- test\_\_tile\_mip

## SOURCE CODE

```
0001 function [tf] = near(x,y,tol)
0002 % NEAR  Test if two values are close to each other
0003 %
0004 %   [TF] = NEAR(X,Y,TOL)
0005 %
0006 %   Tests if |X - Y| <= TOL.  If TOL is not given, the default is 1e-5.
0007 %   If Y is not given, the default is 0.  X and Y can be single numbers,
0008 %   vectors, or matrices.
0009 
0010 if nargin < 3
0011     tol = 1e-5;
0012 end
0013 
0014 if nargin < 2
0015     y = 0;
0016 end
0017 
0018 tf = all(abs(x(:) - y(:)) <= tol);
```

---

Generated on Thu 11-Aug-2011 15:06:22 by **m2html** © 2005
