# Supplementary material for: TIGER: Toolbox for integrating genome-scale metabolic models, expression data, and transcriptional regulatory networks
Source: BMC Syst Biol. 2011 Sep 23;5:147. doi: 10.1186/1752-0509-5-147 (PMC3224351; doi:10.1186/1752-0509-5-147)
Supplement: Additional file 2 — TIGER source code. Source code, documentation, and tutorials are also available online at http://bme.virginia.edu/csbl/downloads/ or http://csbl.bitbucket.org/tiger. [file 1752-0509-5-147-S2.GZ › tiger/doc/m2html/tiger/util/make_tiger_doc.html]

Description of make\_tiger\_doc


Home > tiger > util > make\_tiger\_doc.m

# make\_tiger\_doc

## PURPOSE

## SYNOPSIS

**This is a script file.**

## DESCRIPTION

## CROSS-REFERENCE INFORMATION

This function calls:


This function is called by:


## SOURCE CODE

```
0001 
0002 prev = cd;
0003 cd('~/work');
0004 
0005 m2html('mfiles','tiger','htmldir','tiger/doc/m2html', ...
0006        'recursive','on','global','on')
0007 
0008 cd('~/work/tiger/util');
0009 system(['perl add_categories.pl ../doc/m2html/index.html ' ...
0010         'categories.txt > ../doc/m2html/index2.html'])
0011 system('mv ../doc/m2html/index2.html ../doc/m2html/index.html')
0012    
0013 cd(prev);
```

---

Generated on Thu 11-Aug-2011 15:06:22 by **m2html** © 2005
