# Supplementary material for: TIGER: Toolbox for integrating genome-scale metabolic models, expression data, and transcriptional regulatory networks
Source: BMC Syst Biol. 2011 Sep 23;5:147. doi: 10.1186/1752-0509-5-147 (PMC3224351; doi:10.1186/1752-0509-5-147)
Supplement: Additional file 2 — TIGER source code. Source code, documentation, and tutorials are also available online at http://bme.virginia.edu/csbl/downloads/ or http://csbl.bitbucket.org/tiger. [file 1752-0509-5-147-S2.GZ › tiger/doc/m2html/tiger/index.html]

Index for Directory tiger


|  |  |
| --- | --- |
| Master index | Index for tiger |

# Index for tiger

## Matlab files in this directory:

|  |  |
| --- | --- |
| about\_tiger |  |
| add\_column | Add a column to a TIGER model structure |
| add\_diff | Add difference variables toa TIGER model |
| add\_growth\_constraint | Add minimum growth constraint to a model. |
| add\_row | Add a row to a TIGER model structure |
| add\_rule | Add rules to a TIGER model |
| assert\_tiger | Assert that a structure is an TIGER model. |
| bind\_mets | Bind metabolites to exchange reactions. |
| bind\_var | Bind variables to a indicator variable |
| check\_tiger | Check size and orientation of TIGER fields |
| close\_bounds | Return bounds that have been opened by OPEN\_BOUNDS |
| cobra\_to\_tiger | Convert a COBRA model to a TIGER model |
| convert\_gpr | Add the GPR rules as constraints to the model. |
| convert\_ids | Create name, indices, and logical indices from an array |
| create\_empty\_tiger | Create an empty TIGER model structure. |
| extract\_cobra | Extract the original COBRA model from a TIGER structure. |
| fba | Run Flux Balance Analysis on a TIGER model. |
| find\_associated\_rules | Find rules associated with an atom |
| find\_exchange\_rxns | Find locations of exchange reactions |
| find\_infeasible\_rules | Determine which rules make a model infeasible. |
| fva | Flux Variability Analysis |
| make\_milp | Convert a TIGER structure to a CMPI MILP. |
| minimal\_genome | Calculate a minimal genome |
| moma | Minimization of Metabolic Adjustment |
| open\_bounds | Open all bounds to a max value |
| remove\_column | Remove column(s) from a TIGER model |
| remove\_null\_rules | Remove NULL rules from a TIGER model |
| remove\_row | Remove row(s) from a TIGER model |
| remove\_rule | Remove rule(s) previously added to a TIGER model |
| scale\_bounds | Apply a scaling factor to upper and lower bounds |
| set\_fieldval | Set values in a TIGER structure field |
| set\_var | Set bounds on a variable |
| show\_exchange\_rxns | Show exchange reactions (and fluxes) |
| show\_sol | Show a solution vector |
| show\_tiger | Show a TIGER model as a MIP |
| single\_gene\_ko | Perform single gene knockout simulations |
| solve\_tiger | Solve a TIGER model. |
| update\_rule | Re-compile rule(s) previously added to a TIGER model |

## Subsequent directories:

- .hg
- @cmpi
- cobra
- doc
- elf
- parsing
- test
- tie
- util

---

Generated on Thu 11-Aug-2011 15:06:20 by **m2html** © 2005
