# Supplementary material for: TIGER: Toolbox for integrating genome-scale metabolic models, expression data, and transcriptional regulatory networks
Source: BMC Syst Biol. 2011 Sep 23;5:147. doi: 10.1186/1752-0509-5-147 (PMC3224351; doi:10.1186/1752-0509-5-147)
Supplement: Additional file 2 — TIGER source code. Source code, documentation, and tutorials are also available online at http://bme.virginia.edu/csbl/downloads/ or http://csbl.bitbucket.org/tiger. [file 1752-0509-5-147-S2.GZ › tiger/doc/m2html/tiger/assert_tiger.html]

Description of assert\_tiger


Home > tiger > assert\_tiger.m

# assert\_tiger

## PURPOSE

**Assert that a structure is an TIGER model.**

## SYNOPSIS

**function [tiger] = assert\_tiger(model)**

## DESCRIPTION

```
 ASSERT_TIGER  Assert that a structure is an TIGER model.

   [TIGER] = ASSERT_TIGER(MODEL)

   Checks that the structure MODEL is a TIGER model.  If not, converts
   MODEL to a TIGER model and warns that this procedure is not efficient
   for repeated calls to the parent function.
```

## CROSS-REFERENCE INFORMATION

This function calls:

- cobra\_to\_tiger Convert a COBRA model to a TIGER model
- create\_empty\_tiger Create an empty TIGER model structure.

This function is called by:

- add\_rule Add rules to a TIGER model
- convert\_gpr Add the GPR rules as constraints to the model.
- gimme Gene Inactivity Moderated by Metabolism and Expression
- made Metabolic Adjustment by Differential Expression

## SOURCE CODE

```
0001 function [tiger] = assert_tiger(model)
0002 % ASSERT_TIGER  Assert that a structure is an TIGER model.
0003 %
0004 %   [TIGER] = ASSERT_TIGER(MODEL)
0005 %
0006 %   Checks that the structure MODEL is a TIGER model.  If not, converts
0007 %   MODEL to a TIGER model and warns that this procedure is not efficient
0008 %   for repeated calls to the parent function.
0009 
0010 fields = {'A','b','vartypes','ctypes','rownames','varnames','obj'};
0011 
0012 if ~all(isfield(model,fields))
0013     % convert model
0014     fprintf('This model is not a TIGER model.  It will automatically\n');
0015     fprintf('be converted.  For repeated calls to this function, it\n');
0016     fprintf('is more efficient to convert beforehand.\n');
0017     
0018     if isempty(model)
0019         tiger = create_empty_tiger();
0020     else
0021         tiger = cobra_to_tiger(model);
0022     end
0023 else
0024     tiger = model;
0025 end
0026
```

---

Generated on Thu 11-Aug-2011 15:06:22 by **m2html** © 2005
