# Supplementary material for: TIGER: Toolbox for integrating genome-scale metabolic models, expression data, and transcriptional regulatory networks
Source: BMC Syst Biol. 2011 Sep 23;5:147. doi: 10.1186/1752-0509-5-147 (PMC3224351; doi:10.1186/1752-0509-5-147)
Supplement: Additional file 2 — TIGER source code. Source code, documentation, and tutorials are also available online at http://bme.virginia.edu/csbl/downloads/ or http://csbl.bitbucket.org/tiger. [file 1752-0509-5-147-S2.GZ › tiger/doc/m2html/tiger/cobra/make_c_matrix.html]

Description of make\_c\_matrix


Home > tiger > cobra > make\_c\_matrix.m

# make\_c\_matrix

## PURPOSE

**Make reaction/gene correlation (C) matrix**

## SYNOPSIS

**function [C,model] = make\_c\_matrix(model,varargin)**

## DESCRIPTION

```
 MAKE_C_MATRIX  Make reaction/gene correlation (C) matrix

   [C,MODEL] = MAKE_C_MATRIX(MODEL,...params...)

   Creates C, a |rxns| x |genes| matrix containing a correlation 
   coefficient between a reaction and the genes in the corresponding GPR.
   MODEL is returned with C additional fields used in the calculation
   (rxnGeneMat and rules).

   Parameters
   'normalize'  If true, the entries for each reaction are normalized to
                sum to one.
   'cutoff'     If a GPR contains more than 'cutoff' genes, the
                coefficients are estimated by Monte Carlo sampling.
                (Default = 10)
   'samples'    If Monte Carlo sampling is used, 'samples' number of 
                draws are taken.  (Default = 1000)
   'verbose'    If true (default = false), a status bar is displayed.
```

## CROSS-REFERENCE INFORMATION

This function calls:

- convert\_grRules Parse grRules into rules for the COBRA toolbox
- make\_rxnGeneMat Build a rxnGeneMat for cobra models
- int2bin Convert an integer to an array of binary values
- statusbar

This function is called by:

- map\_genes\_to\_rxns Map measurements from gene to reactions

## SOURCE CODE

```
0001 function [C,model] = make_c_matrix(model,varargin)
0002 % MAKE_C_MATRIX  Make reaction/gene correlation (C) matrix
0003 %
0004 %   [C,MODEL] = MAKE_C_MATRIX(MODEL,...params...)
0005 %
0006 %   Creates C, a |rxns| x |genes| matrix containing a correlation
0007 %   coefficient between a reaction and the genes in the corresponding GPR.
0008 %   MODEL is returned with C additional fields used in the calculation
0009 %   (rxnGeneMat and rules).
0010 %
0011 %   Parameters
0012 %   'normalize'  If true, the entries for each reaction are normalized to
0013 %                sum to one.
0014 %   'cutoff'     If a GPR contains more than 'cutoff' genes, the
0015 %                coefficients are estimated by Monte Carlo sampling.
0016 %                (Default = 10)
0017 %   'samples'    If Monte Carlo sampling is used, 'samples' number of
0018 %                draws are taken.  (Default = 1000)
0019 %   'verbose'    If true (default = false), a status bar is displayed.
0020 
0021 p = inputParser();
0022 p.addParamValue('normalize',true);
0023 p.addParamValue('cutoff',10);
0024 p.addParamValue('samples',1000);
0025 p.addParamValue('verbose',false);
0026 
0027 p.parse(varargin{:});
0028 
0029 normalize = p.Results.normalize;
0030 cutoff = p.Results.cutoff;
0031 n_samples = p.Results.samples;
0032 verbose = p.Results.verbose;
0033 
0034 % check for correct COBRA fields
0035 if ~isfield(model,'rxnGeneMat')
0036     model.rxnGeneMat = make_rxnGeneMat(model);
0037 end
0038 
0039 if ~isfield(model,'rules')
0040     model.rules = convert_grRules(model);
0041 end
0042 
0043 RGM = model.rxnGeneMat;
0044 [nrxns,ngenes] = size(RGM);
0045 rules = model.rules;
0046 
0047 % number of genes in each reaction
0048 N = sum(RGM,2);
0049 
0050 C = zeros(nrxns,ngenes);
0051 
0052 x = zeros(ngenes,1);
0053 statbar = statusbar(nrxns,verbose);
0054 statbar.start('C Matrix calculation');
0055 for r = 1 : nrxns
0056     statbar.update(r);
0057     
0058     if isempty(rules{r})
0059         continue;
0060     end
0061     
0062     idxs = find(RGM(r,:));
0063     
0064     if N(r) <= cutoff
0065         % compute coefficients manually
0066         s11_00 = zeros(1,N(r));
0067         s01_10 = zeros(1,N(r));
0068         
0069         for i = 0 : 2^N(r) - 1
0070             state = int2bin(i,N(r));
0071             x(idxs) = state;
0072             onoff = eval(rules{r});
0073             
0074             s11_00 = s11_00 + (state == onoff);
0075             s01_10 = s01_10 + (state ~= onoff);
0076         end
0077         x(idxs) = 0;
0078         
0079         C(r,idxs) = (s11_00 - s01_10) / 2^N(r);
0080     else
0081         % compute coefficients using Monte Carlo
0082         data = zeros(n_samples,N(r)+1);
0083         data(:,1:N(r)) = randi(2,n_samples,N(r)) - 1;
0084         for i = 1 : n_samples
0085             x(idxs) = data(i,1:N(r));
0086             data(i,end) = eval(rules{r});
0087         end
0088         x(idxs) = 0;
0089         
0090         corrm = corr(data);
0091         C(r,idxs) = corrm(end,1:end-1);
0092     end
0093 end
0094 
0095 if normalize
0096     for i = 1 : nrxns
0097         if all(C(i,:) == 0)
0098             continue;
0099         end
0100         
0101         Cmin = min(C(i,:));
0102         Cmax = max(C(i,:));
0103         C(i,:) = (C(i,:) - Cmin) / (Cmax - Cmin);
0104     end
0105 end
0106 
0107 if nargout > 1
0108     model.C = C;
0109 end
0110 
0111 
0112
```

---

Generated on Thu 11-Aug-2011 15:06:22 by **m2html** © 2005
