# Supplementary material for: TIGER: Toolbox for integrating genome-scale metabolic models, expression data, and transcriptional regulatory networks
Source: BMC Syst Biol. 2011 Sep 23;5:147. doi: 10.1186/1752-0509-5-147 (PMC3224351; doi:10.1186/1752-0509-5-147)
Supplement: Additional file 2 — TIGER source code. Source code, documentation, and tutorials are also available online at http://bme.virginia.edu/csbl/downloads/ or http://csbl.bitbucket.org/tiger. [file 1752-0509-5-147-S2.GZ › tiger/doc/m2html/tiger/cobra/index.html]

Index for Directory tiger/cobra


|  |  |
| --- | --- |
| Master index | Index for tiger/cobra |

# Index for tiger/cobra

## Matlab files in this directory:

|  |  |
| --- | --- |
| average\_by\_subsystem | Average gene or flux data by subsystem |
| convert\_grRules | Parse grRules into rules for the COBRA toolbox |
| decompose\_gpr | Show summary statistics on the GPR of a COBRA model |
| make\_c\_matrix | Make reaction/gene correlation (C) matrix |
| make\_rxnGeneMat | Build a rxnGeneMat for cobra models |
| map\_genes\_to\_rxns | Map measurements from gene to reactions |

---

Generated on Thu 11-Aug-2011 15:06:20 by **m2html** © 2005
