# Supplementary material for: TIGER: Toolbox for integrating genome-scale metabolic models, expression data, and transcriptional regulatory networks
Source: BMC Syst Biol. 2011 Sep 23;5:147. doi: 10.1186/1752-0509-5-147 (PMC3224351; doi:10.1186/1752-0509-5-147)
Supplement: Additional file 2 — TIGER source code. Source code, documentation, and tutorials are also available online at http://bme.virginia.edu/csbl/downloads/ or http://csbl.bitbucket.org/tiger. [file 1752-0509-5-147-S2.GZ › tiger/doc/m2html/tiger/cobra/convert_grRules.html]

Description of convert\_grRules


Home > tiger > cobra > convert\_grRules.m

# convert\_grRules

## PURPOSE

**Parse grRules into rules for the COBRA toolbox**

## SYNOPSIS

**function [rules] = convert\_grRules(cobra)**

## DESCRIPTION

```
 CONVERT_GRRULES  Parse grRules into rules for the COBRA toolbox

   [RULES] = CONVERT_GRRULES(COBRA)

   Parses grRules (human-readable) into the COBRA rules format
   (i.e., x(1) | x(4)).  Returns a cell array of the rule strings.
```

## CROSS-REFERENCE INFORMATION

This function calls:

- parse\_string Parse a rule string into an EXPR object
- map Generate a new list by applying a function

This function is called by:

- make\_c\_matrix Make reaction/gene correlation (C) matrix
- cobra\_model Test model in COBRA format

## SUBFUNCTIONS

- function [rule] = convert\_aux(str)
- function switch\_atom(e)

## SOURCE CODE

```
0001 function [rules] = convert_grRules(cobra)
0002 % CONVERT_GRRULES  Parse grRules into rules for the COBRA toolbox
0003 %
0004 %   [RULES] = CONVERT_GRRULES(COBRA)
0005 %
0006 %   Parses grRules (human-readable) into the COBRA rules format
0007 %   (i.e., x(1) | x(4)).  Returns a cell array of the rule strings.
0008 
0009 rules = map(@convert_aux,cobra.grRules);
0010 
0011 function [rule] = convert_aux(str)
0012     e = parse_string(str);
0013     e.iterif(@(x) x.is_atom,@(x) switch_atom(x));
0014     rule = e.to_string();
0015 end
0016 
0017 function switch_atom(e)
0018     [~,loc] = ismember(e.id,cobra.genes);
0019     e.id = sprintf('x(%i)',loc);
0020 end
0021 
0022 end
```

---

Generated on Thu 11-Aug-2011 15:06:22 by **m2html** © 2005
