# Supplementary material for: TIGER: Toolbox for integrating genome-scale metabolic models, expression data, and transcriptional regulatory networks
Source: BMC Syst Biol. 2011 Sep 23;5:147. doi: 10.1186/1752-0509-5-147 (PMC3224351; doi:10.1186/1752-0509-5-147)
Supplement: Additional file 2 — TIGER source code. Source code, documentation, and tutorials are also available online at http://bme.virginia.edu/csbl/downloads/ or http://csbl.bitbucket.org/tiger. [file 1752-0509-5-147-S2.GZ › tiger/doc/m2html/tiger/cobra/average_by_subsystem.html]

Description of average\_by\_subsystem


Home > tiger > cobra > average\_by\_subsystem.m

# average\_by\_subsystem

## PURPOSE

**Average gene or flux data by subsystem**

## SYNOPSIS

**function [vals,subsystems] = average\_by\_subsystem(model,data)**

## DESCRIPTION

```
 AVERAGE_BY_SUBSYSTEM  Average gene or flux data by subsystem

   [VALS,SUBSYSTEMS] = AVERAGE_BY_SUBSYSTEMS(MODEL,DATA)

   Average gene or flux data by grouping values by subsystem.  SUBSYSTEMS
   is a unique cell of subsystems corresponding to the average values in
   VALS.  The format of each entry in SUBSYSTEMS is "s [i,j]", where s is
   the name of the subsystem, and i and j are the number of reactions and
   genes mapped to that subsystem.

   MODEL must contain a "subSystems" field of subsystems for each 
   reaction.  If LENGTH(DATA) == LENGTH(MODEL.GENES), the values in DATA
   are mapped to reaction susing MAP_GENES_TO_RXNS.
```

## CROSS-REFERENCE INFORMATION

This function calls:

- make\_rxnGeneMat Build a rxnGeneMat for cobra models
- map\_genes\_to\_rxns Map measurements from gene to reactions
- count Count the number of nonzero elements in a vector

This function is called by:


## SOURCE CODE

```
0001 function [vals,subsystems] = average_by_subsystem(model,data)
0002 % AVERAGE_BY_SUBSYSTEM  Average gene or flux data by subsystem
0003 %
0004 %   [VALS,SUBSYSTEMS] = AVERAGE_BY_SUBSYSTEMS(MODEL,DATA)
0005 %
0006 %   Average gene or flux data by grouping values by subsystem.  SUBSYSTEMS
0007 %   is a unique cell of subsystems corresponding to the average values in
0008 %   VALS.  The format of each entry in SUBSYSTEMS is "s [i,j]", where s is
0009 %   the name of the subsystem, and i and j are the number of reactions and
0010 %   genes mapped to that subsystem.
0011 %
0012 %   MODEL must contain a "subSystems" field of subsystems for each
0013 %   reaction.  If LENGTH(DATA) == LENGTH(MODEL.GENES), the values in DATA
0014 %   are mapped to reaction susing MAP_GENES_TO_RXNS.
0015 
0016 data(~isfinite(data)) = 0;
0017 
0018 if ~isfield(model,'rxnGeneMat')
0019     model.rxnGeneMat = make_rxnGeneMat(model);
0020 end
0021 
0022 assert(isfield(model,'subSystems'), ...
0023        'model does not contain a "subSystems" field');
0024 
0025 if length(data) == length(model.genes)
0026     data = map_genes_to_rxns(model,data);
0027 end
0028 
0029 subsystems = unique(model.subSystems);
0030 [~,subids] = ismember(model.subSystems,subsystems);
0031 
0032 vals = zeros(length(subsystems),1);
0033 for i = 1 : length(subsystems)
0034     insub = ismember(subids,i);
0035     nrxns = count(insub > 0);
0036     ngenes = count( sum(model.rxnGeneMat(insub,:),1) > 0 );
0037     
0038     vals(i) = mean(data(insub));
0039     subsystems{i} = sprintf('%s [%i,%i]',subsystems{i},nrxns,ngenes);
0040 end
0041
```

---

Generated on Thu 11-Aug-2011 15:06:22 by **m2html** © 2005
