# Supplementary material for: TIGER: Toolbox for integrating genome-scale metabolic models, expression data, and transcriptional regulatory networks
Source: BMC Syst Biol. 2011 Sep 23;5:147. doi: 10.1186/1752-0509-5-147 (PMC3224351; doi:10.1186/1752-0509-5-147)
Supplement: Additional file 2 — TIGER source code. Source code, documentation, and tutorials are also available online at http://bme.virginia.edu/csbl/downloads/ or http://csbl.bitbucket.org/tiger. [file 1752-0509-5-147-S2.GZ › tiger/doc/m2html/tiger/cobra/map_genes_to_rxns.html]

Description of map\_genes\_to\_rxns


Home > tiger > cobra > map\_genes\_to\_rxns.m

# map\_genes\_to\_rxns

## PURPOSE

**Map measurements from gene to reactions**

## SYNOPSIS

**function [rxn\_vals,model] = map\_genes\_to\_rxns(model,gene\_vals)**

## DESCRIPTION

```
 MAP_GENES_TO_RXNS  Map measurements from gene to reactions

   [RXN_VALS,MODEL] = MAP_GENES_TO_RXNS(MODEL,GENE_VALS)

   Maps GENE_VALS to RXN_VALS (genes to reactions) using weighting from
   the GPR in MODEL.  The weighting is calculated using the C matrix,
   which is added to the MODEL if it does not already exist.
```

## CROSS-REFERENCE INFORMATION

This function calls:

- make\_c\_matrix Make reaction/gene correlation (C) matrix

This function is called by:

- average\_by\_subsystem Average gene or flux data by subsystem

## SOURCE CODE

```
0001 function [rxn_vals,model] = map_genes_to_rxns(model,gene_vals)
0002 % MAP_GENES_TO_RXNS  Map measurements from gene to reactions
0003 %
0004 %   [RXN_VALS,MODEL] = MAP_GENES_TO_RXNS(MODEL,GENE_VALS)
0005 %
0006 %   Maps GENE_VALS to RXN_VALS (genes to reactions) using weighting from
0007 %   the GPR in MODEL.  The weighting is calculated using the C matrix,
0008 %   which is added to the MODEL if it does not already exist.
0009 
0010 if ~isfield(model,'C')
0011     model.C = make_c_matrix(model);
0012 end
0013 
0014 rxn_vals = model.C * gene_vals;
```

---

Generated on Thu 11-Aug-2011 15:06:22 by **m2html** © 2005
