# Supplementary material for: TIGER: Toolbox for integrating genome-scale metabolic models, expression data, and transcriptional regulatory networks
Source: BMC Syst Biol. 2011 Sep 23;5:147. doi: 10.1186/1752-0509-5-147 (PMC3224351; doi:10.1186/1752-0509-5-147)
Supplement: Additional file 2 — TIGER source code. Source code, documentation, and tutorials are also available online at http://bme.virginia.edu/csbl/downloads/ or http://csbl.bitbucket.org/tiger. [file 1752-0509-5-147-S2.GZ › tiger/doc/m2html/tiger/show_tiger.html]

Description of show\_tiger


Home > tiger > show\_tiger.m

# show\_tiger

## PURPOSE

**Show a TIGER model as a MIP**

## SYNOPSIS

**function show\_tiger(tiger,varargin)**

## DESCRIPTION

```
 SHOW_TIGER  Show a TIGER model as a MIP

   SHOW_TIGER(TIGER,...params...)

   Displays a TIGER model in equation form.  The following parameters can
   be given:
       'bounds'  Show the bounds and type for each variable.
       'rxns'    Treat continuous variables as "reactions" and display 
                 the chemical reaction.
```

## CROSS-REFERENCE INFORMATION

This function calls:

- make\_milp Convert a TIGER structure to a CMPI MILP.

This function is called by:


## SUBFUNCTIONS

- function show\_reaction(idx)
- function print\_moieties(idxs)

## SOURCE CODE

```
0001 function show_tiger(tiger,varargin)
0002 % SHOW_TIGER  Show a TIGER model as a MIP
0003 %
0004 %   SHOW_TIGER(TIGER,...params...)
0005 %
0006 %   Displays a TIGER model in equation form.  The following parameters can
0007 %   be given:
0008 %       'bounds'  Show the bounds and type for each variable.
0009 %       'rxns'    Treat continuous variables as "reactions" and display
0010 %                 the chemical reaction.
0011 
0012 showvars = ismember('bounds',varargin);
0013 showrxns = ismember('rxns',varargin);
0014 
0015 cmpi.show_mip(make_milp(tiger),'showvars',showvars);
0016 
0017 if showrxns
0018     [m,n] = size(tiger.A);
0019     % find metabolite rows
0020     is_met = false(m,1);
0021     for i = 1 : m
0022         is_met(i) = all(tiger.vartypes(tiger.A(i,:) ~= 0) == 'c');
0023     end
0024     
0025     fprintf('\n\n----- Reactions -----\n');
0026     for i = 1 : n
0027         if tiger.vartypes(i) == 'c'
0028             show_reaction(i);
0029         end
0030     end
0031 end
0032 
0033 function show_reaction(idx)
0034     reacts = find(is_met & tiger.A(:,idx) < 0);
0035     prods  = find(is_met & tiger.A(:,idx) > 0);
0036     
0037     fprintf('%s : ',tiger.varnames{idx});
0038     print_moieties(reacts);
0039     if (tiger.lb(idx) < 0) || (isfield(tiger,'rev') && tiger.rev(idx))
0040         fprintf(' <-> ');
0041     else
0042         fprintf(' -> ');
0043     end
0044     print_moieties(prods);
0045     fprintf('\n');
0046 
0047     function print_moieties(idxs)
0048         for j = 1 : length(idxs)
0049             if abs(tiger.A(idxs(j),idx)) == 1
0050                 fprintf(tiger.rownames{idxs(j)});
0051             else
0052                 fprintf('%f %s',num2str(abs(tiger.A(idxs(j),idx))), ...
0053                                 tiger.rownames{idxs(j)});
0054             end
0055             
0056             if j < length(idxs)
0057                 fprintf(' + ');
0058             end
0059         end
0060     end
0061 end
0062 
0063 end
0064     
0065
```

---

Generated on Thu 11-Aug-2011 15:06:22 by **m2html** © 2005
