# Supplementary material for: TIGER: Toolbox for integrating genome-scale metabolic models, expression data, and transcriptional regulatory networks
Source: BMC Syst Biol. 2011 Sep 23;5:147. doi: 10.1186/1752-0509-5-147 (PMC3224351; doi:10.1186/1752-0509-5-147)
Supplement: Additional file 2 — TIGER source code. Source code, documentation, and tutorials are also available online at http://bme.virginia.edu/csbl/downloads/ or http://csbl.bitbucket.org/tiger. [file 1752-0509-5-147-S2.GZ › tiger/doc/m2html/tiger/convert_gpr.html]

Description of convert\_gpr


Home > tiger > convert\_gpr.m

# convert\_gpr

## PURPOSE

**Add the GPR rules as constraints to the model.**

## SYNOPSIS

**function [tiger] = convert\_gpr(tiger,varargin)**

## DESCRIPTION

```
 CONVERT_GPR  Add the GPR rules as constraints to the model.

   [TIGER] = CONVERT_GPR(TIGER,...ADD_RULE params...)

   Each GPR expression is converted to a rule and added to the model.
   Extra parameters will be passed to the ADD_RULE function.
```

## CROSS-REFERENCE INFORMATION

This function calls:

- add\_rule Add rules to a TIGER model
- assert\_tiger Assert that a structure is an TIGER model.
- bind\_var Bind variables to a indicator variable
- cellzip Zip two cell arrays by a function
- map Generate a new list by applying a function

This function is called by:

- cobra\_to\_tiger Convert a COBRA model to a TIGER model

## SOURCE CODE

```
0001 function [tiger] = convert_gpr(tiger,varargin)
0002 % CONVERT_GPR  Add the GPR rules as constraints to the model.
0003 %
0004 %   [TIGER] = CONVERT_GPR(TIGER,...ADD_RULE params...)
0005 %
0006 %   Each GPR expression is converted to a rule and added to the model.
0007 %   Extra parameters will be passed to the ADD_RULE function.
0008 
0009 tiger = assert_tiger(tiger);
0010 
0011 RXN_PRE = 'RXN__';
0012 
0013 Ngenes = length(tiger.genes);
0014 [m,n] = size(tiger.A);
0015 
0016 rxns  = find(~cellfun(@isempty,tiger.gpr));
0017 Nrxns = length(rxns);
0018 
0019 rxn_names = map(@(x) [RXN_PRE x],tiger.varnames(rxns));
0020 gpr_rules = cellzip(@(x,y) [x ' <=> "' y '"'],tiger.gpr(rxns),rxn_names);
0021 
0022 %tiger.obj = [tiger.obj; zeros(Nrxns+Ngenes,1)];
0023 %tiger.A = [tiger.A sparse(m,Nrxns+Ngenes)];
0024 %tiger.varnames = [tiger.varnames; tiger.genes; rxn_names];
0025 %tiger.vartypes = [tiger.vartypes; repmat('b',Nrxns+Ngenes,1)];
0026 %tiger.lb = [tiger.lb; zeros(Nrxns+Ngenes,1)];
0027 %tiger.ub = [tiger.ub;  ones(Nrxns+Ngenes,1)];
0028 
0029 tiger = add_rule(tiger,gpr_rules,varargin{:});
0030 tiger = bind_var(tiger,tiger.varnames(rxns),rxn_names);
```

---

Generated on Thu 11-Aug-2011 15:06:22 by **m2html** © 2005
