# Supplementary material for: TIGER: Toolbox for integrating genome-scale metabolic models, expression data, and transcriptional regulatory networks
Source: BMC Syst Biol. 2011 Sep 23;5:147. doi: 10.1186/1752-0509-5-147 (PMC3224351; doi:10.1186/1752-0509-5-147)
Supplement: Additional file 2 — TIGER source code. Source code, documentation, and tutorials are also available online at http://bme.virginia.edu/csbl/downloads/ or http://csbl.bitbucket.org/tiger. [file 1752-0509-5-147-S2.GZ › tiger/doc/m2html/tiger/scale_bounds.html]

Description of scale\_bounds


Home > tiger > scale\_bounds.m

# scale\_bounds

## PURPOSE

**Apply a scaling factor to upper and lower bounds**

## SYNOPSIS

**function [tiger] = scale\_bounds(tiger,scaling)**

## DESCRIPTION

```
 SCALE_BOUNDS  Apply a scaling factor to upper and lower bounds

   [TIGER] = SCALE_BOUNDS(TIGER,SCALING)

   Scales TIGER.lb and TIGER.ub by SCALING and returns the modified
   model.  The scaling can be useful for avoiding numerical stabilities
   from Big-M constraints.
```

## CROSS-REFERENCE INFORMATION

This function calls:


This function is called by:


## SOURCE CODE

```
0001 function [tiger] = scale_bounds(tiger,scaling)
0002 % SCALE_BOUNDS  Apply a scaling factor to upper and lower bounds
0003 %
0004 %   [TIGER] = SCALE_BOUNDS(TIGER,SCALING)
0005 %
0006 %   Scales TIGER.lb and TIGER.ub by SCALING and returns the modified
0007 %   model.  The scaling can be useful for avoiding numerical stabilities
0008 %   from Big-M constraints.
0009 
0010 tiger.lb = scaling * tiger.lb;
0011 tiger.ub = scaling * tiger.ub;
```

---

Generated on Thu 11-Aug-2011 15:06:22 by **m2html** © 2005
