# Supplementary material for: TIGER: Toolbox for integrating genome-scale metabolic models, expression data, and transcriptional regulatory networks
Source: BMC Syst Biol. 2011 Sep 23;5:147. doi: 10.1186/1752-0509-5-147 (PMC3224351; doi:10.1186/1752-0509-5-147)
Supplement: Additional file 2 — TIGER source code. Source code, documentation, and tutorials are also available online at http://bme.virginia.edu/csbl/downloads/ or http://csbl.bitbucket.org/tiger. [file 1752-0509-5-147-S2.GZ › tiger/doc/m2html/tiger/remove_row.html]

Description of remove\_row


Home > tiger > remove\_row.m

# remove\_row

## PURPOSE

**Remove row(s) from a TIGER model**

## SYNOPSIS

**function [tiger] = remove\_row(tiger,row\_ids)**

## DESCRIPTION

```
 REMOVE_ROW  Remove row(s) from a TIGER model

   [TIGER] = REMOVE_ROW(TIGER,ROW_IDS)

   Remove rows ROW_IDS from a TIGER model and return the modified
   structure.  ROW_IDS are any valid IDs (see CONVERT_IDS).
```

## CROSS-REFERENCE INFORMATION

This function calls:

- convert\_ids Create name, indices, and logical indices from an array

This function is called by:

- remove\_null\_rules Remove NULL rules from a TIGER model

## SOURCE CODE

```
0001 function [tiger] = remove_row(tiger,row_ids)
0002 % REMOVE_ROW  Remove row(s) from a TIGER model
0003 %
0004 %   [TIGER] = REMOVE_ROW(TIGER,ROW_IDS)
0005 %
0006 %   Remove rows ROW_IDS from a TIGER model and return the modified
0007 %   structure.  ROW_IDS are any valid IDs (see CONVERT_IDS).
0008 
0009 ids = ~convert_ids(tiger.rownames,row_ids,'logical');
0010 
0011 tiger.A = tiger.A(ids,:);
0012 tiger.b = tiger.b(ids);
0013 
0014 tiger.rownames = tiger.rownames(ids);
0015 tiger.ctypes = tiger.ctypes(ids);
0016 
0017 tiger.ind = tiger.ind(ids);
0018 tiger.indtypes = tiger.indtypes(ids);
0019 
0020 tiger.param.rule_id = tiger.param.rule_id(ids);
0021
```

---

Generated on Thu 11-Aug-2011 15:06:22 by **m2html** © 2005
