# Supplementary material for: TIGER: Toolbox for integrating genome-scale metabolic models, expression data, and transcriptional regulatory networks
Source: BMC Syst Biol. 2011 Sep 23;5:147. doi: 10.1186/1752-0509-5-147 (PMC3224351; doi:10.1186/1752-0509-5-147)
Supplement: Additional file 2 — TIGER source code. Source code, documentation, and tutorials are also available online at http://bme.virginia.edu/csbl/downloads/ or http://csbl.bitbucket.org/tiger. [file 1752-0509-5-147-S2.GZ › tiger/doc/m2html/tiger/show_sol.html]

Description of show\_sol


Home > tiger > show\_sol.m

# show\_sol

## PURPOSE

**Show a solution vector**

## SYNOPSIS

**function show\_sol(tiger,sol,show\_cond)**

## DESCRIPTION

```
 SHOW_SOL  Show a solution vector

   SHOW_SOL(TIGER,SOL,SHOW_COND)
   SHOW_SOL(TIGER,X,SHOW_COND)

   Shows the values for each nonzero variable in the solution structure
   SOL.  The optional string SHOW_COND contains a test on the variable
   'x' to identify which fluxes should be shown.  The default is
   'x ~= 0'.  If SHOW_COND = 'all', all variables are shown.

   A vector of variable values X may be provided instead of the solution
   structure SOL.
```

## CROSS-REFERENCE INFORMATION

This function calls:


This function is called by:


## SOURCE CODE

```
0001 function show_sol(tiger,sol,show_cond)
0002 % SHOW_SOL  Show a solution vector
0003 %
0004 %   SHOW_SOL(TIGER,SOL,SHOW_COND)
0005 %   SHOW_SOL(TIGER,X,SHOW_COND)
0006 %
0007 %   Shows the values for each nonzero variable in the solution structure
0008 %   SOL.  The optional string SHOW_COND contains a test on the variable
0009 %   'x' to identify which fluxes should be shown.  The default is
0010 %   'x ~= 0'.  If SHOW_COND = 'all', all variables are shown.
0011 %
0012 %   A vector of variable values X may be provided instead of the solution
0013 %   structure SOL.
0014 
0015 if nargin < 3
0016     show_cond = 'x ~= 0';
0017 end
0018 
0019 if isa(sol,'struct')
0020     x = sol.x;
0021 else
0022     x = sol;
0023 end
0024 
0025 if isempty(x)
0026     fprintf('No solution -- sol.x is empty.\n');
0027     return;
0028 end
0029 
0030 if strcmpi(show_cond,'all')
0031     to_show = 1:length(x);
0032 else
0033     to_show = find(eval(show_cond));
0034 end
0035     
0036 x(tiger.vartypes ~= 'c') = round(x(tiger.vartypes ~= 'c'));
0037 
0038 fprintf('\n');
0039 
0040 if isa(sol,'struct')
0041     fprintf(' Objective value:  %08f\n',sol.val);
0042     fprintf('   Solution flag:  %i\n',sol.flag);
0043 end
0044 
0045 fprintf('Optimal solution:\n');
0046 for i = 1 : length(to_show)
0047     if tiger.vartypes(to_show(i)) == 'c'
0048         fmt = '%10s:  %+08f\n';
0049     else
0050         fmt = '%10s:  %i\n';
0051     end
0052     fprintf(fmt,tiger.varnames{to_show(i)},x(to_show(i)));    
0053 end
0054 fprintf('\n');
0055
```

---

Generated on Thu 11-Aug-2011 15:06:22 by **m2html** © 2005
