# Supplementary material for: TIGER: Toolbox for integrating genome-scale metabolic models, expression data, and transcriptional regulatory networks
Source: BMC Syst Biol. 2011 Sep 23;5:147. doi: 10.1186/1752-0509-5-147 (PMC3224351; doi:10.1186/1752-0509-5-147)
Supplement: Additional file 2 — TIGER source code. Source code, documentation, and tutorials are also available online at http://bme.virginia.edu/csbl/downloads/ or http://csbl.bitbucket.org/tiger. [file 1752-0509-5-147-S2.GZ › tiger/doc/m2html/tiger/solve_tiger.html]

Description of solve\_tiger


Home > tiger > solve\_tiger.m

# solve\_tiger

## PURPOSE

**Solve a TIGER model.**

## SYNOPSIS

**function [sol] = solve\_tiger(tiger,sense)**

## DESCRIPTION

```
 SOLVE_TIGER  Solve a TIGER model.

   [SOL] = SOLVE_TIGER(TIGER,SENSE)

   Solve a TIGER model structure and return a CMPI solution structure.  
   SENSE can be either 'min' for minimization (default) or 'max' for 
   maximization.
```

## CROSS-REFERENCE INFORMATION

This function calls:


This function is called by:

- test\_\_remove\_rule
- run\_trn\_comparison

## SOURCE CODE

```
0001 function [sol] = solve_tiger(tiger,sense)
0002 % SOLVE_TIGER  Solve a TIGER model.
0003 %
0004 %   [SOL] = SOLVE_TIGER(TIGER,SENSE)
0005 %
0006 %   Solve a TIGER model structure and return a CMPI solution structure.
0007 %   SENSE can be either 'min' for minimization (default) or 'max' for
0008 %   maximization.
0009 
0010 if nargin < 2 && ~isfield(tiger,'sense')
0011     sense = 'min';
0012 end
0013 
0014 if nargin > 1 && ~isempty(sense)
0015     switch sense
0016         case {'min','minimize'}
0017             tiger.sense = 1;
0018         case {'max','maximize'}
0019             tiger.sense = -1;
0020         otherwise
0021             error('invalid sense: %s',sense);
0022     end
0023 end
0024 
0025 sol = cmpi.solve_mip(tiger);
```

---

Generated on Thu 11-Aug-2011 15:06:22 by **m2html** © 2005
