# Supplementary material for: TIGER: Toolbox for integrating genome-scale metabolic models, expression data, and transcriptional regulatory networks
Source: BMC Syst Biol. 2011 Sep 23;5:147. doi: 10.1186/1752-0509-5-147 (PMC3224351; doi:10.1186/1752-0509-5-147)
Supplement: Additional file 2 — TIGER source code. Source code, documentation, and tutorials are also available online at http://bme.virginia.edu/csbl/downloads/ or http://csbl.bitbucket.org/tiger. [file 1752-0509-5-147-S2.GZ › tiger/doc/m2html/tiger/bind_var.html]

Description of bind\_var


Home > tiger > bind\_var.m

# bind\_var

## PURPOSE

**Bind variables to a indicator variable**

## SYNOPSIS

**function [tiger] = bind\_var(tiger,vars,inds,varargin)**

## DESCRIPTION

```
 BIND_VAR  Bind variables to a indicator variable

   [TIGER] = BIND_VAR(TIGER,VARS,INDS,...params...)

   For each variable v in VARS and corresponding indicator I in INDS,
   adds constraints such that v=0 if I=0.

   If parameter 'iff' is true, the binding is such that v=0 if and only
   if I=0.  ('iff' is false by default.)

   The bounds used when adding these rules are determined by the
   parameters:
       (default)  LB = min(TIGER.lb), i.e. the lowest lower bound in the
                  entire model.  UB = max(TIGER.ub), the largest upper
                  bound in the model.
       'tight'    If true, the upper and lower bounds for v are used.
                  This may be more numerically stable, but can add
                  complications if the variable bounds are changed later,
                  as these changes will not be reflected in the binding
                  constraints.
       'lb','ub'  Number specifying LB and UB; these are used for every
                  variable.
```

## CROSS-REFERENCE INFORMATION

This function calls:

- add\_row Add a row to a TIGER model structure
- add\_rule Add rules to a TIGER model
- convert\_ids Create name, indices, and logical indices from an array
- cellzip Zip two cell arrays by a function

This function is called by:

- bind\_mets Bind metabolites to exchange reactions.
- convert\_gpr Add the GPR rules as constraints to the model.
- cobra\_to\_elf Create an ELF model from a COBRA structure
- convert\_rules

## SOURCE CODE

```
0001 function [tiger] = bind_var(tiger,vars,inds,varargin)
0002 % BIND_VAR  Bind variables to a indicator variable
0003 %
0004 %   [TIGER] = BIND_VAR(TIGER,VARS,INDS,...params...)
0005 %
0006 %   For each variable v in VARS and corresponding indicator I in INDS,
0007 %   adds constraints such that v=0 if I=0.
0008 %
0009 %   If parameter 'iff' is true, the binding is such that v=0 if and only
0010 %   if I=0.  ('iff' is false by default.)
0011 %
0012 %   The bounds used when adding these rules are determined by the
0013 %   parameters:
0014 %       (default)  LB = min(TIGER.lb), i.e. the lowest lower bound in the
0015 %                  entire model.  UB = max(TIGER.ub), the largest upper
0016 %                  bound in the model.
0017 %       'tight'    If true, the upper and lower bounds for v are used.
0018 %                  This may be more numerically stable, but can add
0019 %                  complications if the variable bounds are changed later,
0020 %                  as these changes will not be reflected in the binding
0021 %                  constraints.
0022 %       'lb','ub'  Number specifying LB and UB; these are used for every
0023 %                  variable.
0024 
0025 p = inputParser;
0026 p.addParamValue('iff',false);
0027 p.addParamValue('tight',false);
0028 p.addParamValue('lb',[]);
0029 p.addParamValue('ub',[]);
0030 p.parse(varargin{:});
0031 
0032 tight = p.Results.tight;
0033 default_lb = p.Results.lb;
0034 default_ub = p.Results.ub;
0035 if isempty(default_lb)
0036     default_lb = min(tiger.lb);
0037 end
0038 if isempty(default_ub)
0039     default_ub = max(tiger.ub);
0040 end
0041 
0042 assert(length(vars) == length(inds), ...
0043        'VARS and INDS must have the same length.');
0044 
0045 % make sure we have names
0046 [vars,var_idxs] = convert_ids(tiger.varnames,vars);
0047 [inds,ind_idxs] = convert_ids(tiger.varnames,inds);
0048 
0049 if p.Results.iff
0050     rules = cellzip(@(x,y) sprintf('"%s" ~= 0 <=> "%s"',x,y),vars,inds);
0051 
0052     if ~tight
0053         prev_lb = tiger.lb;
0054         prev_ub = tiger.ub;
0055         tiger.lb(var_idxs) = default_lb;
0056         tiger.ub(var_idxs) = default_ub;
0057 
0058         tiger = add_rule(tiger,rules);
0059 
0060         tiger.lb(1:length(prev_lb)) = prev_lb;
0061         tiger.ub(1:length(prev_ub)) = prev_ub;
0062     else
0063         tiger = add_rule(tiger,rules);
0064     end
0065 else
0066     N = length(var_idxs);
0067     A = zeros(2*N,size(tiger.A,2));
0068     ctypes = repmat(' ',2*N,1);
0069     for i = 1 : N
0070         if tight
0071             A(  i,[var_idxs(i) ind_idxs(i)]) = [1 -tiger.ub(var_idxs(i))];
0072             A(i+N,[var_idxs(i) ind_idxs(i)]) = [1 -tiger.lb(var_idxs(i))];
0073         else
0074             A(  i,[var_idxs(i) ind_idxs(i)]) = [1 -default_ub];
0075             A(i+N,[var_idxs(i) ind_idxs(i)]) = [1 -default_lb];
0076         end
0077         ctypes(  i) = '<';
0078         ctypes(i+N) = '>';
0079     end
0080     
0081     tiger = add_row(tiger,A,ctypes);
0082 end
```

---

Generated on Thu 11-Aug-2011 15:06:22 by **m2html** © 2005
