# Supplementary material for: TIGER: Toolbox for integrating genome-scale metabolic models, expression data, and transcriptional regulatory networks
Source: BMC Syst Biol. 2011 Sep 23;5:147. doi: 10.1186/1752-0509-5-147 (PMC3224351; doi:10.1186/1752-0509-5-147)
Supplement: Additional file 2 — TIGER source code. Source code, documentation, and tutorials are also available online at http://bme.virginia.edu/csbl/downloads/ or http://csbl.bitbucket.org/tiger. [file 1752-0509-5-147-S2.GZ › tiger/doc/m2html/tiger/find_infeasible_rules.html]

Description of find\_infeasible\_rules


Home > tiger > find\_infeasible\_rules.m

# find\_infeasible\_rules

## PURPOSE

**Determine which rules make a model infeasible.**

## SYNOPSIS

**function [infeasible,side] = find\_infeasible\_rules(tiger,rules)**

## DESCRIPTION

```
 FIND_INFEASIBLE_RULES  Determine which rules make a model infeasible.

   [INFEASIBLE,SIDE] = FIND_INFEASIBLE_RULES(TIGER,RULES,...params...)

   Find a minimal set of rules which cannot be satisfied when finding a
   feasible solution.  Reports which rules are not feasible and which 
   side of the rule is not satisfiable.

   Inputs
   TIGER   TIGER model structure
   RULES   Cell of strings or EXPR objects containing rules to test.
           These rules should not have been previously added to the TIGER
           model.

           If RULES is empty or the functions is called with only one
           argument, all previously added rules are removed from the 
           model and used in the infeasibility calculation.  The indices
           returned in INFEASIBLE reference the cell TIGER.param.rules.

   Outputs
   INFEASIBLE  Array of indices corresponding to rules in RULES that are
               not satisfiable in any feasible solution.
   SIDE        Character array describing the side of the rule that was
               infeasible.  'l' corresponds to the left side, 'r' is the
               right side.

   Parameters
   'display'   If true (default), display the infeasible rules.
```

## CROSS-REFERENCE INFORMATION

This function calls:

- add\_rule Add rules to a TIGER model
- make\_milp Convert a TIGER structure to a CMPI MILP.
- expr
- parse\_string Parse a rule string into an EXPR object
- array2names Create a cell of names from an array of numbers
- map Generate a new list by applying a function

This function is called by:

- infeas\_study
- load\_rules

## SUBFUNCTIONS

- function [new] = append\_s(e,side)

## SOURCE CODE

```
0001 function [infeasible,side] = find_infeasible_rules(tiger,rules)
0002 % FIND_INFEASIBLE_RULES  Determine which rules make a model infeasible.
0003 %
0004 %   [INFEASIBLE,SIDE] = FIND_INFEASIBLE_RULES(TIGER,RULES,...params...)
0005 %
0006 %   Find a minimal set of rules which cannot be satisfied when finding a
0007 %   feasible solution.  Reports which rules are not feasible and which
0008 %   side of the rule is not satisfiable.
0009 %
0010 %   Inputs
0011 %   TIGER   TIGER model structure
0012 %   RULES   Cell of strings or EXPR objects containing rules to test.
0013 %           These rules should not have been previously added to the TIGER
0014 %           model.
0015 %
0016 %           If RULES is empty or the functions is called with only one
0017 %           argument, all previously added rules are removed from the
0018 %           model and used in the infeasibility calculation.  The indices
0019 %           returned in INFEASIBLE reference the cell TIGER.param.rules.
0020 %
0021 %   Outputs
0022 %   INFEASIBLE  Array of indices corresponding to rules in RULES that are
0023 %               not satisfiable in any feasible solution.
0024 %   SIDE        Character array describing the side of the rule that was
0025 %               infeasible.  'l' corresponds to the left side, 'r' is the
0026 %               right side.
0027 %
0028 %   Parameters
0029 %   'display'   If true (default), display the infeasible rules.
0030 
0031 if nargin < 3 || isempty(indicators)
0032     indicators = false;
0033 end
0034 
0035 N = length(rules);
0036 exprs = cell(1,N);
0037 for i = 1 : N
0038     if isa(rules{i},'char')
0039         exprs{i} = parse_string(rules{i});
0040     else
0041         exprs{i} = rules{i}.copy;
0042     end
0043 end
0044 
0045 s_names = {};
0046 s_rules = [];
0047 
0048 for i = 1 : N
0049     if exprs{i}.IFF
0050         exprs{i}.lexpr = append_s(exprs{i}.lexpr,'l');
0051     end
0052     exprs{i}.rexpr = append_s(exprs{i}.rexpr,'r');
0053 end
0054 
0055 model = add_rule(tiger,exprs);
0056 
0057 % indicators
0058 if indicators
0059     ind_rows = find(model.ind);
0060     ind_inds = model.ind(ind_rows);
0061     and_vars = array2names('inf_ind_AND[%i]',ind_inds);
0062     or_vars  = array2names('inf_ind_OR[%i]',ind_inds);
0063     sub_vars = arary2names('inf_ind_SUB[%i]',ind_inds);
0064 end 
0065 
0066 [~,loc] = ismember(s_names,model.varnames);
0067 model.obj(:) = 0;
0068 model.obj(loc) = 1;
0069 
0070 sol = cmpi.solve_mip(make_milp(model));
0071 if isempty(sol.x)
0072     warning('Model cannot be made feasible.');
0073     infeasible = [];
0074     side = '';
0075     return;
0076 end
0077 
0078 state = logical(round(sol.x(loc)));
0079 infeasible = s_rules(state);
0080 side = map(@(x) x(end),s_names(state));
0081 
0082 function [new] = append_s(e,side)
0083     s_name = sprintf('_s%i%s',i,side);
0084     s_names{end+1} = s_name;
0085     s_rules(end+1) = i;
0086     new = expr();
0087     new.OR = true;
0088     new.lexpr = expr();
0089     new.lexpr.id = s_name;
0090     new.rexpr = e;
0091 end
0092 
0093 end
```

---

Generated on Thu 11-Aug-2011 15:06:22 by **m2html** © 2005
