# Supplementary material for: TIGER: Toolbox for integrating genome-scale metabolic models, expression data, and transcriptional regulatory networks
Source: BMC Syst Biol. 2011 Sep 23;5:147. doi: 10.1186/1752-0509-5-147 (PMC3224351; doi:10.1186/1752-0509-5-147)
Supplement: Additional file 2 — TIGER source code. Source code, documentation, and tutorials are also available online at http://bme.virginia.edu/csbl/downloads/ or http://csbl.bitbucket.org/tiger. [file 1752-0509-5-147-S2.GZ › tiger/doc/m2html/tiger/remove_null_rules.html]

Description of remove\_null\_rules


Home > tiger > remove\_null\_rules.m

# remove\_null\_rules

## PURPOSE

**Remove NULL rules from a TIGER model**

## SYNOPSIS

**function [tiger] = remove\_null\_rules(tiger,varargin)**

## DESCRIPTION

```
 REMOVE_NULL_RULES  Remove NULL rules from a TIGER model

   [TIGER] = REMOVE_NULL_RULES(TIGER,...params...)

   Removes any null rules that have been added to a TIGER model,
   including nulls left behind by REMOVE_RULE.

   If the parameter 'remove_rows' is true (default = false), rows in A
   that were created for the null rules are removed.
```

## CROSS-REFERENCE INFORMATION

This function calls:

- remove\_row Remove row(s) from a TIGER model

This function is called by:

- remove\_rule Remove rule(s) previously added to a TIGER model

## SOURCE CODE

```
0001 function [tiger] = remove_null_rules(tiger,varargin)
0002 % REMOVE_NULL_RULES  Remove NULL rules from a TIGER model
0003 %
0004 %   [TIGER] = REMOVE_NULL_RULES(TIGER,...params...)
0005 %
0006 %   Removes any null rules that have been added to a TIGER model,
0007 %   including nulls left behind by REMOVE_RULE.
0008 %
0009 %   If the parameter 'remove_rows' is true (default = false), rows in A
0010 %   that were created for the null rules are removed.
0011 
0012 p = inputParser;
0013 p.addParamValue('remove_rows',false);
0014 p.parse(varargin{:});
0015 
0016 remove_rows = p.Results.remove_rows;
0017 
0018 is_null = cellfun(@(x) x.NULL,tiger.param.rules);
0019 nulls = find(is_null);
0020 
0021 to_remove = false(size(tiger.A,1),1);
0022 rule_id = tiger.param.rule_id;
0023 for i = 1 : length(nulls)
0024     idx = nulls(i);
0025     if remove_rows
0026         to_remove = to_remove | rule_id == idx;
0027     else
0028         rule_id(rule_id == idx) = 0;
0029     end
0030     rule_id(rule_id > idx) = rule_id(rule_id > idx) - 1;
0031     nulls(i+1:end) = nulls(i+1:end) - 1;
0032 end
0033 
0034 tiger.param.rule_id = rule_id;
0035 tiger.param.rules = tiger.param.rules(~is_null);
0036 
0037 if remove_rows
0038     tiger = remove_row(tiger,to_remove);
0039 end
```

---

Generated on Thu 11-Aug-2011 15:06:22 by **m2html** © 2005
