# Supplementary material for: TIGER: Toolbox for integrating genome-scale metabolic models, expression data, and transcriptional regulatory networks
Source: BMC Syst Biol. 2011 Sep 23;5:147. doi: 10.1186/1752-0509-5-147 (PMC3224351; doi:10.1186/1752-0509-5-147)
Supplement: Additional file 2 — TIGER source code. Source code, documentation, and tutorials are also available online at http://bme.virginia.edu/csbl/downloads/ or http://csbl.bitbucket.org/tiger. [file 1752-0509-5-147-S2.GZ › tiger/doc/m2html/tiger/fva.html]

Description of fva


Home > tiger > fva.m

# fva

## PURPOSE

**Flux Variability Analysis**

## SYNOPSIS

**function [minflux,maxflux] = fva(tiger,varargin)**

## DESCRIPTION

```
 FVA  Flux Variability Analysis

   [MINFLUX,MAXFLUX] = FVA(TIGER,...params...)

   Calculates the minimum and maximum allowable flux through a reaction
   given a minimum fraction of the objective.

   Parameters
   'vars'    Indices of variables for the variability calculation.
             Default is all reactions in the S matrix.
   'frac'    Fraction of the objective fraction that must be satisfied by
             each flux distribution.  Default is 1.0.
   'status'  If true (default), a status bar is displayed.
```

## CROSS-REFERENCE INFORMATION

This function calls:

- add\_growth\_constraint Add minimum growth constraint to a model.
- convert\_ids Create name, indices, and logical indices from an array
- statusbar

This function is called by:

- eva Enzyme variability analysis

## SUBFUNCTIONS

- function [val] = get\_objval(model)

## SOURCE CODE

```
0001 function [minflux,maxflux] = fva(tiger,varargin)
0002 % FVA  Flux Variability Analysis
0003 %
0004 %   [MINFLUX,MAXFLUX] = FVA(TIGER,...params...)
0005 %
0006 %   Calculates the minimum and maximum allowable flux through a reaction
0007 %   given a minimum fraction of the objective.
0008 %
0009 %   Parameters
0010 %   'vars'    Indices of variables for the variability calculation.
0011 %             Default is all reactions in the S matrix.
0012 %   'frac'    Fraction of the objective fraction that must be satisfied by
0013 %             each flux distribution.  Default is 1.0.
0014 %   'status'  If true (default), a status bar is displayed.
0015 
0016 p = inputParser;
0017 p.addParamValue('vars',1:size(tiger.S,2));
0018 p.addParamValue('frac',1.0);
0019 p.addParamValue('status',true);
0020 p.parse(varargin{:});
0021 
0022 vars = convert_ids(tiger.varnames,p.Results.vars,'index');
0023 frac = p.Results.frac;
0024 status = p.Results.status;
0025 
0026 nvars = length(vars);
0027 minflux = zeros(nvars,1);
0028 maxflux = zeros(nvars,1);
0029 
0030 tiger = add_growth_constraint(tiger,frac);
0031 
0032 statbar = statusbar(nvars,status);
0033 statbar.start('Flux Variability status');
0034 for i = 1 : nvars
0035     tiger.obj(:) = 0;
0036     
0037     tiger.obj(vars(i)) = 1;
0038     
0039     tiger.sense = 1;
0040     minflux(i) = get_objval(tiger);
0041  
0042     tiger.sense = -1;
0043     maxflux(i) = get_objval(tiger);
0044     
0045     statbar.update(i);
0046 end
0047 
0048 
0049 function [val] = get_objval(model)
0050     sol = cmpi.solve_mip(model);
0051     if ~isempty(sol.x);
0052         val = sol.val;
0053     else
0054         val = NaN;
0055     end
0056
```

---

Generated on Thu 11-Aug-2011 15:06:22 by **m2html** © 2005
