# Supplementary material for: TIGER: Toolbox for integrating genome-scale metabolic models, expression data, and transcriptional regulatory networks
Source: BMC Syst Biol. 2011 Sep 23;5:147. doi: 10.1186/1752-0509-5-147 (PMC3224351; doi:10.1186/1752-0509-5-147)
Supplement: Additional file 2 — TIGER source code. Source code, documentation, and tutorials are also available online at http://bme.virginia.edu/csbl/downloads/ or http://csbl.bitbucket.org/tiger. [file 1752-0509-5-147-S2.GZ › tiger/doc/m2html/tiger/remove_column.html]

Description of remove\_column


Home > tiger > remove\_column.m

# remove\_column

## PURPOSE

**Remove column(s) from a TIGER model**

## SYNOPSIS

**function [tiger] = remove\_column(tiger,col\_ids)**

## DESCRIPTION

```
 REMOVE_COLUMN  Remove column(s) from a TIGER model

   [TIGER] = REMOVE_COLUMN(TIGER,COL_IDS)

   Remove columns COL_IDS from a TIGER model and return the modified
   structure.  COL_IDS are any valid IDs (see CONVERT_IDS).
```

## CROSS-REFERENCE INFORMATION

This function calls:

- convert\_ids Create name, indices, and logical indices from an array

This function is called by:


## SOURCE CODE

```
0001 function [tiger] = remove_column(tiger,col_ids)
0002 % REMOVE_COLUMN  Remove column(s) from a TIGER model
0003 %
0004 %   [TIGER] = REMOVE_COLUMN(TIGER,COL_IDS)
0005 %
0006 %   Remove columns COL_IDS from a TIGER model and return the modified
0007 %   structure.  COL_IDS are any valid IDs (see CONVERT_IDS).
0008 
0009 ids = ~convert_ids(tiger.varnames,col_ids,'logical');
0010 
0011 tiger.A = tiger.A(:,ids);
0012 tiger.lb = tiger.lb(ids);
0013 tiger.ub = tiger.ub(ids);
0014 
0015 tiger.obj = tiger.obj(ids);
0016 
0017 tiger.varnames = tiger.varnames(ids);
0018 tiger.vartypes = tiger.vartypes(ids);
0019 
0020 tiger.param.fixedvar = tiger.param.fixedvar(ids);
0021 
0022 % update TIGER.ind
0023 removed = find(~ids);
0024 ind = tiger.ind;
0025 for i = 1 : length(removed)
0026     ind(ind > removed(i)) = ind(ind > removed(i)) - 1;
0027 end
0028 tiger.ind = ind;
```

---

Generated on Thu 11-Aug-2011 15:06:22 by **m2html** © 2005
