# Supplementary material for: TIGER: Toolbox for integrating genome-scale metabolic models, expression data, and transcriptional regulatory networks
Source: BMC Syst Biol. 2011 Sep 23;5:147. doi: 10.1186/1752-0509-5-147 (PMC3224351; doi:10.1186/1752-0509-5-147)
Supplement: Additional file 2 — TIGER source code. Source code, documentation, and tutorials are also available online at http://bme.virginia.edu/csbl/downloads/ or http://csbl.bitbucket.org/tiger. [file 1752-0509-5-147-S2.GZ › tiger/doc/m2html/tiger/make_milp.html]

Description of make\_milp


Home > tiger > make\_milp.m

# make\_milp

## PURPOSE

**Convert a TIGER structure to a CMPI MILP.**

## SYNOPSIS

**function [milp] = make\_milp(tiger,sense)**

## DESCRIPTION

```
 MAKE_MILP  Convert a TIGER structure to a CMPI MILP.

   [MILP] = MAKE_MILP(TIGER,SENSE)

   Create a CMPI MILP structure froma TIGER model.  SENSE can be either
   a numerical sense (1 -> min, -1 -> max), or a string ('max' or 'min').
```

## CROSS-REFERENCE INFORMATION

This function calls:


This function is called by:

- find\_infeasible\_rules Determine which rules make a model infeasible.
- show\_tiger Show a TIGER model as a MIP

## SUBFUNCTIONS

- function copy\_if(field)

## SOURCE CODE

```
0001 function [milp] = make_milp(tiger,sense)
0002 % MAKE_MILP  Convert a TIGER structure to a CMPI MILP.
0003 %
0004 %   [MILP] = MAKE_MILP(TIGER,SENSE)
0005 %
0006 %   Create a CMPI MILP structure froma TIGER model.  SENSE can be either
0007 %   a numerical sense (1 -> min, -1 -> max), or a string ('max' or 'min').
0008 
0009 if nargin < 2 || isempty(sense)
0010     sense = 1;
0011 end
0012 if isa(sense,'char')
0013     switch sense
0014         case {'max','maximize'}
0015             milp.sense = -1;
0016         case {'min','minimize'}
0017             milp.sense =  1;
0018     end
0019 else
0020     milp.sense = sense;
0021 end
0022 
0023 milp.obj = tiger.obj;
0024 milp.A = tiger.A;
0025 milp.b = tiger.b;
0026 
0027 milp.lb = tiger.lb;
0028 milp.ub = tiger.ub;
0029 
0030 milp.ctypes = tiger.ctypes';
0031 milp.vartypes = upper(tiger.vartypes');
0032 
0033 milp.varnames = tiger.varnames;
0034 milp.rownames = tiger.rownames;
0035 
0036 copy_if('ind');
0037 copy_if('indtypes');
0038 
0039 copy_if('Q');
0040 copy_if('Qd');
0041 copy_if('Qc');
0042 
0043 
0044 function copy_if(field)
0045     if isfield(tiger,field)
0046         milp.(field) = tiger.(field);
0047     end
0048 end
0049 
0050 end
```

---

Generated on Thu 11-Aug-2011 15:06:22 by **m2html** © 2005
