# Supplementary material for: TIGER: Toolbox for integrating genome-scale metabolic models, expression data, and transcriptional regulatory networks
Source: BMC Syst Biol. 2011 Sep 23;5:147. doi: 10.1186/1752-0509-5-147 (PMC3224351; doi:10.1186/1752-0509-5-147)
Supplement: Additional file 2 — TIGER source code. Source code, documentation, and tutorials are also available online at http://bme.virginia.edu/csbl/downloads/ or http://csbl.bitbucket.org/tiger. [file 1752-0509-5-147-S2.GZ › tiger/doc/m2html/tiger/update_rule.html]

Description of update\_rule


Home > tiger > update\_rule.m

# update\_rule

## PURPOSE

**Re-compile rule(s) previously added to a TIGER model**

## SYNOPSIS

**function [tiger] = update\_rule(tiger,old\_rules,new\_rules,varargin)**

## DESCRIPTION

```
 UPDATE_RULE  Re-compile rule(s) previously added to a TIGER model

   [TIGER] = UPDATE_RULE(TIGER,OLD_RULES,NEW_RULES,...params...)

   UPDATE_RULE takes a list of rules (OLD_RULES) and replaces them with
   NEW_RULES.  The modified model structure is returned.

   If any entry in NEW_RULES is empty, or if only two arguments are 
   given, then the rules in OLD_RULES are recompiled.

   If OLD_RULES is empty (or not given), then all rules are re-compiled.

   Parameters given to the function are passed to REMOVE_RULE.
```

## CROSS-REFERENCE INFORMATION

This function calls:

- add\_rule Add rules to a TIGER model
- remove\_rule Remove rule(s) previously added to a TIGER model

This function is called by:


## SOURCE CODE

```
0001 function [tiger] = update_rule(tiger,old_rules,new_rules,varargin)
0002 % UPDATE_RULE  Re-compile rule(s) previously added to a TIGER model
0003 %
0004 %   [TIGER] = UPDATE_RULE(TIGER,OLD_RULES,NEW_RULES,...params...)
0005 %
0006 %   UPDATE_RULE takes a list of rules (OLD_RULES) and replaces them with
0007 %   NEW_RULES.  The modified model structure is returned.
0008 %
0009 %   If any entry in NEW_RULES is empty, or if only two arguments are
0010 %   given, then the rules in OLD_RULES are recompiled.
0011 %
0012 %   If OLD_RULES is empty (or not given), then all rules are re-compiled.
0013 %
0014 %   Parameters given to the function are passed to REMOVE_RULE.
0015 
0016 if nargin < 2 || isempty(old_rules)
0017     old_rules = tiger.param.rules;
0018 end
0019 
0020 if nargin < 3 || isempty(new_rules)
0021     new_rules = old_rules;
0022 end
0023 
0024 N = length(old_rules);
0025 for i = 1 : N
0026     tiger = remove_rule(tiger,old_rules{i},varargin{:});
0027     if isempty(new_rules{i})
0028         new_rules{i} = old_rules{i}.copy;
0029     end
0030 end
0031 
0032 tiger = add_rule(tiger,new_rules);
```

---

Generated on Thu 11-Aug-2011 15:06:22 by **m2html** © 2005
