# Supplementary material for: TIGER: Toolbox for integrating genome-scale metabolic models, expression data, and transcriptional regulatory networks
Source: BMC Syst Biol. 2011 Sep 23;5:147. doi: 10.1186/1752-0509-5-147 (PMC3224351; doi:10.1186/1752-0509-5-147)
Supplement: Additional file 2 — TIGER source code. Source code, documentation, and tutorials are also available online at http://bme.virginia.edu/csbl/downloads/ or http://csbl.bitbucket.org/tiger. [file 1752-0509-5-147-S2.GZ › tiger/doc/m2html/tiger/add_growth_constraint.html]

Description of add\_growth\_constraint


Home > tiger > add\_growth\_constraint.m

# add\_growth\_constraint

## PURPOSE

**Add minimum growth constraint to a model.**

## SYNOPSIS

**function [tiger,sol] = add\_growth\_constraint(tiger,val,varargin)**

## DESCRIPTION

```
 ADD_GROWTH_CONSTRAINT  Add minimum growth constraint to a model.

   [TIGER,SOL] = ADD_GROWTH_CONSTRAINT(TIGER,VAL,...params...)

   Adds a constraint that requires a minimum flux through the objective
   reaction.

   Inputs
   TIGER   TIGER model structure.
   VAL     Constraining value (see the 'valtype' parameter for details).

   Outputs
   TIGER   TIGER model with growth constraint added.
   SOL     CMPI solution object from the FBA calculation.

   Parameters
   'ctype'     Character indicating the type of constraint to add:
                   '>'  -->  v_obj >= VAL  (default)
                   '='  -->  v_obj  = VAL
   'valtype'   If set to 'frac' (default), the argument VAL is a fraction
               of maximum objective flux that must be acheived.  FBA is
               run to determine the maximum value.  If set to 'abs', the
               argument VAL is interpreted as an actual flux value to be
               acheived.
```

## CROSS-REFERENCE INFORMATION

This function calls:

- add\_row Add a row to a TIGER model structure
- fba Run Flux Balance Analysis on a TIGER model.

This function is called by:

- mea
- fba Run Flux Balance Analysis on a TIGER model.
- fva Flux Variability Analysis
- minimal\_genome Calculate a minimal genome
- infeas\_study
- load\_rules
- gal\_test
- run\_trn\_comparison
- diffadj Formulate and solve the differential adjustment problem
- gimme Gene Inactivity Moderated by Metabolism and Expression
- imat Integrative Metabolic Analysis Tool

## SOURCE CODE

```
0001 function [tiger,sol] = add_growth_constraint(tiger,val,varargin)
0002 % ADD_GROWTH_CONSTRAINT  Add minimum growth constraint to a model.
0003 %
0004 %   [TIGER,SOL] = ADD_GROWTH_CONSTRAINT(TIGER,VAL,...params...)
0005 %
0006 %   Adds a constraint that requires a minimum flux through the objective
0007 %   reaction.
0008 %
0009 %   Inputs
0010 %   TIGER   TIGER model structure.
0011 %   VAL     Constraining value (see the 'valtype' parameter for details).
0012 %
0013 %   Outputs
0014 %   TIGER   TIGER model with growth constraint added.
0015 %   SOL     CMPI solution object from the FBA calculation.
0016 %
0017 %   Parameters
0018 %   'ctype'     Character indicating the type of constraint to add:
0019 %                   '>'  -->  v_obj >= VAL  (default)
0020 %                   '='  -->  v_obj  = VAL
0021 %   'valtype'   If set to 'frac' (default), the argument VAL is a fraction
0022 %               of maximum objective flux that must be acheived.  FBA is
0023 %               run to determine the maximum value.  If set to 'abs', the
0024 %               argument VAL is interpreted as an actual flux value to be
0025 %               acheived.
0026 
0027 if nargin < 2
0028     error('two inputs required');
0029 end
0030 
0031 p = inputParser;
0032 p.addParamValue('ctype','>');
0033 p.addParamValue('valtype','frac');
0034 p.parse(varargin{:});
0035 
0036 switch p.Results.valtype
0037     case 'frac'
0038         sol = fba(tiger);
0039 %         if sol.val > 1e-8
0040 %             warning('FBA objective near zero.');
0041 %         end
0042         value = val*sol.val;
0043     case 'abs'
0044         value = val;
0045 end
0046 
0047 tiger = add_row(tiger,1);
0048 
0049 tiger.A(end,:) = tiger.obj';
0050 tiger.b(end) = value;
0051 tiger.ctypes(end) = p.Results.ctype;
0052 tiger.rownames{end} = 'GROWTH';
0053
```

---

Generated on Thu 11-Aug-2011 15:06:22 by **m2html** © 2005
