# Supplementary material for: TIGER: Toolbox for integrating genome-scale metabolic models, expression data, and transcriptional regulatory networks
Source: BMC Syst Biol. 2011 Sep 23;5:147. doi: 10.1186/1752-0509-5-147 (PMC3224351; doi:10.1186/1752-0509-5-147)
Supplement: Additional file 2 — TIGER source code. Source code, documentation, and tutorials are also available online at http://bme.virginia.edu/csbl/downloads/ or http://csbl.bitbucket.org/tiger. [file 1752-0509-5-147-S2.GZ › tiger/doc/m2html/tiger/cobra_to_tiger.html]

Description of cobra\_to\_tiger


Home > tiger > cobra\_to\_tiger.m

# cobra\_to\_tiger

## PURPOSE

**Convert a COBRA model to a TIGER model**

## SYNOPSIS

**function [tiger] = cobra\_to\_tiger(cobra,add\_gpr,varargin)**

## DESCRIPTION

```
 COBRA_TO_TIGER  Convert a COBRA model to a TIGER model

   [TIGER] = COBRA_TO_TIGER(COBRA,CONVERT_GPR,...ADD_RULE params...)

   Convert a COBRA model structure to a TIGER model structure.

   Inputs
   COBRA        COBRA toolbox model structure
   CONVERT_GPR  If true, add the GPR constraints as rules.
                (default = true)
   params       Extra parameters are passed to ADD_RULE.

   Outputs
   TIGER        TIGER model structure.
```

## CROSS-REFERENCE INFORMATION

This function calls:

- convert\_gpr Add the GPR rules as constraints to the model.
- create\_empty\_tiger Create an empty TIGER model structure.
- array2names Create a cell of names from an array of numbers

This function is called by:

- assert\_tiger Assert that a structure is an TIGER model.
- cobra\_to\_elf Create an ELF model from a COBRA structure
- test\_\_fba
- test\_\_gimme
- test\_\_imat
- test\_\_indicators
- test\_\_made
- test\_\_solve\_multiple\_mips
- test\_\_tile\_mip
- convert\_rules
- create\_yeast\_trn\_model
- load\_rules
- imat Integrative Metabolic Analysis Tool

## SOURCE CODE

```
0001 function [tiger] = cobra_to_tiger(cobra,add_gpr,varargin)
0002 % COBRA_TO_TIGER  Convert a COBRA model to a TIGER model
0003 %
0004 %   [TIGER] = COBRA_TO_TIGER(COBRA,CONVERT_GPR,...ADD_RULE params...)
0005 %
0006 %   Convert a COBRA model structure to a TIGER model structure.
0007 %
0008 %   Inputs
0009 %   COBRA        COBRA toolbox model structure
0010 %   CONVERT_GPR  If true, add the GPR constraints as rules.
0011 %                (default = true)
0012 %   params       Extra parameters are passed to ADD_RULE.
0013 %
0014 %   Outputs
0015 %   TIGER        TIGER model structure.
0016 
0017 if nargin < 2 || isempty(add_gpr)
0018     add_gpr = true;
0019 end
0020 
0021 tiger = rmfield(cobra,'c');
0022 
0023 % get default params
0024 empty_tiger = create_empty_tiger();
0025 tiger.param = empty_tiger.param;
0026 
0027 [m,n] = size(tiger.S);
0028 
0029 if isfield(cobra,'rxns')
0030     tiger.varnames = cobra.rxns(:);
0031 else
0032     tiger.varnames = array2names('rxn',1:n)';
0033 end
0034 
0035 if isfield(cobra,'mets')
0036     tiger.rownames = cobra.mets(:);
0037 else
0038     tiger.rownames = array2names('row',1:m)';
0039 end
0040 
0041 tiger.A = tiger.S;
0042 
0043 tiger.obj = cobra.c(:);
0044 tiger.ctypes = repmat('=',m,1);
0045 tiger.vartypes = repmat('c',n,1);
0046 
0047 tiger.gpr = cobra.grRules(:);
0048 tiger.genes = cobra.genes(:);
0049 
0050 tiger.ind = zeros(m,1);
0051 tiger.indtypes = repmat(' ',m,1);
0052 
0053 if add_gpr
0054     % reset bounds
0055     orig_N = size(cobra.S,2);
0056     orig_lb = tiger.lb;
0057     orig_ub = tiger.ub;
0058 
0059     tiger.lb(:) = min(tiger.lb);
0060     tiger.ub(:) = max(tiger.ub);
0061     
0062     tiger = convert_gpr(tiger,varargin{:});
0063     
0064     tiger.lb(1:orig_N) = orig_lb;
0065     tiger.ub(1:orig_N) = orig_ub;
0066 end
0067 
0068 
0069 
0070
```

---

Generated on Thu 11-Aug-2011 15:06:22 by **m2html** © 2005
