# Supplementary material for: TIGER: Toolbox for integrating genome-scale metabolic models, expression data, and transcriptional regulatory networks
Source: BMC Syst Biol. 2011 Sep 23;5:147. doi: 10.1186/1752-0509-5-147 (PMC3224351; doi:10.1186/1752-0509-5-147)
Supplement: Additional file 2 — TIGER source code. Source code, documentation, and tutorials are also available online at http://bme.virginia.edu/csbl/downloads/ or http://csbl.bitbucket.org/tiger. [file 1752-0509-5-147-S2.GZ › tiger/doc/m2html/tiger/moma.html]

Description of moma


Home > tiger > moma.m

# moma

## PURPOSE

**Minimization of Metabolic Adjustment**

## SYNOPSIS

**function [sol] = moma(tiger,flux\_vals,flux\_ids)**

## DESCRIPTION

```
 MOMA  Minimization of Metabolic Adjustment

   [SOL] = MOMA(TIGER,FLUX_VALS)
   [SOL] = MOMA(TIGER,FLUX_VALS,FLUX_IDS)

   Calculates and returns a CMPI solution structure for the MOMA
   algorithm:
       minimize (v - FLUX_VALS)^2
       s.t.  Sv = 0
             lb <= v <= ub

   If only a subset of fluxes are specified, the indices can be given in
   the vector FLUX_IDS.  The corresponding objective is then:
       minimize (v(FLUX_IDS) - FLUX_VALS)^2
```

## CROSS-REFERENCE INFORMATION

This function calls:

- convert\_ids Create name, indices, and logical indices from an array

This function is called by:


## SOURCE CODE

```
0001 function [sol] = moma(tiger,flux_vals,flux_ids)
0002 % MOMA  Minimization of Metabolic Adjustment
0003 %
0004 %   [SOL] = MOMA(TIGER,FLUX_VALS)
0005 %   [SOL] = MOMA(TIGER,FLUX_VALS,FLUX_IDS)
0006 %
0007 %   Calculates and returns a CMPI solution structure for the MOMA
0008 %   algorithm:
0009 %       minimize (v - FLUX_VALS)^2
0010 %       s.t.  Sv = 0
0011 %             lb <= v <= ub
0012 %
0013 %   If only a subset of fluxes are specified, the indices can be given in
0014 %   the vector FLUX_IDS.  The corresponding objective is then:
0015 %       minimize (v(FLUX_IDS) - FLUX_VALS)^2
0016 
0017 N = size(tiger.A,2);
0018 
0019 if nargin < 3
0020     flux_ids = 1 : size(tiger.S,2);
0021 end
0022 
0023 flux_idxs = convert_ids(tiger.varnames,flux_ids,'index');
0024 
0025 tiger.Qc.w = zeros(N,1);
0026 tiger.Qc.c = zeros(N,1);
0027 
0028 tiger.Qc.w(flux_idxs) = 1;
0029 tiger.Qc.c(flux_idxs) = flux_vals;
0030 
0031 tiger.obj(:) = 0;
0032 
0033 sol = cmpi.solve_mip(tiger);
```

---

Generated on Thu 11-Aug-2011 15:06:22 by **m2html** © 2005
