# Supplementary material for: TIGER: Toolbox for integrating genome-scale metabolic models, expression data, and transcriptional regulatory networks
Source: BMC Syst Biol. 2011 Sep 23;5:147. doi: 10.1186/1752-0509-5-147 (PMC3224351; doi:10.1186/1752-0509-5-147)
Supplement: Additional file 2 — TIGER source code. Source code, documentation, and tutorials are also available online at http://bme.virginia.edu/csbl/downloads/ or http://csbl.bitbucket.org/tiger. [file 1752-0509-5-147-S2.GZ › tiger/doc/m2html/tiger/elf/index.html]

Index for Directory tiger/elf


|  |  |
| --- | --- |
| Master index | Index for tiger/elf |

# Index for tiger/elf

## Matlab files in this directory:

|  |  |
| --- | --- |
| cobra\_to\_elf | Create an ELF model from a COBRA structure |
| dare |  |
| eva | Enzyme variability analysis |
| mea |  |
| remove\_rev\_cons | Remove reversibility constraints from an ELF model |
| restore\_rev\_cons | Restore reversibility constraints in an ELF model |

---

Generated on Thu 11-Aug-2011 15:06:20 by **m2html** © 2005
