# Supplementary material for: TIGER: Toolbox for integrating genome-scale metabolic models, expression data, and transcriptional regulatory networks
Source: BMC Syst Biol. 2011 Sep 23;5:147. doi: 10.1186/1752-0509-5-147 (PMC3224351; doi:10.1186/1752-0509-5-147)
Supplement: Additional file 2 — TIGER source code. Source code, documentation, and tutorials are also available online at http://bme.virginia.edu/csbl/downloads/ or http://csbl.bitbucket.org/tiger. [file 1752-0509-5-147-S2.GZ › tiger/doc/m2html/tiger/elf/mea.html]

Description of mea


Home > tiger > elf > mea.m

# mea

## PURPOSE

## SYNOPSIS

**function [sol] = mea(elf,actnorm,norun,obj\_frac)**

## DESCRIPTION

## CROSS-REFERENCE INFORMATION

This function calls:

- add\_growth\_constraint Add minimum growth constraint to a model.
- convert\_ids Create name, indices, and logical indices from an array

This function is called by:

- dare

## SOURCE CODE

```
0001 function [sol] = mea(elf,actnorm,norun,obj_frac)
0002 
0003 if nargin < 4 || isempty(obj_frac)
0004     obj_frac = 0.99;
0005 end
0006 
0007 if nargin < 3 || isempty(norun)
0008     norun = false;
0009 end
0010 
0011 if nargin < 2 || isempty(actnorm)
0012     actnorm = 'euclid';
0013 end
0014 
0015 elf = add_growth_constraint(elf,obj_frac);
0016 elf.obj(:) = 0;
0017 nA = size(elf.A,2);
0018 idxs = convert_ids(elf.varnames,elf.genes,'index');
0019 
0020 no_gpr_rxns = find(cellfun(@isempty,elf.grRules));
0021 
0022 idxs = [idxs; no_gpr_rxns];
0023 
0024 switch actnorm
0025     case {'euclid','quad','two'}
0026         elf.Q = spalloc(nA,nA,length(idxs));
0027         for i = 1 : length(idxs)
0028             elf.Q(idxs(i),idxs(i)) = 1;
0029         end
0030     case {'one','taxi','manhattan'}
0031         elf.obj(idxs) = 1;
0032 end
0033 
0034 if norun
0035     sol = elf;
0036 else
0037     sol = cmpi.solve_mip(elf);
0038 end
0039
```

---

Generated on Thu 11-Aug-2011 15:06:22 by **m2html** © 2005
