# Supplementary material for: TIGER: Toolbox for integrating genome-scale metabolic models, expression data, and transcriptional regulatory networks
Source: BMC Syst Biol. 2011 Sep 23;5:147. doi: 10.1186/1752-0509-5-147 (PMC3224351; doi:10.1186/1752-0509-5-147)
Supplement: Additional file 2 — TIGER source code. Source code, documentation, and tutorials are also available online at http://bme.virginia.edu/csbl/downloads/ or http://csbl.bitbucket.org/tiger. [file 1752-0509-5-147-S2.GZ › tiger/doc/m2html/tiger/elf/dare.html]

Description of dare


Home > tiger > elf > dare.m

# dare

## PURPOSE

## SYNOPSIS

**function [fluxes] = dare(elf,fold\_change,gene\_names,alpha,obj\_frac,bounds)**

## DESCRIPTION

## CROSS-REFERENCE INFORMATION

This function calls:

- mea
- diffadj Formulate and solve the differential adjustment problem

This function is called by:


## SOURCE CODE

```
0001 function [fluxes] = dare(elf,fold_change,gene_names,alpha,obj_frac,bounds)
0002 
0003 if nargin < 6 || isempty(bounds)
0004     bounds = [];
0005 end
0006 
0007 [ngenes,ntrans] = size(fold_change);
0008 assert(ngenes == length(gene_names), ...
0009        'FOLD_CHANGE and GENE_NAMES must have the same # of rows');
0010 
0011 d = ones(ngenes,ntrans);
0012 d(fold_change < 1) = -1;
0013 
0014 [tf,gene_locs] = ismember(gene_names,elf.varnames);
0015 genes = gene_names(tf);
0016 gene_locs = gene_locs(tf);
0017 fold_change = fold_change(tf,:);
0018 
0019 ngenes = length(genes);
0020 
0021 mip = mea(elf,[],true,0);
0022 mip.Q = (1-alpha) .* mip.Q;
0023 weights = alpha*(fold_change - 1);
0024 
0025 [~,sol] = diffadj(mip,genes,d,-weights,[],bounds,[],obj_frac);
0026 
0027 if ~isempty(sol.x)
0028     fluxes = cell(1,ntrans+1);
0029     n = size(elf.A,2);
0030     for i = 1 : ntrans + 1
0031         fluxes{i} = sol.x((i-1)*n+(1:n));
0032     end
0033 else
0034     fluxes = [];
0035 end
```

---

Generated on Thu 11-Aug-2011 15:06:22 by **m2html** © 2005
