# Supplementary material for: TIGER: Toolbox for integrating genome-scale metabolic models, expression data, and transcriptional regulatory networks
Source: BMC Syst Biol. 2011 Sep 23;5:147. doi: 10.1186/1752-0509-5-147 (PMC3224351; doi:10.1186/1752-0509-5-147)
Supplement: Additional file 2 — TIGER source code. Source code, documentation, and tutorials are also available online at http://bme.virginia.edu/csbl/downloads/ or http://csbl.bitbucket.org/tiger. [file 1752-0509-5-147-S2.GZ › tiger/doc/m2html/tiger/elf/cobra_to_elf.html]

Description of cobra\_to\_elf


Home > tiger > elf > cobra\_to\_elf.m

# cobra\_to\_elf

## PURPOSE

**Create an ELF model from a COBRA structure**

## SYNOPSIS

**function [elf] = cobra\_to\_elf(cobra,varargin)**

## DESCRIPTION

```
 COBRA_TO_ELF  Create an ELF model from a COBRA structure

   [ELF] = COBRA_TO_ELF(COBRA,...params...)

   Create an ELF model structure from an existing COBRA structure.  The
   following naming conventions are used for rows and columns added to 
   the model:
       Columns:
           RGB(i)_v    The ith RGB for reaction v.
           v__f        Forward portion of a reversible reaction.
           v__r        Reverse portion of a reversible reaction.
           v_FOR_IND   Indicator; true if a forward portion carries flux.
           v_REV_IND   Indicator; true if a reverse portion carries flux.

       If the parameter 'gene_indicators' is true (default = false), a
       set of indicator variables I_x are added for each gene x.  These
       variables are bound to the gene activities.  A function handle
       'indof' is added to ELF, where indof(gene) is the index of I_gene.

       Rows:
           SUM_RGB_v   Original flux v must sum to the fluxes of all 
                       corresponding RGBs.
           SUM_FR_v    Original flux v must sum to the forward minus
                       reverse reations.
           ELF_REV_CON1 - ELF_REV_CON3  Reversibility constraints; a
                       forward and reverse reaction cannot both carry
                       flux in the same solution vector.
```

## CROSS-REFERENCE INFORMATION

This function calls:

- add\_column Add a column to a TIGER model structure
- add\_row Add a row to a TIGER model structure
- bind\_var Bind variables to a indicator variable
- cobra\_to\_tiger Convert a COBRA model to a TIGER model
- make\_dnf Make lists of atoms in the Disjunctive Normal Form
- parse\_string Parse a rule string into an EXPR object
- array2names Create a cell of names from an array of numbers
- map Generate a new list by applying a function

This function is called by:


## SUBFUNCTIONS

- function add\_reactants(names,rxn\_idx)
- function [tiger] = make\_irreversible\_rxns(tiger)

## SOURCE CODE

```
0001 function [elf] = cobra_to_elf(cobra,varargin)
0002 % COBRA_TO_ELF  Create an ELF model from a COBRA structure
0003 %
0004 %   [ELF] = COBRA_TO_ELF(COBRA,...params...)
0005 %
0006 %   Create an ELF model structure from an existing COBRA structure.  The
0007 %   following naming conventions are used for rows and columns added to
0008 %   the model:
0009 %       Columns:
0010 %           RGB(i)_v    The ith RGB for reaction v.
0011 %           v__f        Forward portion of a reversible reaction.
0012 %           v__r        Reverse portion of a reversible reaction.
0013 %           v_FOR_IND   Indicator; true if a forward portion carries flux.
0014 %           v_REV_IND   Indicator; true if a reverse portion carries flux.
0015 %
0016 %       If the parameter 'gene_indicators' is true (default = false), a
0017 %       set of indicator variables I_x are added for each gene x.  These
0018 %       variables are bound to the gene activities.  A function handle
0019 %       'indof' is added to ELF, where indof(gene) is the index of I_gene.
0020 %
0021 %       Rows:
0022 %           SUM_RGB_v   Original flux v must sum to the fluxes of all
0023 %                       corresponding RGBs.
0024 %           SUM_FR_v    Original flux v must sum to the forward minus
0025 %                       reverse reations.
0026 %           ELF_REV_CON1 - ELF_REV_CON3  Reversibility constraints; a
0027 %                       forward and reverse reaction cannot both carry
0028 %                       flux in the same solution vector.
0029 
0030 p = inputParser;
0031 p.addParamValue('gene_indicators',false);
0032 p.parse();
0033 
0034 make_gene_inds = p.Results.gene_indicators;
0035     
0036 elf = cobra_to_tiger(cobra,false);
0037 genes = elf.genes;
0038 ngenes = length(genes);
0039 
0040 elf = add_row(elf,[],'=',0,genes);
0041 nmets = size(elf.A,1);
0042 elf = make_irreversible_rxns(elf);
0043 nrxns = size(elf.A,2);
0044 
0045 act_bound = max(max(abs(elf.lb),abs(elf.ub)));
0046 
0047 % define gene activities
0048 elf = add_column(elf,genes,'c',0,act_bound);
0049 elf.A(size(cobra.S,1)+(1:ngenes),end-ngenes+1:end) = eye(ngenes);
0050 
0051 % define binary gene indicators
0052 if make_gene_inds
0053     elf.indof = @(x) ['I_' x];
0054     gene_inds = map(elf.indof,genes);
0055     elf = add_column(elf,gene_inds,'b');
0056     elf = bind_var(elf,genes,gene_inds);
0057 end
0058     
0059 % at this point, only irreversible reactions have GPRs
0060 
0061 gpr = elf.gpr;
0062 for i = 1 : nrxns
0063     if isempty(gpr{i})
0064         continue;
0065     end
0066     
0067     e = parse_string(gpr{i});
0068     ands = make_dnf(e,true);
0069     if length(ands) == 1
0070         add_reactants(ands{1},i);
0071     else
0072         N = length(ands);
0073         n = size(elf.A,2);
0074         rgb_names = array2names(['RGB(%i)_' elf.varnames{i}],1:N);
0075         elf = add_column(elf,rgb_names,'c',0,act_bound);
0076         for j = 1 : N
0077             elf.A(1:nmets,n+j) = elf.A(1:nmets,i);
0078             add_reactants(ands{j},n+j);
0079         end
0080         elf.A(1:nmets,i) = 0;
0081         elf = add_row(elf,[],'=',0,['SUM_RGB_' elf.varnames{i}]);
0082         elf.A(end,i) = -1;
0083         elf.A(end,n+1:n+j) = 1;
0084     end
0085 end
0086 
0087 function add_reactants(names,rxn_idx)
0088     [~,locs] = ismember(names,elf.rownames);
0089     elf.A(locs,rxn_idx) = -1;
0090 end
0091 
0092 end
0093      
0094 
0095 function [tiger] = make_irreversible_rxns(tiger)
0096     if ~isfield(tiger,'rev')
0097         rev = tiger.lb < 0;
0098     else
0099         rev = tiger.rev;
0100     end
0101     
0102     n = size(tiger.A,2);
0103     for i = 1 : n
0104         if rev(i)
0105             % add forward and reverse reactions
0106             f = size(tiger.A,2) + 1;
0107             r = f + 1;
0108             tiger = add_column(tiger, ...
0109                                [tiger.varnames{i} '__f'], ...
0110                                'c',0,tiger.ub(i));
0111             tiger.A(:,f) = tiger.A(:,i);
0112             tiger = add_column(tiger, ...
0113                                [tiger.varnames{i} '__r'], ...
0114                                'c',0,-tiger.lb(i));
0115             tiger.A(:,r) = -tiger.A(:,i);
0116             
0117             % move the GPR onto the new reactions
0118             tiger.gpr{f} = tiger.gpr{i};
0119             tiger.gpr{r} = tiger.gpr{i};
0120             tiger.gpr{i} = '';
0121             
0122             % sum the forward and reverse rxns into the original
0123             tiger.A(:,i) = 0;
0124             tiger = add_row(tiger,[],'=',0,['SUM_FR_' tiger.varnames{i}]);
0125             tiger.A(end,[i f r]) = [-1 1 -1];
0126             
0127             % add indicator to avoid flux loops
0128             f_ind = f + 2;
0129             r_ind = f + 3;
0130             ind_names = {[tiger.varnames{i} '_FOR_IND'], ...
0131                          [tiger.varnames{i} '_REV_IND']};
0132             tiger = add_column(tiger,ind_names);
0133             tiger = add_row(tiger,[],'<<=',[],'ELF_REV_CON%i');
0134             tiger.A(end-2,[f f_ind]) = [1 -tiger.ub(f)];
0135             tiger.A(end-1,[r r_ind]) = [1 -tiger.ub(r)];
0136             tiger.A(end,[f_ind r_ind]) = [1 1];
0137             tiger.b(end) = 1;
0138             
0139             % hack TODO: move indicators to end of reaction list
0140             tiger.gpr{f_ind} = '';
0141             tiger.gpr{r_ind} = '';
0142         end
0143     end
0144 end
0145 
0146
```

---

Generated on Thu 11-Aug-2011 15:06:22 by **m2html** © 2005
