# Supplementary material for: TIGER: Toolbox for integrating genome-scale metabolic models, expression data, and transcriptional regulatory networks
Source: BMC Syst Biol. 2011 Sep 23;5:147. doi: 10.1186/1752-0509-5-147 (PMC3224351; doi:10.1186/1752-0509-5-147)
Supplement: Additional file 2 — TIGER source code. Source code, documentation, and tutorials are also available online at http://bme.virginia.edu/csbl/downloads/ or http://csbl.bitbucket.org/tiger. [file 1752-0509-5-147-S2.GZ › tiger/doc/m2html/tiger/elf/eva.html]

Description of eva


Home > tiger > elf > eva.m

# eva

## PURPOSE

**Enzyme variability analysis**

## SYNOPSIS

**function [minact,maxact] = eva(elf,varargin)**

## DESCRIPTION

```
 EVA  Enzyme variability analysis

   [MINACT,MAXACT] = EVA(ELF,...params...)

   Calculates the minimium (MINACT) and maximum (MAXACT) enzyme 
   activities at a specified optimal flux value.  Parameters are the same
   as for FVA.  If the parameter 'vars' is not specified, the default is
   ELF.genes.
```

## CROSS-REFERENCE INFORMATION

This function calls:

- fva Flux Variability Analysis
- struct2list Convert a structure to a parameter list

This function is called by:


## SOURCE CODE

```
0001 function [minact,maxact] = eva(elf,varargin)
0002 % EVA  Enzyme variability analysis
0003 %
0004 %   [MINACT,MAXACT] = EVA(ELF,...params...)
0005 %
0006 %   Calculates the minimium (MINACT) and maximum (MAXACT) enzyme
0007 %   activities at a specified optimal flux value.  Parameters are the same
0008 %   as for FVA.  If the parameter 'vars' is not specified, the default is
0009 %   ELF.genes.
0010 
0011 p = inputParser;
0012 p.addParamValue('vars',elf.genes);
0013 p.KeepUnmatched = true;
0014 p.parse(varargin{:});
0015 unmatched = struct2list(p.Unmatched);
0016 
0017 [minact,maxact] = fva(elf,'vars',p.Results.vars,unmatched{:});
```

---

Generated on Thu 11-Aug-2011 15:06:22 by **m2html** © 2005
