# Supplementary material for: TIGER: Toolbox for integrating genome-scale metabolic models, expression data, and transcriptional regulatory networks
Source: BMC Syst Biol. 2011 Sep 23;5:147. doi: 10.1186/1752-0509-5-147 (PMC3224351; doi:10.1186/1752-0509-5-147)
Supplement: Additional file 2 — TIGER source code. Source code, documentation, and tutorials are also available online at http://bme.virginia.edu/csbl/downloads/ or http://csbl.bitbucket.org/tiger. [file 1752-0509-5-147-S2.GZ › tiger/doc/m2html/tiger/elf/restore_rev_cons.html]

Description of restore\_rev\_cons


Home > tiger > elf > restore\_rev\_cons.m

# restore\_rev\_cons

## PURPOSE

**Restore reversibility constraints in an ELF model**

## SYNOPSIS

**function [elf] = restore\_rev\_cons(elf)**

## DESCRIPTION

```
 RESTORE_REV_CONS  Restore reversibility constraints in an ELF model
```

## CROSS-REFERENCE INFORMATION

This function calls:

- find\_like Find matches in a cell of strings

This function is called by:


## SOURCE CODE

```
0001 function [elf] = restore_rev_cons(elf)
0002 % RESTORE_REV_CONS  Restore reversibility constraints in an ELF model
0003 
0004 [~,locs] = find_like('^ELF_REV_CON',elf.rownames);
0005 elf.A(locs,:) = elf.rev_cons.A;
0006 elf.b(locs) = elf.rev_cons.b;
```

---

Generated on Thu 11-Aug-2011 15:06:22 by **m2html** © 2005
