# Supplementary material for: TIGER: Toolbox for integrating genome-scale metabolic models, expression data, and transcriptional regulatory networks
Source: BMC Syst Biol. 2011 Sep 23;5:147. doi: 10.1186/1752-0509-5-147 (PMC3224351; doi:10.1186/1752-0509-5-147)
Supplement: Additional file 2 — TIGER source code. Source code, documentation, and tutorials are also available online at http://bme.virginia.edu/csbl/downloads/ or http://csbl.bitbucket.org/tiger. [file 1752-0509-5-147-S2.GZ › tiger/doc/m2html/tiger/add_rule.html]

Description of add\_rule


Home > tiger > add\_rule.m

# add\_rule

## PURPOSE

**Add rules to a TIGER model**

## SYNOPSIS

**function [tiger] = add\_rule(tiger,rule,varargin)**

## DESCRIPTION

```
 ADD_RULE  Add rules to a TIGER model

   [TIGER] = ADD_RULE(TIGER,RULE,...params...)

   Convert rules to a MILP format and add them to a TIGER model.

   Inputs
   TIGER   TIGER model structure.  If empty, a new TIGER structure will
           be created.
   RULE    Rule or cell array of rules to be added.  Rules can be either
           a text string or an EXPR object (text strings will be parsed
           into EXPR objects).  If multiple rules are to be added to a 
           model, it is more efficient to call ADD_RULE once with a cell
           array of rules, rather than makeing repeated calls to ADD_RULE
           with single rules.

   Outputs
   TIGER   TIGER model structure with rules added.

   Parameters
   'default_lb'  Default lower bound for new variables found in the 
                 rules.  Default is 0.
   'default_ub'  Default upper bound for new variables found in the 
                 rules.  Default is 1.
   'bounds'      Cell specifing upper and lower bounds for new variables.
                 The first entry is a cell of variable names.  The second
                 and third entries are vectors containing the lower and
                 upper bounds for each variable name.  If a atom name is
                 found in the rules that is not listed, the default 
                 bounds are used.
   'not_type'    Type of NOT indicator to use for multilevel variables.
                 Options are
                     'inverted'  NOT x =  x_max - x  (default)
                     'binary'    NOT x =  1  if x > 0
                                          0  otherwise
   'ind_prefix'  String denoting the prefix used when creating indicator
                 variable names.  Default is 'I'.
   'ind_width'   Integer denoting the number of digits used to create
                 indicator variable names.  Extra places will be zero-
                 padded.  Default is 4.
   'not_prefix'  String denoting the prefix used when creating negated
                 variable names.  Default is 'NOT__'.
   'numeric'     If true, atoms resembling numeric constants are parsed
                 as such.  (default = true)
   'keep_rules'  If true (default), ADD_RULE remembers which rows were
                 created to compile each rule.  These associations are
                 stored in TIGER.param.rule_id, which if
                 TIGER.param.rule_id(i) = j, then row i was created to
                 parse the rule TIGER.param.rules{j}.
```

## CROSS-REFERENCE INFORMATION

This function calls:

- assert\_tiger Assert that a structure is an TIGER model.
- check\_tiger Check size and orientation of TIGER fields
- create\_empty\_tiger Create an empty TIGER model structure.
- expr
- parse\_string Parse a rule string into an EXPR object
- array2names Create a cell of names from an array of numbers
- assert\_cell Assert that variable is a cell array.
- map Generate a new list by applying a function

This function is called by:

- add\_diff Add difference variables toa TIGER model
- bind\_var Bind variables to a indicator variable
- convert\_gpr Add the GPR rules as constraints to the model.
- find\_infeasible\_rules Determine which rules make a model infeasible.
- test\_\_indicators
- test\_\_multilevel
- test\_\_remove\_rule
- convert\_rules
- create\_yeast\_trn\_model
- infeas\_study
- load\_rules
- run\_new\_rules
- imat Integrative Metabolic Analysis Tool
- update\_rule Re-compile rule(s) previously added to a TIGER model

## SUBFUNCTIONS

- function prepare\_conditional(cond)
- function switch\_nots(e)
- function switch\_aux(e)
- function add\_not\_con(not\_var,not\_ind)
- function simplify\_rule(r)
- function simplify\_expr(e)
- function [ind\_expr] = make\_substitution(e)
- function [lb,ub] = get\_expr\_bounds(e)
- function [ind\_name] = get\_next\_ind\_name()
- function add\_var(name,lb,ub)
- function simple\_rule\_to\_ineqs(r)
- function addrow(coefs,ctype,rhs,loc)
- function [tf] = is\_binary(e)
- function [tf] = is\_multilevel(e)

## SOURCE CODE

```
0001 function [tiger] = add_rule(tiger,rule,varargin)
0002 % ADD_RULE  Add rules to a TIGER model
0003 %
0004 %   [TIGER] = ADD_RULE(TIGER,RULE,...params...)
0005 %
0006 %   Convert rules to a MILP format and add them to a TIGER model.
0007 %
0008 %   Inputs
0009 %   TIGER   TIGER model structure.  If empty, a new TIGER structure will
0010 %           be created.
0011 %   RULE    Rule or cell array of rules to be added.  Rules can be either
0012 %           a text string or an EXPR object (text strings will be parsed
0013 %           into EXPR objects).  If multiple rules are to be added to a
0014 %           model, it is more efficient to call ADD_RULE once with a cell
0015 %           array of rules, rather than makeing repeated calls to ADD_RULE
0016 %           with single rules.
0017 %
0018 %   Outputs
0019 %   TIGER   TIGER model structure with rules added.
0020 %
0021 %   Parameters
0022 %   'default_lb'  Default lower bound for new variables found in the
0023 %                 rules.  Default is 0.
0024 %   'default_ub'  Default upper bound for new variables found in the
0025 %                 rules.  Default is 1.
0026 %   'bounds'      Cell specifing upper and lower bounds for new variables.
0027 %                 The first entry is a cell of variable names.  The second
0028 %                 and third entries are vectors containing the lower and
0029 %                 upper bounds for each variable name.  If a atom name is
0030 %                 found in the rules that is not listed, the default
0031 %                 bounds are used.
0032 %   'not_type'    Type of NOT indicator to use for multilevel variables.
0033 %                 Options are
0034 %                     'inverted'  NOT x =  x_max - x  (default)
0035 %                     'binary'    NOT x =  1  if x > 0
0036 %                                          0  otherwise
0037 %   'ind_prefix'  String denoting the prefix used when creating indicator
0038 %                 variable names.  Default is 'I'.
0039 %   'ind_width'   Integer denoting the number of digits used to create
0040 %                 indicator variable names.  Extra places will be zero-
0041 %                 padded.  Default is 4.
0042 %   'not_prefix'  String denoting the prefix used when creating negated
0043 %                 variable names.  Default is 'NOT__'.
0044 %   'numeric'     If true, atoms resembling numeric constants are parsed
0045 %                 as such.  (default = true)
0046 %   'keep_rules'  If true (default), ADD_RULE remembers which rows were
0047 %                 created to compile each rule.  These associations are
0048 %                 stored in TIGER.param.rule_id, which if
0049 %                 TIGER.param.rule_id(i) = j, then row i was created to
0050 %                 parse the rule TIGER.param.rules{j}.
0051 
0052 assert(nargin >= 2, 'ADD_RULE requires at least two inputs.');
0053 
0054 % if there is no starting model, start with a blank model
0055 if isempty(tiger)
0056     tiger = create_empty_tiger();
0057 end
0058 
0059 % check that a TIGER model was given (and convert if COBRA)
0060 tiger = assert_tiger(tiger);
0061 
0062 % begin parameter checking
0063 p = inputParser;
0064 
0065 p.addParamValue('ind_prefix','I',@ischar);
0066 ind_width_val = @(x) validateattributes(x,{'numeric'}, ...
0067                                         {'scalar','integer','<=',1});
0068 p.addParamValue('ind_width',4,ind_width_val);
0069 p.addParamValue('not_prefix','NOT__',@ischar);
0070 
0071 bound_val = @(x) validateattributes(x,{'numeric'},{'scalar','real'});
0072 p.addParamValue('default_lb',0,bound_val);
0073 p.addParamValue('default_ub',1,bound_val);
0074 
0075 p.addParamValue('bounds',[]);
0076 
0077 valid_not_type = @(x) ismember(x,{'pseudo-binary','inverted'});
0078 p.addParamValue('not_type','inverted',valid_not_type);
0079 
0080 p.addParamValue('numeric',true);
0081 
0082 p.addParamValue('keep_rules',true);
0083 
0084 p.parse(varargin{:});
0085 
0086 IND_PRE = p.Results.ind_prefix;
0087 IND_WIDTH = p.Results.ind_width;  % number of digits for indicator names
0088 ind_counter = tiger.param.ind;
0089 
0090 NOT_PRE = p.Results.not_prefix;
0091 not_type = p.Results.not_type;
0092 
0093 default_lb = p.Results.default_lb;
0094 default_ub = p.Results.default_ub;
0095 
0096 user_bounds = p.Results.bounds;  % TODO: add support for user bounds
0097 
0098 parse_numeric = p.Results.numeric;
0099 
0100 keep_rules = p.Results.keep_rules;
0101 
0102 % rule parsing
0103 rules = assert_cell(parse_string(rule,parse_numeric));
0104 N = length(rules);
0105 
0106 % Get all atoms in the expression list.  This is repeated in
0107 % simplify_rule, but do it on all atoms here for efficiency.
0108 atoms = cellfun(@(x) x.atoms,rules,'Uniform',false);
0109 atoms = setdiff(unique([atoms{:}]),tiger.varnames);
0110 add_var(atoms);
0111 
0112 % measure the size of the original model
0113 [orig_m,orig_n] = size(tiger.A);
0114 
0115 % TODO: pre-allocate A better
0116 A = tiger.A;
0117 b = tiger.b;
0118 ctypes = tiger.ctypes;
0119 ind = tiger.ind;
0120 indtypes = tiger.indtypes;
0121 roff = size(A,1);  % row offset for adding constraints
0122 
0123 if keep_rules
0124     tiger.param.rules(end+(1:N)) = map(@(x) x.copy,rules);
0125 end
0126 
0127 % simplify the rules and convert to inequalities
0128 for i = 1 : N
0129     if keep_rules
0130         current_rule_id = i;
0131     else
0132         current_rule_id = 0;
0133     end
0134     
0135     simplify_rule(rules{i});
0136 end
0137 
0138 % add new entries to the TIGER model
0139 Nvars_added = size(A,2) - orig_n;
0140 tiger.A = A;
0141 tiger.b = b;
0142 tiger.ctypes = ctypes;
0143 rownames = array2names('ROW%i',orig_m+1:size(A,1));
0144 tiger.rownames = [tiger.rownames; rownames];
0145 tiger.obj = [tiger.obj; zeros(Nvars_added,1)];
0146 tiger.ind = ind;
0147 tiger.indtypes = indtypes;
0148 
0149 tiger.param.ind = ind_counter;
0150 
0151 tiger = check_tiger(tiger);
0152 
0153 function prepare_conditional(cond)
0154     % Remove '>', '<', and '~=' operators
0155     switch cond.cond_op
0156         case '>'
0157             cond.cond_op = '<=';
0158             cond.negated = ~cond.negated;
0159         case '<'
0160             cond.cond_op = '>=';
0161             cond.negated = ~cond.negated;
0162         case '~='
0163             cond.cond_op = '=';
0164             cond.negated = ~cond.negated;
0165     end
0166     
0167     if strcmp(cond.cond_op,'=')
0168         % switch (a = b) to (a >= b) AND (a <= b) to avoid indicators
0169         % on equality constraints
0170         lexpr = cond.copy;
0171         lexpr.cond_op = '>=';
0172         rexpr = cond.copy;
0173         rexpr.cond_op = '<=';
0174         
0175         cond.cond_op = '';
0176         cond.AND = true;
0177         cond.lexpr = lexpr;
0178         cond.rexpr = rexpr;
0179         
0180         cond.demorgan();
0181     end
0182 end
0183             
0184 function switch_nots(e)
0185     % Create negated variables to remove negated atoms.
0186     e.iterif(@(x) x.is_atom && x.negated,@switch_aux);
0187     
0188     function switch_aux(e)
0189         not_name = [NOT_PRE e.id];
0190         add_not_con(e.id,not_name);
0191         e.id = not_name;
0192         e.negated = false;
0193     end
0194 end
0195 
0196 function add_not_con(not_var,not_ind)
0197     if strcmpi(not_type,'binary')
0198         pseudo_rule = sprintf('%s > 0 <=> %s',not_var,not_ind);
0199         simplify_rule(parse_string(pseudo_rule));
0200     else
0201         [~,var_idx] = ismember(not_var,tiger.varnames);
0202         tf = ismember(not_ind,tiger.varnames);
0203         if ~tf
0204             add_var(not_ind,tiger.lb(var_idx),tiger.ub(var_idx));
0205             ind_idx = length(tiger.varnames);
0206             roff = roff + 1;
0207             A(roff,[var_idx,ind_idx]) = [1 1];
0208             b(roff) = tiger.ub(var_idx);
0209             ctypes(roff) = '=';
0210         end
0211     end
0212 end
0213 
0214 function simplify_rule(r)
0215     % Simplify a rule.  The resulting
0216     % rules are of the form:
0217     %                atom -> atom
0218     %                cond -> atom
0219     %       atom AND atom -> atom
0220     %       atom OR  atom -> atom
0221     % The simple rules are converted to inequalities.
0222     
0223     atoms = r.atoms;
0224     tf = ismember(atoms,tiger.varnames);
0225     add_var(atoms(~tf));
0226     
0227     % move nots down to the atoms
0228     r.demorgan();
0229     
0230     % prepare conditionals
0231     r.iterif(@(e) e.is_cond,@prepare_conditional);
0232 
0233     if ~r.rexpr.is_atom
0234         r.rexpr = make_substitution(r.rexpr);
0235     end
0236     if ~r.lexpr.is_simple
0237         simplify_expr(r.lexpr);
0238     end
0239     
0240     switch_nots(r);
0241         
0242     % convert to ineqs
0243     simple_rule_to_ineqs(r);
0244 end
0245 
0246 function simplify_expr(e)
0247     % Simplifies an expression.  If the left or right subexpressions
0248     % are not atoms, they are replaced with an indicator variable.
0249     % This function modifies the expression in place.
0250     if e.is_cond
0251         % cannot replace entire expression directly; must copy manually
0252         new_ind = make_substitution(e);
0253         e.cond_op = '';
0254         e.id = new_ind.id;
0255         e.negated = new_ind.negated;
0256         e.lexpr = [];
0257         e.rexpr = [];
0258         return;
0259     end
0260     if ~e.lexpr.is_atom
0261         e.lexpr = make_substitution(e.lexpr);
0262     end
0263     if ~e.rexpr.is_atom
0264         e.rexpr = make_substitution(e.rexpr);
0265     end
0266 end
0267 
0268 function [ind_expr] = make_substitution(e)
0269     % Returns an indicator to replace the expression 'e'.
0270     % 'e' is formed into a rule with the indicator.  This rule
0271     % is passed to 'simplify_rule' to be added to the list of
0272     % simple rules.  The indicator is added to the list of
0273     % variable names in the TIGER structure.
0274     %
0275     % The indicator expression returned is not linked to the
0276     % new rule (a copy is made in this function).  The expression
0277     % passed in is copied as well, so the expression in the parent
0278     % rule can be modified in place.
0279     ind_name = get_next_ind_name();
0280     ind_expr = expr();
0281     ind_expr.id = ind_name;
0282 
0283     ind_rule = expr();
0284     ind_rule.IFF = true;
0285     ind_rule.lexpr = e.copy;
0286     ind_rule.rexpr = ind_expr.copy;
0287     
0288     % leave the negation on the indicator
0289     if e.negated
0290         ind_expr.negated = true;
0291         ind_rule.lexpr.negated = false;
0292     end
0293     
0294     [ind_lb,ind_ub] = get_expr_bounds(e);
0295     add_var(ind_name,ind_lb,ind_ub);
0296     
0297     simplify_rule(ind_rule);
0298 end
0299 
0300 function [lb,ub] = get_expr_bounds(e)
0301     % Get upper and lower bounds on an expression
0302     % TODO  tighten bounds on AND and OR
0303     if e.is_numeric
0304         lb = 0;
0305         ub = 0;
0306     elseif e.is_atom
0307         [~,loc] = ismember(e.id,tiger.varnames);
0308         lb = tiger.lb(loc);
0309         ub = tiger.ub(loc);
0310     elseif e.is_cond
0311         % conditionals use binary indicators
0312         lb = 0;
0313         ub = 1;
0314     else
0315         [llb,lub] = get_expr_bounds(e.lexpr);
0316         [rlb,rub] = get_expr_bounds(e.rexpr);
0317         lb = min([llb rlb]);
0318         ub = max([lub rub]);
0319     end
0320 end
0321 
0322 function [ind_name] = get_next_ind_name()
0323     % Get the next indicator name
0324     ind_counter = ind_counter + 1;
0325     ind_name = sprintf([IND_PRE '%0*i'], IND_WIDTH, ind_counter);
0326 end
0327 
0328 function add_var(name,lb,ub)
0329     % Add a variable to VARNAMES and place correct bounds and vartype.
0330     % If LB and UB are not given, default bounds or user-specified bounds
0331     % are used.
0332     names = assert_cell(name);
0333     Nnames = length(names);
0334     if nargin < 2 || isempty(lb)
0335         lbs = repmat(default_lb,1,Nnames);
0336     else
0337         if length(lb) == 1
0338             lbs = repmat(lb,1,Nnames);
0339         else
0340             lbs = lb;
0341         end
0342     end
0343     if nargin < 3 || isempty(ub)
0344         ubs = repmat(default_ub,1,Nnames);
0345     else
0346         if length(ub) == 1
0347             ubs = repmat(ub,1,Nnames);
0348         else
0349             ubs = ub;
0350         end
0351     end
0352         
0353     % if user specified bounds, change from default
0354     if ~isempty(user_bounds)
0355         [tf,loc] = ismember(user_bounds{1},names);
0356         if any(tf)
0357             lbs(loc(tf)) = user_bounds{2}(tf);
0358             ubs(loc(tf)) = user_bounds{3}(tf);
0359         end
0360     end
0361     
0362     tiger.varnames(end+1:end+Nnames) = names;
0363     tiger.lb(end+1:end+Nnames) = lbs;
0364     tiger.ub(end+1:end+Nnames) = ubs;
0365     
0366     vartypes = repmat('b',Nnames,1);
0367     vartypes(ubs > 1) = 'i';
0368     tiger.vartypes(end+1:end+Nnames) = vartypes;
0369 end
0370 
0371 function simple_rule_to_ineqs(r)
0372     e = r.lexpr;
0373     I = r.rexpr.id;
0374     [~,Iloc] = ismember(I,tiger.varnames);
0375     
0376     if e.is_atom
0377         [~,loc] = ismember(e.id,tiger.varnames);
0378         if r.IFF
0379             % x <=> I ~> x = I
0380             addrow([1 -1],'=',0,[loc Iloc]);
0381         else
0382             % x => I ~> I >= x
0383             addrow([1 -1],'<',0,[loc Iloc]);
0384         end
0385         return;
0386     end
0387     
0388     if e.is_cond
0389         assert(ismember(e.cond_op,{'<=','=','>='}), ...
0390                'Operator %s should have been removed.',e.cond_op);
0391         lname = e.lexpr.id;
0392         [~,lloc] = ismember(lname,tiger.varnames);
0393         rname = e.rexpr.id;
0394         [~,rloc] = ismember(rname,tiger.varnames);
0395         op = e.cond_op;
0396         if e.rexpr.is_numeric
0397             addrow(1,op(1),str2double(rname),lloc);
0398         else
0399             addrow([1 -1],op(1),0,[lloc rloc]);
0400         end
0401         ind(roff) = Iloc;
0402         if r.IFF
0403             indtypes(roff) = 'b';
0404         else
0405             indtypes(roff) = 'p';
0406         end
0407         
0408         return;
0409     end
0410     
0411     x = r.lexpr.lexpr.id;
0412     y = r.lexpr.rexpr.id;
0413     [~,xloc] = ismember(x,tiger.varnames);
0414     [~,yloc] = ismember(y,tiger.varnames);
0415     locs = [xloc yloc Iloc];
0416     
0417     multilevel = is_multilevel(r.lexpr);
0418     
0419     xmax = tiger.ub(xloc);
0420     xmin = tiger.lb(xloc);
0421     ymax = tiger.ub(yloc);
0422     ymin = tiger.lb(yloc);
0423     
0424     xrange = xmax - xmin;
0425     yrange = ymax - ymin;
0426     
0427     if ~multilevel && e.AND
0428         addrow([2 2 -4],'<',3);
0429         if r.IFF
0430             addrow([2 2 -4],'>',-1);
0431         end
0432     elseif ~multilevel && e.OR
0433         addrow([-1 -1 3],'>',0);
0434         if r.IFF
0435             addrow([-1 -1 3],'<',2);
0436         end
0437     elseif e.is_junc
0438         % multilevel expressions
0439         if e.AND
0440             Iaux = get_next_ind_name();
0441             I_exp = parse_string(sprintf('%s > %s <=> %s',x,y,Iaux));
0442             simplify_rule(I_exp);
0443             [~,Iaux_loc] = ismember(Iaux,tiger.varnames);
0444             addrow([1  xrange -1],'<',     0,[xloc Iaux_loc Iloc]);
0445             addrow([1 -yrange -1],'<',yrange,[yloc Iaux_loc Iloc]);
0446             if r.IFF
0447                 addrow([1 -1],'<',0,[Iloc xloc]);
0448                 addrow([1 -1],'<',0,[Iloc yloc]);
0449             end
0450         elseif e.OR
0451             addrow([1 -1],'>',0,[Iloc xloc]);
0452             addrow([1 -1],'>',0,[Iloc yloc]);
0453             if r.IFF
0454                 Iaux = get_next_ind_name();
0455                 I_exp = parse_string(sprintf('%s > %s <=> %s',x,y,Iaux));
0456                 simplify_rule(I_exp);
0457                 [~,Iaux_loc] = ismember(Iaux,tiger.varnames);
0458                 addrow([1 -xrange -1],'>',-xrange,[xloc Iaux_loc Iloc]);
0459                 addrow([1  yrange -1],'>',      0,[yloc Iaux_loc Iloc]);
0460             end
0461         end
0462     end
0463     
0464     function addrow(coefs,ctype,rhs,loc)
0465         if nargin < 4
0466             loc = locs;
0467         end
0468         roff = roff + 1;
0469         A(roff,loc) = coefs;
0470         b(roff) = rhs;
0471         ctypes(roff) = ctype;
0472         ind(roff) = 0;
0473         indtypes(roff) = ' ';
0474         
0475         % assign the row to a rule
0476         tiger.param.rule_id(roff) = current_rule_id;
0477     end   
0478 end
0479 
0480 function [tf] = is_binary(e)
0481     if e.is_atom
0482         [~,loc] = ismember(e.id,tiger.varnames);
0483         tf = tiger.vartypes(loc) == 'b';
0484     elseif e.is_cond
0485         tf = true;
0486     elseif e.is_junc
0487         tf = is_binary(e.lexpr) && is_binary(e.rexpr);
0488     end
0489 end
0490 
0491 function [tf] = is_multilevel(e)
0492     tf = ~is_binary(e);
0493 end
0494 
0495 end % add_rule
0496
```

---

Generated on Thu 11-Aug-2011 15:06:22 by **m2html** © 2005
