# Supplementary material for: TIGER: Toolbox for integrating genome-scale metabolic models, expression data, and transcriptional regulatory networks
Source: BMC Syst Biol. 2011 Sep 23;5:147. doi: 10.1186/1752-0509-5-147 (PMC3224351; doi:10.1186/1752-0509-5-147)
Supplement: Additional file 2 — TIGER source code. Source code, documentation, and tutorials are also available online at http://bme.virginia.edu/csbl/downloads/ or http://csbl.bitbucket.org/tiger. [file 1752-0509-5-147-S2.GZ › tiger/doc/m2html/tiger/open_bounds.html]

Description of open\_bounds


Home > tiger > open\_bounds.m

# open\_bounds

## PURPOSE

**Open all bounds to a max value**

## SYNOPSIS

**function [tiger,bounds] = open\_bounds(tiger)**

## DESCRIPTION

```
 OPEN_BOUNDS  Open all bounds to a max value

   [TIGER,BOUNDS] = OPEN_BOUNDS(TIGER)

   Converts all lower and upper bounds to the minimum and maximum bounds
   in the respective field.  Returns the modified models and a BOUNDS
   structure that can be used to reset the bounds to their original
   values (see CLOSE_BOUNDS).
```

## CROSS-REFERENCE INFORMATION

This function calls:


This function is called by:

- create\_yeast\_trn\_model

## SOURCE CODE

```
0001 function [tiger,bounds] = open_bounds(tiger)
0002 % OPEN_BOUNDS  Open all bounds to a max value
0003 %
0004 %   [TIGER,BOUNDS] = OPEN_BOUNDS(TIGER)
0005 %
0006 %   Converts all lower and upper bounds to the minimum and maximum bounds
0007 %   in the respective field.  Returns the modified models and a BOUNDS
0008 %   structure that can be used to reset the bounds to their original
0009 %   values (see CLOSE_BOUNDS).
0010 
0011 bounds.lb = tiger.lb;
0012 bounds.ub = tiger.ub;
0013 bounds.N = length(tiger.lb);
0014 
0015 tiger.lb(:) = min(tiger.lb);
0016 tiger.ub(:) = max(tiger.ub);
```

---

Generated on Thu 11-Aug-2011 15:06:22 by **m2html** © 2005
