# Supplementary material for: TIGER: Toolbox for integrating genome-scale metabolic models, expression data, and transcriptional regulatory networks
Source: BMC Syst Biol. 2011 Sep 23;5:147. doi: 10.1186/1752-0509-5-147 (PMC3224351; doi:10.1186/1752-0509-5-147)
Supplement: Additional file 2 — TIGER source code. Source code, documentation, and tutorials are also available online at http://bme.virginia.edu/csbl/downloads/ or http://csbl.bitbucket.org/tiger. [file 1752-0509-5-147-S2.GZ › tiger/doc/m2html/tiger/create_empty_tiger.html]

Description of create\_empty\_tiger


Home > tiger > create\_empty\_tiger.m

# create\_empty\_tiger

## PURPOSE

**Create an empty TIGER model structure.**

## SYNOPSIS

**function [tiger] = create\_empty\_tiger()**

## DESCRIPTION

```
 CREATE_EMPTY_TIGER  Create an empty TIGER model structure.
```

## CROSS-REFERENCE INFORMATION

This function calls:


This function is called by:

- add\_rule Add rules to a TIGER model
- assert\_tiger Assert that a structure is an TIGER model.
- cobra\_to\_tiger Convert a COBRA model to a TIGER model
- test\_\_add\_diff
- test\_\_diffadj
- test\_\_miqp
- test\_\_multilevel
- test\_\_remove\_rule

## SOURCE CODE

```
0001 function [tiger] = create_empty_tiger()
0002 % CREATE_EMPTY_TIGER  Create an empty TIGER model structure.
0003 
0004 tiger.A = [];
0005 tiger.b = [];
0006 tiger.lb = [];
0007 tiger.ub = [];
0008 tiger.obj = [];
0009 
0010 tiger.varnames = {};
0011 tiger.rownames = {};
0012 
0013 tiger.ctypes = '';
0014 tiger.vartypes = '';
0015 
0016 tiger.ind = [];
0017 tiger.indtypes = '';
0018 
0019 tiger.param.ind = 0;
0020 tiger.param.fixedvar = [];
0021 tiger.param.rules = {};
0022 tiger.param.rule_id = [];
```

---

Generated on Thu 11-Aug-2011 15:06:22 by **m2html** © 2005
