# Supplementary material for: TIGER: Toolbox for integrating genome-scale metabolic models, expression data, and transcriptional regulatory networks
Source: BMC Syst Biol. 2011 Sep 23;5:147. doi: 10.1186/1752-0509-5-147 (PMC3224351; doi:10.1186/1752-0509-5-147)
Supplement: Additional file 2 — TIGER source code. Source code, documentation, and tutorials are also available online at http://bme.virginia.edu/csbl/downloads/ or http://csbl.bitbucket.org/tiger. [file 1752-0509-5-147-S2.GZ › tiger/doc/m2html/tiger/single_gene_ko.html]

Description of single\_gene\_ko


Home > tiger > single\_gene\_ko.m

# single\_gene\_ko

## PURPOSE

**Perform single gene knockout simulations**

## SYNOPSIS

**function [grRatio,grRateKO,grRateWT] = single\_gene\_ko(tiger,genes)**

## DESCRIPTION

```
 SINGLE_GENE_KO  Perform single gene knockout simulations

   [grRatio,grRateKO,grRateWT] = SINGLE_GENE_KO(TIGER,GENES)

   Performs knockouts of each gene in the cell GENES.  If a GENES is not
   given, uses all genes in the cell TIGER.genes.

   Outputs
   grRatio   Ratio of knockout and wild-type growth rates.
   grRateKO  Growth rate for each knockout.
   grRateWT  Wild-type growth rate.
```

## CROSS-REFERENCE INFORMATION

This function calls:

- convert\_ids Create name, indices, and logical indices from an array
- fba Run Flux Balance Analysis on a TIGER model.
- statusbar

This function is called by:


## SOURCE CODE

```
0001 function [grRatio,grRateKO,grRateWT] = single_gene_ko(tiger,genes)
0002 % SINGLE_GENE_KO  Perform single gene knockout simulations
0003 %
0004 %   [grRatio,grRateKO,grRateWT] = SINGLE_GENE_KO(TIGER,GENES)
0005 %
0006 %   Performs knockouts of each gene in the cell GENES.  If a GENES is not
0007 %   given, uses all genes in the cell TIGER.genes.
0008 %
0009 %   Outputs
0010 %   grRatio   Ratio of knockout and wild-type growth rates.
0011 %   grRateKO  Growth rate for each knockout.
0012 %   grRateWT  Wild-type growth rate.
0013 
0014 if nargin < 2
0015     genes = tiger.genes;
0016 end
0017 
0018 idxs = convert_ids(tiger.varnames,genes,'index');
0019 
0020 N = length(genes);
0021 statbar = statusbar(N);
0022 
0023 grRateKO = zeros(N,1);
0024 
0025 sol = fba(tiger);
0026 grRateWT = sol.val;
0027 
0028 statbar.start('Single Gene Deletion status');
0029 for i = 1 : N
0030     m = tiger;
0031     m.ub(idxs(i)) = 0;
0032     sol = fba(m);
0033     if isempty(sol.val)
0034         % infeasible; assume lethal
0035         sol.val = 0;
0036     end
0037     grRateKO(i) = sol.val;
0038     statbar.update(i);
0039 end
0040 
0041 grRatio = grRateKO / grRateWT;
```

---

Generated on Thu 11-Aug-2011 15:06:22 by **m2html** © 2005
