# Supplementary material for: TIGER: Toolbox for integrating genome-scale metabolic models, expression data, and transcriptional regulatory networks
Source: BMC Syst Biol. 2011 Sep 23;5:147. doi: 10.1186/1752-0509-5-147 (PMC3224351; doi:10.1186/1752-0509-5-147)
Supplement: Additional file 2 — TIGER source code. Source code, documentation, and tutorials are also available online at http://bme.virginia.edu/csbl/downloads/ or http://csbl.bitbucket.org/tiger. [file 1752-0509-5-147-S2.GZ › tiger/doc/m2html/tiger/test/tutorial_model.html]

Description of tutorial\_model


Home > tiger > test > tutorial\_model.m

# tutorial\_model

## PURPOSE

**r2 r3 r4 r5**

## SYNOPSIS

**This is a script file.**

## DESCRIPTION

```
          r1 r2 r3 r4 r5
```

## CROSS-REFERENCE INFORMATION

This function calls:


This function is called by:


## SOURCE CODE

```
0001 
0002 %          r1 r2 r3 r4 r5
0003 cobra.S = [ 1 -1  0  0  0;  % A
0004             0  1  0 -1  0;  % B
0005             0  0  1 -1  0;  % C
0006             0  0  0  1 -1]; % D
0007 
0008 cobra.c = [ 0  0  0  0  1]';
0009 cobra.lb = -10*ones(size(cobra.c));
0010 cobra.ub =  10*ones(size(cobra.c));
0011 
0012 cobra.b = zeros(size(cobra.S,1),1);
0013         
0014 cobra.rxns = {'rxn1';'rxn2';'rxn3';'rxn4';'rxn5'};
0015 cobra.mets = {'A';'B';'C';'D'};
0016 
0017 cobra.genes = {'AB1';'AB2';'BCD1';'BCD2'};
0018 cobra.grRules = {'';'AB1 or AB2';'';'BCD1 and BCD2';''};
```

---

Generated on Thu 11-Aug-2011 15:06:22 by **m2html** © 2005
