# Supplementary material for: TIGER: Toolbox for integrating genome-scale metabolic models, expression data, and transcriptional regulatory networks
Source: BMC Syst Biol. 2011 Sep 23;5:147. doi: 10.1186/1752-0509-5-147 (PMC3224351; doi:10.1186/1752-0509-5-147)
Supplement: Additional file 2 — TIGER source code. Source code, documentation, and tutorials are also available online at http://bme.virginia.edu/csbl/downloads/ or http://csbl.bitbucket.org/tiger. [file 1752-0509-5-147-S2.GZ › tiger/doc/m2html/tiger/test/unit/test_tiger.html]

Description of test\_tiger


Home > tiger > test > unit > test\_tiger.m

# test\_tiger

## PURPOSE

**Run unit tests on the TIGER package**

## SYNOPSIS

**function test\_tiger()**

## DESCRIPTION

```
 TEST_TIGER  Run unit tests on the TIGER package

   Before running this function, move to the TIGER/test/unit directory.
   Unit tests are located in TIGER/test/unit/tests.
```

## CROSS-REFERENCE INFORMATION

This function calls:

- show\_padded Show padded strings

This function is called by:


## SOURCE CODE

```
0001 function test_tiger()
0002 % TEST_TIGER  Run unit tests on the TIGER package
0003 %
0004 %   Before running this function, move to the TIGER/test/unit directory.
0005 %   Unit tests are located in TIGER/test/unit/tests.
0006 
0007 home;
0008 fprintf('\nTIGER unit testing:\n\n');
0009 
0010 PREFIX = 'test__';
0011 
0012 files = dir('tests');
0013 to_run = {};
0014 for f = 1 : length(files)
0015     if length(files(f).name) > length(PREFIX) ...
0016         && strcmp(files(f).name(1:length(PREFIX)),PREFIX) ...
0017         && strcmp(files(f).name(end-1:end),'.m')
0018         to_run{end+1} = files(f).name(1:end-2);
0019     end
0020 end
0021 
0022 error_count = 0;
0023 error_messages = {};
0024 
0025 cd('tests');
0026 for file = 1 : length(to_run)
0027     prev_error_count = error_count;
0028     try
0029         eval(to_run{file});
0030     catch ME
0031         error_count = error_count + 1;
0032         error_messages{end+1} = ME.message;
0033     end
0034     Nerror = error_count - prev_error_count;
0035     if Nerror == 0
0036         show_padded(['Running ' to_run{file} '.m'],'ok');
0037     else
0038         show_padded(['Running ' to_run{file} '.m'],'FAIL');
0039         disp('   Error(s):');
0040         for errorj = prev_error_count+1 : error_count
0041             disp(['      ',error_messages{errorj}]);
0042         end
0043     end
0044 end
0045 cd('..');
0046 
0047 if error_count == 0
0048     fprintf('\nTesting completed with no errors.\n\n');
0049 else
0050     fprintf('\nTesting FAILED with %i error(s).\n\n',error_count);
0051 end
0052
```

---

Generated on Thu 11-Aug-2011 15:06:22 by **m2html** © 2005
