# Supplementary material for: TIGER: Toolbox for integrating genome-scale metabolic models, expression data, and transcriptional regulatory networks
Source: BMC Syst Biol. 2011 Sep 23;5:147. doi: 10.1186/1752-0509-5-147 (PMC3224351; doi:10.1186/1752-0509-5-147)
Supplement: Additional file 2 — TIGER source code. Source code, documentation, and tutorials are also available online at http://bme.virginia.edu/csbl/downloads/ or http://csbl.bitbucket.org/tiger. [file 1752-0509-5-147-S2.GZ › tiger/doc/m2html/tiger/test/unit/tests/test__indicators.html]

Description of test\_\_indicators


Home > tiger > test > unit > tests > test\_\_indicators.m

# test\_\_indicators

## PURPOSE

## SYNOPSIS

**This is a script file.**

## DESCRIPTION

## CROSS-REFERENCE INFORMATION

This function calls:

- add\_rule Add rules to a TIGER model
- cobra\_to\_tiger Convert a COBRA model to a TIGER model
- fba Run Flux Balance Analysis on a TIGER model.
- cobra\_model Test model in COBRA format
- init\_test
- near Test if two values are close to each other

This function is called by:


## SOURCE CODE

```
0001 
0002 init_test
0003 
0004 cobra_model
0005 
0006 tiger = cobra_to_tiger(cobra);
0007 sol1 = fba(tiger);
0008 
0009 t = add_rule(tiger,'r1 < -0.5 <=> not g5a');
0010 sol2 = fba(t);
0011 
0012 assert(near(sol2.val,0.5),'indicator did not work');
```

---

Generated on Thu 11-Aug-2011 15:06:22 by **m2html** © 2005
