# Supplementary material for: TIGER: Toolbox for integrating genome-scale metabolic models, expression data, and transcriptional regulatory networks
Source: BMC Syst Biol. 2011 Sep 23;5:147. doi: 10.1186/1752-0509-5-147 (PMC3224351; doi:10.1186/1752-0509-5-147)
Supplement: Additional file 2 — TIGER source code. Source code, documentation, and tutorials are also available online at http://bme.virginia.edu/csbl/downloads/ or http://csbl.bitbucket.org/tiger. [file 1752-0509-5-147-S2.GZ › tiger/doc/m2html/tiger/test/unit/tests/test__imat.html]

Description of test\_\_imat


Home > tiger > test > unit > tests > test\_\_imat.m

# test\_\_imat

## PURPOSE

## SYNOPSIS

**This is a script file.**

## DESCRIPTION

## CROSS-REFERENCE INFORMATION

This function calls:

- cobra\_to\_tiger Convert a COBRA model to a TIGER model
- cobra\_model Test model in COBRA format
- init\_test
- imat Integrative Metabolic Analysis Tool
- near Test if two values are close to each other

This function is called by:


## SOURCE CODE

```
0001 
0002 init_test
0003 cobra_model
0004 
0005 levels     = [    2    1     0     0];
0006 gene_names = {'g7a','g7b','g6','g5a'};
0007 weights    = [    4    1     3     1];
0008 
0009 tiger = cobra_to_tiger(cobra);
0010 [levels,genes,sol,t] = imat(tiger,levels,'gene_names',gene_names, ...
0011                                          'flux_eps',0.3, ...
0012                                          'weights',weights);
0013 
0014 assert(near(levels,[2 2 0 1]),'levels incorrect');
```

---

Generated on Thu 11-Aug-2011 15:06:22 by **m2html** © 2005
