# Supplementary material for: TIGER: Toolbox for integrating genome-scale metabolic models, expression data, and transcriptional regulatory networks
Source: BMC Syst Biol. 2011 Sep 23;5:147. doi: 10.1186/1752-0509-5-147 (PMC3224351; doi:10.1186/1752-0509-5-147)
Supplement: Additional file 2 — TIGER source code. Source code, documentation, and tutorials are also available online at http://bme.virginia.edu/csbl/downloads/ or http://csbl.bitbucket.org/tiger. [file 1752-0509-5-147-S2.GZ › tiger/doc/m2html/tiger/test/unit/tests/index.html]

Index for Directory tiger/test/unit/tests


|  |  |
| --- | --- |
| Master index | Index for tiger/test/unit/tests |

# Index for tiger/test/unit/tests

## Matlab files in this directory:

|  |  |
| --- | --- |
| cobra\_model | Test model in COBRA format |
| init\_test |  |
| test\_\_add\_diff |  |
| test\_\_create\_table |  |
| test\_\_diffadj |  |
| test\_\_fba |  |
| test\_\_find\_optimal\_states |  |
| test\_\_gimme |  |
| test\_\_imat |  |
| test\_\_indicators |  |
| test\_\_made |  |
| test\_\_miqp |  |
| test\_\_multilevel |  |
| test\_\_remove\_rule |  |
| test\_\_solve\_multiple\_mips |  |
| test\_\_tile\_mip |  |

---

Generated on Thu 11-Aug-2011 15:06:20 by **m2html** © 2005
