# Supplementary material for: TIGER: Toolbox for integrating genome-scale metabolic models, expression data, and transcriptional regulatory networks
Source: BMC Syst Biol. 2011 Sep 23;5:147. doi: 10.1186/1752-0509-5-147 (PMC3224351; doi:10.1186/1752-0509-5-147)
Supplement: Additional file 2 — TIGER source code. Source code, documentation, and tutorials are also available online at http://bme.virginia.edu/csbl/downloads/ or http://csbl.bitbucket.org/tiger. [file 1752-0509-5-147-S2.GZ › tiger/doc/m2html/tiger/test/unit/tests/init_test.html]

Description of init\_test


Home > tiger > test > unit > tests > init\_test.m

# init\_test

## PURPOSE

## SYNOPSIS

**This is a script file.**

## DESCRIPTION

## CROSS-REFERENCE INFORMATION

This function calls:


This function is called by:

- test\_\_add\_diff
- test\_\_diffadj
- test\_\_fba
- test\_\_gimme
- test\_\_imat
- test\_\_indicators
- test\_\_miqp
- test\_\_multilevel
- test\_\_remove\_rule
- test\_\_solve\_multiple\_mips

## SOURCE CODE

```
0001 
0002 cmpi.init();
0003 cmpi.set_solver('cplex');
```

---

Generated on Thu 11-Aug-2011 15:06:22 by **m2html** © 2005
