# Supplementary material for: TIGER: Toolbox for integrating genome-scale metabolic models, expression data, and transcriptional regulatory networks
Source: BMC Syst Biol. 2011 Sep 23;5:147. doi: 10.1186/1752-0509-5-147 (PMC3224351; doi:10.1186/1752-0509-5-147)
Supplement: Additional file 2 — TIGER source code. Source code, documentation, and tutorials are also available online at http://bme.virginia.edu/csbl/downloads/ or http://csbl.bitbucket.org/tiger. [file 1752-0509-5-147-S2.GZ › tiger/doc/m2html/tiger/test/unit/tests/test__miqp.html]

Description of test\_\_miqp


Home > tiger > test > unit > tests > test\_\_miqp.m

# test\_\_miqp

## PURPOSE

## SYNOPSIS

**This is a script file.**

## DESCRIPTION

## CROSS-REFERENCE INFORMATION

This function calls:

- add\_column Add a column to a TIGER model structure
- add\_row Add a row to a TIGER model structure
- create\_empty\_tiger Create an empty TIGER model structure.
- init\_test
- near Test if two values are close to each other

This function is called by:


## SOURCE CODE

```
0001 
0002 init_test
0003 
0004 mip.obj = [-2 -6]';
0005 mip.A = sparse([1 1; -1 2; 2 1]);
0006 mip.b = [2;2;3];
0007 mip.lb = [0 0]';
0008 mip.ub = [100 100]';
0009 
0010 mip.ctypes = '<<<';
0011 mip.vartypes = 'cc';
0012 
0013 mip.Q = [0.5 0; 0 1];
0014 
0015 sol = cmpi.solve_mip(mip);
0016 assert(near(sol.val,-25/3),'MIQP Q obj val');
0017 
0018 t = create_empty_tiger();
0019 t = add_column(t,3,'c');
0020 t = add_row(t,[1 1 1],'<',1);
0021 
0022 t.Qc.w = [1 1 1];
0023 t.Qc.c = [0.3 0.2 0.1];
0024 
0025 sol = cmpi.solve_mip(t);
0026 assert(near(sol.x,t.Qc.c),'MIQP Qc obj val');
0027 
0028 t.Qc = [];
0029 t.Qd = [0 1 0;
0030         0 0 1;
0031         1 0 0];
0032 t.ctypes(1) = '=';
0033 sol = cmpi.solve_mip(t);
0034 assert(near(sol.x,[1/3 1/3 1/3]),'MIQP Qd obj val');
```

---

Generated on Thu 11-Aug-2011 15:06:22 by **m2html** © 2005
