# Supplementary material for: TIGER: Toolbox for integrating genome-scale metabolic models, expression data, and transcriptional regulatory networks
Source: BMC Syst Biol. 2011 Sep 23;5:147. doi: 10.1186/1752-0509-5-147 (PMC3224351; doi:10.1186/1752-0509-5-147)
Supplement: Additional file 2 — TIGER source code. Source code, documentation, and tutorials are also available online at http://bme.virginia.edu/csbl/downloads/ or http://csbl.bitbucket.org/tiger. [file 1752-0509-5-147-S2.GZ › tiger/doc/m2html/tiger/test/unit/tests/test__multilevel.html]

Description of test\_\_multilevel


Home > tiger > test > unit > tests > test\_\_multilevel.m

# test\_\_multilevel

## PURPOSE

## SYNOPSIS

**This is a script file.**

## DESCRIPTION

## CROSS-REFERENCE INFORMATION

This function calls:

- add\_rule Add rules to a TIGER model
- create\_empty\_tiger Create an empty TIGER model structure.
- fba Run Flux Balance Analysis on a TIGER model.
- set\_fieldval Set values in a TIGER structure field
- set\_var Set bounds on a variable
- init\_test
- near Test if two values are close to each other

This function is called by:


## SOURCE CODE

```
0001 
0002 init_test
0003 
0004 %%
0005 % IFF tests
0006 
0007 tiger = create_empty_tiger();
0008 
0009 rules = {'a & b <=> c';
0010          'c | d <=> f'};
0011      
0012 tiger = add_rule(tiger,rules,'default_ub',3);
0013 
0014 t = set_var(tiger,'b',3);
0015 t = set_fieldval(t,'obj','c',1);
0016 sol = fba(t);
0017 assert(near(sol.val,3),'multi iff and1');
0018 
0019 t = set_var(tiger,'b',2);
0020 t = set_fieldval(t,'obj','c',1);
0021 sol = fba(t);
0022 assert(near(sol.val,2),'multi iff and2');
0023 
0024 t = set_var(tiger,'c',1);
0025 t = set_fieldval(t,'obj','f',1);
0026 sol = fba(t);
0027 assert(near(sol.val,3),'multi iff or1');
0028 
0029 t = set_var(tiger,{'c','d'},1);
0030 t = set_fieldval(t,'obj','f',1);
0031 sol = fba(t);
0032 assert(near(sol.val,1),'multi iff or2');
0033 
0034 %%
0035 % IF tests
0036 
0037 tiger = create_empty_tiger();
0038 
0039 rules = {'a & b => c';
0040          'c | d => f'};
0041      
0042 tiger = add_rule(tiger,rules,'default_ub',3);
0043 
0044 t = set_var(tiger,'b',3);
0045 t = set_fieldval(t,'obj','c',1);
0046 sol = fba(t);
0047 assert(near(sol.val,3),'multi if and1');
0048 
0049 t = set_var(tiger,'b',2);
0050 t = set_fieldval(t,'obj','c',-1);
0051 sol = fba(t);
0052 assert(near(sol.val,0),'multi if and2');
0053 
0054 t = set_var(tiger,'c',1);
0055 t = set_fieldval(t,'obj','f',-1);
0056 sol = fba(t);
0057 assert(near(sol.val,-1),'multi if or1');
0058 
0059 t = set_var(tiger,{'c','d'},2);
0060 t = set_fieldval(t,'obj','f',-1);
0061 sol = fba(t);
0062 assert(near(sol.val,-2),'multi if or2');
0063
```

---

Generated on Thu 11-Aug-2011 15:06:22 by **m2html** © 2005
