# Supplementary material for: TIGER: Toolbox for integrating genome-scale metabolic models, expression data, and transcriptional regulatory networks
Source: BMC Syst Biol. 2011 Sep 23;5:147. doi: 10.1186/1752-0509-5-147 (PMC3224351; doi:10.1186/1752-0509-5-147)
Supplement: Additional file 2 — TIGER source code. Source code, documentation, and tutorials are also available online at http://bme.virginia.edu/csbl/downloads/ or http://csbl.bitbucket.org/tiger. [file 1752-0509-5-147-S2.GZ › tiger/doc/m2html/tiger/test/unit/tests/cobra_model.html]

Description of cobra\_model


Home > tiger > test > unit > tests > cobra\_model.m

# cobra\_model

## PURPOSE

**Test model in COBRA format**

## SYNOPSIS

**This is a script file.**

## DESCRIPTION

```
 COBRA_MODEL  Test model in COBRA format
```

## CROSS-REFERENCE INFORMATION

This function calls:

- convert\_grRules Parse grRules into rules for the COBRA toolbox
- make\_rxnGeneMat Build a rxnGeneMat for cobra models
- array2names Create a cell of names from an array of numbers

This function is called by:

- test\_\_fba
- test\_\_gimme
- test\_\_imat
- test\_\_indicators
- test\_\_made
- test\_\_solve\_multiple\_mips
- test\_\_tile\_mip

## SOURCE CODE

```
0001 % COBRA_MODEL  Test model in COBRA format
0002 
0003 %             1  2  3  4  5  6  7  8  9
0004 cobra.S =  [ -1  0  0  0 -1  0  0  0  0;   % A
0005               0 -1  0 -1  0  0  0  0  0;   % C
0006               0  0 -1  0  0 -1  0  0  0;   % F
0007               0  0  0  0  1 -1 -1  0  0;   % B
0008               0  0  0  1  0  0 -1 -1  0;   % D
0009               0  0  0  0  0  1  1  0 -1;   % E
0010               0  0  0  0  0  0  0  1  0 ]; % G
0011 
0012 cobra.lb = [ -1 -1 -1 -1 -1  0 -1  0  0 ]';
0013 cobra.ub = [  1  1  1  1  1  1  1  1  1 ]';
0014 
0015 cobra.c  = [  0  0  0  0  0  0  0  0  1 ]';
0016 
0017 [m,n] = size(cobra.S);
0018 
0019 cobra.b = zeros(m,1);
0020 
0021 cobra.rxns = array2names('r%i',1:n);
0022 cobra.mets = {'A','C','F','B','D','E','G'}';
0023 
0024 cobra.genes = {'g4','g5a','g5b','g5c','g5d', ...
0025                'g6','g7a','g7b','g8','i8'}';
0026 
0027 cobra.grRules = {'';
0028                  '';
0029                  '';
0030                  'g4';
0031                  'g5a and (g5b or (g5c and g5d))';
0032                  'g6';
0033                  'g7a and g7b';
0034                  'g8 or i8';
0035                  ''};
0036 
0037 cobra.rules = convert_grRules(cobra);
0038 cobra.rxnGeneMat = make_rxnGeneMat(cobra);
```

---

Generated on Thu 11-Aug-2011 15:06:22 by **m2html** © 2005
