# Supplementary material for: TIGER: Toolbox for integrating genome-scale metabolic models, expression data, and transcriptional regulatory networks
Source: BMC Syst Biol. 2011 Sep 23;5:147. doi: 10.1186/1752-0509-5-147 (PMC3224351; doi:10.1186/1752-0509-5-147)
Supplement: Additional file 2 — TIGER source code. Source code, documentation, and tutorials are also available online at http://bme.virginia.edu/csbl/downloads/ or http://csbl.bitbucket.org/tiger. [file 1752-0509-5-147-S2.GZ › tiger/doc/m2html/tiger/test/unit/tests/test__tile_milp.html]

Description of test\_\_tile\_milp


Home > tiger > test > unit > tests > test\_\_tile\_milp.m

# test\_\_tile\_milp

## PURPOSE

## SYNOPSIS

**This is a script file.**

## DESCRIPTION

## CROSS-REFERENCE INFORMATION

This function calls:

- cobra\_to\_tiger Convert a COBRA model to a TIGER model
- fba Run Flux Balance Analysis on a TIGER model.
- set\_var Set bounds on a variable
- cobra\_model Test model in COBRA format
- near Test if two values are close to each other

This function is called by:


## SOURCE CODE

```
0001 
0002 cobra_model;
0003 tiger = cobra_to_tiger(cobra);
0004 
0005 t1 = tiger;
0006 t1 = set_var(t1,'r1',-0.3);
0007 sol1 = fba(t1);
0008 
0009 t2 = tiger;
0010 t2 = set_var(t2,'r1',-0.2);
0011 sol2 = fba(t2);
0012 
0013 t3 = tiger;
0014 t3 = set_var(t3,'r1',-0.4);
0015 t3.obj = -1*t3.obj;
0016 t3.sense = -1;
0017 sol3 = cmpi.solve_mip(t3);
0018 
0019 n = size(tiger.A,2);
0020 
0021 milp = cmpi.tile_milp(t1,t2,t3);
0022 sol = cmpi.solve_mip(milp);
0023 val1 = t1.obj'*sol.x(1:n);
0024 val2 = t2.obj'*sol.x(n+1:2*n);
0025 val3 = t3.obj'*sol.x(2*n+1:3*n);
0026 
0027 assert(near(sol1.val,val1),'model 1');
0028 assert(near(sol2.val,val2),'model 2');
0029 assert(near(sol3.val,val3),'model 3');
0030
```

---

Generated on Thu 04-Aug-2011 09:58:54 by **m2html** © 2005
