# Supplementary material for: TIGER: Toolbox for integrating genome-scale metabolic models, expression data, and transcriptional regulatory networks
Source: BMC Syst Biol. 2011 Sep 23;5:147. doi: 10.1186/1752-0509-5-147 (PMC3224351; doi:10.1186/1752-0509-5-147)
Supplement: Additional file 2 — TIGER source code. Source code, documentation, and tutorials are also available online at http://bme.virginia.edu/csbl/downloads/ or http://csbl.bitbucket.org/tiger. [file 1752-0509-5-147-S2.GZ › tiger/doc/m2html/tiger/test/unit/tests/test__gimme.html]

Description of test\_\_gimme


Home > tiger > test > unit > tests > test\_\_gimme.m

# test\_\_gimme

## PURPOSE

## SYNOPSIS

**This is a script file.**

## DESCRIPTION

## CROSS-REFERENCE INFORMATION

This function calls:

- cobra\_to\_tiger Convert a COBRA model to a TIGER model
- fba Run Flux Balance Analysis on a TIGER model.
- cobra\_model Test model in COBRA format
- init\_test
- gimme Gene Inactivity Moderated by Metabolism and Expression
- near Test if two values are close to each other

This function is called by:


## SOURCE CODE

```
0001 
0002 init_test
0003 cobra_model
0004 
0005 express    = [   10   10     1     2    3];
0006 gene_names = {'g5a','g4','g5b','g5c','g6'};
0007 
0008 tiger = cobra_to_tiger(cobra);
0009 [states,genes,sol,t] = gimme(tiger,express,5,'gene_names',gene_names);
0010 
0011 assert(near(states,[1 1 0 1 0]),'states incorrect');
0012 assert(cmpi.is_acceptable_exit(fba(t)),'nonfunctional');
```

---

Generated on Thu 11-Aug-2011 15:06:22 by **m2html** © 2005
