# Supplementary material for: TIGER: Toolbox for integrating genome-scale metabolic models, expression data, and transcriptional regulatory networks
Source: BMC Syst Biol. 2011 Sep 23;5:147. doi: 10.1186/1752-0509-5-147 (PMC3224351; doi:10.1186/1752-0509-5-147)
Supplement: Additional file 2 — TIGER source code. Source code, documentation, and tutorials are also available online at http://bme.virginia.edu/csbl/downloads/ or http://csbl.bitbucket.org/tiger. [file 1752-0509-5-147-S2.GZ › tiger/doc/m2html/tiger/test/unit/tests/test__add_diff.html]

Description of test\_\_add\_diff


Home > tiger > test > unit > tests > test\_\_add\_diff.m

# test\_\_add\_diff

## PURPOSE

## SYNOPSIS

**This is a script file.**

## DESCRIPTION

## CROSS-REFERENCE INFORMATION

This function calls:

- add\_column Add a column to a TIGER model structure
- add\_diff Add difference variables toa TIGER model
- create\_empty\_tiger Create an empty TIGER model structure.
- fba Run Flux Balance Analysis on a TIGER model.
- set\_fieldval Set values in a TIGER structure field
- set\_var Set bounds on a variable
- init\_test
- near Test if two values are close to each other

This function is called by:


## SOURCE CODE

```
0001 
0002 init_test();
0003 
0004 tiger = create_empty_tiger();
0005 
0006 % TODO  find out why this is infeasible
0007 % tiger = add_column(tiger,{'x','y','z','w'},'cbib',[0,0,0,1],[6.3,1,3,1]);
0008 % tiger = add_diff(tiger,{'x','y','z','w'},{'y','x','x','y'});
0009 
0010 tiger = add_column(tiger,{'x','y','z','w'},'cbib',[0,0,0,0],[6.3,1,3,1]);
0011 tiger = add_diff(tiger,{'x','y','z','w'},{'y','x','x','y'});
0012 
0013 
0014 t = set_var(tiger,'x',2.67');
0015 t = set_var(t,'z',2);
0016 
0017 t1min = set_fieldval(t,'obj','diff__x_y',-1);
0018 t1max = set_fieldval(t,'obj','diff__x_y', 1);
0019 t2min = set_fieldval(t,'obj','diff__y_x',-1);
0020 t2max = set_fieldval(t,'obj','diff__y_x', 1);
0021 t3min = set_fieldval(t,'obj','diff__z_x',-1);
0022 t3max = set_fieldval(t,'obj','diff__z_x', 1);
0023 t4min = set_fieldval(t,'obj','diff__w_y',-1);
0024 t4max = set_fieldval(t,'obj','diff__w_y', 1);
0025 
0026 sol1min = fba(t1min);
0027 sol1max = fba(t1max);
0028 %show_sol(t1,sol1)
0029 assert(near(-sol1min.val,1.67),'sol1 min');
0030 assert(near( sol1max.val,2.67),'sol1 max');
0031 
0032 sol2min = fba(t2min);
0033 sol2max = fba(t2max);
0034 %show_sol(t2,sol2)
0035 assert(near(-sol2min.val,1.67),'sol2 min');
0036 assert(near( sol2max.val,2.67),'sol2 max');
0037 
0038 sol3min = fba(t3min);
0039 sol3max = fba(t3max);
0040 %show_sol(t3,sol3)
0041 assert(near(-sol3min.val,0.67),'sol3 min');
0042 assert(near( sol3max.val,0.67),'sol3 max');
0043 
0044 sol4min = fba(t4min);
0045 sol4max = fba(t4max);
0046 %show_sol(t4,sol4)
0047 assert(near(-sol4min.val,0),'sol4 min');
0048 assert(near( sol4max.val,1),'sol4 max');
```

---

Generated on Thu 11-Aug-2011 15:06:22 by **m2html** © 2005
