# Supplementary material for: TIGER: Toolbox for integrating genome-scale metabolic models, expression data, and transcriptional regulatory networks
Source: BMC Syst Biol. 2011 Sep 23;5:147. doi: 10.1186/1752-0509-5-147 (PMC3224351; doi:10.1186/1752-0509-5-147)
Supplement: Additional file 2 — TIGER source code. Source code, documentation, and tutorials are also available online at http://bme.virginia.edu/csbl/downloads/ or http://csbl.bitbucket.org/tiger. [file 1752-0509-5-147-S2.GZ › tiger/doc/m2html/tiger/test/unit/tests/test__find_optimal_states.html]

Description of test\_\_find\_optimal\_states


Home > tiger > test > unit > tests > test\_\_find\_optimal\_states.m

# test\_\_find\_optimal\_states

## PURPOSE

## SYNOPSIS

**This is a script file.**

## DESCRIPTION

## CROSS-REFERENCE INFORMATION

This function calls:

- find\_optimal\_states Find optimal binary states

This function is called by:


## SOURCE CODE

```
0001 
0002 T = [ 0 1 0 0;
0003       0 0 2 0;
0004       0 0 0 3 ];
0005 
0006 d = [ 1 -1 -1 ];
0007 w = [ 1  3 10 ];
0008   
0009 [states,score,matched,total] = find_optimal_states(d,T,w);
```

---

Generated on Thu 11-Aug-2011 15:06:22 by **m2html** © 2005
