# Supplementary material for: TIGER: Toolbox for integrating genome-scale metabolic models, expression data, and transcriptional regulatory networks
Source: BMC Syst Biol. 2011 Sep 23;5:147. doi: 10.1186/1752-0509-5-147 (PMC3224351; doi:10.1186/1752-0509-5-147)
Supplement: Additional file 2 — TIGER source code. Source code, documentation, and tutorials are also available online at http://bme.virginia.edu/csbl/downloads/ or http://csbl.bitbucket.org/tiger. [file 1752-0509-5-147-S2.GZ › tiger/doc/m2html/tiger/test/unit/tests/test__create_table.html]

Description of test\_\_create\_table


Home > tiger > test > unit > tests > test\_\_create\_table.m

# test\_\_create\_table

## PURPOSE

## SYNOPSIS

**This is a script file.**

## DESCRIPTION

## CROSS-REFERENCE INFORMATION

This function calls:

- create\_table Format and display tabular data

This function is called by:


## SOURCE CODE

```
0001 
0002 cols = {'first', 'second', 'third'};
0003 rows = {'A', 'B', 'Ces', 'D'};
0004 
0005 data = rand(4,3);
0006 
0007 create_table(data,'columnlabels',cols,'rowlabels',rows);
```

---

Generated on Thu 11-Aug-2011 15:06:22 by **m2html** © 2005
