# Supplementary material for: TIGER: Toolbox for integrating genome-scale metabolic models, expression data, and transcriptional regulatory networks
Source: BMC Syst Biol. 2011 Sep 23;5:147. doi: 10.1186/1752-0509-5-147 (PMC3224351; doi:10.1186/1752-0509-5-147)
Supplement: Additional file 2 — TIGER source code. Source code, documentation, and tutorials are also available online at http://bme.virginia.edu/csbl/downloads/ or http://csbl.bitbucket.org/tiger. [file 1752-0509-5-147-S2.GZ › tiger/doc/m2html/tiger/test/unit/tests/test__fba.html]

Description of test\_\_fba


Home > tiger > test > unit > tests > test\_\_fba.m

# test\_\_fba

## PURPOSE

## SYNOPSIS

**This is a script file.**

## DESCRIPTION

## CROSS-REFERENCE INFORMATION

This function calls:

- cobra\_to\_tiger Convert a COBRA model to a TIGER model
- fba Run Flux Balance Analysis on a TIGER model.
- set\_var Set bounds on a variable
- cobra\_model Test model in COBRA format
- init\_test
- near Test if two values are close to each other

This function is called by:


## SOURCE CODE

```
0001 
0002 cobra_model
0003 init_test
0004 
0005 tiger = cobra_to_tiger(cobra);
0006 
0007 wtsol = fba(tiger);
0008 assert(near(wtsol.val,1),'original fba');
0009 
0010 sol = fba(set_var(tiger,'g5a',0));
0011 assert(near(sol.val),'g5a ko');
0012 
0013 sol = fba(set_var(tiger,'g7a',0));
0014 assert(near(sol.val,wtsol.val),'g7a ko');
0015 
0016 sol = fba(set_var(tiger,{'g7a','g6'},0));
0017 assert(near(sol.val),'g7a + g6 ko');
```

---

Generated on Thu 11-Aug-2011 15:06:22 by **m2html** © 2005
