# Supplementary material for: TIGER: Toolbox for integrating genome-scale metabolic models, expression data, and transcriptional regulatory networks
Source: BMC Syst Biol. 2011 Sep 23;5:147. doi: 10.1186/1752-0509-5-147 (PMC3224351; doi:10.1186/1752-0509-5-147)
Supplement: Additional file 2 — TIGER source code. Source code, documentation, and tutorials are also available online at http://bme.virginia.edu/csbl/downloads/ or http://csbl.bitbucket.org/tiger. [file 1752-0509-5-147-S2.GZ › tiger/doc/m2html/tiger/test/unit/tests/test__solve_multiple_mips.html]

Description of test\_\_solve\_multiple\_mips


Home > tiger > test > unit > tests > test\_\_solve\_multiple\_mips.m

# test\_\_solve\_multiple\_mips

## PURPOSE

## SYNOPSIS

**This is a script file.**

## DESCRIPTION

## CROSS-REFERENCE INFORMATION

This function calls:

- cobra\_to\_tiger Convert a COBRA model to a TIGER model
- cobra\_model Test model in COBRA format
- init\_test
- near Test if two values are close to each other

This function is called by:


## SOURCE CODE

```
0001 
0002 
0003 init_test
0004 
0005 cobra_model
0006 
0007 tiger = cobra_to_tiger(cobra);
0008 
0009 nrxns = size(tiger.S,2);
0010 tiger.obj(:) = 0;
0011 alts.obj = repmat(tiger.obj,1,2*nrxns);
0012 
0013 for i = 1 : nrxns
0014     alts.obj(i,i) = 1;
0015     alts.obj(i,i+nrxns) = -1;
0016 end
0017 
0018 sols1 = cmpi.solve_multiple_mips(tiger,alts,'restart',false);
0019 
0020 sols2 = cmpi.solve_multiple_mips(tiger,alts,'restart',true);
0021 
0022 
0023 assert(near(sols1.flag,sols2.flag),'flag off');
0024 assert(near(sols1.val,sols2.val),'vals off');
```

---

Generated on Thu 11-Aug-2011 15:06:22 by **m2html** © 2005
