# Supplementary material for: TIGER: Toolbox for integrating genome-scale metabolic models, expression data, and transcriptional regulatory networks
Source: BMC Syst Biol. 2011 Sep 23;5:147. doi: 10.1186/1752-0509-5-147 (PMC3224351; doi:10.1186/1752-0509-5-147)
Supplement: Additional file 2 — TIGER source code. Source code, documentation, and tutorials are also available online at http://bme.virginia.edu/csbl/downloads/ or http://csbl.bitbucket.org/tiger. [file 1752-0509-5-147-S2.GZ › tiger/doc/m2html/tiger/test/unit/tests/test__made.html]

Description of test\_\_made


Home > tiger > test > unit > tests > test\_\_made.m

# test\_\_made

## PURPOSE

## SYNOPSIS

**This is a script file.**

## DESCRIPTION

## CROSS-REFERENCE INFORMATION

This function calls:

- cobra\_to\_tiger Convert a COBRA model to a TIGER model
- cobra\_model Test model in COBRA format
- made Metabolic Adjustment by Differential Expression

This function is called by:


## SOURCE CODE

```
0001 
0002 cobra_model
0003 
0004 gene_names = {'g7a','g7b','g4','g8c'};
0005 
0006 fc =    [   2   2;  % g7a
0007             1 0.9;  % g7b
0008             2   2;  % g4
0009             2   2]; % g8
0010             
0011 pvals = [ 0.1 0.1;  % g7a
0012           0.1 0.1;  % g7b
0013           0.1 0.1;  % g4
0014           0.1 0.1]; % g8
0015 
0016 tiger = cobra_to_tiger(cobra);
0017 [sol] = made(tiger,fc,pvals, ...
0018              'weighting','linear', ...
0019              'gene_names',gene_names, ...
0020              'verbose',false);
```

---

Generated on Thu 11-Aug-2011 15:06:22 by **m2html** © 2005
