# Supplementary material for: TIGER: Toolbox for integrating genome-scale metabolic models, expression data, and transcriptional regulatory networks
Source: BMC Syst Biol. 2011 Sep 23;5:147. doi: 10.1186/1752-0509-5-147 (PMC3224351; doi:10.1186/1752-0509-5-147)
Supplement: Additional file 2 — TIGER source code. Source code, documentation, and tutorials are also available online at http://bme.virginia.edu/csbl/downloads/ or http://csbl.bitbucket.org/tiger. [file 1752-0509-5-147-S2.GZ › tiger/doc/m2html/tiger/test/unit/tests/test__diffadj.html]

Description of test\_\_diffadj


Home > tiger > test > unit > tests > test\_\_diffadj.m

# test\_\_diffadj

## PURPOSE

## SYNOPSIS

**This is a script file.**

## DESCRIPTION

## CROSS-REFERENCE INFORMATION

This function calls:

- add\_column Add a column to a TIGER model structure
- add\_row Add a row to a TIGER model structure
- create\_empty\_tiger Create an empty TIGER model structure.
- init\_test
- diffadj Formulate and solve the differential adjustment problem
- near Test if two values are close to each other

This function is called by:


## SOURCE CODE

```
0001 
0002 init_test
0003 
0004 tiger = create_empty_tiger();
0005 tiger = add_column(tiger,{'a','b','c','x','y','z'},'bbbiii', ...
0006                          [0;0;0;0;0;0],[1;1;1;2;2;3],[],[]);
0007 tiger = add_row(tiger,3);
0008 
0009 tiger.genes = tiger.varnames;
0010 tiger.S = tiger.A;
0011 
0012 vars = 1:6;
0013 
0014 %     1   2   3
0015 d = [   1   1;  % a
0016        -1   1;  % b
0017        -1  -1;  % c
0018         1   0;  % x
0019        -1  -1;  % y
0020         1  -1]; % z
0021     
0022 w = [   2   4;  % a
0023         3   1;  % b
0024         2   1;  % c
0025         1   1;  % x
0026         2   2;  % y
0027         1   1]; % z
0028     
0029 [states,sol,mip_error,models] = diffadj(tiger,vars,d,w);
0030 
0031 assert(mip_error == 0,'mip error');
0032 
0033 optimal = [ 0     0     1
0034             1     0     1
0035             1     0     0
0036             0     2     2
0037             2     0     0
0038             0     3     0 ];
0039 
0040 assert(near(states,optimal),'answer error');
```

---

Generated on Thu 11-Aug-2011 15:06:22 by **m2html** © 2005
