# Supplementary material for: TIGER: Toolbox for integrating genome-scale metabolic models, expression data, and transcriptional regulatory networks
Source: BMC Syst Biol. 2011 Sep 23;5:147. doi: 10.1186/1752-0509-5-147 (PMC3224351; doi:10.1186/1752-0509-5-147)
Supplement: Additional file 2 — TIGER source code. Source code, documentation, and tutorials are also available online at http://bme.virginia.edu/csbl/downloads/ or http://csbl.bitbucket.org/tiger. [file 1752-0509-5-147-S2.GZ › tiger/doc/m2html/tiger/test/unit/tests/test__remove_rule.html]

Description of test\_\_remove\_rule


Home > tiger > test > unit > tests > test\_\_remove\_rule.m

# test\_\_remove\_rule

## PURPOSE

## SYNOPSIS

**This is a script file.**

## DESCRIPTION

## CROSS-REFERENCE INFORMATION

This function calls:

- add\_column Add a column to a TIGER model structure
- add\_rule Add rules to a TIGER model
- create\_empty\_tiger Create an empty TIGER model structure.
- remove\_rule Remove rule(s) previously added to a TIGER model
- set\_fieldval Set values in a TIGER structure field
- solve\_tiger Solve a TIGER model.
- init\_test
- near Test if two values are close to each other

This function is called by:


## SOURCE CODE

```
0001 
0002 init_test();
0003 
0004 t = create_empty_tiger();
0005 
0006 t = add_column(t,'v','c');
0007 
0008 r1 = 'a | b => c';
0009 r2 = 'v < 0.33 => ~b';
0010 r3 = 'c & b <=> f';
0011 
0012 t = add_rule(t,{r1,r2,r3});
0013 t = set_fieldval(t,'obj',{'b','v'},[-1 1]);
0014 
0015 s = solve_tiger(t);
0016 %show_sol(t,solve_tiger(t));
0017 
0018 t2 = remove_rule(t,r2);
0019 s2 = solve_tiger(t2);
0020 %show_sol(t2,solve_tiger(t2));
0021 
0022 assert(near(s.val,-0.67),'original obj val');
0023 assert(near(s2.val,-1),'removed obj val');
```

---

Generated on Thu 11-Aug-2011 15:06:22 by **m2html** © 2005
