# Supplementary material for: TIGER: Toolbox for integrating genome-scale metabolic models, expression data, and transcriptional regulatory networks
Source: BMC Syst Biol. 2011 Sep 23;5:147. doi: 10.1186/1752-0509-5-147 (PMC3224351; doi:10.1186/1752-0509-5-147)
Supplement: Additional file 2 — TIGER source code. Source code, documentation, and tutorials are also available online at http://bme.virginia.edu/csbl/downloads/ or http://csbl.bitbucket.org/tiger. [file 1752-0509-5-147-S2.GZ › tiger/doc/m2html/tiger/test/yeast/index.html]

Index for Directory tiger/test/yeast


|  |  |
| --- | --- |
| Master index | Index for tiger/test/yeast |

# Index for tiger/test/yeast

## Matlab files in this directory:

|  |  |
| --- | --- |
| apply\_aliases | Change atom aliases to the same name |
| convert\_rules |  |
| create\_yeast\_trn\_model |  |
| infeas\_study |  |
| load\_rules |  |
| minimal\_genome\_study |  |
| run\_new\_rules |  |
| run\_trn\_comparison |  |

## Other Matlab-specific files in this directory:

- ind750.mat

## Subsequent directories:

- old

---

Generated on Thu 11-Aug-2011 15:06:20 by **m2html** © 2005
