# Supplementary material for: TIGER: Toolbox for integrating genome-scale metabolic models, expression data, and transcriptional regulatory networks
Source: BMC Syst Biol. 2011 Sep 23;5:147. doi: 10.1186/1752-0509-5-147 (PMC3224351; doi:10.1186/1752-0509-5-147)
Supplement: Additional file 2 — TIGER source code. Source code, documentation, and tutorials are also available online at http://bme.virginia.edu/csbl/downloads/ or http://csbl.bitbucket.org/tiger. [file 1752-0509-5-147-S2.GZ › tiger/doc/m2html/tiger/test/yeast/load_rules.html]

Description of load\_rules


Home > tiger > test > yeast > load\_rules.m

# load\_rules

## PURPOSE

## SYNOPSIS

**This is a script file.**

## DESCRIPTION

## CROSS-REFERENCE INFORMATION

This function calls:

- add\_column Add a column to a TIGER model structure
- add\_growth\_constraint Add minimum growth constraint to a model.
- add\_rule Add rules to a TIGER model
- cobra\_to\_tiger Convert a COBRA model to a TIGER model
- find\_infeasible\_rules Determine which rules make a model infeasible.
- parse\_string Parse a rule string into an EXPR object
- apply\_aliases Change atom aliases to the same name
- celliter Iterate over elements in a cell
- find\_like Find matches in a cell of strings
- map Generate a new list by applying a function

This function is called by:


## SOURCE CODE

```
0001 
0002 load ind750
0003 
0004 fid = fopen('aliases.csv');
0005 aliases = textscan(fid,'%q%q','Delimiter',',','CollectOutput',true);
0006 aliases = aliases{1};
0007 fclose(fid);
0008 
0009 fid = fopen('rules.csv');
0010 raw = textscan(fid,'%q%q','Delimiter',',','CollectOutput',true);
0011 raw = raw{1};
0012 fclose(fid);
0013 
0014 Nrules = size(raw,1);
0015 rules = cell(Nrules,1);
0016 for i = 1 : Nrules
0017     rules{i} = [raw{i,2}(4:end) ' <=> ' raw{i,1}];
0018 end
0019 
0020 % remove infeasible
0021 infeas = [79 104 130 300 428];
0022 for i = 1 : length(infeas)
0023     rules{infeas(i)} = 'a => b';
0024 end
0025     
0026 exprs = parse_string(rules);
0027 celliter(@(x) apply_aliases(x,aliases),exprs);
0028 
0029 all_atoms = map(@(x) x.atoms,exprs);
0030 all_atoms = unique([all_atoms{:}])';
0031 
0032 unbound = setdiff(find_like('^[^Y]',all_atoms), ...
0033                   find_like('\[e\]$',cobra.mets));
0034 
0035 tic; tiger_base = cobra_to_tiger(cobra); toc
0036 mets = find_like('\[e\]$',all_atoms);              
0037 tiger_base = add_column(tiger_base,mets);
0038 %tiger_base = bind_mets(tiger_base);
0039 %tiger_base = bind_var(tiger_base,{'EX_glc(e)'},{'glc[e]'},'iff',true);
0040 
0041 tinf = add_growth_constraint(tiger_base,0.01,'valtype','abs');
0042 [infeas,side] = find_infeasible_rules(tinf,exprs);
0043 
0044 
0045 tic; trn  = add_rule(tiger_base,exprs);  toc
0046 
0047 %trn = bind_mets(trn);
0048 %trn = bind_var(trn,{'EX_glc(e)'},{'glc[e]'},'iff',true);
0049 
0050 save yeast_trn_iff.mat trn
```

---

Generated on Thu 11-Aug-2011 15:06:22 by **m2html** © 2005
