# Supplementary material for: TIGER: Toolbox for integrating genome-scale metabolic models, expression data, and transcriptional regulatory networks
Source: BMC Syst Biol. 2011 Sep 23;5:147. doi: 10.1186/1752-0509-5-147 (PMC3224351; doi:10.1186/1752-0509-5-147)
Supplement: Additional file 2 — TIGER source code. Source code, documentation, and tutorials are also available online at http://bme.virginia.edu/csbl/downloads/ or http://csbl.bitbucket.org/tiger. [file 1752-0509-5-147-S2.GZ › tiger/doc/m2html/tiger/test/yeast/convert_rules.html]

Description of convert\_rules


Home > tiger > test > yeast > convert\_rules.m

# convert\_rules

## PURPOSE

## SYNOPSIS

**This is a script file.**

## DESCRIPTION

## CROSS-REFERENCE INFORMATION

This function calls:

- add\_rule Add rules to a TIGER model
- bind\_mets Bind metabolites to exchange reactions.
- bind\_var Bind variables to a indicator variable
- cobra\_to\_tiger Convert a COBRA model to a TIGER model
- parse\_string Parse a rule string into an EXPR object
- apply\_aliases Change atom aliases to the same name

This function is called by:


## SOURCE CODE

```
0001 
0002 load yeast_raw_rules.mat
0003 load yeast_aliases.mat
0004 
0005 N = length(yeast_raw_rules);
0006 if_rules  = cell(N,1);
0007 iff_rules = cell(N,1);
0008 for i = 1 : N
0009     pred = yeast_raw_rules{i,2};
0010     if_rules{i}  = [pred(3:end)  ' => ' yeast_raw_rules{i,1}];
0011     iff_rules{i} = [pred(3:end) ' <=> ' yeast_raw_rules{i,1}];
0012 end
0013 
0014 if_exprs  = cellfun(@parse_string, if_rules,'Uniform',false);
0015 iff_exprs = cellfun(@parse_string,iff_rules,'Uniform',false);
0016 
0017 cellfun(@(x) apply_aliases(x,yeast_aliases), if_exprs);
0018 cellfun(@(x) apply_aliases(x,yeast_aliases),iff_exprs);
0019 
0020 tic; tiger_base = cobra_to_tiger(cobra); toc
0021 
0022 tic; if_trn  = add_rule(tiger_base,if_exprs);  toc
0023 tic; iff_trn = add_rule(tiger_base,iff_exprs); toc
0024 
0025 yeast_if = bind_mets(if_trn);
0026 yeast_if = bind_var(yeast_if,{'EX_glc(e)'},{'glc[e]'});
0027 yeast_iff = bind_mets(iff_trn);
0028 yeast_iff = bind_var(yeast_iff,{'EX_glc(e)'},{'glc[e]'});
```

---

Generated on Thu 11-Aug-2011 15:06:22 by **m2html** © 2005
