# Supplementary material for: TIGER: Toolbox for integrating genome-scale metabolic models, expression data, and transcriptional regulatory networks
Source: BMC Syst Biol. 2011 Sep 23;5:147. doi: 10.1186/1752-0509-5-147 (PMC3224351; doi:10.1186/1752-0509-5-147)
Supplement: Additional file 2 — TIGER source code. Source code, documentation, and tutorials are also available online at http://bme.virginia.edu/csbl/downloads/ or http://csbl.bitbucket.org/tiger. [file 1752-0509-5-147-S2.GZ › tiger/doc/m2html/tiger/test/yeast/infeas_study.html]

Description of infeas\_study


Home > tiger > test > yeast > infeas\_study.m

# infeas\_study

## PURPOSE

## SYNOPSIS

**This is a script file.**

## DESCRIPTION

## CROSS-REFERENCE INFORMATION

This function calls:

- add\_growth\_constraint Add minimum growth constraint to a model.
- add\_rule Add rules to a TIGER model
- find\_infeasible\_rules Determine which rules make a model infeasible.

This function is called by:


## SOURCE CODE

```
0001 
0002 base_mets = add_rule(base,met_rules);
0003 %base_mets = base;
0004 
0005 base_mets.lb(1:1266) = cobra.lb;
0006 base_mets.ub(1:1266) = cobra.ub;
0007 
0008 wg = add_growth_constraint(base_mets,0.3);
0009 %wg = set_var(wg,'glc[e]',1);
0010 
0011 
0012 
0013 [infeas,side] = find_infeasible_rules(wg,exprs)
0014
```

---

Generated on Thu 11-Aug-2011 15:06:22 by **m2html** © 2005
