# Supplementary material for: TIGER: Toolbox for integrating genome-scale metabolic models, expression data, and transcriptional regulatory networks
Source: BMC Syst Biol. 2011 Sep 23;5:147. doi: 10.1186/1752-0509-5-147 (PMC3224351; doi:10.1186/1752-0509-5-147)
Supplement: Additional file 2 — TIGER source code. Source code, documentation, and tutorials are also available online at http://bme.virginia.edu/csbl/downloads/ or http://csbl.bitbucket.org/tiger. [file 1752-0509-5-147-S2.GZ › tiger/doc/m2html/tiger/test/yeast/create_yeast_trn_model.html]

Description of create\_yeast\_trn\_model


Home > tiger > test > yeast > create\_yeast\_trn\_model.m

# create\_yeast\_trn\_model

## PURPOSE

## SYNOPSIS

**function create\_yeast\_trn\_model()**

## DESCRIPTION

## CROSS-REFERENCE INFORMATION

This function calls:

- add\_rule Add rules to a TIGER model
- close\_bounds Return bounds that have been opened by OPEN\_BOUNDS
- cobra\_to\_tiger Convert a COBRA model to a TIGER model
- open\_bounds Open all bounds to a max value
- parse\_string Parse a rule string into an EXPR object
- apply\_aliases Change atom aliases to the same name
- cellfilter Return a subset of a cell array
- celliter Iterate over elements in a cell
- find\_like Find matches in a cell of strings

This function is called by:


## SUBFUNCTIONS

- function [str] = combine\_rules(rules)

## SOURCE CODE

```
0001 function create_yeast_trn_model()
0002 
0003 load ind750
0004 
0005 [cobra,bounds] = open_bounds(cobra);
0006 
0007 fid = fopen('aliases.csv');
0008 aliases = textscan(fid,'%q%q','Delimiter',',','CollectOutput',true);
0009 aliases = aliases{1};
0010 fclose(fid);
0011 
0012 fid = fopen('interactions.csv');
0013 raw = textscan(fid,'%q%q%q%q','Delimiter',',','CollectOutput',true);
0014 raw = raw{1};
0015 fclose(fid);
0016 
0017 fid = fopen('met_rules.csv');
0018 met_rules = textscan(fid,'%s','Delimiter','$','CollectOutput',true);
0019 met_rules = met_rules{1};
0020 met_rules = met_rules(1:66);
0021 fclose(fid);
0022 
0023 orfs = unique(raw(:,1));
0024 Norfs = length(orfs);
0025 rules = cell(Norfs,1);
0026 for i = 1 : Norfs
0027     [~,~,tf] = find_like(['^' orfs{i} '$'],raw(:,1));
0028     orf_rules = raw(tf,:);
0029     [~,~,tf] = cellfilter(@(x) x == 'A',orf_rules(:,4));
0030     actstr = combine_rules(orf_rules( tf,:));
0031     repstr = combine_rules(orf_rules(~tf,:));
0032     
0033     if ~isempty(actstr) && ~isempty(repstr)
0034         rules{i} = sprintf('(%s) or not(%s) <=> %s', ...
0035                            actstr,repstr,orfs{i});
0036     elseif ~isempty(actstr)
0037         rules{i} = sprintf('%s => %s',actstr,orfs{i});
0038     elseif ~isempty(repstr)
0039         rules{i} = sprintf('%s => not %s',repstr,orfs{i});
0040     else
0041         rules{i} = '';
0042     end
0043 end
0044     
0045 rules = cellfilter(@(x) ~isempty(x),rules);
0046 rules{end+1} = 'HAP1 and ("EX_o2(e)" > -0.24338) => YPR065W';
0047 rules{end+1} = 'etoh[e] <=> not o2[e]';
0048 
0049 exprs = parse_string(rules);
0050 celliter(@(x) apply_aliases(x,aliases),exprs);
0051 
0052 tic
0053 base = cobra_to_tiger(cobra);
0054 toc
0055 tic
0056 base_rules = add_rule(base,exprs);
0057 yeast_trn = add_rule(base_rules,met_rules);
0058 toc
0059 
0060 yeast_trn = close_bounds(yeast_trn,bounds);
0061 
0062 save yeast_trn_model.mat base yeast_trn exprs met_rules rules bounds
0063 
0064 
0065 function [str] = combine_rules(rules)
0066     is_na = @(x) strcmp(x,'NA');
0067     
0068     str = '';
0069     N = size(rules,1);
0070     for i = 1 : N
0071         tf  = rules{i,2};
0072         met = rules{i,3};
0073         if ~is_na(tf) && ~is_na(met)
0074             to_add = sprintf('("%s" & "%s")',tf,met);
0075         elseif ~is_na(tf)
0076             to_add = ['"' tf '"'];
0077         elseif ~is_na(met)
0078             to_add = ['"' met '"'];
0079         else
0080             to_add = '';
0081         end
0082         
0083         if ~isempty(to_add)
0084             if i > 1
0085                 str = [str ' or ' to_add];
0086             else
0087                 str = to_add;
0088             end
0089         end
0090     end
0091             
0092
```

---

Generated on Thu 11-Aug-2011 15:06:22 by **m2html** © 2005
