# Supplementary material for: TIGER: Toolbox for integrating genome-scale metabolic models, expression data, and transcriptional regulatory networks
Source: BMC Syst Biol. 2011 Sep 23;5:147. doi: 10.1186/1752-0509-5-147 (PMC3224351; doi:10.1186/1752-0509-5-147)
Supplement: Additional file 2 — TIGER source code. Source code, documentation, and tutorials are also available online at http://bme.virginia.edu/csbl/downloads/ or http://csbl.bitbucket.org/tiger. [file 1752-0509-5-147-S2.GZ › tiger/doc/m2html/tiger/test/yeast/run_new_rules.html]

Description of run\_new\_rules


Home > tiger > test > yeast > run\_new\_rules.m

# run\_new\_rules

## PURPOSE

## SYNOPSIS

**This is a script file.**

## DESCRIPTION

## CROSS-REFERENCE INFORMATION

This function calls:

- add\_rule Add rules to a TIGER model
- close\_bounds Return bounds that have been opened by OPEN\_BOUNDS
- fba Run Flux Balance Analysis on a TIGER model.

This function is called by:


## SOURCE CODE

```
0001 load yeast_trn_model
0002 
0003 base_closed = close_bounds(base,bounds);
0004 base_mets = add_rule(base_closed,met_rules);
0005 
0006 y = add_rule(base_mets,{'o2[e] or not ROX1 <=> HAP1', ...
0007                         'o2[e] and HAP1 <=> ROX1', ...
0008                         '"EX_o2(e)" < -0.24338 <=> high_o2', ...
0009                         'glc[e] and HAP1 and (not ROX1 or high_o2)) <=> YHR007C'});
0010                     
0011 fba(y)
```

---

Generated on Thu 11-Aug-2011 15:06:22 by **m2html** © 2005
