# Supplementary material for: TIGER: Toolbox for integrating genome-scale metabolic models, expression data, and transcriptional regulatory networks
Source: BMC Syst Biol. 2011 Sep 23;5:147. doi: 10.1186/1752-0509-5-147 (PMC3224351; doi:10.1186/1752-0509-5-147)
Supplement: Additional file 2 — TIGER source code. Source code, documentation, and tutorials are also available online at http://bme.virginia.edu/csbl/downloads/ or http://csbl.bitbucket.org/tiger. [file 1752-0509-5-147-S2.GZ › tiger/doc/m2html/tiger/test/yeast/apply_aliases.html]

Description of apply\_aliases


Home > tiger > test > yeast > apply\_aliases.m

# apply\_aliases

## PURPOSE

**Change atom aliases to the same name**

## SYNOPSIS

**function apply\_aliases(ex,aliases)**

## DESCRIPTION

```
 APPLY_ALIASES  Change atom aliases to the same name

   [EX] = APPLY_ALIASES(EX,ALIASES)

   ALIASES is a N x 2 cell of aliases, where the first column is the
   alias, and the second column is the name that should be substituted
   for the alias.
```

## CROSS-REFERENCE INFORMATION

This function calls:


This function is called by:

- convert\_rules
- create\_yeast\_trn\_model
- load\_rules

## SUBFUNCTIONS

- function swapif(e,name,alias)

## SOURCE CODE

```
0001 function apply_aliases(ex,aliases)
0002 % APPLY_ALIASES  Change atom aliases to the same name
0003 %
0004 %   [EX] = APPLY_ALIASES(EX,ALIASES)
0005 %
0006 %   ALIASES is a N x 2 cell of aliases, where the first column is the
0007 %   alias, and the second column is the name that should be substituted
0008 %   for the alias.
0009 
0010 tf = ismember(aliases(:,1),ex.atoms);
0011 aliases = aliases(tf,:);
0012 
0013 atom_test = @(e) e.is_atom;
0014 for i = 1 : size(aliases,1)
0015     ex.iterif(atom_test,@(x) swapif(x,aliases{i,1},aliases{i,2}));
0016 end
0017     
0018 function swapif(e,name,alias)
0019     if strcmp(e.id,name)
0020         e.id = alias;
0021     end
0022
```

---

Generated on Thu 11-Aug-2011 15:06:22 by **m2html** © 2005
