# Supplementary material for: TIGER: Toolbox for integrating genome-scale metabolic models, expression data, and transcriptional regulatory networks
Source: BMC Syst Biol. 2011 Sep 23;5:147. doi: 10.1186/1752-0509-5-147 (PMC3224351; doi:10.1186/1752-0509-5-147)
Supplement: Additional file 2 — TIGER source code. Source code, documentation, and tutorials are also available online at http://bme.virginia.edu/csbl/downloads/ or http://csbl.bitbucket.org/tiger. [file 1752-0509-5-147-S2.GZ › tiger/doc/m2html/tiger/test/yeast/old/gal_test.html]

Description of gal\_test


Home > tiger > test > yeast > old > gal\_test.m

# gal\_test

## PURPOSE

## SYNOPSIS

**This is a script file.**

## DESCRIPTION

## CROSS-REFERENCE INFORMATION

This function calls:

- add\_growth\_constraint Add minimum growth constraint to a model.
- fba Run Flux Balance Analysis on a TIGER model.

This function is called by:


## SOURCE CODE

```
0001 
0002 gal = add_growth_constraint(trn,0.13,'ctype','=','valtype','abs');
0003 gal.lb(carbon_idxs) = 0;
0004 gal.lb(425) = -100;
0005 
0006 gal.obj(425) = 1;
0007 
0008 sol = fba(gal)
0009 
0010 %cmpi.show_mip(make_milp(gal))
```

---

Generated on Thu 11-Aug-2011 15:06:22 by **m2html** © 2005
