# Supplementary material for: TIGER: Toolbox for integrating genome-scale metabolic models, expression data, and transcriptional regulatory networks
Source: BMC Syst Biol. 2011 Sep 23;5:147. doi: 10.1186/1752-0509-5-147 (PMC3224351; doi:10.1186/1752-0509-5-147)
Supplement: Additional file 2 — TIGER source code. Source code, documentation, and tutorials are also available online at http://bme.virginia.edu/csbl/downloads/ or http://csbl.bitbucket.org/tiger. [file 1752-0509-5-147-S2.GZ › tiger/doc/m2html/tiger/test/yeast/minimal_genome_study.html]

Description of minimal\_genome\_study


Home > tiger > test > yeast > minimal\_genome\_study.m

# minimal\_genome\_study

## PURPOSE

## SYNOPSIS

**This is a script file.**

## DESCRIPTION

## CROSS-REFERENCE INFORMATION

This function calls:

- convert\_ids Create name, indices, and logical indices from an array

This function is called by:


## SOURCE CODE

```
0001 
0002 cmpi.init();
0003 load ind750
0004 load yeast_tiger
0005 
0006 is_ex_f = @(x) length(x) >= 3 && strcmp(x(1:3),'EX_');
0007 ex_idxs = find(cellfun(is_ex_f,cobra.rxns));
0008 
0009 minimal_mets = {'h2o(e)','nh4(e)','o2(e)','pi(e)','so4(e)'};
0010 minimal_idxs = [    436,     456,     458,   466,     476 ];
0011 
0012 carbon_sources = {'glc','gal','fruc','man','sucr','etoh','glyc','pyr','rib','succ'};
0013 carbon_idxs    = [  428   425    423   451    481    420    432   470   471    480];
0014 
0015 carbon_idxs = carbon_idxs(1:3);
0016 
0017 biomass = 1266;
0018 
0019 
0020 tiger.lb(ex_idxs) = 0;
0021 tiger.lb(minimal_idxs) = -1000;
0022 tiger.sense = -1;
0023 
0024 Ncarbon = length(carbon_idxs);
0025 alts.lb = repmat(tiger.lb,1,Ncarbon);
0026 for i = 1 : Ncarbon
0027     alts.lb(carbon_idxs(i),i) = -10;
0028 end
0029 
0030 sols = cmpi.solve_multiple_mips(tiger,alts,'restart',true);
0031 
0032 growth_rates = 0.3 * sols.val;
0033 alts.lb(biomass,:) = growth_rates;
0034 
0035 [~,gene_locs] = convert_ids(tiger.varnames,tiger.genes);
0036 tiger.obj(:) = 0;
0037 tiger.obj(gene_locs) = 1;
0038 tiger.sense = 1;
0039 
0040 cmpi.set_option('Display','off');
0041 cmpi.set_option('AbsOptTol',25);
0042 cmpi.set_option('MaxTime',30);
0043 sols = cmpi.solve_multiple_mips(tiger,alts,'restart',false);
0044 
0045 
0046
```

---

Generated on Thu 11-Aug-2011 15:06:22 by **m2html** © 2005
