# Supplementary material for: TIGER: Toolbox for integrating genome-scale metabolic models, expression data, and transcriptional regulatory networks
Source: BMC Syst Biol. 2011 Sep 23;5:147. doi: 10.1186/1752-0509-5-147 (PMC3224351; doi:10.1186/1752-0509-5-147)
Supplement: Additional file 2 — TIGER source code. Source code, documentation, and tutorials are also available online at http://bme.virginia.edu/csbl/downloads/ or http://csbl.bitbucket.org/tiger. [file 1752-0509-5-147-S2.GZ › tiger/doc/m2html/tiger/test/yeast/run_trn_comparison.html]

Description of run\_trn\_comparison


Home > tiger > test > yeast > run\_trn\_comparison.m

# run\_trn\_comparison

## PURPOSE

## SYNOPSIS

**This is a script file.**

## DESCRIPTION

## CROSS-REFERENCE INFORMATION

This function calls:

- add\_growth\_constraint Add minimum growth constraint to a model.
- fba Run Flux Balance Analysis on a TIGER model.
- solve\_tiger Solve a TIGER model.

This function is called by:


## SOURCE CODE

```
0001 
0002 load ind750.mat
0003 load yeast_trn_iff.mat
0004 changeCobraSolver('cplex');
0005 
0006 is_ex_f = @(x) length(x) >= 3 && strcmp(x(1:3),'EX_');
0007 ex_idxs = find(cellfun(is_ex_f,cobra.rxns));
0008 
0009 minimal_mets = {'h2o(e)','nh4(e)','o2(e)','pi(e)','so4(e)'};
0010 minimal_idxs = [    436,     456,     458,   466,     476 ];
0011 
0012 %carbon_sources = {'glc','gal','fruc','man','sucr','etoh','glyc','lac','pyr','rib','succ'};
0013 %carbon_idxs    = [  428   425    423   451    481    420    432   446   470   471    480];
0014 
0015 carbon_sources = {'glc','gal','fruc','man','sucr','etoh','glyc','pyr','rib','succ'};
0016 carbon_idxs    = [  428   425    423   451    481    420    432   470   471    480];
0017 
0018 ko_names = {   'ADR1',   'CAT8',   'GAL4',   'MIG1',   'MIG2',   'MTH1',   'NRG1',   'RGT1',   'SIP4'};
0019 ko_genes = {'YDR216W','YMR280C','YPL248C','YGL035C','YGL209W','YDR277C','YDR043C','YKL038W','YJL089W'};
0020 
0021 %   WT  adr1  cat8  gal4  mig1  mig2  mth1  nrg1  rgt1  sip4
0022 growth_rates = ...
0023 [ 0.21  0.21  0.21  0.21  0.21  0.20  0.21  0.21  0.21  0.21; ... % glu
0024   0.13  0.13  0.13  0.03  0.12  0.13  0.11  0.14  0.08  0.13; ... % gal
0025   0.20  0.20  0.20  0.20  0.20  0.20  0.21  0.20  0.20  0.20; ... % fruc
0026   0.20  0.20  0.20  0.19  0.17  0.19  0.20  0.19  0.18  0.19; ... % man
0027   0.21  0.21  0.20  0.19  0.20  0.20  0.21  0.20  0.19  0.20; ... % sucr
0028   0.02  0.03  0.01  0.04  0.02  0.03  0.04  0.04  0.01  0.03; ... % etoh
0029   0.02  0.03  0.01  0.04  0.03  0.03  0.03  0.03  0.01  0.02; ... % glyc
0030 %  0.03  0.05  0.01  0.05  0.03  0.05  0.05  0.04  0.02  0.05; ... % lac
0031   0.04  0.05  0.01  0.06  0.05  0.05  0.04  0.04  0.04  0.05; ... % pyr
0032   0.03  0.03  0.01  0.03  0.02  0.03  0.03  0.03  0.01  0.03; ... % rib
0033   0.03  0.04  0.01  0.04  0.04  0.04  0.04  0.03  0.04  0.04  ];  % succ
0034 
0035 imh805_rates = ...
0036 [ 0.21  0.21  0.21  0.21  0.21  0.21  0.21  0.21  0.21  0.21; ...
0037   0.13  0.13  0.13  0.03  0.13  0.13  0.11  0.13  0.09  0.13; ...
0038   0.20  0.20  0.19  0.20  0.20  0.20  0.20  0.20  0.20  0.20; ...
0039   0.19  0.19  0.19  0.19  0.17  0.19  0.19  0.19  0.19  0.19; ...
0040   0.20  0.20  0.20  0.20  0.20  0.20  0.20  0.20  0.20  0.20; ...
0041   0.03  0.03  0.02  0.03  0.03  0.03  0.03  0.03  0.03  0.03; ...
0042   0.03  0.03  0.01  0.03  0.03  0.03  0.03  0.03  0.03  0.03; ...
0043 %  0.05  0.05  0.01  0.05  0.05  0.05  0.05  0.05  0.05  0.05; ...
0044   0.05  0.05  0.01  0.05  0.05  0.05  0.05  0.05  0.05  0.05; ...
0045   0.03  0.03  0.02  0.03  0.03  0.03  0.03  0.03  0.00  0.03; ...
0046   0.04  0.04  0.02  0.04  0.04  0.04  0.04  0.04  0.04  0.04  ];
0047   
0048 
0049 %%
0050 
0051 uptake_rates = zeros(size(carbon_idxs));
0052 
0053 trn_con = add_growth_constraint(trn,0,'valtype','abs','ctype','=');
0054 
0055 for i = 1 : length(carbon_sources)
0056     trn_con.lb(ex_idxs) = 0;
0057     trn_con.lb(minimal_idxs) = -1000;
0058     source = carbon_idxs(i);
0059     trn_con.obj(:) = 0;
0060     trn_con.obj(source) = -1;
0061     trn_con.lb(source) = -100;
0062     trn_con.b(end) = growth_rates(i,1);
0063     sol = solve_tiger(trn_con);
0064     if ~isempty(sol.x)
0065         uptake_rates(i) = sol.x(source);
0066     end
0067 end
0068 
0069 %%
0070 tiger_rates = zeros(size(growth_rates));
0071 for carbon = 1 : length(carbon_sources)
0072     tiger = trn;
0073     tiger.lb(carbon_idxs) = 0;
0074     tiger.lb(carbon_idxs(carbon)) = uptake_rates(carbon);
0075     sol = fba(tiger);
0076     tiger_rates(carbon,1) = sol.val;
0077     for i = 1 : length(ko_genes)
0078         [tf,loc] = ismember(ko_genes{i},tiger.varnames);
0079         if tf
0080             disp(['knocking out ' ko_genes{i} ' in ' carbon_sources{carbon}])
0081             tiger.ub(loc) = 0;
0082             sol = fba(tiger);
0083             if ~isempty(sol.val)
0084                 tiger_rates(carbon,1+i) = sol.val;
0085             end
0086         else
0087             tiger_rates(carbon,1+i) = -1;
0088         end
0089     end
0090 end
0091 
0092 %%
0093 
0094 imh805_error = abs(growth_rates - imh805_rates) ./ growth_rates;
0095 tiger_error  = abs(growth_rates - tiger_rates) ./ growth_rates;
0096 
0097 sum(imh805_error(:).^2)
0098 sum(tiger_error(:).^2)
0099 
0100 length(find(imh805_error(:) > 1e-5))
0101 length(find(tiger_error(:) > 1e-5))
0102 
0103 imh805_error - tiger_error
0104
```

---

Generated on Thu 11-Aug-2011 15:06:22 by **m2html** © 2005
