# Supplementary material for: TIGER: Toolbox for integrating genome-scale metabolic models, expression data, and transcriptional regulatory networks
Source: BMC Syst Biol. 2011 Sep 23;5:147. doi: 10.1186/1752-0509-5-147 (PMC3224351; doi:10.1186/1752-0509-5-147)
Supplement: Additional file 2 — TIGER source code. Source code, documentation, and tutorials are also available online at http://bme.virginia.edu/csbl/downloads/ or http://csbl.bitbucket.org/tiger. [file 1752-0509-5-147-S2.GZ › tiger/doc/m2html/tiger/@cmpi/tile_milp.html]

Description of tile\_milp


Home > tiger > @cmpi > tile\_milp.m

# tile\_milp

## PURPOSE

**Combine several MILPs into a single structure**

## SYNOPSIS

**function [milp] = tile\_milp(varargin)**

## DESCRIPTION

```
 TILE_MILP  Combine several MILPs into a single structure

   [MILP] = TILE_MILP(MILP1,MILP2,...)

   Combines N MILP problem structures into a single structure.  The
   resulting A matrix is a block-diagonal tiling of the individual A
   matrices.

   Variable names for each problem are appended with '[i]'.  For example,
   if three structures were given, each with a variable 'x', the 
   resulting variable names would be 'x[1]', 'x[2]', and 'x[3]'.
```

## CROSS-REFERENCE INFORMATION

This function calls:

- celliter Iterate over elements in a cell
- map

This function is called by:

- cmpi

## SUBFUNCTIONS

- function concat\_fields(name)

## SOURCE CODE

```
0001 function [milp] = tile_milp(varargin)
0002 % TILE_MILP  Combine several MILPs into a single structure
0003 %
0004 %   [MILP] = TILE_MILP(MILP1,MILP2,...)
0005 %
0006 %   Combines N MILP problem structures into a single structure.  The
0007 %   resulting A matrix is a block-diagonal tiling of the individual A
0008 %   matrices.
0009 %
0010 %   Variable names for each problem are appended with '[i]'.  For example,
0011 %   if three structures were given, each with a variable 'x', the
0012 %   resulting variable names would be 'x[1]', 'x[2]', and 'x[3]'.
0013 
0014 % format string used for creating model-specific variable names
0015 VARNAME_FMT = '%s[%i]';
0016 
0017 N = length(varargin);
0018 milps = map(@cmpi.check_mip,varargin);
0019 
0020 if N == 0
0021     error('at least one MILP must be provided');
0022 end
0023 
0024 if N == 1
0025     milp = milps{1};
0026     return;
0027 end
0028 
0029 % shift the indicator indices
0030 ns = cellfun(@(x) size(x.A,2),milps);
0031 offsets = cumsum(ns) - ns(1);
0032 for i = 1 : N
0033     milps{i}.ind = milps{i}.ind + offsets(i);
0034 end
0035 
0036 As = map(@(x) x.A,milps);
0037 milp.A = blkdiag(As{:});
0038 
0039 % make sure all MILPs have the same sense as the first MILP
0040 if isfield(milps{1},'sense')
0041     milp.sense = milps{1}.sense;
0042 else
0043     milp.sense = 1;
0044 end
0045 for i = 2 : N
0046     if isfield(milps{i},'sense') && milps{i}.sense ~= milps{1}.sense
0047         milps{i}.obj = -1*milps{i}.obj;
0048     end
0049 end
0050 
0051 % create model-specific variable names
0052 for i = 1 : N
0053     milps{i}.varnames = map(@(x) sprintf(VARNAME_FMT,x,i), ...
0054                             milps{i}.varnames);
0055 end
0056 
0057 % concatenate all other fields
0058 field_names = {'obj','b','ctypes','vartypes','lb','ub', ...
0059                'ind','indtypes','rownames','varnames'};
0060 celliter(@concat_fields,field_names);
0061 
0062 milp.options = milps{1}.options;
0063 
0064 % start over the indicator counter
0065 milp.param.ind = 0;
0066 
0067 function concat_fields(name)
0068     fields = map(@(x) x.(name),milps);
0069     milp.(name) = vertcat(fields{:});
0070 end
0071 
0072 end
```

---

Generated on Tue 02-Aug-2011 16:03:37 by **m2html** © 2005
