# Supplementary material for: TIGER: Toolbox for integrating genome-scale metabolic models, expression data, and transcriptional regulatory networks
Source: BMC Syst Biol. 2011 Sep 23;5:147. doi: 10.1186/1752-0509-5-147 (PMC3224351; doi:10.1186/1752-0509-5-147)
Supplement: Additional file 2 — TIGER source code. Source code, documentation, and tutorials are also available online at http://bme.virginia.edu/csbl/downloads/ or http://csbl.bitbucket.org/tiger. [file 1752-0509-5-147-S2.GZ › tiger/doc/m2html/tiger/@cmpi/verify_sol.html]

Description of verify\_sol


Home > tiger > @cmpi > verify\_sol.m

# verify\_sol

## PURPOSE

**Verify a solution is feasible**

## SYNOPSIS

**function [tf] = verify\_sol(mip,sol,tol)**

## DESCRIPTION

```
 VERIFY_SOL  Verify a solution is feasible

   [TF] = VERIFY_SOL(MIP,SOL,TOL)

   Verifies that the solution structure SOL satisfies the constraints
   in MIP to within tolerance TOL.  The default tolerance is 1e-8.
```

## CROSS-REFERENCE INFORMATION

This function calls:


This function is called by:

- cmpi

## SOURCE CODE

```
0001 function [tf] = verify_sol(mip,sol,tol)
0002 % VERIFY_SOL  Verify a solution is feasible
0003 %
0004 %   [TF] = VERIFY_SOL(MIP,SOL,TOL)
0005 %
0006 %   Verifies that the solution structure SOL satisfies the constraints
0007 %   in MIP to within tolerance TOL.  The default tolerance is 1e-8.
0008 
0009 if nargin < 3,  tol = 1e-8; end
0010 
0011 switch class(sol)
0012     case 'double'
0013         x = sol(:);
0014     case 'struct'
0015         x = sol.x(:);
0016     otherwise
0017         error(['invalid class for argument sol: ' class(sol)]);
0018 end
0019 
0020 resid = mip.A * x - mip.b(:);
0021 
0022 leq = (mip.ctypes == '<');
0023 geq = (mip.ctypes == '>');
0024 eq  = (mip.ctypes == '=');
0025 
0026 tf = all(resid(leq) <= tol) ...
0027         && all(resid(geq) >= -tol) ...
0028         && all( abs(resid(eq)) <= tol );
0029 
0030 tf = tf && ( all(x <= mip.ub(:) + tol) ...
0031                 && all(x >= mip.lb(:) - tol) );
0032
```

---

Generated on Thu 11-Aug-2011 15:06:22 by **m2html** © 2005
