# Supplementary material for: TIGER: Toolbox for integrating genome-scale metabolic models, expression data, and transcriptional regulatory networks
Source: BMC Syst Biol. 2011 Sep 23;5:147. doi: 10.1186/1752-0509-5-147 (PMC3224351; doi:10.1186/1752-0509-5-147)
Supplement: Additional file 2 — TIGER source code. Source code, documentation, and tutorials are also available online at http://bme.virginia.edu/csbl/downloads/ or http://csbl.bitbucket.org/tiger. [file 1752-0509-5-147-S2.GZ › tiger/doc/m2html/tiger/@cmpi/index.html]

Index for Directory tiger/@cmpi


|  |  |
| --- | --- |
| Master index | Index for tiger/@cmpi |

# Index for tiger/@cmpi

## Matlab files in this directory:

|  |  |
| --- | --- |
| add\_obj\_constraint | Add minimal objective constraint to a MILP |
| check\_field | Check if a field exists in a structure. |
| check\_mip | Ensure that the sense, ind, and indtypes fields are filled |
| cmpi |  |
| convert\_indicators | Convert indicators to MILP constraints |
| convert\_miqp | Prepare a MIQP for solution |
| get\_cplex\_flag | Convert a CPLEX flag to a CMPI flag |
| miqp\_type | Return the quadratic type of a MIQP problem |
| set\_cplex\_opts | Convert a CMPI options structure into CPLEX opts |
| set\_gurobi\_opts | Convert a CMPI options structure into GUROBI opts |
| show\_mip | Show equations for a MIP structure |
| solve\_mip | Solve a Mixed Integer Programming problem |
| solve\_multiple\_mips |  |
| tile\_mip | Combine several MIPs into a single structure |
| verify\_sol | Verify a solution is feasible |

---

Generated on Thu 11-Aug-2011 15:06:20 by **m2html** © 2005
