# Supplementary material for: TIGER: Toolbox for integrating genome-scale metabolic models, expression data, and transcriptional regulatory networks
Source: BMC Syst Biol. 2011 Sep 23;5:147. doi: 10.1186/1752-0509-5-147 (PMC3224351; doi:10.1186/1752-0509-5-147)
Supplement: Additional file 2 — TIGER source code. Source code, documentation, and tutorials are also available online at http://bme.virginia.edu/csbl/downloads/ or http://csbl.bitbucket.org/tiger. [file 1752-0509-5-147-S2.GZ › tiger/doc/m2html/tiger/@cmpi/tile_mip.html]

Description of tile\_mip


Home > tiger > @cmpi > tile\_mip.m

# tile\_mip

## PURPOSE

**Combine several MIPs into a single structure**

## SYNOPSIS

**function [mip] = tile\_mip(varargin)**

## DESCRIPTION

```
 TILE_MIP  Combine several MIPs into a single structure

   [MIP] = TILE_MIP(MIP1,MIP2,...)

   Combines N MIP problem structures into a single structure.  The
   resulting A matrix is a block-diagonal tiling of the individual A
   matrices.

   Variable names for each problem are appended with '[i]'.  For example,
   if three structures were given, each with a variable 'x', the 
   resulting variable names would be 'x[1]', 'x[2]', and 'x[3]'.
```

## CROSS-REFERENCE INFORMATION

This function calls:

- celliter Iterate over elements in a cell
- map Generate a new list by applying a function

This function is called by:

- cmpi

## SUBFUNCTIONS

- function concat\_fields(name)

## SOURCE CODE

```
0001 function [mip] = tile_mip(varargin)
0002 % TILE_MIP  Combine several MIPs into a single structure
0003 %
0004 %   [MIP] = TILE_MIP(MIP1,MIP2,...)
0005 %
0006 %   Combines N MIP problem structures into a single structure.  The
0007 %   resulting A matrix is a block-diagonal tiling of the individual A
0008 %   matrices.
0009 %
0010 %   Variable names for each problem are appended with '[i]'.  For example,
0011 %   if three structures were given, each with a variable 'x', the
0012 %   resulting variable names would be 'x[1]', 'x[2]', and 'x[3]'.
0013 
0014 % format string used for creating model-specific variable names
0015 VARNAME_FMT = '%s[%i]';
0016 
0017 N = length(varargin);
0018 mips = map(@cmpi.check_mip,varargin);
0019 
0020 if N == 0
0021     error('at least one mip must be provided');
0022 end
0023 
0024 % copy all parameters
0025 mip = mips{1};
0026 
0027 if N == 1
0028     return;
0029 end
0030 
0031 % shift the indicator indices
0032 ns = cellfun(@(x) size(x.A,2),mips);
0033 offsets = cumsum(ns) - ns(1);
0034 for i = 1 : N
0035     nonzero = mips{i}.ind > 0;
0036     mips{i}.ind(nonzero) = mips{i}.ind(nonzero) + offsets(i);
0037 end
0038 
0039 As = map(@(x) x.A,mips);
0040 mip.A = blkdiag(As{:});
0041 
0042 % make sure all mips have the same sense as the first mip
0043 if isfield(mips{1},'sense')
0044     mip.sense = mips{1}.sense;
0045 else
0046     mip.sense = 1;
0047 end
0048 for i = 2 : N
0049     if isfield(mips{i},'sense') && mips{i}.sense ~= mips{1}.sense
0050         mips{i}.obj = -1*mips{i}.obj;
0051     end
0052 end
0053 
0054 % create model-specific variable names
0055 for i = 1 : N
0056     mips{i}.varnames = map(@(x) sprintf(VARNAME_FMT,x,i), ...
0057                             mips{i}.varnames);
0058 end
0059 
0060 % concatenate all other fields
0061 field_names = {'obj','b','ctypes','vartypes','lb','ub', ...
0062                'ind','indtypes','rownames','varnames'};
0063 celliter(@concat_fields,field_names);
0064 
0065 mip.options = mips{1}.options;
0066 
0067 % start over the indicator counter
0068 mip.param.ind = 0;
0069 
0070 % convert the quadratic terms
0071 qptype = cmpi.miqp_type(mips{1});
0072 if ~isempty(qptype)
0073     switch cmpi.miqp_type(mips{1})
0074         case 'Q'
0075             Qs = map(@(x) x.Q,mips);
0076             mip.Q = blkdiag(Qs{:});
0077         case 'Qd'
0078             Qds = map(@(x) x.Qd,mips);
0079             mip.Qd = blkdiag(Qds{:});
0080         case 'Qc'
0081             Qcws = map(@(x) x.Qc.w(:),mips);
0082             Qccs = map(@(x) x.Qc.c(:),mips);
0083             mip.Qc.w = vertcat(Qcws{:});
0084             mip.Qc.c = vertcat(Qccs{:});
0085     end
0086 end
0087         
0088 
0089 function concat_fields(name)
0090     fields = map(@(x) x.(name),mips);
0091     mip.(name) = vertcat(fields{:});
0092 end
0093 
0094 end
```

---

Generated on Thu 11-Aug-2011 15:06:22 by **m2html** © 2005
