# Supplementary material for: TIGER: Toolbox for integrating genome-scale metabolic models, expression data, and transcriptional regulatory networks
Source: BMC Syst Biol. 2011 Sep 23;5:147. doi: 10.1186/1752-0509-5-147 (PMC3224351; doi:10.1186/1752-0509-5-147)
Supplement: Additional file 2 — TIGER source code. Source code, documentation, and tutorials are also available online at http://bme.virginia.edu/csbl/downloads/ or http://csbl.bitbucket.org/tiger. [file 1752-0509-5-147-S2.GZ › tiger/doc/m2html/tiger/@cmpi/set_cplex_opts.html]

Description of set\_cplex\_opts


Home > tiger > @cmpi > set\_cplex\_opts.m

# set\_cplex\_opts

## PURPOSE

**Convert a CMPI options structure into CPLEX opts**

## SYNOPSIS

**function [opts,cplex] = set\_cplex\_opts(options,cplex)**

## DESCRIPTION

```
 SET_CPLEX_OPTS  Convert a CMPI options structure into CPLEX opts
```

## CROSS-REFERENCE INFORMATION

This function calls:


This function is called by:

- cmpi

## SUBFUNCTIONS

- function setifdef(cmpifield,cplexfield)

## SOURCE CODE

```
0001 function [opts,cplex] = set_cplex_opts(options,cplex)
0002 % SET_CPLEX_OPTS  Convert a CMPI options structure into CPLEX opts
0003 
0004 if nargin > 1
0005     opts = [];
0006     
0007     if isfield(options,'MaxTime')
0008         cplex.Param.timelimit.Cur = options.MaxTime;
0009     end
0010     if isfield(options,'MaxIter')
0011         cplex.mip.limits.solutions.Cur = options.MaxIter;
0012     end
0013     if isfield(options,'MaxNodes')
0014         cplex.Param.mip.limits.nodes.Cur = options.MaxNodes;
0015     end
0016     if isfield(options,'FeasTol')
0017         cplex.Param.simplex.tolerances.feasibility.Cur = options.FeasTol;
0018     end
0019     if isfield(options,'IntFeasTol')
0020         cplex.Param.mip.tolerances.integrality.Cur = options.IntFeasTol;
0021     end
0022     if isfield(options,'OptTol')
0023         cplex.Param.mip.tolerances.mipgap.Cur = options.OptTol;
0024     end
0025     if isfield(options,'AbsOptTol')
0026         cplex.Param.mip.tolerances.absmipgap.Cur = options.AbsOptTol;
0027     end
0028 else
0029     cplex = [];
0030     
0031     opts = cplexoptimset;
0032     setifdef('MaxTime','MaxTime');
0033     setifdef('MaxIter','MaxIter');
0034     setifdef('MaxNodes','MaxNodes');
0035     setifdef('FeasTol','EpRHS');
0036     setifdef('IntFeasTol','TolXInteger');
0037     setifdef('OptTol','EpGap');
0038     setifdef('Display','Diagnostics');
0039     if isfield(options,'Display') && strcmpi(options.Display,'on')
0040         opts.Display = 'iter';
0041     end
0042 end
0043     
0044 function setifdef(cmpifield,cplexfield)
0045     if isfield(options,cmpifield)
0046         opts.(cplexfield) = options.(cmpifield);
0047     end
0048 end
0049 
0050 end
```

---

Generated on Thu 11-Aug-2011 15:06:22 by **m2html** © 2005
