# Supplementary material for: TIGER: Toolbox for integrating genome-scale metabolic models, expression data, and transcriptional regulatory networks
Source: BMC Syst Biol. 2011 Sep 23;5:147. doi: 10.1186/1752-0509-5-147 (PMC3224351; doi:10.1186/1752-0509-5-147)
Supplement: Additional file 2 — TIGER source code. Source code, documentation, and tutorials are also available online at http://bme.virginia.edu/csbl/downloads/ or http://csbl.bitbucket.org/tiger. [file 1752-0509-5-147-S2.GZ › tiger/doc/m2html/tiger/@cmpi/solve_milp.html]

Description of solve\_milp


Home > tiger > @cmpi > solve\_milp.m

# solve\_milp

## PURPOSE

**Solve a Mixed Integer Linear Programming problem**

## SYNOPSIS

**function [sol] = solve\_milp(milp,solver)**

## DESCRIPTION

```
 SOLVE_MILP  Solve a Mixed Integer Linear Programming problem

   [SOL] = SOLVE_MILP(MILP,SOLVER)

   Solves the Mixed Integer Linear Programming problem
       min sense*obj*x
       subject to
           A*x (<=/=/>=) b
           lb <= x <= ub

   Inputs
   MILP    Problem structure.  Fields include:
               obj       Objective coefficients
               sense     Direction of optimization:
                              1    Minimization
                             -1    Maximization
               A         Coefficient matrix for constraints.
               b         Right-hand side of constraints.
               ctypes    Char array denoting the type of each constraint:
                             '<'   a*x <= b
                             '='   a*x == b
                             '>'   a*x >= b
               lb        Lower bound for each variable.
               ub        Upper bound for each variable.
               vartypes  Char array denoting the type of each variable:
                             'C'   Continuous
                             'B'   Binary
                             'I'   Integer
               options   Solver options (see below).  If not given, the
                         options in the global variable CMPI_OPTIONS are
                         used (if defined).
                             MaxTime     Maximum solution time (seconds)
                             MaxIter     Maximum simplex iterations
                             MaxNodes    Maximum number of nodes
                             Display     Turn reporting to the screen
                                         'on' or 'off' (default)
                             FeasTol     Feasibility tolerance
                             IntFeasTol  Integer feasibility tolerance
                             OptTol      Optimality tolerance
   SOLVER  Solver to use.  If not given, the value of the global
           variable CMPI_SOLVER is used.

   Outputs
   SOL     Solution structure with fields:
               x       Optimal vector
               val     Objective values (sense*c*x)
               flag    Exit flag.  Possible values are:
                           1   Not started
                           2   Optimal
                           3   Infeasible
                           4   Infeasible or unbounded
                           5   Unbounded
                           6   Objective worse than user cutoff
                           7   Iteration limit reached
                           8   Node limit reached
                           9   Time limit reached
                           10  Solution limit reached
                           11  User interruption
                           12  Numerical difficulties
                           13  Suboptimal solution
               output  Other solver-specific output
```

## CROSS-REFERENCE INFORMATION

This function calls:

- check\_field Check if a field exists in a structure.
- get\_cplex\_flag Convert a CPLEX flag to a CMPI flag

This function is called by:


## SOURCE CODE

```
0001 function [sol] = solve_milp(milp,solver)
0002 % SOLVE_MILP  Solve a Mixed Integer Linear Programming problem
0003 %
0004 %   [SOL] = SOLVE_MILP(MILP,SOLVER)
0005 %
0006 %   Solves the Mixed Integer Linear Programming problem
0007 %       min sense*obj*x
0008 %       subject to
0009 %           A*x (<=/=/>=) b
0010 %           lb <= x <= ub
0011 %
0012 %   Inputs
0013 %   MILP    Problem structure.  Fields include:
0014 %               obj       Objective coefficients
0015 %               sense     Direction of optimization:
0016 %                              1    Minimization
0017 %                             -1    Maximization
0018 %               A         Coefficient matrix for constraints.
0019 %               b         Right-hand side of constraints.
0020 %               ctypes    Char array denoting the type of each constraint:
0021 %                             '<'   a*x <= b
0022 %                             '='   a*x == b
0023 %                             '>'   a*x >= b
0024 %               lb        Lower bound for each variable.
0025 %               ub        Upper bound for each variable.
0026 %               vartypes  Char array denoting the type of each variable:
0027 %                             'C'   Continuous
0028 %                             'B'   Binary
0029 %                             'I'   Integer
0030 %               options   Solver options (see below).  If not given, the
0031 %                         options in the global variable CMPI_OPTIONS are
0032 %                         used (if defined).
0033 %                             MaxTime     Maximum solution time (seconds)
0034 %                             MaxIter     Maximum simplex iterations
0035 %                             MaxNodes    Maximum number of nodes
0036 %                             Display     Turn reporting to the screen
0037 %                                         'on' or 'off' (default)
0038 %                             FeasTol     Feasibility tolerance
0039 %                             IntFeasTol  Integer feasibility tolerance
0040 %                             OptTol      Optimality tolerance
0041 %   SOLVER  Solver to use.  If not given, the value of the global
0042 %           variable CMPI_SOLVER is used.
0043 %
0044 %   Outputs
0045 %   SOL     Solution structure with fields:
0046 %               x       Optimal vector
0047 %               val     Objective values (sense*c*x)
0048 %               flag    Exit flag.  Possible values are:
0049 %                           1   Not started
0050 %                           2   Optimal
0051 %                           3   Infeasible
0052 %                           4   Infeasible or unbounded
0053 %                           5   Unbounded
0054 %                           6   Objective worse than user cutoff
0055 %                           7   Iteration limit reached
0056 %                           8   Node limit reached
0057 %                           9   Time limit reached
0058 %                           10  Solution limit reached
0059 %                           11  User interruption
0060 %                           12  Numerical difficulties
0061 %                           13  Suboptimal solution
0062 %               output  Other solver-specific output
0063 
0064 warning('This function is deprecated.  Use SOLVE_MIP instead.');
0065 
0066 if nargin < 2,  solver = cmpi.get_solver(); end
0067 
0068 if ~issparse(milp.A)
0069     milp.A = sparse(milp.A);
0070 end
0071 
0072 if ~isfield(milp,'options')
0073     milp.options = cmpi.get_options();
0074 end
0075 
0076 switch solver
0077     case 'gurobi'
0078         [sol.x, sol.val, sol.flag, sol.output] = ...
0079             gurobi_mex(milp.obj, ...
0080                        milp.sense, ...
0081                        milp.A, ...
0082                        milp.b, ...
0083                        milp.ctypes, ...
0084                        milp.lb, ...
0085                        milp.ub, ...
0086                        milp.vartypes, ...
0087                        milp.options);
0088         
0089     case 'ilog_cplex'        
0090         opts = cmpi.set_cplex_opts(milp.options);
0091         
0092         milp.b = milp.b(:);
0093 
0094         Aineq = [ milp.A(milp.ctypes == '<',:); 
0095                  -milp.A(milp.ctypes == '>',:)];
0096         bineq = [ milp.b(milp.ctypes == '<');
0097                  -milp.b(milp.ctypes == '>')];
0098              
0099         Aeq = milp.A(milp.ctypes == '=',:);
0100         beq = milp.b(milp.ctypes == '=');
0101         
0102         [sol.x,sol.val,~,sol.output] = ...
0103             cplexmilp(milp.sense*milp.obj(:), ...
0104                       Aineq, bineq, ...
0105                       Aeq, beq, ...
0106                       [], [], [], ...
0107                       milp.lb(:), milp.ub(:), ...
0108                       milp.vartypes, ...
0109                       [], ...
0110                       opts);
0111 
0112         sol.val = milp.sense*sol.val;
0113                    
0114         sol.flag = cmpi.get_cplex_flag(sol.output.cplexstatus);
0115 
0116     case 'cplex_multi'
0117         cplex = Cplex();
0118         if milp.sense == 1
0119            cplex.Model.sense = 'minimize';
0120         else
0121            cplex.Model.sense = 'maximize';
0122         end
0123         cplex.Model.obj = milp.obj(:);
0124         milp.A(milp.ctypes == '>',:) = -milp.A(milp.ctypes == '>',:);
0125         milp.b(milp.ctypes == '>') = -milp.b(milp.ctypes == '>');
0126         cplex.Model.A = milp.A;
0127         cplex.Model.ctype = milp.vartypes;
0128         cplex.Model.lb = milp.lb(:);
0129         cplex.Model.ub = milp.ub(:);
0130         cplex.Model.rhs = milp.b(:);
0131         lhs = -inf*ones(size(milp.b(:)));
0132         %lhs(:) = -inf;
0133         lhs(milp.ctypes == '=') = milp.b(milp.ctypes == '=');
0134         cplex.Model.lhs = lhs;
0135         
0136         cplex.writeModel('prob.lp');
0137         
0138         cplex.solve();
0139         sol.x = check_field('x', cplex.Solution);
0140         sol.flag = get_cplex_flag(cplex.Solution.status);
0141         sol.val = check_field('objval', cplex.Solution);
0142         sol.output = cplex.Solution.statusstring;
0143         
0144         cplex.Param.mip.pool.relgap.Cur = 1000.0;
0145         cplex.populate();
0146         sol.pool = check_field('solution', cplex.Solution.pool);
0147 
0148     otherwise
0149         error('Unrecognized solver: %s',solver);
0150 end
0151 
0152 end
0153 
0154 
0155
```

---

Generated on Tue 02-Aug-2011 16:03:37 by **m2html** © 2005
