# Supplementary material for: TIGER: Toolbox for integrating genome-scale metabolic models, expression data, and transcriptional regulatory networks
Source: BMC Syst Biol. 2011 Sep 23;5:147. doi: 10.1186/1752-0509-5-147 (PMC3224351; doi:10.1186/1752-0509-5-147)
Supplement: Additional file 2 — TIGER source code. Source code, documentation, and tutorials are also available online at http://bme.virginia.edu/csbl/downloads/ or http://csbl.bitbucket.org/tiger. [file 1752-0509-5-147-S2.GZ › tiger/doc/m2html/tiger/@cmpi/check_mip.html]

Description of check\_mip


Home > tiger > @cmpi > check\_mip.m

# check\_mip

## PURPOSE

**Ensure that the sense, ind, and indtypes fields are filled**

## SYNOPSIS

**function [mip] = check\_mip(mip)**

## DESCRIPTION

```
 CHECK_MIP  Ensure that the sense, ind, and indtypes fields are filled

   [MIP] = CHECK_MIP(MIP)

   Checks that the fields 'sense', 'ind', and 'indtypes' fields exist 
   and are the correct size.  Returns the valided MIP.
```

## CROSS-REFERENCE INFORMATION

This function calls:

- expand\_to Expand a vector to length N
- fill\_to Fill a short to empty vector

This function is called by:

- cmpi

## SUBFUNCTIONS

- function set\_if\_not\_field(name,val)

## SOURCE CODE

```
0001 function [mip] = check_mip(mip)
0002 % CHECK_MIP  Ensure that the sense, ind, and indtypes fields are filled
0003 %
0004 %   [MIP] = CHECK_MIP(MIP)
0005 %
0006 %   Checks that the fields 'sense', 'ind', and 'indtypes' fields exist
0007 %   and are the correct size.  Returns the valided MIP.
0008 
0009 m = size(mip.A,1);
0010 
0011 set_if_not_field('sense',1);
0012 set_if_not_field('ind',zeros(m,1));
0013 set_if_not_field('indtypes',repmat(' ',m,1));
0014 set_if_not_field('options',[]);
0015 
0016 if length(mip.ind) < m
0017     mip.ind = expand_to(mip.ind,m);
0018 end
0019 if length(mip.indtypes) < m
0020     mip.indtypes = fill_to(mip.ind,m,' ');
0021 end
0022 
0023 function set_if_not_field(name,val)
0024     if ~isfield(mip,name)
0025         mip.(name) = val;
0026     end
0027 end
0028 
0029 end
```

---

Generated on Thu 11-Aug-2011 15:06:22 by **m2html** © 2005
