# Supplementary material for: TIGER: Toolbox for integrating genome-scale metabolic models, expression data, and transcriptional regulatory networks
Source: BMC Syst Biol. 2011 Sep 23;5:147. doi: 10.1186/1752-0509-5-147 (PMC3224351; doi:10.1186/1752-0509-5-147)
Supplement: Additional file 2 — TIGER source code. Source code, documentation, and tutorials are also available online at http://bme.virginia.edu/csbl/downloads/ or http://csbl.bitbucket.org/tiger. [file 1752-0509-5-147-S2.GZ › tiger/doc/m2html/tiger/@cmpi/set_gurobi_opts.html]

Description of set\_gurobi\_opts


Home > tiger > @cmpi > set\_gurobi\_opts.m

# set\_gurobi\_opts

## PURPOSE

**Convert a CMPI options structure into GUROBI opts**

## SYNOPSIS

**function [opts] = set\_gurobi\_opts(options)**

## DESCRIPTION

```
 SET_GUROBI_OPTS  Convert a CMPI options structure into GUROBI opts
```

## CROSS-REFERENCE INFORMATION

This function calls:


This function is called by:

- cmpi

## SOURCE CODE

```
0001 function [opts] = set_gurobi_opts(options)
0002 % SET_GUROBI_OPTS  Convert a CMPI options structure into GUROBI opts
0003 
0004 if isfield(options,'MaxTime')
0005     opts.TimeLimit = options.MaxTime;
0006 end
0007 if isfield(options,'MaxIter')
0008     opts.IterationLimit = options.MaxIter;
0009 end
0010 if isfield(options,'MaxNodes')
0011     opts.NodeLimit = options.MaxNodes;
0012 end
0013 if isfield(options,'FeasTol')
0014     opts.FeasibilityTol = options.FeasTol;
0015 end
0016 if isfield(options,'IntFeasTol')
0017     opts.IntFeasTol = options.IntFeasTol;
0018 end
0019 if isfield(options,'OptTol')
0020     opts.OptimalityTol = options.OptTol;
0021 end
0022 if isfield(options,'AbsOptTol')
0023     opts.MIPGapAbs = options.AbsOptTol;
0024 end
0025 
0026 if isfield(options,'Display') && strcmpi(options.Display,'on')
0027     opts.DisplayInterval = 5;
0028     opts.OutputFlag = 1;
0029     opts.Display = 2;
0030 else
0031     opts.DisplayInterval = 0;
0032     opts.OutputFlag = 0;
0033     opts.Display = 0;
0034 end
```

---

Generated on Thu 11-Aug-2011 15:06:22 by **m2html** © 2005
