# Supplementary material for: TIGER: Toolbox for integrating genome-scale metabolic models, expression data, and transcriptional regulatory networks
Source: BMC Syst Biol. 2011 Sep 23;5:147. doi: 10.1186/1752-0509-5-147 (PMC3224351; doi:10.1186/1752-0509-5-147)
Supplement: Additional file 2 — TIGER source code. Source code, documentation, and tutorials are also available online at http://bme.virginia.edu/csbl/downloads/ or http://csbl.bitbucket.org/tiger. [file 1752-0509-5-147-S2.GZ › tiger/doc/m2html/tiger/@cmpi/convert_indicators.html]

Description of convert\_indicators


Home > tiger > @cmpi > convert\_indicators.m

# convert\_indicators

## PURPOSE

**Convert indicators to MILP constraints**

## SYNOPSIS

**function [mip] = convert\_indicators(mip)**

## DESCRIPTION

```
 CONVERT_INDICATORS  Convert indicators to MILP constraints

   [MIP] = CONVERT_INDICATORS(MIP)

   Converts the indicators indentified by the 'ind' and 'indtypes' fields
   to MILP constraints.  The constraints generated depend on the 
   indicator types:
       'p'  Ax (<=/=/>=) b  => I = 1  becomes  Ax + s (<=/=/>=) b
                                               I + s >= IND_EPS

       'b'  Ax (<=/=/>=) b <=> I = 1  becomes  Ax + s (<=/=/>=) b
                                               I + s >= IND_EPS
                                               s_lb*(1-I) <= s
                                               s <= s_ub*(1-I)

   The function adds a slack variable s to each constraint that is tied 
   to an indicator and either 1 or 3 constraints per indicator, depending
   on if the indicator was a positive ('p') or bound ('b') indicator.

   The CMPI setting IND_EPS is used to construct one of the constraints
   for each indicator.
```

## CROSS-REFERENCE INFORMATION

This function calls:

- add\_column Add a column to a TIGER model structure
- add\_row Add a row to a TIGER model structure

This function is called by:

- cmpi

## SOURCE CODE

```
0001 function [mip] = convert_indicators(mip)
0002 % CONVERT_INDICATORS  Convert indicators to MILP constraints
0003 %
0004 %   [MIP] = CONVERT_INDICATORS(MIP)
0005 %
0006 %   Converts the indicators indentified by the 'ind' and 'indtypes' fields
0007 %   to MILP constraints.  The constraints generated depend on the
0008 %   indicator types:
0009 %       'p'  Ax (<=/=/>=) b  => I = 1  becomes  Ax + s (<=/=/>=) b
0010 %                                               I + s >= IND_EPS
0011 %
0012 %       'b'  Ax (<=/=/>=) b <=> I = 1  becomes  Ax + s (<=/=/>=) b
0013 %                                               I + s >= IND_EPS
0014 %                                               s_lb*(1-I) <= s
0015 %                                               s <= s_ub*(1-I)
0016 %
0017 %   The function adds a slack variable s to each constraint that is tied
0018 %   to an indicator and either 1 or 3 constraints per indicator, depending
0019 %   on if the indicator was a positive ('p') or bound ('b') indicator.
0020 %
0021 %   The CMPI setting IND_EPS is used to construct one of the constraints
0022 %   for each indicator.
0023 
0024 IND_EPS = cmpi.get_ind_eps();
0025 
0026 mip.ind = cmpi.check_field('ind',mip);
0027 mip.indtypes = cmpi.check_field('indtypes',mip);
0028 
0029 idx_p = find((mip.ind > 0) & (mip.indtypes == 'p'));
0030 idx_b = find((mip.ind > 0) & (mip.indtypes == 'b'));
0031 
0032 np = length(idx_p);
0033 nb = length(idx_b);
0034 N = np + nb;
0035 
0036 if N == 0
0037     return;
0038 end
0039 
0040 % ctypes '=' and '<' should have been removed in add_rule
0041 if any(mip.ctypes([idx_p idx_b]) == '=')
0042     warning('Indicators Ax = b not supported.');
0043 end
0044 
0045 % switch Ax <= b to -Ax >= -b
0046 leq_rows = intersect(find(mip.ctypes == '<'),[idx_p idx_b]);
0047 mip.A(leq_rows,:) = -mip.A(leq_rows,:);
0048 mip.b(leq_rows) = -mip.b(leq_rows);
0049 mip.ctypes(leq_rows) = '>';
0050     
0051 [m,n] = size(mip.A);
0052 
0053 i = [idx_b idx_p];
0054 j = 1:N;
0055 s = ones(1,N);
0056 IA = sparse(i,j,s,m,N,N);
0057 
0058 s_ub = zeros(1,N);
0059 s_lb = zeros(1,N);
0060 
0061 % find bounds for each slack variable
0062 for k = 1 : N
0063     Aup = mip.A(i(k),:) .* mip.ub';
0064     Adn = mip.A(i(k),:) .* mip.lb';
0065 
0066     s_ub(k) = mip.b(i(k)) - sum(min(Aup,Adn));
0067     s_lb(k) = mip.b(i(k)) - sum(max(Aup,Adn));
0068 end
0069 mip = add_column(mip,[],'c',s_lb,s_ub,[],IA);
0070 
0071 mip = add_row(mip,np+2*nb);
0072 roff = m;
0073 for k = 1 : N
0074     % I + s > 0
0075     roff = roff + 1;
0076     mip.A(roff,[mip.ind(i(k)) n+k]) = [1 1];
0077     mip.b(roff) = IND_EPS;
0078     mip.ctypes(roff) = '>';
0079 end
0080 
0081 for k = 1 : nb
0082     % s <= ub(s)*(1 - I)
0083     roff = roff + 1;
0084     mip.A(roff,[mip.ind(idx_b(k)) n+k]) = [s_ub(k) 1];
0085     mip.b(roff) = s_ub(k);
0086     mip.ctypes(roff) = '<';
0087 end
```

---

Generated on Thu 11-Aug-2011 15:06:22 by **m2html** © 2005
