# Supplementary material for: TIGER: Toolbox for integrating genome-scale metabolic models, expression data, and transcriptional regulatory networks
Source: BMC Syst Biol. 2011 Sep 23;5:147. doi: 10.1186/1752-0509-5-147 (PMC3224351; doi:10.1186/1752-0509-5-147)
Supplement: Additional file 2 — TIGER source code. Source code, documentation, and tutorials are also available online at http://bme.virginia.edu/csbl/downloads/ or http://csbl.bitbucket.org/tiger. [file 1752-0509-5-147-S2.GZ › tiger/doc/m2html/tiger/@cmpi/get_cplex_flag.html]

Description of get\_cplex\_flag


Home > tiger > @cmpi > get\_cplex\_flag.m

# get\_cplex\_flag

## PURPOSE

**Convert a CPLEX flag to a CMPI flag**

## SYNOPSIS

**function [flag] = get\_cplex\_flag(flag)**

## DESCRIPTION

```
 GET_CPLEX_FLAG  Convert a CPLEX flag to a CMPI flag
```

## CROSS-REFERENCE INFORMATION

This function calls:


This function is called by:

- cmpi

## SOURCE CODE

```
0001 function [flag] = get_cplex_flag(flag)
0002 % GET_CPLEX_FLAG  Convert a CPLEX flag to a CMPI flag
0003 
0004 switch flag
0005   case {1,101,102,121}
0006      % (integer) optimal
0007      flag = 2;
0008   case {2,3,103}
0009      % infeasible
0010      flag = 3;
0011   case {4}
0012      % infeasible or unbounded
0013      flag = 4;
0014   case {10}
0015      % iteration limit
0016      flag = 7;
0017   case {11,107,108}
0018      % time limit
0019      flag = 9;
0020   case {104}
0021      % integer solution limit
0022      flag = 10;
0023   case {105,106}
0024      % node limit reached
0025      flag = 8;
0026   otherwise
0027      flag = 0;
0028 end
```

---

Generated on Thu 11-Aug-2011 15:06:22 by **m2html** © 2005
