# Supplementary material for: TIGER: Toolbox for integrating genome-scale metabolic models, expression data, and transcriptional regulatory networks
Source: BMC Syst Biol. 2011 Sep 23;5:147. doi: 10.1186/1752-0509-5-147 (PMC3224351; doi:10.1186/1752-0509-5-147)
Supplement: Additional file 2 — TIGER source code. Source code, documentation, and tutorials are also available online at http://bme.virginia.edu/csbl/downloads/ or http://csbl.bitbucket.org/tiger. [file 1752-0509-5-147-S2.GZ › tiger/doc/m2html/tiger/@cmpi/convert_miqp.html]

Description of convert\_miqp


Home > tiger > @cmpi > convert\_miqp.m

# convert\_miqp

## PURPOSE

**Prepare a MIQP for solution**

## SYNOPSIS

**function [mip] = convert\_miqp(mip)**

## DESCRIPTION

```
 CONVERT_MIQP  Prepare a MIQP for solution

   [MIP] = CONVERT_MIQP(MIP)

   Converts a general MIQP problem to standard form:
       min sense*( x'*Q*x + obj*x )
       subject to
           A*x (<=/=/>=) b
           lb <= x <= ub

   General MIQP problems are defined by three fields:
       Q     Matrix with standard quadratic weights for x'*Q*x.

       Qd    If Qd(i,j) = k, then the objective entry is 
             k*(x(i) - x(j))^2.  A difference variable d is added such
             that d = (x(i) - x(j)), and the entry Q(d,d) = k.

       Qc.w  If Qc.w(i) and Qc.c(i) are nonzero (Qc.w and Qc.c are 
       Qc.c  vectors the same size as x), then the objective entry is
             Qc.w(i)*(x(i) - Qc.c(i))^2, i.e., the weighted least-square
             distance between x(i) and a constant c(i).
```

## CROSS-REFERENCE INFORMATION

This function calls:

- add\_column Add a column to a TIGER model structure
- add\_row Add a row to a TIGER model structure
- count Count the number of nonzero elements in a vector

This function is called by:

- cmpi

## SOURCE CODE

```
0001 function [mip] = convert_miqp(mip)
0002 % CONVERT_MIQP  Prepare a MIQP for solution
0003 %
0004 %   [MIP] = CONVERT_MIQP(MIP)
0005 %
0006 %   Converts a general MIQP problem to standard form:
0007 %       min sense*( x'*Q*x + obj*x )
0008 %       subject to
0009 %           A*x (<=/=/>=) b
0010 %           lb <= x <= ub
0011 %
0012 %   General MIQP problems are defined by three fields:
0013 %       Q     Matrix with standard quadratic weights for x'*Q*x.
0014 %
0015 %       Qd    If Qd(i,j) = k, then the objective entry is
0016 %             k*(x(i) - x(j))^2.  A difference variable d is added such
0017 %             that d = (x(i) - x(j)), and the entry Q(d,d) = k.
0018 %
0019 %       Qc.w  If Qc.w(i) and Qc.c(i) are nonzero (Qc.w and Qc.c are
0020 %       Qc.c  vectors the same size as x), then the objective entry is
0021 %             Qc.w(i)*(x(i) - Qc.c(i))^2, i.e., the weighted least-square
0022 %             distance between x(i) and a constant c(i).
0023 
0024 has_Q  = ~isempty(cmpi.check_field('Q',mip));
0025 has_Qd = ~isempty(cmpi.check_field('Qd',mip));
0026 has_Qc = ~isempty(cmpi.check_field('Qc',mip));
0027 
0028 assert(sum([has_Q has_Qd has_Qc]) <= 1, ...
0029        'only one of Q, Qd, and Qc can be specified');
0030 
0031 [m,n] = size(mip.A);
0032  
0033 if has_Q
0034     % move all nonzero elements in Q to the lower half
0035     mip.Q  = tril(mip.Q) + triu(mip.Q)'.*double(tril(mip.Q) == 0);
0036 elseif has_Qd
0037     % move all nonzero elements in Q to the lower half
0038     Qd = mip.Qd;
0039     Qd = tril(Qd) + triu(Qd)'.*double(tril(Qd) == 0);
0040     
0041     mip.Q = spalloc(n,n,nnz(Qd));
0042     [I,J,w] = find(Qd);
0043     Nadd = length(w);
0044     mip = add_column(mip,Nadd,'c');
0045     mip = add_row(mip,Nadd);
0046     for i = 1 : Nadd
0047         mip.A(m+i,[I(i) J(i) n+i]) = [1 -1 1];
0048         mip.Q(n+i,n+i) = w(i);
0049         lb = min(mip.lb([I(i) J(i)]));
0050         ub = max(mip.ub([I(i) J(i)]));
0051         mip.lb(n+i) = -(ub - lb);
0052         mip.ub(n+i) = ub - lb;
0053     end
0054 elseif has_Qc
0055     mip.Q = spalloc(n,n,nnz(mip.Qc.w));
0056     Nadd = count(mip.Qc.w);
0057     mip = add_column(mip,Nadd,'c');
0058     mip = add_row(mip,Nadd);
0059     rowidx = m;
0060     colidx = 0;
0061     for i = 1 : length(mip.Qc.w)
0062         if mip.Qc.w(i) ~= 0
0063             rowidx = rowidx + 1;
0064             colidx = colidx + 1;
0065             mip.A(rowidx,[n+colidx i]) = [1 -1];
0066             mip.b(rowidx) = -mip.Qc.c(i);
0067             mip.Q(n+colidx,n+colidx) = mip.Qc.w(i);
0068             bound = mip.ub(i) - mip.lb(i);
0069             mip.ub(n+colidx) =  bound;
0070             mip.lb(n+colidx) = -bound;
0071         end
0072     end
0073 end
0074 
0075 % make Q symmetric
0076 mip.Q = tril(mip.Q) + tril(mip.Q,-1)';
0077 
0078
```

---

Generated on Thu 11-Aug-2011 15:06:22 by **m2html** © 2005
