# Supplementary material for: TIGER: Toolbox for integrating genome-scale metabolic models, expression data, and transcriptional regulatory networks
Source: BMC Syst Biol. 2011 Sep 23;5:147. doi: 10.1186/1752-0509-5-147 (PMC3224351; doi:10.1186/1752-0509-5-147)
Supplement: Additional file 2 — TIGER source code. Source code, documentation, and tutorials are also available online at http://bme.virginia.edu/csbl/downloads/ or http://csbl.bitbucket.org/tiger. [file 1752-0509-5-147-S2.GZ › tiger/doc/m2html/tiger/@cmpi/solve_multiple_mips.html]

Description of solve\_multiple\_mips


Home > tiger > @cmpi > solve\_multiple\_mips.m

# solve\_multiple\_mips

## PURPOSE

## SYNOPSIS

**function [sols] = solve\_multiple\_mips(mip,alts,varargin)**

## DESCRIPTION

## CROSS-REFERENCE INFORMATION

This function calls:


This function is called by:

- cmpi

## SUBFUNCTIONS

- function record\_sol(s,j)

## SOURCE CODE

```
0001 function [sols] = solve_multiple_mips(mip,alts,varargin)
0002 
0003 p = inputParser;
0004 p.addParamValue('restart',true);
0005 p.parse(varargin{:});
0006 
0007 if isempty(alts)
0008     sols = cmpi.solve_mip(mip);
0009     return;
0010 end
0011 
0012 fields = fieldnames(alts);
0013 Ncols = cellfun(@(x) size(alts.(x),2),fields);
0014 Niter = Ncols(1);
0015 assert(all(Ncols == Niter), ...
0016        'dimensions of each field in ALT are not consistent');
0017 
0018 % start the solution
0019 solver = cmpi.get_solver();
0020 
0021 if ~issparse(mip.A)
0022     mip.A = sparse(mip.A);
0023 end
0024 
0025 if ~isfield(mip,'sense') || isempty(mip.sense)
0026     mip.sense = 1;
0027 end
0028 
0029 if ~isfield(mip,'options') || isempty(mip.options)
0030     mip.options = cmpi.get_options();
0031 end
0032 
0033 % preserve size before conversion
0034 N = size(mip.A,2);
0035 
0036 mip = cmpi.convert_indicators(mip);
0037 
0038 sols.x = zeros(N,Niter);
0039 sols.val = zeros(1,Niter);
0040 sols.output = cell(1,Niter);
0041 sols.flag = zeros(1,Niter);
0042 sols.time = zeros(1,Niter);
0043 
0044 if ~p.Results.restart
0045     solver = 'CMPI__NO_RESTART';
0046 end
0047 
0048 switch solver
0049     case 'cplex'
0050         % start acceleration
0051         cplex = Cplex();
0052         cplex.DisplayFunc = [];
0053         if mip.sense == 1
0054             cplex.Model.sense = 'minimize';
0055         else
0056             cplex.Model.sense = 'maximize';
0057         end
0058         cplex.Model.obj = mip.obj;
0059         mip.A(mip.ctypes == '>',:) = -mip.A(mip.ctypes == '>',:);
0060         mip.b(mip.ctypes == '>') = -mip.b(mip.ctypes == '>');
0061         cplex.Model.A = mip.A;
0062         cplex.Model.ctype = upper(mip.vartypes);
0063         cplex.Model.lb = mip.lb(:);
0064         cplex.Model.ub = mip.ub(:);
0065         cplex.Model.rhs = mip.b(:);
0066         lhs = -inf*ones(size(mip.b(:)));
0067         lhs(mip.ctypes == '=') = mip.b(mip.ctypes == '=');
0068         cplex.Model.lhs = lhs;
0069         
0070         [~,cplex] = cmpi.set_cplex_opts(mip.options,cplex);
0071         if isfield(mip.options,'Display')
0072             if strcmpi(mip.options.Display,'off')
0073                 cplex.DisplayFunc = [];
0074             end
0075         end
0076         
0077         for i = 1 : Niter
0078             for f = 1 : length(fields)
0079                 cplex.Model.(fields{f}) = alts.(fields{f})(:,i);
0080             end
0081             start_time = tic;
0082             cplex.solve();
0083             total_time = toc(start_time);
0084             sol.x = cplex.Solution.x;
0085             sol.flag = cmpi.get_cplex_flag(cplex.Solution.status);
0086             sol.val = cplex.Solution.objval;
0087             sol.output = cplex.Solution.statusstring;
0088             record_sol(sol,i);
0089             if length(cplex.MipStart) > 1
0090                 cplex.MipStart = cplex.MipStart(end);
0091             end
0092         end
0093         
0094     otherwise
0095         % call solve_mip on each case
0096         for i = 1 : Niter
0097             for f = 1 : length(fields)
0098                 mip.(fields{f}) = alts.(fields{f})(:,i);
0099             end
0100             start_time = tic;
0101             sol = cmpi.solve_mip(mip);
0102             total_time = toc(start_time);
0103             record_sol(sol,i);
0104         end
0105 end
0106 
0107 function record_sol(s,j)
0108     if ~isempty(s.x)
0109         sols.x(:,j) = s.x(1:N);
0110     end
0111     sols.val(j) = s.val;
0112     sols.output{j} = s.output;
0113     sols.flag(j) = s.flag;
0114     sols.time(j) = total_time;
0115 end
0116 
0117 end
```

---

Generated on Thu 11-Aug-2011 15:06:22 by **m2html** © 2005
