# Supplementary material for: TIGER: Toolbox for integrating genome-scale metabolic models, expression data, and transcriptional regulatory networks
Source: BMC Syst Biol. 2011 Sep 23;5:147. doi: 10.1186/1752-0509-5-147 (PMC3224351; doi:10.1186/1752-0509-5-147)
Supplement: Additional file 2 — TIGER source code. Source code, documentation, and tutorials are also available online at http://bme.virginia.edu/csbl/downloads/ or http://csbl.bitbucket.org/tiger. [file 1752-0509-5-147-S2.GZ › tiger/doc/m2html/tiger/@cmpi/show_mip.html]

Description of show\_mip


Home > tiger > @cmpi > show\_mip.m

# show\_mip

## PURPOSE

**Show equations for a MIP structure**

## SYNOPSIS

**function show\_mip(mip,varargin)**

## DESCRIPTION

```
 SHOW_MIP  Show equations for a MIP structure

   SHOW_MIP(MIP,...params...)

   Print algebraic equations for a MIP structure.

   Inputs
   MIP      CMPI model structure

   Parameters
   'rowidxs'  Indexes of rows (constraints) to show. (default = all rows)
   'varidxs'  Indexes of variables to show. (default = all rows)
   'rownames' Cell array of names for each row.  Empty values are 
              replaced with 'ROW_x'.  May be shorter than the number of 
              rows; extra 'ROW_x' names are added automatically.
   'varnames' Cell array of name for each column.  Format is the same as
              for 'rownames'.
   'showvars' If true, show bounds on each variable.  (default = true)
```

## CROSS-REFERENCE INFORMATION

This function calls:

- printbuffer

This function is called by:

- cmpi

## SUBFUNCTIONS

- function [zipped] = zip\_names(default,given)
- function show\_coef\_list(constraint)
- function [str] = make\_coef\_name\_pair(coef,name,first)

## SOURCE CODE

```
0001 function show_mip(mip,varargin)
0002 % SHOW_MIP  Show equations for a MIP structure
0003 %
0004 %   SHOW_MIP(MIP,...params...)
0005 %
0006 %   Print algebraic equations for a MIP structure.
0007 %
0008 %   Inputs
0009 %   MIP      CMPI model structure
0010 %
0011 %   Parameters
0012 %   'rowidxs'  Indexes of rows (constraints) to show. (default = all rows)
0013 %   'varidxs'  Indexes of variables to show. (default = all rows)
0014 %   'rownames' Cell array of names for each row.  Empty values are
0015 %              replaced with 'ROW_x'.  May be shorter than the number of
0016 %              rows; extra 'ROW_x' names are added automatically.
0017 %   'varnames' Cell array of name for each column.  Format is the same as
0018 %              for 'rownames'.
0019 %   'showvars' If true, show bounds on each variable.  (default = true)
0020 
0021 [nrows,nvars] = size(mip.A);
0022 
0023 default_varnames = arrayfun(@(x) ['x(' num2str(x) ')'], 1:nvars, ...
0024                             'Uniform', false);
0025 default_rownames = arrayfun(@(x) ['ROW_' num2str(x)], 1:nrows, ...
0026                             'Uniform', false);
0027 
0028 p = inputParser;
0029 
0030 p.addParamValue('rowidxs',1 : nrows);
0031 p.addParamValue('varidxs',1 : nvars);
0032 p.addParamValue('rownames',default_rownames);
0033 p.addParamValue('varnames',default_varnames);
0034 p.addParamValue('showvars',true);
0035 
0036 p.parse(varargin{:});
0037 
0038 showvars = p.Results.showvars;
0039 
0040 % default ranges and names
0041 if isfield(mip,'varnames')
0042     varnames = mip.varnames;
0043 else
0044     varnames = p.Results.varnames;
0045 end
0046 if isfield(mip,'rownames')
0047     rownames = mip.rownames;
0048 else
0049     rownames = p.Results.rownames;
0050 end
0051 
0052 varidxs = p.Results.varidxs;
0053 if isa(varidxs,'logical')
0054     varidxs = find(varidxs);
0055 end
0056 
0057 rowidxs = p.Results.rowidxs;
0058 if isa(rowidxs,'logical')
0059     rowidxs = find(rowidxs);
0060 end                     
0061 
0062 % expand the names if an incomplete list was given
0063 varnames = zip_names(default_varnames,varnames);
0064 rownames = zip_names(default_rownames,rownames);
0065 
0066 % show quadratic
0067 qptype = cmpi.miqp_type(mip);
0068 if ~isempty(qptype)
0069     fprintf('\n\n----- Quadratic Objective -----\n');
0070     pb = printbuffer();
0071     pb.start_wrap;
0072     switch qptype
0073         case 'Q'
0074             fprintf('Q:  ');
0075             [I,J] = find(mip.Q);
0076             for i = 1 : length(I)
0077                 if I(i) == J(i)
0078                     rxn_name = [mip.varnames{I(i)} '^2'];
0079                 else
0080                     rxn_name = [mip.varanems{I(i)} '*' ...
0081                                 mip.varnames{J(i)}];
0082                 end
0083                 pb.printf(make_coef_name_pair(mip.Q(I(i),J(i)), ...
0084                                               rxn_name, ...
0085                                               i == 1));
0086             end
0087         case 'Qd'
0088             fprintf('Qd:  ');
0089             [I,J] = find(mip.Qd);
0090             for i = 1 : length(I)
0091                 rxn_name = sprintf('(%s - %s)^2',mip.varnames{I(i)}, ...
0092                                                  mip.varnames{J(i)});
0093                 pb.printf(make_coef_name_pair(mip.Qd(I(i),J(i)), ...
0094                                               rxn_name, ...
0095                                               i == 1));
0096             end
0097         case 'Qc'
0098             I = find(mip.Qc.w);
0099             for i = 1 : length(I)
0100                 rxn_name = sprintf('(%s - %s)^2',mip.varnames{I(i)}, ...
0101                                                  num2str(mip.Qc.c(I(i))));
0102                 pb.printf(make_coef_name_pair(mip.Qc.w(I(i)), ...
0103                                               rxn_name, ...
0104                                               i == 1));
0105             end
0106             
0107     end
0108     pb.stop_wrap;
0109 end
0110     
0111 % show objective
0112 fprintf('\n\n----- Objective -----\n');
0113 pb = printbuffer;
0114 pb.start_wrap;
0115 pb.printf('obj:  ');
0116 show_coef_list(mip.obj);
0117 pb.stop_wrap;
0118 
0119 % show constraints
0120 fprintf('\n\n----- Constraints -----\n');
0121 for r = 1 : length(rowidxs)
0122     row = rowidxs(r);
0123     pb = printbuffer;
0124     pb.start_wrap;
0125     pb.printf('%s:  ',rownames{row});
0126     show_coef_list(mip.A(row,:));
0127     switch mip.ctypes(r)
0128         case '<'
0129             fprintf(' <= ');
0130         case '>'
0131             fprintf(' >= ');
0132         case '='
0133             fprintf(' = ');
0134     end
0135     fprintf('%g\n',mip.b(r));
0136     pb.stop_wrap;
0137 end
0138 
0139 % show indicators
0140 if isfield(mip,'ind') && any(mip.ind)
0141     fprintf('\n\n----- Indicators -----\n');
0142     rows = find(mip.ind);
0143     inds = mip.ind(rows);
0144     types = mip.indtypes(rows);
0145     for i = 1 : length(inds)
0146         if types(i) == 'p'
0147             fprintf('   %s  => %s\n',rownames{rows(i)},varnames{inds(i)});
0148         else
0149             fprintf('   %s <=> %s\n',rownames{rows(i)},varnames{inds(i)});
0150         end
0151     end
0152 end
0153 
0154 if showvars
0155     % show variable bounds
0156     maxlength = max(cellfun(@(x) length(x), varnames));
0157     fmt = ['%' num2str(maxlength + 2) 's'];
0158     fprintf('\n\n----- Variable bounds -----\n');
0159     for i = 1 : length(varidxs)
0160         fprintf(fmt,[varnames{varidxs(i)} ':']);
0161         fprintf('  %s',mip.vartypes(varidxs(i)));
0162         fprintf('  [%g,%g]\n', [mip.lb(varidxs(i)) mip.ub(varidxs(i))]);
0163     end
0164 end
0165 
0166 
0167 function [zipped] = zip_names(default,given)
0168     zipped = default;
0169     for j = 1 : length(given)
0170         if ~isempty(given{j})
0171             zipped{j} = given{j};
0172         end
0173     end
0174 end
0175     
0176 function show_coef_list(constraint)
0177     nonzeros = find(constraint(varidxs));
0178     for j = 1 : length(nonzeros)
0179         col = nonzeros(j);
0180         coef = constraint(col);
0181         pb.printf(make_coef_name_pair(coef,varnames{col},j==1));
0182     end
0183 end
0184 
0185 function [str] = make_coef_name_pair(coef,name,first)
0186     if coef < 0 && first
0187         sign = '-';
0188     elseif coef < 0 && ~first
0189         sign = ' - ';
0190     elseif first
0191         sign = '';
0192     else
0193         sign = ' + ';
0194     end
0195     
0196     if abs(coef) == 1
0197         numstr = '';
0198     else
0199         numstr = [num2str(abs(coef)) '*'];
0200     end
0201     
0202     str = sprintf('%s%s%s',sign,numstr,name);
0203 end      
0204 
0205 end
0206
```

---

Generated on Thu 11-Aug-2011 15:06:22 by **m2html** © 2005
