# Supplementary material for: TIGER: Toolbox for integrating genome-scale metabolic models, expression data, and transcriptional regulatory networks
Source: BMC Syst Biol. 2011 Sep 23;5:147. doi: 10.1186/1752-0509-5-147 (PMC3224351; doi:10.1186/1752-0509-5-147)
Supplement: Additional file 2 — TIGER source code. Source code, documentation, and tutorials are also available online at http://bme.virginia.edu/csbl/downloads/ or http://csbl.bitbucket.org/tiger. [file 1752-0509-5-147-S2.GZ › tiger/doc/m2html/tiger/@cmpi/add_obj_constraint.html]

Description of add\_obj\_constraint


Home > tiger > @cmpi > add\_obj\_constraint.m

# add\_obj\_constraint

## PURPOSE

**Add minimal objective constraint to a MILP**

## SYNOPSIS

**function [milp] = add\_obj\_constraint(milp,frac)**

## DESCRIPTION

```
 ADD_OBJ_CONSTRAINT  Add minimal objective constraint to a MILP

   [MILP] = ADD_OBJ_CONSTRAINT(MILP,FRAC)

   Adds a constraint the c'x >= FRAC*fmax, where
       fmax = max c'x s.t. Ax <= b

   Inputs
   MILP    CMPI MILP problem structure
   FRAC    Fraction of maximum objective value that must be
           reached (optional, default = 1.0)

   Outputs
   MILP    CMPI MILP problem structure with objective constraint
```

## CROSS-REFERENCE INFORMATION

This function calls:


This function is called by:

- cmpi

## SOURCE CODE

```
0001 function [milp] = add_obj_constraint(milp,frac)
0002 % ADD_OBJ_CONSTRAINT  Add minimal objective constraint to a MILP
0003 %
0004 %   [MILP] = ADD_OBJ_CONSTRAINT(MILP,FRAC)
0005 %
0006 %   Adds a constraint the c'x >= FRAC*fmax, where
0007 %       fmax = max c'x s.t. Ax <= b
0008 %
0009 %   Inputs
0010 %   MILP    CMPI MILP problem structure
0011 %   FRAC    Fraction of maximum objective value that must be
0012 %           reached (optional, default = 1.0)
0013 %
0014 %   Outputs
0015 %   MILP    CMPI MILP problem structure with objective constraint
0016 
0017 if nargin < 2,  frac = 1.0; end
0018 
0019 sol = cmpi.solve_milp(milp);
0020 
0021 milp.A = [milp.A; -milp.obj(:)'];
0022 milp.b(end+1) = -frac*sol.val;
0023 milp.ctypes(end+1) = '<';
0024
```

---

Generated on Thu 11-Aug-2011 15:06:22 by **m2html** © 2005
