# Supplementary material for: TIGER: Toolbox for integrating genome-scale metabolic models, expression data, and transcriptional regulatory networks
Source: BMC Syst Biol. 2011 Sep 23;5:147. doi: 10.1186/1752-0509-5-147 (PMC3224351; doi:10.1186/1752-0509-5-147)
Supplement: Additional file 2 — TIGER source code. Source code, documentation, and tutorials are also available online at http://bme.virginia.edu/csbl/downloads/ or http://csbl.bitbucket.org/tiger. [file 1752-0509-5-147-S2.GZ › tiger/doc/m2html/tiger/@cmpi/miqp_type.html]

Description of miqp\_type


Home > tiger > @cmpi > miqp\_type.m

# miqp\_type

## PURPOSE

**Return the quadratic type of a MIQP problem**

## SYNOPSIS

**function [qtype] = miqp\_type(mip)**

## DESCRIPTION

```
 MIQP_TYPE  Return the quadratic type of a MIQP problem

   [QTYPE] = MIQP_TYPE(MIP)

   Return value:
       'Q'   Standard form quadratic
       'Qd'  Quadratic difference (x_i - x_j)^2
       'Qc'  Quadratic constant (x_i - c)^2
       []    Not quadratic
```

## CROSS-REFERENCE INFORMATION

This function calls:


This function is called by:

- cmpi

## SOURCE CODE

```
0001 function [qtype] = miqp_type(mip)
0002 % MIQP_TYPE  Return the quadratic type of a MIQP problem
0003 %
0004 %   [QTYPE] = MIQP_TYPE(MIP)
0005 %
0006 %   Return value:
0007 %       'Q'   Standard form quadratic
0008 %       'Qd'  Quadratic difference (x_i - x_j)^2
0009 %       'Qc'  Quadratic constant (x_i - c)^2
0010 %       []    Not quadratic
0011 
0012 if isfield(mip,'Q') && ~isempty(mip.Q) && ~all(mip.Q(:) == 0)
0013     qtype = 'Q';
0014 elseif isfield(mip,'Qd') && ~isempty(mip.Qd)
0015     qtype = 'Qd';
0016 elseif isfield(mip,'Qc') && ~isempty(mip.Qc)
0017     qtype = 'Qc';
0018 else
0019     qtype = [];
0020 end
```

---

Generated on Thu 11-Aug-2011 15:06:22 by **m2html** © 2005
