# Supplementary material for: TIGER: Toolbox for integrating genome-scale metabolic models, expression data, and transcriptional regulatory networks
Source: BMC Syst Biol. 2011 Sep 23;5:147. doi: 10.1186/1752-0509-5-147 (PMC3224351; doi:10.1186/1752-0509-5-147)
Supplement: Additional file 2 — TIGER source code. Source code, documentation, and tutorials are also available online at http://bme.virginia.edu/csbl/downloads/ or http://csbl.bitbucket.org/tiger. [file 1752-0509-5-147-S2.GZ › tiger/doc/m2html/tiger/@cmpi/cmpi.html]

Description of cmpi


Home > tiger > @cmpi > cmpi.m

# cmpi

## PURPOSE

## SYNOPSIS

**This is a script file.**

## DESCRIPTION

## CROSS-REFERENCE INFORMATION

This function calls:

- add\_obj\_constraint Add minimal objective constraint to a MILP
- check\_field Check if a field exists in a structure.
- check\_mip Ensure that the sense, ind, and indtypes fields are filled
- cmpi
- convert\_indicators Convert indicators to MILP constraints
- convert\_miqp Prepare a MIQP for solution
- get\_cplex\_flag Convert a CPLEX flag to a CMPI flag
- miqp\_type Return the quadratic type of a MIQP problem
- set\_cplex\_opts Convert a CMPI options structure into CPLEX opts
- set\_gurobi\_opts Convert a CMPI options structure into GUROBI opts
- show\_mip Show equations for a MIP structure
- solve\_mip Solve a Mixed Integer Programming problem
- solve\_multiple\_mips
- tile\_mip Combine several MIPs into a single structure
- verify\_sol Verify a solution is feasible

This function is called by:

- cmpi

## SUBFUNCTIONS

- function [ctype] = make\_ctype(leq,geq,eq)
- function [vartype] = make\_vartype(c,b,i,s,n)
- function [sense] = make\_sense(s)
- function [tf] = is\_acceptable\_exit(sol)
- function set\_option(option,val)
- function clear\_options()
- function [opts] = get\_options()
- function set\_solver(solver)
- function [solver] = get\_solver()
- function [ind\_eps] = get\_ind\_eps()
- function init()
- function assert\_init()

## SOURCE CODE

```
0001 classdef cmpi
0002 % CMPI  Common Mathematical Programming Interface
0003 %
0004 %   CMPI defines a common interface for mathematical programming solvers.
0005 %   Its methods help construct and solve LP/QP/MILP/MIQP problems.
0006 %
0007 %   CMPI represents a mathematical program as a structure with various
0008 %   fields.  A description of these fields can be found in the
0009 %   documentation for the SOLVE_MIP function (help solve_mip).
0010     
0011 
0012 properties (Constant)
0013     % Initial (default) values
0014     init_SOLVER = 'cplex';
0015     init_IND_EPS = 1e-8;
0016 end
0017 
0018 methods (Static)
0019     function [ctype] = make_ctype(leq,geq,eq)
0020         % MAKE_CTYPE  Make contraint type identifiers
0021         %
0022         %   [CTYPE] = MAKE_CTYPE(LEQ,GEQ,EQ)
0023         %
0024         %   Returns a char array defining the constraint types for
0025         %   LEQ '<=' inequalities, GEQ '<=' inequalities, and
0026         %   EQ equalities.
0027         
0028         if nargin < 3,   eq = 0; end
0029         if nargin < 2,  geq = 0; end
0030 
0031         ctype = [repmat('<', 1, leq) ...
0032                  repmat('>', 1, geq) ...
0033                  repmat('=', 1, eq)];
0034     end
0035 
0036     function [vartype] = make_vartype(c,b,i,s,n)
0037         % MAKE_VARTYPE  Make variable type identifiers
0038         %
0039         %   [VARTYPE] = MAKE_VARTYPE(C,B,I,S,N)
0040         %
0041         %   Returns a char array defining the variable types for
0042         %   C continuous, B binary, I integer, S semi-continuous,
0043         %   and N semi-integer-continuous variables.
0044         
0045         if nargin < 5,  n = 0; end
0046         if nargin < 4,  s = 0; end
0047         if nargin < 3,  i = 0; end
0048         if nargin < 2,  b = 0; end
0049 
0050         vartype = [repmat('C', 1, c) ...
0051                    repmat('B', 1, b) ...
0052                    repmat('I', 1, i) ...
0053                    repmat('S', 1, s) ...
0054                    repmat('N', 1, n)];
0055     end
0056 
0057     function [sense] = make_sense(s)
0058         % MAKE_SENSE  Convert optimization sense to an integer
0059         %
0060         %   Converts the strings 'min', 'minimize', 'max', and 'maximize'
0061         %   into the corresponding optimization directions (1 and -1).
0062         
0063         switch lower(s)
0064             case {'min','minimize'}
0065                 sense =  1;
0066             case {'max','maximize'}
0067                 sense = -1;
0068         end
0069     end
0070 
0071     function [tf] = is_acceptable_exit(sol)
0072         % IS_ACCEPTABLE_EXIT  Determine if a solution was found
0073         %
0074         %   Determines if a CMPI solution structure contains an error
0075         %   code that returns a valid, feasible solution.  Acceptable
0076         %   exits are:
0077         %       Optimal
0078         %       Iteration limit reached
0079         %       Node limit reached
0080         %       Time limit reached
0081         %       Solution limit reached
0082         
0083         tf = ( (sol.flag == 2) || (sol.flag == 7) ...
0084               || (sol.flag == 8) || (sol.flag == 9) ...
0085               || (sol.flag == 10) ) && ~isempty(sol.x);
0086     end
0087 
0088     function set_option(option,val)
0089         % SET_OPTIONS  Set a default solver option
0090         %
0091         %   Sets an option in the default option structure.  For a
0092         %   description of the solver options, see the documentation
0093         %   for SOLVE_MILP.
0094         
0095         global CMPI_OPTIONS
0096         CMPI_OPTIONS.(option) = val;
0097     end
0098 
0099     function clear_options()
0100         % CLEAR_OPTIONS  Clear the default solver options
0101         global CMPI_OPTIONS
0102         CMPI_OPTIONS = [];
0103     end
0104 
0105     function [opts] = get_options()
0106         % GET_OPTIONS  Return the default solver options
0107         cmpi.assert_init();
0108         global CMPI_OPTIONS
0109         opts = CMPI_OPTIONS;
0110     end
0111 
0112     function set_solver(solver)
0113         % SET_SOLVER  Set the default solver name
0114         global CMPI_SOLVER
0115         CMPI_SOLVER = solver;
0116     end
0117 
0118     function [solver] = get_solver()
0119         % GET_SOLVER  Get the default solver name
0120         cmpi.assert_init();
0121         global CMPI_SOLVER
0122         solver = CMPI_SOLVER;
0123     end
0124 
0125     function [ind_eps] = get_ind_eps()
0126         % GET_IND_EPS  Get epsilon for indicator constraints
0127         cmpi.assert_init();
0128         global CMPI_IND_EPS
0129         ind_eps = CMPI_IND_EPS;
0130     end
0131     
0132     function init()
0133         % INIT  Initialze CMPI
0134         %
0135         %   Creates the global variables and default settings for the
0136         %   solver name and options.
0137         
0138         global CMPI_OPTIONS
0139         global CMPI_SOLVER
0140         global CMPI_IND_EPS
0141         global CMPI_INITIALIZED
0142 
0143         CMPI_SOLVER = cmpi.init_SOLVER;
0144         CMPI_IND_EPS = cmpi.init_IND_EPS;
0145         
0146         CMPI_INITIALIZED = true;
0147     end
0148 
0149     function assert_init()
0150         % ASSERT_INIT  Assert that CMPI has been initialized
0151         global CMPI_INITIALIZED
0152         
0153         if ~CMPI_INITIALIZED
0154             cmpi.init();
0155         end
0156     end
0157     
0158     % external static methods
0159     [sol] = solve_mip(mip)
0160     [val] = check_field(field,structure)
0161     [tf] = verify_sol(milp,sol,tol)
0162     [milp] = add_obj_constraint(milp,frac)
0163     [tf,violators] = check_bounded_rxns(model,sol,tol)
0164     show_mip(mip,rowidxs,colidxs,rownames,colnames,showvars)
0165     [opts,cplex] = set_cplex_opts(options,cplex)
0166     [opts] = set_gurobi_opts(options)
0167     [flag] = get_cplex_flag(status)
0168     [mip] = convert_indicators(mip)
0169     [mip] = convert_miqp(mip)
0170     [milp] = tile_milp(varargin)
0171     [mip] = tile_mip(varargin)
0172     [mip] = check_mip(mip)
0173     [sols] = solve_multiple_mips(mip,alts,varargin)
0174     [qtype] = miqp_type(mip)
0175 end
0176 
0177 end % class
0178         
0179         
0180
```

---

Generated on Thu 11-Aug-2011 15:06:22 by **m2html** © 2005
