# Supplementary material for: TIGER: Toolbox for integrating genome-scale metabolic models, expression data, and transcriptional regulatory networks
Source: BMC Syst Biol. 2011 Sep 23;5:147. doi: 10.1186/1752-0509-5-147 (PMC3224351; doi:10.1186/1752-0509-5-147)
Supplement: Additional file 2 — TIGER source code. Source code, documentation, and tutorials are also available online at http://bme.virginia.edu/csbl/downloads/ or http://csbl.bitbucket.org/tiger. [file 1752-0509-5-147-S2.GZ › tiger/doc/m2html/tiger/@cmpi/check_field.html]

Description of check\_field


Home > tiger > @cmpi > check\_field.m

# check\_field

## PURPOSE

**Check if a field exists in a structure.**

## SYNOPSIS

**function [val] = check\_field(field,structure)**

## DESCRIPTION

```
 CHECK_FIELD  Check if a field exists in a structure.

   [VAL] = CHECK_FIELD(FIELD,STRUCTURE)
   
   Checks if FIELD is a defined field in STRUCTURE.  If defined, 
   returns the value in STRUCTURE.FIELD.  If not definied, returns
   the empty array.
```

## CROSS-REFERENCE INFORMATION

This function calls:


This function is called by:

- cmpi

## SOURCE CODE

```
0001 function [val] = check_field(field,structure)
0002 % CHECK_FIELD  Check if a field exists in a structure.
0003 %
0004 %   [VAL] = CHECK_FIELD(FIELD,STRUCTURE)
0005 %
0006 %   Checks if FIELD is a defined field in STRUCTURE.  If defined,
0007 %   returns the value in STRUCTURE.FIELD.  If not definied, returns
0008 %   the empty array.
0009 
0010 if isfield(structure,field)
0011     val = structure.(field);
0012 else
0013     val = [];
0014 end
```

---

Generated on Thu 11-Aug-2011 15:06:22 by **m2html** © 2005
