# Supplementary material for: TIGER: Toolbox for integrating genome-scale metabolic models, expression data, and transcriptional regulatory networks
Source: BMC Syst Biol. 2011 Sep 23;5:147. doi: 10.1186/1752-0509-5-147 (PMC3224351; doi:10.1186/1752-0509-5-147)
Supplement: Additional file 2 — TIGER source code. Source code, documentation, and tutorials are also available online at http://bme.virginia.edu/csbl/downloads/ or http://csbl.bitbucket.org/tiger. [file 1752-0509-5-147-S2.GZ › tiger/doc/m2html/tiger/@cmpi/solve_mip.html]

Description of solve\_mip


Home > tiger > @cmpi > solve\_mip.m

# solve\_mip

## PURPOSE

**Solve a Mixed Integer Programming problem**

## SYNOPSIS

**function [sol] = solve\_mip(mip)**

## DESCRIPTION

```
 SOLVE_MIP  Solve a Mixed Integer Programming problem

   [SOL] = SOLVE_MIP(MIP,SOLVER)

   Solves the Mixed Integer Linear Programming problem
       min sense*obj*x
       subject to
           A*x (<=/=/>=) b
           lb <= x <= ub

   or the Mixed Integer Quadratic Programming problem
       min sense*(x'*Q*x + obj*x)
       subject to
           A*x (<=/=/>=) b
           lb <= x <= ub

   Inputs
   MIP     Problem structure.  Fields include:
               obj       Objective coefficients
               sense     Direction of optimization:
                              1    Minimization
                             -1    Maximization
               A         Coefficient matrix for constraints.
               b         Right-hand side of constraints.
               ctypes    Char array denoting the type of each constraint:
                             '<'   a*x <= b
                             '='   a*x == b
                             '>'   a*x >= b
               lb        Lower bound for each variable.
               ub        Upper bound for each variable.
               vartypes  Char array denoting the type of each variable:
                             'C'   Continuous
                             'B'   Binary
                             'I'   Integer
               options   Solver options (see below).  If not given, the
                         options in the global variable CMPI_OPTIONS are
                         used (if defined).
                             MaxTime     Maximum solution time (seconds)
                             MaxIter     Maximum simplex iterations
                             MaxNodes    Maximum number of nodes
                             Display     Turn reporting to the screen
                                         'on' or 'off' (default)
                             FeasTol     Feasibility tolerance
                             IntFeasTol  Integer feasibility tolerance
                             OptTol      Optimality tolerance
                             AbsOptTol   Absolute optimality tolerance
               Q         Quadratic objective matrix.  If given, the
                         problem is solved as a MIQP.  See CONVERT_MIQP
                         for details on Q and related fields.

   Outputs
   SOL     Solution structure with fields:
               x       Optimal vector
               val     Objective values (sense*c*x)
               flag    Exit flag.  Possible values are:
                           1   Not started
                           2   Optimal
                           3   Infeasible
                           4   Infeasible or unbounded
                           5   Unbounded
                           6   Objective worse than user cutoff
                           7   Iteration limit reached
                           8   Node limit reached
                           9   Time limit reached
                           10  Solution limit reached
                           11  User interruption
                           12  Numerical difficulties
                           13  Suboptimal solution
               output  Other solver-specific output
```

## CROSS-REFERENCE INFORMATION

This function calls:


This function is called by:

- cmpi

## SOURCE CODE

```
0001 function [sol] = solve_mip(mip)
0002 % SOLVE_MIP  Solve a Mixed Integer Programming problem
0003 %
0004 %   [SOL] = SOLVE_MIP(MIP,SOLVER)
0005 %
0006 %   Solves the Mixed Integer Linear Programming problem
0007 %       min sense*obj*x
0008 %       subject to
0009 %           A*x (<=/=/>=) b
0010 %           lb <= x <= ub
0011 %
0012 %   or the Mixed Integer Quadratic Programming problem
0013 %       min sense*(x'*Q*x + obj*x)
0014 %       subject to
0015 %           A*x (<=/=/>=) b
0016 %           lb <= x <= ub
0017 %
0018 %   Inputs
0019 %   MIP     Problem structure.  Fields include:
0020 %               obj       Objective coefficients
0021 %               sense     Direction of optimization:
0022 %                              1    Minimization
0023 %                             -1    Maximization
0024 %               A         Coefficient matrix for constraints.
0025 %               b         Right-hand side of constraints.
0026 %               ctypes    Char array denoting the type of each constraint:
0027 %                             '<'   a*x <= b
0028 %                             '='   a*x == b
0029 %                             '>'   a*x >= b
0030 %               lb        Lower bound for each variable.
0031 %               ub        Upper bound for each variable.
0032 %               vartypes  Char array denoting the type of each variable:
0033 %                             'C'   Continuous
0034 %                             'B'   Binary
0035 %                             'I'   Integer
0036 %               options   Solver options (see below).  If not given, the
0037 %                         options in the global variable CMPI_OPTIONS are
0038 %                         used (if defined).
0039 %                             MaxTime     Maximum solution time (seconds)
0040 %                             MaxIter     Maximum simplex iterations
0041 %                             MaxNodes    Maximum number of nodes
0042 %                             Display     Turn reporting to the screen
0043 %                                         'on' or 'off' (default)
0044 %                             FeasTol     Feasibility tolerance
0045 %                             IntFeasTol  Integer feasibility tolerance
0046 %                             OptTol      Optimality tolerance
0047 %                             AbsOptTol   Absolute optimality tolerance
0048 %               Q         Quadratic objective matrix.  If given, the
0049 %                         problem is solved as a MIQP.  See CONVERT_MIQP
0050 %                         for details on Q and related fields.
0051 %
0052 %   Outputs
0053 %   SOL     Solution structure with fields:
0054 %               x       Optimal vector
0055 %               val     Objective values (sense*c*x)
0056 %               flag    Exit flag.  Possible values are:
0057 %                           1   Not started
0058 %                           2   Optimal
0059 %                           3   Infeasible
0060 %                           4   Infeasible or unbounded
0061 %                           5   Unbounded
0062 %                           6   Objective worse than user cutoff
0063 %                           7   Iteration limit reached
0064 %                           8   Node limit reached
0065 %                           9   Time limit reached
0066 %                           10  Solution limit reached
0067 %                           11  User interruption
0068 %                           12  Numerical difficulties
0069 %                           13  Suboptimal solution
0070 %               output  Other solver-specific output
0071 
0072 solver = cmpi.get_solver();
0073 
0074 if ~issparse(mip.A)
0075     mip.A = sparse(mip.A);
0076 end
0077 
0078 if ~isfield(mip,'sense') || isempty(mip.sense)
0079     mip.sense = 1;
0080 end
0081 
0082 if ~isfield(mip,'options') || isempty(mip.options)
0083     mip.options = cmpi.get_options();
0084 end
0085 
0086 % preserve size before conversion
0087 N = size(mip.A,2);
0088 
0089 mip = cmpi.convert_indicators(mip);
0090 
0091 qp = ~isempty(cmpi.miqp_type(mip));
0092 
0093 if qp
0094     mip = cmpi.convert_miqp(mip);
0095 end
0096 
0097 switch solver
0098     case 'gurobi'
0099         opts = cmpi.set_gurobi_opts(mip.options);
0100         if qp
0101             [opts.QP.qrow,opts.QP.qcol,opts.QP.qval] = find(mip.Q);
0102             opts.QP.qrow = int32(opts.QP.qrow(:)');
0103             opts.QP.qcol = int32(opts.QP.qcol(:)');
0104             opts.QP.qval = opts.QP.qval(:)';
0105         end
0106         [sol.x,sol.val,sol.flag,sol.output] = ...
0107             gurobi_mex(mip.obj, ...
0108                        mip.sense, ...
0109                        mip.A, ...
0110                        mip.b, ...
0111                        mip.ctypes, ...
0112                        mip.lb, ...
0113                        mip.ub, ...
0114                        mip.vartypes, ...
0115                        opts);
0116                    
0117     case 'cplex'        
0118         opts = cmpi.set_cplex_opts(mip.options);
0119         
0120         mip.b = mip.b(:);
0121         mip.vartypes = upper(mip.vartypes);
0122 
0123         Aineq = [ mip.A(mip.ctypes == '<',:); 
0124                  -mip.A(mip.ctypes == '>',:)];
0125         bineq = [ mip.b(mip.ctypes == '<');
0126                  -mip.b(mip.ctypes == '>')];
0127              
0128         Aeq = mip.A(mip.ctypes == '=',:);
0129         beq = mip.b(mip.ctypes == '=');
0130         if qp
0131             [sol.x,sol.val,~,sol.output] = ...
0132                 cplexmiqp(mip.sense.*mip.Q, ...
0133                           mip.sense.*mip.obj(:), ...
0134                           Aineq, bineq, ...
0135                           Aeq, beq, ...
0136                           [], [], [], ...
0137                           mip.lb(:), mip.ub(:), ...
0138                           mip.vartypes(:)', ...
0139                           [], ...
0140                           opts);
0141         else
0142             [sol.x,sol.val,~,sol.output] = ...
0143                 cplexmilp(mip.sense*mip.obj(:), ...
0144                           Aineq, bineq, ...
0145                           Aeq, beq, ...
0146                           [], [], [], ...
0147                           mip.lb(:), mip.ub(:), ...
0148                           mip.vartypes(:)', ...
0149                           [], ...
0150                           opts);
0151         end
0152 
0153         sol.val = mip.sense*sol.val;
0154                    
0155         sol.flag = cmpi.get_cplex_flag(sol.output.cplexstatus);
0156         
0157     otherwise
0158         error('Unrecognized solver: %s',solver);
0159 end
0160 
0161 % remove added variables
0162 if ~isempty(sol.x)
0163     sol.x = sol.x(1:N);
0164 end
0165
```

---

Generated on Thu 11-Aug-2011 15:06:22 by **m2html** © 2005
