# Supplementary material for: TIGER: Toolbox for integrating genome-scale metabolic models, expression data, and transcriptional regulatory networks
Source: BMC Syst Biol. 2011 Sep 23;5:147. doi: 10.1186/1752-0509-5-147 (PMC3224351; doi:10.1186/1752-0509-5-147)
Supplement: Additional file 2 — TIGER source code. Source code, documentation, and tutorials are also available online at http://bme.virginia.edu/csbl/downloads/ or http://csbl.bitbucket.org/tiger. [file 1752-0509-5-147-S2.GZ › tiger/doc/m2html/tiger/add_row.html]

Description of add\_row


Home > tiger > add\_row.m

# add\_row

## PURPOSE

**Add a row to a TIGER model structure**

## SYNOPSIS

**function [tiger] = add\_row(tiger,A,ctype,b,name,ind,indtype)**

## DESCRIPTION

```
 ADD_ROW  Add a row to a TIGER model structure

   [TIGER] = ADD_ROW(TIGER,N)
   [TIGER] = ADD_ROW(TIGER,A,CTYPE,B,NAME,IND,INDTYPE)

   Add a row to an existing structure, updating the corresponding 
   vectors.  The following default values are used:
       A        row of zeros
       CTYPE    '='
       B        0
       NAME     'ROWi', where i is the row index
       IND      0
       INDTYPE  ' '

   If called as ADD_ROW(TIGER,N), N rows are added with the default
   values.  If only a single value is given for each argument, it will
   be repeated for all new rows.
```

## CROSS-REFERENCE INFORMATION

This function calls:

- check\_tiger Check size and orientation of TIGER fields
- array2names Create a cell of names from an array of numbers
- expand\_to Expand a vector to length N
- fill\_to Fill a short to empty vector

This function is called by:

- convert\_indicators Convert indicators to MILP constraints
- convert\_miqp Prepare a MIQP for solution
- add\_diff Add difference variables toa TIGER model
- add\_growth\_constraint Add minimum growth constraint to a model.
- bind\_var Bind variables to a indicator variable
- cobra\_to\_elf Create an ELF model from a COBRA structure
- test\_\_diffadj
- test\_\_miqp
- diffadj Formulate and solve the differential adjustment problem

## SOURCE CODE

```
0001 function [tiger] = add_row(tiger,A,ctype,b,name,ind,indtype)
0002 % ADD_ROW  Add a row to a TIGER model structure
0003 %
0004 %   [TIGER] = ADD_ROW(TIGER,N)
0005 %   [TIGER] = ADD_ROW(TIGER,A,CTYPE,B,NAME,IND,INDTYPE)
0006 %
0007 %   Add a row to an existing structure, updating the corresponding
0008 %   vectors.  The following default values are used:
0009 %       A        row of zeros
0010 %       CTYPE    '='
0011 %       B        0
0012 %       NAME     'ROWi', where i is the row index
0013 %       IND      0
0014 %       INDTYPE  ' '
0015 %
0016 %   If called as ADD_ROW(TIGER,N), N rows are added with the default
0017 %   values.  If only a single value is given for each argument, it will
0018 %   be repeated for all new rows.
0019 
0020 [m,n] = size(tiger.A);
0021 loc = m+1;
0022 
0023 if nargin < 2 || isempty(A)
0024     A = [];
0025 elseif length(A) == 1
0026     A = zeros(A,n);
0027 end
0028 
0029 if nargin < 3 || isempty(ctype), ctype = '='; end
0030 
0031 if nargin < 4 || isempty(b), b = 0; end
0032 
0033 if nargin < 5 || isempty(name)
0034     name = {};
0035 elseif isa(name,'char')
0036     name = {name};
0037 end
0038 
0039 if nargin < 6 || isempty(ind), ind = 0; end
0040 
0041 if nargin < 7 || isempty(indtype), indtype = ' '; end
0042 
0043 N = max([length(name),  ...
0044          length(ctype), ...
0045          length(b),     ...
0046          size(A,1),     ...
0047          length(ind),   ...
0048          length(indtype)]);
0049 
0050 A = expand_to(A,[N n]);
0051 ctype = fill_to(ctype,N);
0052 b = fill_to(b,N);
0053 ind = fill_to(ind,N);
0054 indtype = fill_to(indtype,N);
0055 if length(name) < N
0056     if length(name) == 1
0057         name = array2names(name{1},loc:loc+N-1);
0058     else
0059         name = array2names('ROW%i',loc:loc+N-1);
0060     end
0061 end
0062 
0063 locs = loc + (0:N-1);
0064 
0065 tiger.A(locs,:) = A;
0066 tiger.ctypes(locs) = ctype;
0067 tiger.b(locs) = b;
0068 tiger.rownames(locs) = name;
0069 tiger.ind(locs) = ind;
0070 tiger.indtypes(locs) = indtype;
0071 
0072 tiger.param.rule_id(end+(1:N)) = 0;
0073 
0074 tiger = check_tiger(tiger);
```

---

Generated on Thu 11-Aug-2011 15:06:22 by **m2html** © 2005
