# Supplementary material for: TIGER: Toolbox for integrating genome-scale metabolic models, expression data, and transcriptional regulatory networks
Source: BMC Syst Biol. 2011 Sep 23;5:147. doi: 10.1186/1752-0509-5-147 (PMC3224351; doi:10.1186/1752-0509-5-147)
Supplement: Additional file 2 — TIGER source code. Source code, documentation, and tutorials are also available online at http://bme.virginia.edu/csbl/downloads/ or http://csbl.bitbucket.org/tiger. [file 1752-0509-5-147-S2.GZ › tiger/doc/m2html/tiger/find_exchange_rxns.html]

Description of find\_exchange\_rxns


Home > tiger > find\_exchange\_rxns.m

# find\_exchange\_rxns

## PURPOSE

**Find locations of exchange reactions**

## SYNOPSIS

**function [ex\_locs] = find\_exchange\_rxns(model)**

## DESCRIPTION

```
 FIND_EXCHANGE_RXNS  Find locations of exchange reactions

   Returns the indices of the reactions in an S matrix that only have one
   nonzero entry.
```

## CROSS-REFERENCE INFORMATION

This function calls:


This function is called by:

- show\_exchange\_rxns Show exchange reactions (and fluxes)

## SOURCE CODE

```
0001 function [ex_locs] = find_exchange_rxns(model)
0002 % FIND_EXCHANGE_RXNS  Find locations of exchange reactions
0003 %
0004 %   Returns the indices of the reactions in an S matrix that only have one
0005 %   nonzero entry.
0006 
0007 S = model.S;
0008 
0009 S(S ~= 0) = 1;
0010 ex_locs = find(sum(S,1) == 1)';
```

---

Generated on Thu 11-Aug-2011 15:06:22 by **m2html** © 2005
