# Supplementary material for: TIGER: Toolbox for integrating genome-scale metabolic models, expression data, and transcriptional regulatory networks
Source: BMC Syst Biol. 2011 Sep 23;5:147. doi: 10.1186/1752-0509-5-147 (PMC3224351; doi:10.1186/1752-0509-5-147)
Supplement: Additional file 2 — TIGER source code. Source code, documentation, and tutorials are also available online at http://bme.virginia.edu/csbl/downloads/ or http://csbl.bitbucket.org/tiger. [file 1752-0509-5-147-S2.GZ › tiger/doc/m2html/tiger/convert_ids.html]

Description of convert\_ids


Home > tiger > convert\_ids.m

# convert\_ids

## PURPOSE

**Create name, indices, and logical indices from an array**

## SYNOPSIS

**function [names,idxs,logic] = convert\_ids(all\_names,ids,type)**

## DESCRIPTION

```
 CONVERT_IDS  Create name, indices, and logical indices from an array

   [NAMES,IDXS,LOGIC] = CONVERT_IDS(ALL_NAMES,IDS)
   [IDS] = CONVERT_IDS(ALL_NAMES,IDS,TYPE)

   Creates a cell of names (NAMES), linear indices (IDXS), and logical
   indices (LOGIC) for the values IDS in the cell ALL_NAMES.  IDS can be 
   any of the forms previously mentioned.

   If three inputs are given, then only a single value is returned.  The
   type of id returned is determined by type:
       'name'    -> NAMES
       'index'   -> IDXS
       'logical' -> LOGIC

   Example:
       all_names = {'a','b','c','d'};
       ids = [2 4];
       [n,i,l] = convert_ids(all_names,ids)
           n = {'b','d'}
           i = [2 4]
           l = [0 1 0 1]

       idx = convert_ids(all_names,'c','index')
           idx = 3
```

## CROSS-REFERENCE INFORMATION

This function calls:


This function is called by:

- add\_diff Add difference variables toa TIGER model
- bind\_var Bind variables to a indicator variable
- mea
- fva Flux Variability Analysis
- minimal\_genome Calculate a minimal genome
- moma Minimization of Metabolic Adjustment
- remove\_column Remove column(s) from a TIGER model
- remove\_row Remove row(s) from a TIGER model
- set\_fieldval Set values in a TIGER structure field
- single\_gene\_ko Perform single gene knockout simulations
- minimal\_genome\_study
- diffadj Formulate and solve the differential adjustment problem
- gimme Gene Inactivity Moderated by Metabolism and Expression
- imat Integrative Metabolic Analysis Tool

## SOURCE CODE

```
0001 function [names,idxs,logic] = convert_ids(all_names,ids,type)
0002 % CONVERT_IDS  Create name, indices, and logical indices from an array
0003 %
0004 %   [NAMES,IDXS,LOGIC] = CONVERT_IDS(ALL_NAMES,IDS)
0005 %   [IDS] = CONVERT_IDS(ALL_NAMES,IDS,TYPE)
0006 %
0007 %   Creates a cell of names (NAMES), linear indices (IDXS), and logical
0008 %   indices (LOGIC) for the values IDS in the cell ALL_NAMES.  IDS can be
0009 %   any of the forms previously mentioned.
0010 %
0011 %   If three inputs are given, then only a single value is returned.  The
0012 %   type of id returned is determined by type:
0013 %       'name'    -> NAMES
0014 %       'index'   -> IDXS
0015 %       'logical' -> LOGIC
0016 %
0017 %   Example:
0018 %       all_names = {'a','b','c','d'};
0019 %       ids = [2 4];
0020 %       [n,i,l] = convert_ids(all_names,ids)
0021 %           n = {'b','d'}
0022 %           i = [2 4]
0023 %           l = [0 1 0 1]
0024 %
0025 %       idx = convert_ids(all_names,'c','index')
0026 %           idx = 3
0027 
0028 if isa(ids,'logical')
0029     logic = ids;
0030     names = all_names(logic);
0031     idxs  = find(logic);
0032 elseif isa(ids,'double')
0033     idxs  = ids;
0034     logic = ismember(1:length(all_names),idxs);
0035     names = all_names(idxs);
0036 else
0037     names = ids;
0038     logic = ismember(all_names,names);
0039     [~,idxs] = ismember(names,all_names);
0040 end
0041 
0042 if nargin == 3
0043     assert(nargout == 1,'only one output allowed when TYPE is given');
0044     switch validatestring(type,{'name','index','logical'})
0045         case 'name'
0046             names = names;
0047         case 'index'
0048             names = idxs;
0049         case 'logical'
0050             names = logic;
0051     end
0052 end
```

---

Generated on Thu 11-Aug-2011 15:06:22 by **m2html** © 2005
