# Supplementary material for: TIGER: Toolbox for integrating genome-scale metabolic models, expression data, and transcriptional regulatory networks
Source: BMC Syst Biol. 2011 Sep 23;5:147. doi: 10.1186/1752-0509-5-147 (PMC3224351; doi:10.1186/1752-0509-5-147)
Supplement: Additional file 2 — TIGER source code. Source code, documentation, and tutorials are also available online at http://bme.virginia.edu/csbl/downloads/ or http://csbl.bitbucket.org/tiger. [file 1752-0509-5-147-S2.GZ › tiger/doc/m2html/tiger/tie/index.html]

Index for Directory tiger/tie


|  |  |
| --- | --- |
| Master index | Index for tiger/tie |

# Index for tiger/tie

## Matlab files in this directory:

|  |  |
| --- | --- |
| check\_transition\_matrix | Validate a DIFFADJ matrix of transitions |
| diffadj | Formulate and solve the differential adjustment problem |
| find\_conditions | Find conditions corresponding to a transition |
| find\_optimal\_states | Find optimal binary states |
| gimme | Gene Inactivity Moderated by Metabolism and Expression |
| imat | Integrative Metabolic Analysis Tool |
| made | Metabolic Adjustment by Differential Expression |
| show\_made\_results | Summarize results from the MADE algorithm |

---

Generated on Thu 11-Aug-2011 15:06:20 by **m2html** © 2005
