# Supplementary material for: TIGER: Toolbox for integrating genome-scale metabolic models, expression data, and transcriptional regulatory networks
Source: BMC Syst Biol. 2011 Sep 23;5:147. doi: 10.1186/1752-0509-5-147 (PMC3224351; doi:10.1186/1752-0509-5-147)
Supplement: Additional file 2 — TIGER source code. Source code, documentation, and tutorials are also available online at http://bme.virginia.edu/csbl/downloads/ or http://csbl.bitbucket.org/tiger. [file 1752-0509-5-147-S2.GZ › tiger/doc/m2html/tiger/tie/imat.html]

Description of imat


Home > tiger > tie > imat.m

# imat

## PURPOSE

**Integrative Metabolic Analysis Tool**

## SYNOPSIS

**function [gene\_levels,genes,sol,tiger] = imat(model,levels,varargin)**

## DESCRIPTION

```
 IMAT  Integrative Metabolic Analysis Tool

   [GENE_LEVELS,GENES,SOL,TIGER] = IMAT(MODEL,LEVELS,...parameters...)

   Integrate expression levels with a metabolic model using a modified
   iMAT algorithm [Shlomi, et al. (2008), Nat Biotechnol].  This
   implementation uses expression levels for each gene rather than each
   reaction.

   LEVELS classifies each gene as either lowly (0), normally (1), or
   highly (2) expressed.  If a reaction flux 'v' has bounds
   lb <= v <= ub, then

       LEVELS(i) = 0 --> -eps <= v_i <= eps
       LEVELS(i) = 1 -->   lb <= v_i <= ub
       LEVELS(i) = 2 --> v_i <= -eps OR v_i >= eps

   Inputs
   MODEL   TIGER or COBRA model.  If a TIGER model is given, a COBRA
           model is extracted and re-converted to a TIGER model with
           multilevel GPRs.
   LEVELS  Expression state (0, 1, or 2) for each gene.

   Parameters
   'gene_names'  Cell array of names for genes in LEVELS.  If not given,
                 the default is TIGER.genes.
   'obj_frac'    Fraction of metabolic objective required in the 
                 resulting model (v_obj >= frac*v_obj_max). 
                 Default is 0.
   'flux_eps'    Small flux value used as 'eps' in the above constraints.
                 Default = 1e-3.
   'weights'     Optional weighting for each gene.  The penalty for each
                 gene is  WEIGHTS(i)*|GENES(i) - LEVELS(i)|.
                 Default is 1 for all genes.

   Outputs
   GENE_LEVELS  Expression levels returned by IMAT.
   GENES        Cell of gene names corresponding to GENE_LEVELS.
   SOL          Solution structure with details from the MILP solver.
   TIGER        TIGER model with GENE_LEVELS applied.
```

## CROSS-REFERENCE INFORMATION

This function calls:

- add\_column Add a column to a TIGER model structure
- add\_diff Add difference variables toa TIGER model
- add\_growth\_constraint Add minimum growth constraint to a model.
- add\_rule Add rules to a TIGER model
- cobra\_to\_tiger Convert a COBRA model to a TIGER model
- convert\_ids Create name, indices, and logical indices from an array
- extract\_cobra Extract the original COBRA model from a TIGER structure.
- set\_fieldval Set values in a TIGER structure field
- set\_var Set bounds on a variable
- cellzip Zip two cell arrays by a function
- find\_like Find matches in a cell of strings
- map Generate a new list by applying a function

This function is called by:

- test\_\_imat

## SUBFUNCTIONS

- function [cons] = make\_cons(ind,rxn)

## SOURCE CODE

```
0001 function [gene_levels,genes,sol,tiger] = imat(model,levels,varargin)
0002 % IMAT  Integrative Metabolic Analysis Tool
0003 %
0004 %   [GENE_LEVELS,GENES,SOL,TIGER] = IMAT(MODEL,LEVELS,...parameters...)
0005 %
0006 %   Integrate expression levels with a metabolic model using a modified
0007 %   iMAT algorithm [Shlomi, et al. (2008), Nat Biotechnol].  This
0008 %   implementation uses expression levels for each gene rather than each
0009 %   reaction.
0010 %
0011 %   LEVELS classifies each gene as either lowly (0), normally (1), or
0012 %   highly (2) expressed.  If a reaction flux 'v' has bounds
0013 %   lb <= v <= ub, then
0014 %
0015 %       LEVELS(i) = 0 --> -eps <= v_i <= eps
0016 %       LEVELS(i) = 1 -->   lb <= v_i <= ub
0017 %       LEVELS(i) = 2 --> v_i <= -eps OR v_i >= eps
0018 %
0019 %   Inputs
0020 %   MODEL   TIGER or COBRA model.  If a TIGER model is given, a COBRA
0021 %           model is extracted and re-converted to a TIGER model with
0022 %           multilevel GPRs.
0023 %   LEVELS  Expression state (0, 1, or 2) for each gene.
0024 %
0025 %   Parameters
0026 %   'gene_names'  Cell array of names for genes in LEVELS.  If not given,
0027 %                 the default is TIGER.genes.
0028 %   'obj_frac'    Fraction of metabolic objective required in the
0029 %                 resulting model (v_obj >= frac*v_obj_max).
0030 %                 Default is 0.
0031 %   'flux_eps'    Small flux value used as 'eps' in the above constraints.
0032 %                 Default = 1e-3.
0033 %   'weights'     Optional weighting for each gene.  The penalty for each
0034 %                 gene is  WEIGHTS(i)*|GENES(i) - LEVELS(i)|.
0035 %                 Default is 1 for all genes.
0036 %
0037 %   Outputs
0038 %   GENE_LEVELS  Expression levels returned by IMAT.
0039 %   GENES        Cell of gene names corresponding to GENE_LEVELS.
0040 %   SOL          Solution structure with details from the MILP solver.
0041 %   TIGER        TIGER model with GENE_LEVELS applied.
0042 
0043 p = inputParser;
0044 p.addParamValue('gene_names',model.genes);
0045 p.addParamValue('obj_frac',0);
0046 p.addParamValue('flux_eps',1e-3);
0047 p.addParamValue('weights',1);
0048 p.parse(varargin{:});
0049 
0050 genes = p.Results.gene_names;
0051 frac = p.Results.obj_frac;
0052 flux_eps = p.Results.flux_eps;
0053 weights = p.Results.weights;
0054 
0055 RXN_PRE = 'RXN__';
0056 
0057 model = extract_cobra(model);
0058 tiger = cobra_to_tiger(model,true,'default_ub',2);
0059 
0060 if frac > 0
0061     tiger = add_growth_constraint(tiger,frac);
0062 end
0063 
0064 rxn_inds = find_like(['^' RXN_PRE],tiger.varnames);
0065 rxn_names = map(@(x) x(length(RXN_PRE)+1:end),rxn_inds);
0066 
0067 cons = cellzip(@make_cons,rxn_inds,rxn_names);
0068 tiger = add_rule(tiger,[cons{:}]);
0069    
0070 [tiger,valnames] = add_column(tiger,[],'i',levels,levels);
0071 [tiger,diffvars] = add_diff(tiger,genes,valnames);
0072 
0073 tiger = set_fieldval(tiger,'obj',diffvars,weights);
0074 sol = cmpi.solve_mip(tiger);
0075 if cmpi.is_acceptable_exit(sol)
0076     gene_levels = sol.x(convert_ids(tiger.varnames,genes,'index'));
0077     tiger = set_var(tiger,genes,gene_levels);
0078 else
0079     gene_levels = [];
0080 end
0081 
0082 
0083 function [cons] = make_cons(ind,rxn)
0084     cons = cell(1,2);
0085     cons{1} = sprintf('%s < 1 <=> (%s >= %f) & (%s <= %f)', ...
0086                       ind,rxn,-flux_eps,rxn,flux_eps);
0087     cons{2} = sprintf('%s > 1 <=> (%s <= %f) | (%s >= %f)', ...
0088                       ind,rxn,-flux_eps,rxn,flux_eps);
0089 end
0090 
0091 end
```

---

Generated on Thu 11-Aug-2011 15:06:22 by **m2html** © 2005
