# Supplementary material for: TIGER: Toolbox for integrating genome-scale metabolic models, expression data, and transcriptional regulatory networks
Source: BMC Syst Biol. 2011 Sep 23;5:147. doi: 10.1186/1752-0509-5-147 (PMC3224351; doi:10.1186/1752-0509-5-147)
Supplement: Additional file 2 — TIGER source code. Source code, documentation, and tutorials are also available online at http://bme.virginia.edu/csbl/downloads/ or http://csbl.bitbucket.org/tiger. [file 1752-0509-5-147-S2.GZ › tiger/doc/m2html/tiger/tie/find_optimal_states.html]

Description of find\_optimal\_states


Home > tiger > tie > find\_optimal\_states.m

# find\_optimal\_states

## PURPOSE

**Find optimal binary states**

## SYNOPSIS

**function [states,score,matched,total] = find\_optimal\_states(d,T,w)**

## DESCRIPTION

```
 FIND_OPTIMAL_STATES  Find optimal binary states

   [STATES,SCORE,MATCHED,TOTAL] = FIND_OPTIMAL_STATES(D,T,W)

   Returns a binary sequence STATES that most closely match the
   transitions in D (1 -> increase, -1 -> decrease, 0 -> no change).
   The columns in D are described by the transition matrix T (see MADE
   for details on T).  If given, the transitions are weighted by W; if
   unspecified, W is a vector of ones (i.e., the maximum number of
   transitions is matched).

   SCORE is the MADE score for STATES.  MATCHED is the number of
   transitions in D that are matched by STATES.  TOTAL is the number
   of transitions in D.
```

## CROSS-REFERENCE INFORMATION

This function calls:

- find\_conditions Find conditions corresponding to a transition
- int2bin Convert an integer to an array of binary values

This function is called by:

- test\_\_find\_optimal\_states
- made Metabolic Adjustment by Differential Expression

## SOURCE CODE

```
0001 function [states,score,matched,total] = find_optimal_states(d,T,w)
0002 % FIND_OPTIMAL_STATES  Find optimal binary states
0003 %
0004 %   [STATES,SCORE,MATCHED,TOTAL] = FIND_OPTIMAL_STATES(D,T,W)
0005 %
0006 %   Returns a binary sequence STATES that most closely match the
0007 %   transitions in D (1 -> increase, -1 -> decrease, 0 -> no change).
0008 %   The columns in D are described by the transition matrix T (see MADE
0009 %   for details on T).  If given, the transitions are weighted by W; if
0010 %   unspecified, W is a vector of ones (i.e., the maximum number of
0011 %   transitions is matched).
0012 %
0013 %   SCORE is the MADE score for STATES.  MATCHED is the number of
0014 %   transitions in D that are matched by STATES.  TOTAL is the number
0015 %   of transitions in D.
0016 
0017 if nargin < 3 || isempty(w)
0018     w = ones(size(d));
0019 end
0020 
0021 N = length(T);
0022 total = length(d);
0023 
0024 best_score   = -1e100;
0025 best_states  = zeros(1,N);
0026 best_matched = 0;
0027 for i = 0 : 2^(N-1)
0028     states  = int2bin(i,N);
0029     score   = 0;
0030     matched = 0;
0031     for j = 1 : total
0032         [cond1,cond2] = find_conditions(j,T);
0033         if d(j) == 0
0034             score = score - w(j)*(states(cond1) ~= states(cond2));
0035         else
0036             d_obs = states(cond2) - states(cond1);
0037             score = score - w(j)*abs(d_obs - d(j));
0038         end
0039         matched = matched + (states(cond2) - states(cond1) == d(j));
0040     end
0041     if score > best_score
0042         best_score   = score;
0043         best_states  = states;
0044         best_matched = matched;
0045     end
0046 end
0047 
0048 states  = best_states;
0049 score   = best_score;
0050 matched = best_matched;
```

---

Generated on Thu 11-Aug-2011 15:06:22 by **m2html** © 2005
