# Supplementary material for: TIGER: Toolbox for integrating genome-scale metabolic models, expression data, and transcriptional regulatory networks
Source: BMC Syst Biol. 2011 Sep 23;5:147. doi: 10.1186/1752-0509-5-147 (PMC3224351; doi:10.1186/1752-0509-5-147)
Supplement: Additional file 2 — TIGER source code. Source code, documentation, and tutorials are also available online at http://bme.virginia.edu/csbl/downloads/ or http://csbl.bitbucket.org/tiger. [file 1752-0509-5-147-S2.GZ › tiger/doc/m2html/tiger/tie/check_transition_matrix.html]

Description of check\_transition\_matrix


Home > tiger > tie > check\_transition\_matrix.m

# check\_transition\_matrix

## PURPOSE

**Validate a DIFFADJ matrix of transitions**

## SYNOPSIS

**function [T] = check\_transition\_matrix(T,ncond,ntrans)**

## DESCRIPTION

```
 CHECK_TRANSITION_MATRIX  Validate a DIFFADJ matrix of transitions

   [T] = CHECK_TRANSITION_MATRIX(T,NCOND,NTRANS)

   Asserts that a transition matrix T is correct for a dataset with NCOND
   conditions and NTRANS transitions.  An error is raised if T is
   incorrect.  If T is empty, the default transition structure
   (1 -> 2 -> ... -> NCOND) is created.  The validated T is returned.

   See DIFFADJ and its related function for more information on the
   transition matrix structure.
```

## CROSS-REFERENCE INFORMATION

This function calls:


This function is called by:

- diffadj Formulate and solve the differential adjustment problem
- made Metabolic Adjustment by Differential Expression

## SOURCE CODE

```
0001 function [T] = check_transition_matrix(T,ncond,ntrans)
0002 % CHECK_TRANSITION_MATRIX  Validate a DIFFADJ matrix of transitions
0003 %
0004 %   [T] = CHECK_TRANSITION_MATRIX(T,NCOND,NTRANS)
0005 %
0006 %   Asserts that a transition matrix T is correct for a dataset with NCOND
0007 %   conditions and NTRANS transitions.  An error is raised if T is
0008 %   incorrect.  If T is empty, the default transition structure
0009 %   (1 -> 2 -> ... -> NCOND) is created.  The validated T is returned.
0010 %
0011 %   See DIFFADJ and its related function for more information on the
0012 %   transition matrix structure.
0013 
0014 if isempty(T)
0015     T = zeros(ncond);
0016     for i = 1 : ntrans
0017         T(i,i+1) = i;
0018     end
0019 end
0020 
0021 assert(all(size(T) == [ncond,ncond]), 'T not square or wrong size');
0022 assert(length(find(T)) == ntrans, 'T does not match w and d');
0023 assert(all(ismember(1:ntrans,T(:))), 'T is missing transition indices');
```

---

Generated on Thu 11-Aug-2011 15:06:22 by **m2html** © 2005
