# Supplementary material for: TIGER: Toolbox for integrating genome-scale metabolic models, expression data, and transcriptional regulatory networks
Source: BMC Syst Biol. 2011 Sep 23;5:147. doi: 10.1186/1752-0509-5-147 (PMC3224351; doi:10.1186/1752-0509-5-147)
Supplement: Additional file 2 — TIGER source code. Source code, documentation, and tutorials are also available online at http://bme.virginia.edu/csbl/downloads/ or http://csbl.bitbucket.org/tiger. [file 1752-0509-5-147-S2.GZ › tiger/doc/m2html/tiger/tie/find_conditions.html]

Description of find\_conditions


Home > tiger > tie > find\_conditions.m

# find\_conditions

## PURPOSE

**Find conditions corresponding to a transition**

## SYNOPSIS

**function [cond1,cond2] = find\_conditions(trans,T)**

## DESCRIPTION

```
 FIND_CONDITIONS  Find conditions corresponding to a transition

   [COND1,COND2] = FIND_CONDITIONS(TRANS,T)

   Finds which two conditions correspond to transition TRANS in a
   transition matrix T, i.e., where T(COND1,COND2) = TRANS.

   See DIFFADJ and its related function for more information on the
   transition matrix structure.
```

## CROSS-REFERENCE INFORMATION

This function calls:


This function is called by:

- find\_optimal\_states Find optimal binary states
- made Metabolic Adjustment by Differential Expression

## SOURCE CODE

```
0001 function [cond1,cond2] = find_conditions(trans,T)
0002 % FIND_CONDITIONS  Find conditions corresponding to a transition
0003 %
0004 %   [COND1,COND2] = FIND_CONDITIONS(TRANS,T)
0005 %
0006 %   Finds which two conditions correspond to transition TRANS in a
0007 %   transition matrix T, i.e., where T(COND1,COND2) = TRANS.
0008 %
0009 %   See DIFFADJ and its related function for more information on the
0010 %   transition matrix structure.
0011 
0012 [cond1,cond2] = ind2sub(size(T),find(T(:) == trans));
```

---

Generated on Thu 11-Aug-2011 15:06:22 by **m2html** © 2005
