# Supplementary material for: TIGER: Toolbox for integrating genome-scale metabolic models, expression data, and transcriptional regulatory networks
Source: BMC Syst Biol. 2011 Sep 23;5:147. doi: 10.1186/1752-0509-5-147 (PMC3224351; doi:10.1186/1752-0509-5-147)
Supplement: Additional file 2 — TIGER source code. Source code, documentation, and tutorials are also available online at http://bme.virginia.edu/csbl/downloads/ or http://csbl.bitbucket.org/tiger. [file 1752-0509-5-147-S2.GZ › tiger/doc/m2html/tiger/tie/gimme.html]

Description of gimme


Home > tiger > tie > gimme.m

# gimme

## PURPOSE

**Gene Inactivity Moderated by Metabolism and Expression**

## SYNOPSIS

**function [gene\_states,genes,sol,tiger] =gimme(tiger,express,thresh,varargin)**

## DESCRIPTION

```
 GIMME  Gene Inactivity Moderated by Metabolism and Expression

   [GENE_STATES,GENES,SOL,TIGER] = 
       GIMME(TIGER,EXPRESS,THRESH,...parameters...)

   Integrate expression data with a metabolic model using a modified
   GIMME algorithm [Becker & Palsson (2008), PLoS Comput Biol].  This
   implementation uses an integrated model to map expression values to
   the gene instead of averaging over each reaction.

   Inputs
   TIGER    TIGER model.  COBRA models will be converted to TIGER models
            with a warning.
   EXPRESS  Expression value for each gene.
   THRESH   Threshold for genes to be turned "on".  If not given, the
            default is mean(EXPRESS).

   Parameters
   'gene_names'  Cell array of names for genes in EXPRESS.  If not given,
                 the default is TIGER.genes.
   'obj_frac'    Fraction of metabolic objective required in the 
                 resulting model (v_obj >= frac*v_obj_max). 
                 Default is 0.3.

   Outputs
   GENE_STATES  Binary expression states calculated by GIMME.
   GENES        Cell of gene names corresponding to GENE_STATES.
   SOL          Solution structure with details from the MILP solver.
   TIGER        TIGER model with GENE_STATES applied.
```

## CROSS-REFERENCE INFORMATION

This function calls:

- add\_growth\_constraint Add minimum growth constraint to a model.
- assert\_tiger Assert that a structure is an TIGER model.
- convert\_ids Create name, indices, and logical indices from an array
- set\_var Set bounds on a variable

This function is called by:

- test\_\_gimme

## SOURCE CODE

```
0001 function [gene_states,genes,sol,tiger] = ...
0002                                       gimme(tiger,express,thresh,varargin)
0003 % GIMME  Gene Inactivity Moderated by Metabolism and Expression
0004 %
0005 %   [GENE_STATES,GENES,SOL,TIGER] =
0006 %       GIMME(TIGER,EXPRESS,THRESH,...parameters...)
0007 %
0008 %   Integrate expression data with a metabolic model using a modified
0009 %   GIMME algorithm [Becker & Palsson (2008), PLoS Comput Biol].  This
0010 %   implementation uses an integrated model to map expression values to
0011 %   the gene instead of averaging over each reaction.
0012 %
0013 %   Inputs
0014 %   TIGER    TIGER model.  COBRA models will be converted to TIGER models
0015 %            with a warning.
0016 %   EXPRESS  Expression value for each gene.
0017 %   THRESH   Threshold for genes to be turned "on".  If not given, the
0018 %            default is mean(EXPRESS).
0019 %
0020 %   Parameters
0021 %   'gene_names'  Cell array of names for genes in EXPRESS.  If not given,
0022 %                 the default is TIGER.genes.
0023 %   'obj_frac'    Fraction of metabolic objective required in the
0024 %                 resulting model (v_obj >= frac*v_obj_max).
0025 %                 Default is 0.3.
0026 %
0027 %   Outputs
0028 %   GENE_STATES  Binary expression states calculated by GIMME.
0029 %   GENES        Cell of gene names corresponding to GENE_STATES.
0030 %   SOL          Solution structure with details from the MILP solver.
0031 %   TIGER        TIGER model with GENE_STATES applied.
0032 
0033 tiger = assert_tiger(tiger);
0034 
0035 p = inputParser;
0036 p.addParamValue('gene_names',tiger.genes);
0037 p.addParamValue('obj_frac',0.3);
0038 p.parse(varargin{:});
0039 
0040 genes = p.Results.gene_names;
0041 frac = p.Results.obj_frac;
0042 
0043 assert(nargin >= 2,'GIMME requires at least two inputs');
0044 assert(length(express) == length(genes), ...
0045        'gene_names must match size of EXPRESS');
0046    
0047 if nargin < 3
0048     thresh = mean(express);
0049 end
0050 
0051 off_locs = express < thresh;
0052 off_genes = genes(off_locs);
0053 w = thresh - express(off_locs);
0054 
0055 [~,off_idxs] = convert_ids(tiger.varnames,off_genes);
0056 
0057 if frac > 0
0058     model = add_growth_constraint(tiger,frac);
0059 else
0060     model = tiger;
0061 end
0062 model.obj(:) = 0;
0063 model.obj(off_idxs) = w;
0064 sol = cmpi.solve_mip(model);
0065 
0066 if cmpi.is_acceptable_exit(sol)
0067     gene_states = ones(size(express));
0068     gene_states(off_locs) = round(sol.x(off_idxs));
0069     tiger = set_var(tiger,genes,gene_states);
0070 else
0071     gene_states = [];
0072 end
0073
```

---

Generated on Thu 11-Aug-2011 15:06:22 by **m2html** © 2005
