# Supplementary material for: TIGER: Toolbox for integrating genome-scale metabolic models, expression data, and transcriptional regulatory networks
Source: BMC Syst Biol. 2011 Sep 23;5:147. doi: 10.1186/1752-0509-5-147 (PMC3224351; doi:10.1186/1752-0509-5-147)
Supplement: Additional file 2 — TIGER source code. Source code, documentation, and tutorials are also available online at http://bme.virginia.edu/csbl/downloads/ or http://csbl.bitbucket.org/tiger. [file 1752-0509-5-147-S2.GZ › tiger/doc/m2html/tiger/tie/diffadj.html]

Description of diffadj


Home > tiger > tie > diffadj.m

# diffadj

## PURPOSE

**Formulate and solve the differential adjustment problem**

## SYNOPSIS

**function [states,sol,mip\_error,models] =diffadj(milp,vars,d,w,I,bounds,objs,fracs,verify)**

## DESCRIPTION

```
 DIFFADJ  Formulate and solve the differential adjustment problem

   [STATES,SOL] = DIFFADJ(MILP,VARS,D,W,I,BOUNDS,OBJS,FRACS,METHOD)

   Attempts to match changes between different conditions while
   retaining functional models.

   Inputs
   MILP    MILP model
   VARS    List of indices for variables to adjust
   D       List of differences for each transition:
                1 -> x(t) < x(t')
                0 -> x(t) = x(t')
               -1 -> x(t) > x(t')
           Columns correspond to conditions.  Each row 'i' is a variable
           with index VARS(i).
   W       List of weights for each transition.  Structure is the same
           as for D.
   I       Interaction matrix.  If I(i,j) = k, then the kth column in
           D and W describe the transition between conditions i and j.
           The number of nonzero entries in I should equal the number
           of columns in D and W.
   BOUNDS  Structure array of bounds for each condition:
               BOUNDS{i}.lb
               BOUNDS{i}.ub
           If empty, the bounds in MILP are used.
   OBJS    Cell array of objective coefficients for each condition.  If
           empty, MILP.obj is used.
   FRACS   Fractions of optimal growth that must be possible in each
           condition:
               obj'x >= FRACS(i)*obj'x_max
           If only a single number is given, it is used for every
           condition.  If empty, the default is 0.3.
   VERIFY  If true (default), resulting models are run to verify the 
           minimum objective flux.

   Outputs
   STATES     A |variables| x |conditions| matrix of states (levels) for
              each variable in each condition.
   SOL        Solution object from CMPI.  Contains the additional fields
                  'obj_vals'  Objective values through original models
                  'adj_vals'  Objective values through adjusted models
                  'verified'  True if obj_val(i) >= FRAC(i)*c'x_max
                  'mip'       Full MIP structure
                  'I'         Interaction matrix
                  'variables' Variable structure with fields:
                       'flux'  Reaction fluxes
                       'rxn'   Reaction indicator variables
                       'gene'  Gene indicator variables
   MIP_ERROR  True if an error was encourtered during the optimization.
   MODELS     Models with STATES applied to VARS.
```

## CROSS-REFERENCE INFORMATION

This function calls:

- add\_column Add a column to a TIGER model structure
- add\_diff Add difference variables toa TIGER model
- add\_growth\_constraint Add minimum growth constraint to a model.
- add\_row Add a row to a TIGER model structure
- convert\_ids Create name, indices, and logical indices from an array
- fba Run Flux Balance Analysis on a TIGER model.
- set\_var Set bounds on a variable
- check\_transition\_matrix Validate a DIFFADJ matrix of transitions
- array2names Create a cell of names from an array of numbers
- assert\_cell Assert that variable is a cell array.
- count Count the number of nonzero elements in a vector
- fill\_to Fill a short to empty vector
- map Generate a new list by applying a function

This function is called by:

- dare
- test\_\_diffadj
- made Metabolic Adjustment by Differential Expression

## SUBFUNCTIONS

- function [mip,pos\_idxs,neg\_idxs] = add\_nonbinding\_diff(mip,idxs1,idxs2)
- function [vars] = deparse\_sol(xall,models)

## SOURCE CODE

```
0001 function [states,sol,mip_error,models] = ...
0002                         diffadj(milp,vars,d,w,I,bounds,objs,fracs,verify)
0003 % DIFFADJ  Formulate and solve the differential adjustment problem
0004 %
0005 %   [STATES,SOL] = DIFFADJ(MILP,VARS,D,W,I,BOUNDS,OBJS,FRACS,METHOD)
0006 %
0007 %   Attempts to match changes between different conditions while
0008 %   retaining functional models.
0009 %
0010 %   Inputs
0011 %   MILP    MILP model
0012 %   VARS    List of indices for variables to adjust
0013 %   D       List of differences for each transition:
0014 %                1 -> x(t) < x(t')
0015 %                0 -> x(t) = x(t')
0016 %               -1 -> x(t) > x(t')
0017 %           Columns correspond to conditions.  Each row 'i' is a variable
0018 %           with index VARS(i).
0019 %   W       List of weights for each transition.  Structure is the same
0020 %           as for D.
0021 %   I       Interaction matrix.  If I(i,j) = k, then the kth column in
0022 %           D and W describe the transition between conditions i and j.
0023 %           The number of nonzero entries in I should equal the number
0024 %           of columns in D and W.
0025 %   BOUNDS  Structure array of bounds for each condition:
0026 %               BOUNDS{i}.lb
0027 %               BOUNDS{i}.ub
0028 %           If empty, the bounds in MILP are used.
0029 %   OBJS    Cell array of objective coefficients for each condition.  If
0030 %           empty, MILP.obj is used.
0031 %   FRACS   Fractions of optimal growth that must be possible in each
0032 %           condition:
0033 %               obj'x >= FRACS(i)*obj'x_max
0034 %           If only a single number is given, it is used for every
0035 %           condition.  If empty, the default is 0.3.
0036 %   VERIFY  If true (default), resulting models are run to verify the
0037 %           minimum objective flux.
0038 %
0039 %   Outputs
0040 %   STATES     A |variables| x |conditions| matrix of states (levels) for
0041 %              each variable in each condition.
0042 %   SOL        Solution object from CMPI.  Contains the additional fields
0043 %                  'obj_vals'  Objective values through original models
0044 %                  'adj_vals'  Objective values through adjusted models
0045 %                  'verified'  True if obj_val(i) >= FRAC(i)*c'x_max
0046 %                  'mip'       Full MIP structure
0047 %                  'I'         Interaction matrix
0048 %                  'variables' Variable structure with fields:
0049 %                       'flux'  Reaction fluxes
0050 %                       'rxn'   Reaction indicator variables
0051 %                       'gene'  Gene indicator variables
0052 %   MIP_ERROR  True if an error was encourtered during the optimization.
0053 %   MODELS     Models with STATES applied to VARS.
0054 
0055 USE_NONBINDING_DIFF = true;
0056 
0057 if nargin < 5
0058     I = [];
0059 end
0060 
0061 if nargin < 6 || isempty(bounds)
0062     bounds = [];
0063 end
0064 
0065 if nargin < 7 || isempty(objs)
0066     objs = [];
0067 end
0068 
0069 if nargin < 8 || isempty(fracs)
0070     fracs = 0.3;
0071 end
0072 
0073 if nargin < 9 || isempty(verify)
0074     verify = true;
0075 end
0076 
0077 vars = convert_ids(milp.varnames,vars,'index');
0078 
0079 [nvars,ntrans] = size(w);
0080 ncond = ntrans + 1;
0081 
0082 I = check_transition_matrix(I,ncond,ntrans);
0083 
0084 % fill out fracs
0085 fracs = fill_to(fracs,ncond,0.3);
0086 
0087 % fill out bounds
0088 if isempty(bounds)
0089     bounds.lb = milp.lb;
0090     bounds.ub = milp.ub;
0091 end
0092 bounds = assert_cell(bounds);
0093 if length(bounds) == 1
0094     for i = 2 : ncond
0095         bounds{i} = bounds{1};
0096     end
0097 end
0098 
0099 % fill out objs
0100 if isempty(objs)
0101     objs = milp.obj;
0102 end
0103 objs = assert_cell(objs);
0104 if length(objs) == 1
0105     for i = 2 : ncond
0106         objs{i} = objs{1};
0107     end
0108 end
0109     
0110 % make MILPs for each condition
0111 milps = cell(1,ncond);
0112 obj_vals = zeros(1,ncond);
0113 for i = 1 : ncond
0114     milps{i} = milp;
0115     milps{i}.obj = objs{i};
0116     milps{i}.lb = bounds{i}.lb;
0117     milps{i}.ub = bounds{i}.ub;
0118     
0119     if fracs(i) > 0
0120         [milps{i},sol] = add_growth_constraint(milps{i},fracs(i));
0121         obj_vals(i) = sol.val;
0122     else
0123         obj_vals(i) = 0;
0124     end
0125 end
0126 
0127 colsA = size(milp.A,2);
0128 mip = cmpi.tile_mip(milps{:});
0129 
0130 % compute the index of variable V in condition C
0131 idxof = @(v,c) colsA*(c-1) + v;
0132 
0133 n_con = count(d(:) == 0);
0134 con_idx1 = zeros(1,n_con);
0135 con_idx2 = zeros(1,n_con);
0136 con_objs = zeros(1,n_con);
0137 con_offset = 0;
0138 % create the objective function
0139 for t = 1 : ntrans
0140     % find the interacting conditions
0141     [cond1,cond2] = ind2sub(size(I),find(I(:) == t));
0142     for v = 1 : nvars
0143         idx1 = idxof(vars(v),cond1);
0144         idx2 = idxof(vars(v),cond2);
0145         switch d(v,t)
0146             case {1}
0147                 % increasing
0148                 mip.obj(idx1) = mip.obj(idx1) + w(v,t);
0149                 mip.obj(idx2) = mip.obj(idx2) - w(v,t);
0150             case {-1}
0151                 % decreasing
0152                 mip.obj(idx1) = mip.obj(idx1) - w(v,t);
0153                 mip.obj(idx2) = mip.obj(idx2) + w(v,t);
0154             case {0}
0155                 % constant
0156                 con_offset = con_offset + 1;
0157                 con_idx1(con_offset) = idx1;
0158                 con_idx2(con_offset) = idx2;
0159                 con_objs(con_offset) = w(v,t);
0160         end
0161     end
0162 end
0163 
0164 % create constant variables
0165 if USE_NONBINDING_DIFF
0166     [mip,pos_idxs,neg_idxs] = add_nonbinding_diff(mip,con_idx1,con_idx2);
0167     mip.obj(pos_idxs) = con_objs;
0168     mip.obj(neg_idxs) = con_objs;
0169 else
0170     [mip,con_vars] = add_diff(mip,con_idx1,con_idx2);
0171     [~,con_idxs] = convert_ids(mip.varnames,con_vars);
0172     mip.obj(con_idxs) = con_objs;
0173 end
0174 
0175 sol = cmpi.solve_mip(mip);
0176 sol.mip = mip;
0177 sol.T = I;
0178 
0179 mip_error = ~cmpi.is_acceptable_exit(sol);
0180 if ~mip_error
0181     states = zeros(nvars,ncond);
0182     for c = 1 : ncond
0183         for v = 1 : nvars
0184             states(v,c) = sol.x(idxof(vars(v),c));
0185         end
0186     end
0187 
0188     % round binary variables
0189     [~,bins] = intersect(vars,find(mip.vartypes == 'b'));
0190     states(bins,:) = round(states(bins,:));
0191     
0192     models = cell(1,ncond);
0193     for i = 1 : ncond
0194         % copy models and apply gene states
0195         models{i} = set_var(milps{i},vars,[],states(:,i));
0196     end
0197     
0198     % verify solutions
0199     if verify
0200         sol.verified = false(1,ncond);
0201         sol.adj_vals = zeros(1,ncond);
0202         sol.obj_vals = obj_vals;
0203         for i = 1 : ncond
0204             % run FBA to verify the solution
0205             kosol = fba(models{i});
0206             sol.verified(i) = cmpi.is_acceptable_exit(kosol);
0207             if ~isempty(kosol.val)
0208                 sol.adj_vals(i) = kosol.val;
0209             end
0210         end
0211     end
0212     
0213     sol.variables = deparse_sol(sol.x,milps);
0214 else
0215     % solver did not return a solution
0216     states = [];
0217     models = {};
0218 end
0219 
0220 
0221 function [mip,pos_idxs,neg_idxs] = add_nonbinding_diff(mip,idxs1,idxs2)
0222     N = length(idxs1);
0223     
0224     [m,n] = size(mip.A);
0225     
0226     pos_idxs = n + (1:N);
0227     neg_idxs = n + N + (1:N);
0228     
0229     plb = zeros(N,1);
0230     pub = zeros(N,1);
0231     nlb = zeros(N,1);
0232     nub = zeros(N,1);
0233     varnames = [array2names('nbd_diff_pos%i',pos_idxs); ...
0234                 array2names('nbd_diff_neg%i',neg_idxs)];
0235     
0236     for i = 1 : N
0237         pub(i) = mip.ub(idxs1(i)) - mip.lb(idxs2(i));
0238         nub(i) = mip.ub(idxs2(i)) - mip.lb(idxs1(i));
0239     end
0240     mip = add_column(mip,varnames,'c',[plb;nlb],[pub;nub]);
0241     
0242     mip = add_row(mip,N);
0243     for i = 1 : N
0244         % add constraint var1 - var2 + p_slack - n_slack = 0
0245         mip.A(m+i,[idxs1(i) idxs2(i) pos_idxs(i) neg_idxs(i)]) ...
0246             = [1 -1 1 -1];
0247     end
0248     
0249     
0250 function [vars] = deparse_sol(xall,models)
0251     RXN_PRE = 'RXN__';
0252     genes = models{1}.genes;
0253     ncond = length(models);
0254     nrxns = size(models{1}.S,2);
0255     nvars = size(models{1}.A,2);
0256     
0257     vars = cell(1,ncond);
0258     
0259     rxn_names = map(@(x) [RXN_PRE x],models{1}.varnames(1:nrxns));
0260     [rxn_tf,rxn_idxs] = ismember(rxn_names,models{1}.varnames);
0261     [~,gene_idxs] = ismember(genes,models{1}.varnames);
0262     for i = 1 : ncond
0263         x = xall(nvars*(i-1)+(1:nvars));
0264         
0265         vars{i}.flux = x(1:nrxns);
0266         
0267         vars{i}.rxn = zeros(nrxns,1);
0268         vars{i}.rxn(rxn_tf) = x(rxn_idxs(rxn_tf));
0269         vars{i}.rxn(~rxn_tf) = -1;
0270         
0271         vars{i}.gene = x(gene_idxs);
0272     end
0273         
0274
```

---

Generated on Thu 11-Aug-2011 15:06:22 by **m2html** © 2005
