# Supplementary material for: TIGER: Toolbox for integrating genome-scale metabolic models, expression data, and transcriptional regulatory networks
Source: BMC Syst Biol. 2011 Sep 23;5:147. doi: 10.1186/1752-0509-5-147 (PMC3224351; doi:10.1186/1752-0509-5-147)
Supplement: Additional file 2 — TIGER source code. Source code, documentation, and tutorials are also available online at http://bme.virginia.edu/csbl/downloads/ or http://csbl.bitbucket.org/tiger. [file 1752-0509-5-147-S2.GZ › tiger/doc/m2html/tiger/tie/made.html]

Description of made


Home > tiger > tie > made.m

# made

## PURPOSE

**Metabolic Adjustment by Differential Expression**

## SYNOPSIS

**function [sol] = made(tiger,fold\_change,pvals,varargin)**

## DESCRIPTION

```
 MADE  Metabolic Adjustment by Differential Expression

   [SOL] = MADE(TIGER,FOLD_CHANGE,PVALS,...parameters...)

   Integrate gene expression data with metabolic models using the MADE
   algorithm [Jensen & Papin (2011), Bioinformatics].

   Inputs
   TIGER       TIGER model.  COBRA models will be converted to TIGER 
               models with a warning.
   FOLD_CHANGE Measured fold change from expression data.  Columns
               correspond to conditions, rows correspond to genes.
   PVALS       P-values for changes.  Format is the same as for
               FOLD_CHANGE.

   Parameters
   'gene_names' Cell array of names for genes in expression dataset.
                These correspond to the rows in FOLD_CHANGE and PVALS.
                If none is given, the rows correspond to TIGER.genes.
   'obj_frac'   Fraction of metabolic objective required in the resulting 
                model (v_obj >= frac*v_obj_max). Default is 0.3.  Input 
                can also be a vector giving a separate fraction for each 
                condition.
   'weighting'  Method to convert PVALS to weights.  Options include:
                   'log'     w(p) = -log(p)
                   'linear'  w(p) = 1 - p
                   'unit'    w(p) = 1
                   'none'    No transformation -- PVALS are weights and
                             FOLD_CHANGE is a direction matrix
   'bounds'     Structure array of condition-specific bounds.  For
                example, BOUNDS{i}.lb and BOUNDS{i}.ub are the lower
                and upper bounds for the ith condition.  If not
                specified, the bounds from MODEL are copied to each
                condition.
   'objs'       Cell array of condition-specific objectives.  If not
                specified, the objective MODEL.c is copied to each
                condition.
   'p_thresh'   Threshold above which a P-value is not considered
                significant.  If a gene increases with a P-value
                p > P_THRESH, then it will be held constant with P-value
                1 - p.  The default is 0.5.
   'p_eps'      P-values below P_EPS are considered equal to P_EPS.  This
                is used with log weighting to avoid taking the logarithm
                of very small P-values.  The default is 1e-10.
   'transition_matrix'   A matrix (T) describing the interaction between
                conditions.  If T(i,j) = k, then the kth column in
                FOLD_CHANGE describes the change in expression between
                condition i to condition j.  If not given, FOLD_CHANGE
                assumes 1 -> 2, 2 -> 3, ..., n-1 -> n.
   'remove_rev' Remove reversibility constraints from the model before
                converting to a MIP.  May improve performance if activity
                cycles are not a concern.  (Default = false)
   'theoretical_match'  Calculate the theoretical matches possible and
                adjust the match statistics.  (Default = true)
   'log_fold_change'  If true, onsider FOLD_CHANGE to be log fold change.
                (Default = false)
   'return_models'  If true, the models for each condition are returned.
                (Default = true)
   'verbose'    If true (default), a results table is printed.  Otherwise,
                MADE is silent.

   Output is a solution structure with the following fields:
   output      Solution structure with details from the MILP solver.
   gene_states Binary expression states calculated by MADE.  Columns
               correspond to conditions, rows are genes in both
               the model and the expression data.
   opt_states  Optimal binary expression states.  If no growth constraint
               was enforced, these would be the same as 'gene_states'.
   genes       Cell of gene names corresponding to the rows in
               'gene_states'.
   models      Cell of TIGER model structures with bounds set to
               the results of applying 'gene_states' expression levels.
   condition   Cell array of structures with fields:
                   max_obj_flux  Maximum objective fluxes for each
                                 condition.
                   adj_obj_flux  Adjusted objective fluxes after applying
                                 expression data.
                   flux_ratio    Ratio between adjusted and maximum
                                 objective fluxes.
   transition  Cell array of structures with fields:
                   increasing, increasing_matched
                       Number of gene increasing in transation, and the
                       number that were matched.
                   decreasing, decreasing_matched
                       Number of decresing genes.
                   constant, constant_matched
                       Number of genes with constant expression.
   D_data      Differences in expression in the expression data.  '1'
               indicates a significant increase, '-1' is a significant
               decrease in expression, and '0' is no significant change.
   D_matched   Same as for 'D_data', but for the gene states returned by
               MADE.
   D_optimal   Same as for 'D_data', but for the optimal gene alignment.
   total_transitions   Total number of transitions (number of genes times
                       the number of transitions.
   matched             Total number of transitions matched by MADE.
   theoretical_matches Total number of matches in the optimal alignment.
   match_percent           'matched' / 'total_transitions' * 100
   adjusted_match_percent  'matched' / 'theoretical_matches' * 100
   verified    Logical array indicating if the model for each condition
               can carry the minimum objective flux.
   variables   Variable cell array returned by DIFFADJ.
               See 'help diffadj' for more details.
```

## CROSS-REFERENCE INFORMATION

This function calls:

- assert\_tiger Assert that a structure is an TIGER model.
- check\_transition\_matrix Validate a DIFFADJ matrix of transitions
- diffadj Formulate and solve the differential adjustment problem
- find\_conditions Find conditions corresponding to a transition
- find\_optimal\_states Find optimal binary states
- show\_made\_results Summarize results from the MADE algorithm
- count Count the number of nonzero elements in a vector

This function is called by:

- test\_\_made

## SOURCE CODE

```
0001 function [sol] = made(tiger,fold_change,pvals,varargin)
0002 % MADE  Metabolic Adjustment by Differential Expression
0003 %
0004 %   [SOL] = MADE(TIGER,FOLD_CHANGE,PVALS,...parameters...)
0005 %
0006 %   Integrate gene expression data with metabolic models using the MADE
0007 %   algorithm [Jensen & Papin (2011), Bioinformatics].
0008 %
0009 %   Inputs
0010 %   TIGER       TIGER model.  COBRA models will be converted to TIGER
0011 %               models with a warning.
0012 %   FOLD_CHANGE Measured fold change from expression data.  Columns
0013 %               correspond to conditions, rows correspond to genes.
0014 %   PVALS       P-values for changes.  Format is the same as for
0015 %               FOLD_CHANGE.
0016 %
0017 %   Parameters
0018 %   'gene_names' Cell array of names for genes in expression dataset.
0019 %                These correspond to the rows in FOLD_CHANGE and PVALS.
0020 %                If none is given, the rows correspond to TIGER.genes.
0021 %   'obj_frac'   Fraction of metabolic objective required in the resulting
0022 %                model (v_obj >= frac*v_obj_max). Default is 0.3.  Input
0023 %                can also be a vector giving a separate fraction for each
0024 %                condition.
0025 %   'weighting'  Method to convert PVALS to weights.  Options include:
0026 %                   'log'     w(p) = -log(p)
0027 %                   'linear'  w(p) = 1 - p
0028 %                   'unit'    w(p) = 1
0029 %                   'none'    No transformation -- PVALS are weights and
0030 %                             FOLD_CHANGE is a direction matrix
0031 %   'bounds'     Structure array of condition-specific bounds.  For
0032 %                example, BOUNDS{i}.lb and BOUNDS{i}.ub are the lower
0033 %                and upper bounds for the ith condition.  If not
0034 %                specified, the bounds from MODEL are copied to each
0035 %                condition.
0036 %   'objs'       Cell array of condition-specific objectives.  If not
0037 %                specified, the objective MODEL.c is copied to each
0038 %                condition.
0039 %   'p_thresh'   Threshold above which a P-value is not considered
0040 %                significant.  If a gene increases with a P-value
0041 %                p > P_THRESH, then it will be held constant with P-value
0042 %                1 - p.  The default is 0.5.
0043 %   'p_eps'      P-values below P_EPS are considered equal to P_EPS.  This
0044 %                is used with log weighting to avoid taking the logarithm
0045 %                of very small P-values.  The default is 1e-10.
0046 %   'transition_matrix'   A matrix (T) describing the interaction between
0047 %                conditions.  If T(i,j) = k, then the kth column in
0048 %                FOLD_CHANGE describes the change in expression between
0049 %                condition i to condition j.  If not given, FOLD_CHANGE
0050 %                assumes 1 -> 2, 2 -> 3, ..., n-1 -> n.
0051 %   'remove_rev' Remove reversibility constraints from the model before
0052 %                converting to a MIP.  May improve performance if activity
0053 %                cycles are not a concern.  (Default = false)
0054 %   'theoretical_match'  Calculate the theoretical matches possible and
0055 %                adjust the match statistics.  (Default = true)
0056 %   'log_fold_change'  If true, onsider FOLD_CHANGE to be log fold change.
0057 %                (Default = false)
0058 %   'return_models'  If true, the models for each condition are returned.
0059 %                (Default = true)
0060 %   'verbose'    If true (default), a results table is printed.  Otherwise,
0061 %                MADE is silent.
0062 %
0063 %   Output is a solution structure with the following fields:
0064 %   output      Solution structure with details from the MILP solver.
0065 %   gene_states Binary expression states calculated by MADE.  Columns
0066 %               correspond to conditions, rows are genes in both
0067 %               the model and the expression data.
0068 %   opt_states  Optimal binary expression states.  If no growth constraint
0069 %               was enforced, these would be the same as 'gene_states'.
0070 %   genes       Cell of gene names corresponding to the rows in
0071 %               'gene_states'.
0072 %   models      Cell of TIGER model structures with bounds set to
0073 %               the results of applying 'gene_states' expression levels.
0074 %   condition   Cell array of structures with fields:
0075 %                   max_obj_flux  Maximum objective fluxes for each
0076 %                                 condition.
0077 %                   adj_obj_flux  Adjusted objective fluxes after applying
0078 %                                 expression data.
0079 %                   flux_ratio    Ratio between adjusted and maximum
0080 %                                 objective fluxes.
0081 %   transition  Cell array of structures with fields:
0082 %                   increasing, increasing_matched
0083 %                       Number of gene increasing in transation, and the
0084 %                       number that were matched.
0085 %                   decreasing, decreasing_matched
0086 %                       Number of decresing genes.
0087 %                   constant, constant_matched
0088 %                       Number of genes with constant expression.
0089 %   D_data      Differences in expression in the expression data.  '1'
0090 %               indicates a significant increase, '-1' is a significant
0091 %               decrease in expression, and '0' is no significant change.
0092 %   D_matched   Same as for 'D_data', but for the gene states returned by
0093 %               MADE.
0094 %   D_optimal   Same as for 'D_data', but for the optimal gene alignment.
0095 %   total_transitions   Total number of transitions (number of genes times
0096 %                       the number of transitions.
0097 %   matched             Total number of transitions matched by MADE.
0098 %   theoretical_matches Total number of matches in the optimal alignment.
0099 %   match_percent           'matched' / 'total_transitions' * 100
0100 %   adjusted_match_percent  'matched' / 'theoretical_matches' * 100
0101 %   verified    Logical array indicating if the model for each condition
0102 %               can carry the minimum objective flux.
0103 %   variables   Variable cell array returned by DIFFADJ.
0104 %               See 'help diffadj' for more details.
0105 
0106 % TODO  don't compute theoretical matches when opt_match is off
0107 
0108 % test inputs
0109 assert(nargin >= 3, 'MADE requires at least three inputs.');
0110 
0111 % make sure the input was an TIGER model
0112 tiger = assert_tiger(tiger);
0113 
0114 ntrans = size(fold_change,2);   % number of transitions
0115 ncond  = ntrans + 1;            % number of conditions
0116 
0117 assert(all(size(pvals) == size(fold_change)), ...
0118        'FOLD_CHANGE and PVALS must have the same dimensions');
0119 
0120 % parse input param/value pairs
0121 p = inputParser;
0122 
0123 p.addParamValue('gene_names',tiger.genes);
0124 
0125 p.addParamValue('obj_frac',0.3);
0126 
0127 valid_weights = {'log','linear','unit','none'};
0128 p.addParamValue('weighting','log',@(x) ismember(x,valid_weights));
0129 
0130 p.addParamValue('bounds',[]);
0131 p.addParamValue('objs',[]);
0132 
0133 pvalidate = @(x) validateattributes(x,'numeric', ...
0134                                     {'scalar','real','positive','<=',1});
0135 p.addParamValue('p_thresh',0.5,pvalidate);
0136 p.addParamValue('p_eps',1e-10,pvalidate);
0137 
0138 p.addParamValue('transition_matrix',[]);
0139 p.addParamValue('remove_rev',false);
0140 p.addParamValue('theoretical_match',true);
0141 p.addParamValue('log_fold_change',true);
0142 p.addParamValue('return_models',true);
0143 
0144 p.addParamValue('verbose',true);
0145 
0146 p.parse(varargin{:});
0147 
0148 verbose = p.Results.verbose;
0149 
0150 find_theor = p.Results.theoretical_match;
0151 
0152 % find the genes to match
0153 genes = p.Results.gene_names;
0154 [tf,gene_locs] = ismember(genes,tiger.varnames);
0155 genes = genes(tf);
0156 gene_locs = gene_locs(tf);
0157 fold_change = fold_change(tf,:);
0158 pvals = pvals(tf,:);
0159 
0160 ngenes = length(genes);
0161 
0162 % set frac for each condition
0163 frac = p.Results.obj_frac;
0164 if length(frac) == 1
0165     fracs = repmat(frac,1,ncond);
0166 end
0167 
0168 p_thresh = p.Results.p_thresh;
0169 p_eps = p.Results.p_eps;
0170 
0171 if strcmpi(p.Results.weighting,'none')
0172     % fold_change is a direction matrix, pvals are weights
0173     d = fold_change;
0174     w = pvals;
0175 else
0176     d = zeros(ngenes,ntrans);
0177     P = pvals;
0178 
0179     if p.Results.log_fold_change
0180         % correct for log fold change
0181         fold_change = fold_change + 1;
0182     end
0183     
0184     % convert significant fold changes to differences
0185     d(fold_change <  1.0 & pvals <= p_thresh) = -1;
0186     d(fold_change >= 1.0 & pvals <= p_thresh) =  1;
0187 
0188     % shift p-values above p_thresh onto [0, 1-p_thresh]
0189     P(pvals > p_thresh) = 1 - pvals(pvals > p_thresh);
0190 
0191     % convert p-values to weights
0192     switch lower(p.Results.weighting)
0193         case 'log'
0194             P(P < p_eps) = p_eps;  % avoid taking log of zero
0195             w = -log(P);
0196         case 'linear'
0197             w = 1 - P;
0198         case 'unit'
0199             w = ones(size(P));
0200     end
0201 end
0202 
0203 bounds = p.Results.bounds;
0204 objs = p.Results.objs;
0205 
0206 T = check_transition_matrix(p.Results.transition_matrix,ncond,ntrans);
0207 
0208 % determine theoretical matches
0209 if find_theor
0210     if verbose, fprintf('Finding optimal matches...'); end
0211     opt_states = zeros(ngenes,ncond);
0212     opt_matched = zeros(ngenes,1);
0213     for i = 1 : ngenes
0214         [opt_states(i,:),~,opt_matched(i)] ...
0215             = find_optimal_states(d(i,:),T,w(i,:));
0216     end
0217     if verbose, fprintf('done\n'); end
0218 end
0219 
0220 % run MADE
0221 [gene_states,diffadj_sol,diffadj_error,milps] ...
0222     = diffadj(tiger,gene_locs,d,w,T,bounds,objs,fracs);
0223 
0224 if diffadj_error
0225     fprintf('Error:  The model was infeasible.\n\n');
0226     sol = diffadj_sol;
0227     return
0228 end
0229 
0230 sol.output = diffadj_sol;
0231 
0232 sol.genes = genes;
0233 sol.gene_states = gene_states;
0234 sol.opt_states = opt_states;
0235 
0236 sol.condition = cell(1,ncond);
0237 for i = 1 : ncond
0238     sol.condition{i}.max_obj_flux = diffadj_sol.obj_vals(i);
0239     sol.condition{i}.adj_obj_flux = diffadj_sol.adj_vals(i);
0240     sol.condition{i}.flux_ratio = diffadj_sol.adj_vals(i) ...
0241                                       / diffadj_sol.obj_vals(i);
0242 end
0243 
0244 T = diffadj_sol.T;
0245 D = zeros(size(d));
0246 if find_theor
0247     Dopt = zeros(size(d));
0248 end
0249 sol.transition = cell(1,ntrans);
0250 for i = 1 : ntrans
0251     [t.condition1,t.condition2] = find_conditions(i,T);
0252     D(:,i) = round(gene_states(:,t.condition2) ...
0253                        - gene_states(:,t.condition1));
0254     if find_theor
0255         Dopt(:,i) = opt_states(:,t.condition2) ...
0256                         - opt_states(:,t.condition1);
0257     end
0258     
0259     t.increasing = count( d(:,i) == 1 );
0260     t.increasing_matched = count( d(:,i) == 1 & D(:,i) == 1 );
0261     
0262     t.decreasing = count( d(:,i) == -1 );
0263     t.decreasing_matched = count( d(:,i) == -1 & D(:,i) == -1 );
0264     
0265     t.constant = count( d(:,i) == 0 );
0266     t.constant_matched = count( d(:,i) == 0 & D(:,i) == 0 );
0267 
0268     sol.transition{i} = t;
0269 end
0270     
0271 sol.D_data = d;
0272 sol.D_matched = D;
0273 sol.D_optimal = Dopt;
0274 
0275 sol.total_transitions = numel(d);
0276 sol.matches = count(D(:) - d(:) == 0);
0277 sol.theoretical_matches = count(Dopt(:) - d(:) == 0);
0278 sol.match_percent = sol.matches / sol.total_transitions * 100;
0279 sol.adjusted_match_percent = sol.matches / sol.theoretical_matches * 100;
0280 
0281 if p.Results.return_models
0282     sol.models = milps;
0283     for i = 1 : ncond
0284         % remove the growth constraint
0285         sol.models{i}.A(end,:) = 0;
0286         sol.models{i}.b(end) = 0;
0287     end
0288 end
0289 sol.verified = diffadj_sol.verified;
0290 
0291 sol.variables = diffadj_sol.variables;
0292 
0293 if verbose
0294     show_made_results(sol);
0295 end
0296 
0297 
0298
```

---

Generated on Thu 11-Aug-2011 15:06:22 by **m2html** © 2005
