# Supplementary material for: TIGER: Toolbox for integrating genome-scale metabolic models, expression data, and transcriptional regulatory networks
Source: BMC Syst Biol. 2011 Sep 23;5:147. doi: 10.1186/1752-0509-5-147 (PMC3224351; doi:10.1186/1752-0509-5-147)
Supplement: Additional file 2 — TIGER source code. Source code, documentation, and tutorials are also available online at http://bme.virginia.edu/csbl/downloads/ or http://csbl.bitbucket.org/tiger. [file 1752-0509-5-147-S2.GZ › tiger/doc/m2html/tiger/tie/show_made_results.html]

Description of show\_made\_results


Home > tiger > tie > show\_made\_results.m

# show\_made\_results

## PURPOSE

**Summarize results from the MADE algorithm**

## SYNOPSIS

**function show\_made\_results(sol,varargin)**

## DESCRIPTION

```
 SHOW_MADE_RESULTS  Summarize results from the MADE algorithm

   SHOW_MADE_RESULTS(SOL,...params...)

   Displays a summary of the results from the MADE algorithm.  SOL is the
   solution structure returned by MADE.  The following parameters
   determine the information shown:
       'summary'  (default) Objective values for unconstrained and
                  adjusted models, counts of transitions matched for each
                  condition.
       'debug'    Show reactions that are "off" which carry nonzero flux.
                  Used to identify integrality errors.
       'all'      Show all of the above.
```

## CROSS-REFERENCE INFORMATION

This function calls:

- count Count the number of nonzero elements in a vector
- create\_table Format and display tabular data

This function is called by:

- made Metabolic Adjustment by Differential Expression

## SOURCE CODE

```
0001 function show_made_results(sol,varargin)
0002 % SHOW_MADE_RESULTS  Summarize results from the MADE algorithm
0003 %
0004 %   SHOW_MADE_RESULTS(SOL,...params...)
0005 %
0006 %   Displays a summary of the results from the MADE algorithm.  SOL is the
0007 %   solution structure returned by MADE.  The following parameters
0008 %   determine the information shown:
0009 %       'summary'  (default) Objective values for unconstrained and
0010 %                  adjusted models, counts of transitions matched for each
0011 %                  condition.
0012 %       'debug'    Show reactions that are "off" which carry nonzero flux.
0013 %                  Used to identify integrality errors.
0014 %       'all'      Show all of the above.
0015 
0016 SUMMARY = 1;
0017 DEBUG = 2;
0018 GENES = 3;
0019 
0020 to_show = false(1,3);
0021 to_show(SUMMARY) = ismember('summary',varargin) || isempty(varargin);
0022 to_show(DEBUG)   = ismember('debug',varargin);
0023 to_show(GENES)   = ismember('genes',varargin);
0024 if ismember('all',varargin)
0025     to_show = true(1,3);
0026 end
0027 
0028 if to_show(SUMMARY)
0029     fprintf(['\n\nMADE:  ', ...
0030              'Metabolic Adjustment by Differential Expression\n']);
0031     fprintf('------------------------------------------------------\n\n');
0032 
0033     fprintf('%i genes found in model with %i conditions.\n\n', ...
0034             length(sol.genes),length(sol.condition));
0035 
0036     fprintf('FBA results:\n');
0037     if ~all(sol.verified)
0038         fprintf('    *** Warning: At least one condition does ***\n');
0039         fprintf('    ***  not meet the objective constraint.  ***\n');
0040     end
0041     fprintf('Condition    Max Obj Flux    Adj Obj Flux    Ratio\n');
0042     for i = 1 : length(sol.condition)
0043         cond = sol.condition{i};
0044         fprintf('    %i         %10f      %10f      %3.2f\n',...
0045                 i,cond.max_obj_flux,cond.adj_obj_flux,cond.flux_ratio);
0046     end
0047 
0048     fprintf('\nGene counts:\n');
0049     fprintf('           |  Increasing    Decreasing      Constant\n');
0050     fprintf('Transition |  Fit / Data    Fit / Data    Fit / Data\n');
0051     for i = 1 : length(sol.transition)
0052         t = sol.transition{i};
0053         fprintf(' %2i ->%2i   | %4i / %4i   %4i / %4i   %4i / %4i\n', ...
0054                 t.condition1,t.condition2, ...
0055                 t.increasing_matched,t.increasing, ...
0056                 t.decreasing_matched,t.decreasing, ...
0057                 t.constant_matched,t.constant);
0058     end
0059 
0060     fprintf('\nTotal match:     %i / %i (%3.1f%%)\n', ...
0061             sol.matches,sol.total_transitions, ...
0062             sol.match_percent);
0063 
0064     % TODO  don't compute theoretical matches when opt_match is off
0065     fprintf('Adjusted match:  %i / %i (%3.1f%%)\n\n', ...
0066             sol.matches,sol.theoretical_matches, ...
0067             sol.adjusted_match_percent);
0068 end
0069 
0070 if to_show(DEBUG)
0071     N_RXNS_SHOW = 10;
0072     
0073     % debuging reactions
0074     vars = sol.variables{1};
0075     off_rxns = round(vars.rxn) == 0 & vars.flux ~= 0.0;
0076     if any(off_rxns)
0077         fluxes = vars.flux;
0078         fluxes(~off_rxns) = 0;
0079         [~,I] = sort(abs(fluxes(:)),1,'descend');
0080         fprintf('\nOff reactions with highest flux:\n');
0081         fprintf('  Rxn             Flux        Indicator\n');
0082         for i = 1 : min([N_RXNS_SHOW,count(off_rxns)])
0083             fprintf('%5i  %15f  %15f\n', ...
0084                     I(i),fluxes(I(i)),vars.rxn(I(i)));
0085         end
0086     else
0087         fprintf('\nNo flux found through non-integral reactions.\n');
0088     end
0089     fprintf('\n');
0090 end
0091 
0092 if to_show(GENES)
0093     % show condition lists
0094     [ngenes,ncond] = size(sol.gene_states);
0095     data = cell(ngenes,2*ncond - 1);
0096     columnlabels = cell(1,2*ncond - 1);
0097     for g = 1 : ngenes
0098         for i = 1 : ncond
0099             data{g,2*(i-1)+1} = num2str(sol.gene_states(g,i));
0100             columnlabels{2*(i-1)+1} = num2str(i);
0101             columnlabels{2*(i-1)+2} = ' ';
0102             
0103             if i == ncond
0104                 break;
0105             end
0106             
0107             if sol.D_data(g,i) == 1
0108                 spacer = ' / ';
0109             elseif sol.D_data(g,i) == -1
0110                 spacer = ' \\ ';
0111             else
0112                 spacer = ' - ';
0113             end
0114             if sol.D_matched(g,i) ~= sol.D_data(g,i)
0115                 spacer([1 end]) = '**';
0116             end
0117             data{g,2*(i-1)+2} = spacer;
0118         end
0119     end
0120     
0121     fprintf('\nGene states (* indicates transition mismatch)\n');
0122     create_table(data,'rowlabels',sol.genes,'columnlabels',columnlabels)
0123     fprintf('\n\n');
0124 end
0125 
0126
```

---

Generated on Thu 11-Aug-2011 15:06:22 by **m2html** © 2005
