# Supplementary material for: TIGER: Toolbox for integrating genome-scale metabolic models, expression data, and transcriptional regulatory networks
Source: BMC Syst Biol. 2011 Sep 23;5:147. doi: 10.1186/1752-0509-5-147 (PMC3224351; doi:10.1186/1752-0509-5-147)
Supplement: Additional file 2 — TIGER source code. Source code, documentation, and tutorials are also available online at http://bme.virginia.edu/csbl/downloads/ or http://csbl.bitbucket.org/tiger. [file 1752-0509-5-147-S2.GZ › tiger/doc/m2html/tiger/check_tiger.html]

Description of check\_tiger


Home > tiger > check\_tiger.m

# check\_tiger

## PURPOSE

**Check size and orientation of TIGER fields**

## SYNOPSIS

**function [tiger] = check\_tiger(tiger)**

## DESCRIPTION

```
 CHECK_TIGER  Check size and orientation of TIGER fields

   [TIGER] = CHECK_TIGER(TIGER)

   Checks that fields in a TIGER model structure are the correct size 
   and orientation.  Returns the validated TIGER model.
```

## CROSS-REFERENCE INFORMATION

This function calls:


This function is called by:

- add\_column Add a column to a TIGER model structure
- add\_row Add a row to a TIGER model structure
- add\_rule Add rules to a TIGER model

## SOURCE CODE

```
0001 function [tiger] = check_tiger(tiger)
0002 % CHECK_TIGER  Check size and orientation of TIGER fields
0003 %
0004 %   [TIGER] = CHECK_TIGER(TIGER)
0005 %
0006 %   Checks that fields in a TIGER model structure are the correct size
0007 %   and orientation.  Returns the validated TIGER model.
0008 
0009 col_fields = {'b','lb','ub','obj','varnames','rownames', ...
0010               'vartypes','ctypes','ind','indtypes'};
0011 
0012 [m,n] = size(tiger.A);
0013 
0014 m_fields = {'b','rownames','ctypes'};
0015 n_fields = {'lb','ub','obj','varnames','vartypes'};
0016 
0017 for i = 1 : length(col_fields)
0018     if size(tiger.(col_fields{i}),1) ~= length(tiger.(col_fields{i}))
0019         tiger.(col_fields{i}) = tiger.(col_fields{i})';
0020     end
0021 end
0022 
0023 for i = 1 : length(m_fields)
0024     assert(length(tiger.(m_fields{i})) == m, ...
0025            'field %s is wrong size\n', m_fields{i});
0026 end
0027 
0028 for i = 1 : length(n_fields)
0029     assert(length(tiger.(n_fields{i})) == n, ...
0030            'field %s is wrong size\n', n_fields{i});
0031 end
```

---

Generated on Thu 11-Aug-2011 15:06:22 by **m2html** © 2005
