# Supplementary material for: TIGER: Toolbox for integrating genome-scale metabolic models, expression data, and transcriptional regulatory networks
Source: BMC Syst Biol. 2011 Sep 23;5:147. doi: 10.1186/1752-0509-5-147 (PMC3224351; doi:10.1186/1752-0509-5-147)
Supplement: Additional file 2 — TIGER source code. Source code, documentation, and tutorials are also available online at http://bme.virginia.edu/csbl/downloads/ or http://csbl.bitbucket.org/tiger. [file 1752-0509-5-147-S2.GZ › tiger/doc/m2html/tiger/add_column.html]

Description of add\_column


Home > tiger > add\_column.m

# add\_column

## PURPOSE

**Add a column to a TIGER model structure**

## SYNOPSIS

**function [tiger,varname] = add\_column(tiger,name,vartype,lb,ub,obj,A)**

## DESCRIPTION

```
 ADD_COLUMN  Add a column to a TIGER model structure

   [TIGER,VARNAME] = ADD_COLUMN(TIGER,N)
   [TIGER,VARNAME] = ADD_COLUMN(TIGER,NAME,VARTYPE,LB,UB,OBJ,A)

   Add a column to an existing structure, updating the corresponding
   vectors.  The following default values are used:
       NAME     'VARi', where i is the column index
       VARTYPE  'b'
       LB       0
       UB       1
       OBJ      0
       A        column of zeros

   If called as ADD_COLUMN(TIGER,N), N columns are added with the default
   values.  More than one name can be given; if only a single value is
   given for the other arguments, it will be repeated for all new 
   columns.

   The return value VARNAME is the name(s) of the columns added.
```

## CROSS-REFERENCE INFORMATION

This function calls:

- check\_tiger Check size and orientation of TIGER fields
- array2names Create a cell of names from an array of numbers
- expand\_to Expand a vector to length N
- fill\_to Fill a short to empty vector

This function is called by:

- convert\_indicators Convert indicators to MILP constraints
- convert\_miqp Prepare a MIQP for solution
- add\_diff Add difference variables toa TIGER model
- cobra\_to\_elf Create an ELF model from a COBRA structure
- test\_\_add\_diff
- test\_\_diffadj
- test\_\_miqp
- test\_\_remove\_rule
- load\_rules
- diffadj Formulate and solve the differential adjustment problem
- imat Integrative Metabolic Analysis Tool

## SOURCE CODE

```
0001 function [tiger,varname] = add_column(tiger,name,vartype,lb,ub,obj,A)
0002 % ADD_COLUMN  Add a column to a TIGER model structure
0003 %
0004 %   [TIGER,VARNAME] = ADD_COLUMN(TIGER,N)
0005 %   [TIGER,VARNAME] = ADD_COLUMN(TIGER,NAME,VARTYPE,LB,UB,OBJ,A)
0006 %
0007 %   Add a column to an existing structure, updating the corresponding
0008 %   vectors.  The following default values are used:
0009 %       NAME     'VARi', where i is the column index
0010 %       VARTYPE  'b'
0011 %       LB       0
0012 %       UB       1
0013 %       OBJ      0
0014 %       A        column of zeros
0015 %
0016 %   If called as ADD_COLUMN(TIGER,N), N columns are added with the default
0017 %   values.  More than one name can be given; if only a single value is
0018 %   given for the other arguments, it will be repeated for all new
0019 %   columns.
0020 %
0021 %   The return value VARNAME is the name(s) of the columns added.
0022 
0023 [m,n] = size(tiger.A);
0024 loc = n+1;
0025 
0026 if nargin < 2 || isempty(name)
0027     name = {};
0028 elseif isa(name,'double')
0029     name = zeros(1,name);  % holder until filled after calculating N
0030 elseif isa(name,'char')
0031     name = {name};
0032 end
0033 
0034 if nargin < 3 || isempty(vartype), vartype = 'b'; end
0035 
0036 if nargin < 4 || isempty(lb), lb = 0; end
0037 
0038 if nargin < 5 || isempty(ub), ub = 1; end
0039 
0040 if nargin < 6 || isempty(obj), obj = 0; end
0041 
0042 if nargin < 7 || isempty(A), A = [];  end
0043 
0044 N = max([length(name), ...
0045          length(lb),   ...
0046          length(ub),   ...
0047          length(obj),  ...
0048          size(A,2),    ...
0049          length(vartype)]);
0050          
0051 if length(name) < N || isa(name,'double')
0052     % TODO:  add to names instead of replace
0053     name = array2names('VAR%i',loc:loc+N-1);
0054 end
0055 vartype = fill_to(vartype,N);
0056 lb = fill_to(lb,N);
0057 ub = fill_to(ub,N);
0058 obj = fill_to(obj,N);
0059 A = expand_to(A,[m N]);
0060 
0061 locs = loc : loc + N-1;
0062 
0063 tiger.varnames(locs) = name;
0064 tiger.vartypes(locs) = vartype;
0065 tiger.lb(locs) = lb;
0066 tiger.ub(locs) = ub;
0067 tiger.obj(locs) = obj;
0068 tiger.A(:,locs) = A;
0069 
0070 % expand the Q, Qd, and Qc fields
0071 if isfield(tiger,'Q') && ~isempty(tiger.Q)
0072     tiger.Q(n+N,n+N) = 0;
0073 end
0074 if isfield(tiger,'Qd') && ~isempty(tiger.Qd)
0075     tiger.Qd(n+N,n+N) = 0;
0076 end
0077 if isfield(tiger,'Qc') && ~isempty(tiger.Qc)
0078     tiger.Qc.w(n+N) = 0;
0079     tiger.Qc.c(n+N) = 0;
0080 end 
0081 
0082 tiger.param.fixedvar(end+(1:N)) = false;
0083 
0084 tiger = check_tiger(tiger);
0085 
0086 if nargout > 1
0087     varname = name;
0088 end
0089 
0090 
0091
```

---

Generated on Thu 11-Aug-2011 15:06:22 by **m2html** © 2005
