# Supplementary material for: TIGER: Toolbox for integrating genome-scale metabolic models, expression data, and transcriptional regulatory networks
Source: BMC Syst Biol. 2011 Sep 23;5:147. doi: 10.1186/1752-0509-5-147 (PMC3224351; doi:10.1186/1752-0509-5-147)
Supplement: Additional file 2 — TIGER source code. Source code, documentation, and tutorials are also available online at http://bme.virginia.edu/csbl/downloads/ or http://csbl.bitbucket.org/tiger. [file 1752-0509-5-147-S2.GZ › tiger/doc/m2html/tiger/set_fieldval.html]

Description of set\_fieldval


Home > tiger > set\_fieldval.m

# set\_fieldval

## PURPOSE

**Set values in a TIGER structure field**

## SYNOPSIS

**function [tiger] = set\_fieldval(tiger,field,id,val)**

## DESCRIPTION

```
 SET_FIELDVAL  Set values in a TIGER structure field

   [TIGER] = SET_FIELDVAL(TIGER,FIELD,ID,VAL)

   Set TIGER.(FIELD)(ID) = VAL.  Attempts to determine if IDS refer to 
   row or variable names based on FIELD, and raises an error if unable
   to do so.
```

## CROSS-REFERENCE INFORMATION

This function calls:

- convert\_ids Create name, indices, and logical indices from an array
- fill\_to Fill a short to empty vector

This function is called by:

- minimal\_genome Calculate a minimal genome
- set\_var Set bounds on a variable
- test\_\_add\_diff
- test\_\_multilevel
- test\_\_remove\_rule
- imat Integrative Metabolic Analysis Tool

## SOURCE CODE

```
0001 function [tiger] = set_fieldval(tiger,field,id,val)
0002 % SET_FIELDVAL  Set values in a TIGER structure field
0003 %
0004 %   [TIGER] = SET_FIELDVAL(TIGER,FIELD,ID,VAL)
0005 %
0006 %   Set TIGER.(FIELD)(ID) = VAL.  Attempts to determine if IDS refer to
0007 %   row or variable names based on FIELD, and raises an error if unable
0008 %   to do so.
0009 
0010 var_fields = {'obj','lb','ub','vartypes'};
0011 row_fields = {'ctypes','b','ind','indtypes'};
0012 
0013 if ismember(field,var_fields)
0014     [~,idxs] = convert_ids(tiger.varnames,id);
0015 elseif ismember(field,row_fields)
0016     [~,idxs] = convert_ids(tiger.rownames,id);
0017 else
0018     error('field not found');
0019 end
0020 
0021 vals = fill_to(val,length(idxs));
0022 
0023 tiger.(field)(idxs) = vals;
```

---

Generated on Thu 11-Aug-2011 15:06:22 by **m2html** © 2005
