# Supplementary material for: TIGER: Toolbox for integrating genome-scale metabolic models, expression data, and transcriptional regulatory networks
Source: BMC Syst Biol. 2011 Sep 23;5:147. doi: 10.1186/1752-0509-5-147 (PMC3224351; doi:10.1186/1752-0509-5-147)
Supplement: Additional file 2 — TIGER source code. Source code, documentation, and tutorials are also available online at http://bme.virginia.edu/csbl/downloads/ or http://csbl.bitbucket.org/tiger. [file 1752-0509-5-147-S2.GZ › tiger/doc/m2html/tiger/close_bounds.html]

Description of close\_bounds


Home > tiger > close\_bounds.m

# close\_bounds

## PURPOSE

**Return bounds that have been opened by OPEN\_BOUNDS**

## SYNOPSIS

**function [tiger] = close\_bounds(tiger,bounds)**

## DESCRIPTION

```
 CLOSE_BOUNDS  Return bounds that have been opened by OPEN_BOUNDS

   [TIGER] = CLOSE_BOUNDS(TIGER,BOUNDS)

   Resets a model that has been opened by OPEN_BOUNDS to the original
   bounds.  BOUNDS is the bounds structure returned by OPEN_BOUNDS.
   Returns the modified model.
```

## CROSS-REFERENCE INFORMATION

This function calls:


This function is called by:

- create\_yeast\_trn\_model
- run\_new\_rules

## SOURCE CODE

```
0001 function [tiger] = close_bounds(tiger,bounds)
0002 % CLOSE_BOUNDS  Return bounds that have been opened by OPEN_BOUNDS
0003 %
0004 %   [TIGER] = CLOSE_BOUNDS(TIGER,BOUNDS)
0005 %
0006 %   Resets a model that has been opened by OPEN_BOUNDS to the original
0007 %   bounds.  BOUNDS is the bounds structure returned by OPEN_BOUNDS.
0008 %   Returns the modified model.
0009 
0010 tiger.lb(1:bounds.N) = bounds.lb;
0011 tiger.ub(1:bounds.N) = bounds.ub;
```

---

Generated on Thu 11-Aug-2011 15:06:22 by **m2html** © 2005
