# Supplementary material for: TIGER: Toolbox for integrating genome-scale metabolic models, expression data, and transcriptional regulatory networks
Source: BMC Syst Biol. 2011 Sep 23;5:147. doi: 10.1186/1752-0509-5-147 (PMC3224351; doi:10.1186/1752-0509-5-147)
Supplement: Additional file 2 — TIGER source code. Source code, documentation, and tutorials are also available online at http://bme.virginia.edu/csbl/downloads/ or http://csbl.bitbucket.org/tiger. [file 1752-0509-5-147-S2.GZ › tiger/doc/m2html/tiger/parsing/parse.html]

Description of parse


Home > tiger > parsing > parse.m

# parse

## PURPOSE

**Parse a sequence of TOKEN objects**

## SYNOPSIS

**function [expression] = parse(tokens,levels,unary)**

## DESCRIPTION

```
 PARSE  Parse a sequence of TOKEN objects

   [EXPRESSION] = PARSE(TOKENS,LEVLES,UNARY)

   Parse a cell of TOKEN objects (TOKENS) to create an EXPR object.

   LEVELS is a cell specifying the operator precedence.  Each entry is a
   cell containing strings identifying an operator at the current level.
   The LEVELS{1} contains the operators of highest precedence.

   UNARY is a cell of operators that should be treated as unary and
   prefix.  All other operators are assumed to be binary infix.
```

## CROSS-REFERENCE INFORMATION

This function calls:

- make\_expr Make an EXPR cons object from tokens

This function is called by:

- parse\_string Parse a rule string into an EXPR object

## SUBFUNCTIONS

- function [ex] = parse\_aux()
- function [level] = get\_level(op)
- function [e] = get\_expr(level)

## SOURCE CODE

```
0001 function [expression] = parse(tokens,levels,unary)
0002 % PARSE  Parse a sequence of TOKEN objects
0003 %
0004 %   [EXPRESSION] = PARSE(TOKENS,LEVLES,UNARY)
0005 %
0006 %   Parse a cell of TOKEN objects (TOKENS) to create an EXPR object.
0007 %
0008 %   LEVELS is a cell specifying the operator precedence.  Each entry is a
0009 %   cell containing strings identifying an operator at the current level.
0010 %   The LEVELS{1} contains the operators of highest precedence.
0011 %
0012 %   UNARY is a cell of operators that should be treated as unary and
0013 %   prefix.  All other operators are assumed to be binary infix.
0014 
0015 expression = parse_aux();
0016 
0017 function [ex] = parse_aux()
0018     ex = get_expr(0);
0019     while tokens.is_another
0020         next = tokens.pop();
0021         if next.is_rparen
0022             return
0023         end
0024         op = next.value;
0025         assert(next.is_op,'Operator expected instead of ''%s''',op);
0026         ex = make_expr(ex,op,get_expr(get_level(op)));
0027     end
0028     if isa(ex,'token')
0029         ex = make_expr(ex);
0030     end
0031 end
0032 
0033 function [level] = get_level(op)
0034     found = cellfun(@(x) ismember(op,x),levels);
0035     level = find(found);
0036 end
0037 
0038 function [e] = get_expr(level)
0039     t = tokens.pop();
0040     if t.is_lparen
0041         e = parse_aux();
0042     elseif t.is_op
0043         op = t.value;
0044         assert(ismember(op,unary),'Unexpected operator ''%s''',op);
0045         e = make_expr(get_expr(get_level(op)));
0046         e.negated = true;
0047     else
0048         e = t;
0049     end
0050     if tokens.is_another
0051         next = tokens.peek();
0052         if next.is_op && get_level(next.value) < level
0053             tokens.pop();
0054             e = make_expr(e,next.value,get_expr(get_level(next.value)));
0055         end
0056     end
0057 end
0058 
0059 end % function
```

---

Generated on Thu 11-Aug-2011 15:06:22 by **m2html** © 2005
