# Supplementary material for: TIGER: Toolbox for integrating genome-scale metabolic models, expression data, and transcriptional regulatory networks
Source: BMC Syst Biol. 2011 Sep 23;5:147. doi: 10.1186/1752-0509-5-147 (PMC3224351; doi:10.1186/1752-0509-5-147)
Supplement: Additional file 2 — TIGER source code. Source code, documentation, and tutorials are also available online at http://bme.virginia.edu/csbl/downloads/ or http://csbl.bitbucket.org/tiger. [file 1752-0509-5-147-S2.GZ › tiger/doc/m2html/tiger/parsing/index.html]

Index for Directory tiger/parsing


|  |  |
| --- | --- |
| Master index | Index for tiger/parsing |

# Index for tiger/parsing

## Matlab files in this directory:

|  |  |
| --- | --- |
| expr |  |
| is\_valid\_expr | Check that an EXPR object is semantically valid |
| is\_valid\_rule | Check that an EXPR object is a valid rule |
| lex | Return a list of TOKENS from a string |
| make\_dnf | Make lists of atoms in the Disjunctive Normal Form |
| make\_expr | Make an EXPR cons object from tokens |
| parse | Parse a sequence of TOKEN objects |
| parse\_string | Parse a rule string into an EXPR object |
| token |  |

---

Generated on Thu 11-Aug-2011 15:06:20 by **m2html** © 2005
