# Supplementary material for: TIGER: Toolbox for integrating genome-scale metabolic models, expression data, and transcriptional regulatory networks
Source: BMC Syst Biol. 2011 Sep 23;5:147. doi: 10.1186/1752-0509-5-147 (PMC3224351; doi:10.1186/1752-0509-5-147)
Supplement: Additional file 2 — TIGER source code. Source code, documentation, and tutorials are also available online at http://bme.virginia.edu/csbl/downloads/ or http://csbl.bitbucket.org/tiger. [file 1752-0509-5-147-S2.GZ › tiger/doc/m2html/tiger/parsing/is_valid_rule.html]

Description of is\_valid\_rule


Home > tiger > parsing > is\_valid\_rule.m

# is\_valid\_rule

## PURPOSE

**Check that an EXPR object is a valid rule**

## SYNOPSIS

**function [tf] = is\_valid\_rule(rule)**

## DESCRIPTION

```
 IS_VALID_RULE  Check that an EXPR object is a valid rule
```

## CROSS-REFERENCE INFORMATION

This function calls:

- is\_valid\_expr Check that an EXPR object is semantically valid
- is\_valid\_rule Check that an EXPR object is a valid rule

This function is called by:

- is\_valid\_rule Check that an EXPR object is a valid rule

## SOURCE CODE

```
0001 function [tf] = is_valid_rule(rule)
0002 % IS_VALID_RULE  Check that an EXPR object is a valid rule
0003 
0004 if isa(rule,'cell')
0005     tf = cellfun(@is_valid_rule,rule);
0006 else
0007     tf =    (rule.is_rule) ...
0008          && is_valid_expr(rule.lexpr) ...
0009          && is_valid_expr(rule.rexpr) ...
0010          && ~rule.negated;
0011 end
0012
```

---

Generated on Thu 11-Aug-2011 15:06:22 by **m2html** © 2005
