# Supplementary material for: TIGER: Toolbox for integrating genome-scale metabolic models, expression data, and transcriptional regulatory networks
Source: BMC Syst Biol. 2011 Sep 23;5:147. doi: 10.1186/1752-0509-5-147 (PMC3224351; doi:10.1186/1752-0509-5-147)
Supplement: Additional file 2 — TIGER source code. Source code, documentation, and tutorials are also available online at http://bme.virginia.edu/csbl/downloads/ or http://csbl.bitbucket.org/tiger. [file 1752-0509-5-147-S2.GZ › tiger/doc/m2html/tiger/parsing/token.html]

Description of token


Home > tiger > parsing > token.m

# token

## PURPOSE

## SYNOPSIS

**This is a script file.**

## DESCRIPTION

## CROSS-REFERENCE INFORMATION

This function calls:

- token

This function is called by:

- lex Return a list of TOKENS from a string
- token

## SUBFUNCTIONS

- function [obj] = token(str,op,lparen,rparen)
- function display(obj)

## SOURCE CODE

```
0001 classdef token
0002 % TOKEN  Parse token.
0003 %
0004 %   TOKEN is a lexical token.  A list of TOKEN objects is generated
0005 %   by the lexer (LEX).
0006     
0007 properties
0008     is_op = false      % true if the token is a valid operator
0009     is_lparen = false  % true if the token is a left parenthesis '('
0010     is_rparen = false  % true if the token is a right parenthesis ')'
0011     quoted = false     % true if the value was originally quoted
0012     
0013     value   % token string
0014 end
0015 
0016 methods
0017     function [obj] = token(str,op,lparen,rparen)
0018         % TOKEN  Create a token from a string.
0019         %
0020         %   [OBJ] = TOKEN(STR,OP,LPAREN,RPAREN)
0021         %
0022         %   Create a token with value STR.  Optional arguments are:
0023         %       OP      true if token is an operator
0024         %       LPAREN  true if token is a left parenthesis
0025         %       RPAREN  true if token is a right parenthesis
0026         
0027         obj.value = str;
0028         
0029         if nargin >= 2 && op
0030             obj.is_op = true;
0031         end
0032         if nargin >= 3 && lparen
0033             obj.is_lparen = true;
0034         end
0035         if nargin >= 4 && rparen
0036             obj.is_rparen = true;
0037         end
0038     end
0039     
0040     function display(obj)
0041         % DISPLAY  Show the value of a token to the toplevel.
0042         indent = '   ';
0043         if obj.is_lparen
0044             disp([indent 'LPAREN']);
0045         elseif obj.is_rparen
0046             disp([indent 'RPAREN']);
0047         elseif obj.is_op
0048             disp([indent 'op: ' obj.value]);
0049         elseif obj.quoted
0050             disp([indent '"' obj.value '"']);
0051         else
0052             disp([indent '''' obj.value '''']);
0053         end
0054     end
0055 end
0056 
0057 end % classdef
0058
```

---

Generated on Thu 11-Aug-2011 15:06:22 by **m2html** © 2005
