# Supplementary material for: TIGER: Toolbox for integrating genome-scale metabolic models, expression data, and transcriptional regulatory networks
Source: BMC Syst Biol. 2011 Sep 23;5:147. doi: 10.1186/1752-0509-5-147 (PMC3224351; doi:10.1186/1752-0509-5-147)
Supplement: Additional file 2 — TIGER source code. Source code, documentation, and tutorials are also available online at http://bme.virginia.edu/csbl/downloads/ or http://csbl.bitbucket.org/tiger. [file 1752-0509-5-147-S2.GZ › tiger/doc/m2html/tiger/parsing/lex.html]

Description of lex


Home > tiger > parsing > lex.m

# lex

## PURPOSE

**Return a list of TOKENS from a string**

## SYNOPSIS

**function [tokens] = lex(str)**

## DESCRIPTION

```
 LEX  Return a list of TOKENS from a string
```

## CROSS-REFERENCE INFORMATION

This function calls:

- token
- stack
- strbuffer

This function is called by:

- parse\_string Parse a rule string into an EXPR object

## SUBFUNCTIONS

- function [tf] = is\_sub\_op(str)
- function [tok] = make\_token(str)
- function [tf] = is\_op(str)

## SOURCE CODE

```
0001 function [tokens] = lex(str)
0002 % LEX  Return a list of TOKENS from a string
0003 
0004 op_subs = { 'and', {'and','AND','&&','&'}, ...
0005             'or' , {'or','OR','||','|'}, ...
0006             'iff', {'iff','Iff','IFF','<=>','<==>','<->','<-->'}, ...
0007             'if' , {'if','If','IF','=>','==>','->','-->'}, ...
0008             '>=' , {'>='}, ...
0009             '>'  , {'>'}, ...
0010             '='  , {'=','=='}, ...
0011             '<'  , {'<'}, ...
0012             '<=' , {'<='}, ...
0013             '~=' , {'~=','!=','<>'}, ...
0014             'not', {'not','NOT','Not','~','!'} };
0015 
0016 alphas = 'ABCDEFGHIJKLMNOPQRSTUVWXYZabcdefghijklmnopqrstuvwxyz';
0017 all_ops = [op_subs{2:2:end}];
0018 opstart = setdiff(cellfun(@(x) {x(1)},all_ops), ...
0019                   arrayfun(@(x) {x}, alphas));
0020 op_keys = op_subs(1:2:end);
0021 op_vals = op_subs(2:2:end);    
0022 
0023 whitespace = {' ','\t'};
0024 
0025 chars = stack(fliplr(str));
0026 tokens = stack();
0027 
0028 
0029 buf = strbuffer();
0030 in_quote = false;
0031 quote = '';
0032 while chars.is_another
0033     ch = chars.pop();
0034     if in_quote
0035         if ch == '\'
0036             buf.append(chars.pop());
0037         else
0038             buf.append(ch);
0039         end
0040         
0041         if ch == quote
0042             tokens.push(make_token());
0043             buf.clear;
0044             in_quote = false;
0045             quote = '';
0046         end
0047         continue;
0048     end
0049     switch ch
0050         case {'(',')'}
0051             if ~isempty(buf)
0052                 tokens.push(make_token());
0053                 buf.clear;
0054             end
0055             tokens.push(make_token(ch));
0056         case whitespace
0057             if ~isempty(buf)
0058                 tokens.push(make_token());
0059                 buf.clear;
0060             end
0061         case opstart
0062             punc = strbuffer(ch);
0063             while chars.is_another && is_sub_op([punc.val chars.peek()])
0064                 punc.append(chars.pop());
0065             end
0066             % punc may contains a complete operator
0067             if is_op(punc.val)
0068                 if ~isempty(buf)
0069                     tokens.push(make_token());
0070                     buf.clear;
0071                 end
0072                 tokens.push(make_token(punc.val));
0073             else
0074                 % this was not a complete operator
0075                 buf.append(punc.val);
0076             end
0077         case {'''','"'}
0078             buf.append(ch);
0079             in_quote = true;
0080             quote = ch;
0081         otherwise
0082             buf.append(ch);
0083     end
0084 end
0085 
0086 if ~isempty(buf)
0087     tokens.push(make_token());
0088 end
0089 
0090 tokens.reverse();
0091 
0092 function [tf] = is_sub_op(str)
0093     like = @(x,s) length(x) >= length(s) && strcmp(x(1:length(s)),s);
0094     tf = any(cellfun(@(x) like(x,str),all_ops));
0095 end
0096 
0097 function [tok] = make_token(str)
0098     if nargin == 0
0099         str = buf.val;
0100     end
0101     
0102     quoted = false;
0103     
0104     if is_op(str)
0105         str = op_keys{cellfun(@(x) ismember(str,x),op_vals)};
0106         tok = token(str,true);
0107     elseif str == '('
0108         tok = token(str,false,true);
0109     elseif str == ')'
0110         tok = token(str,false,false,true);
0111     else
0112         if str(1) == '''' || str(1) == '"'
0113             % strip the quotes
0114             str = str(2:end-1);
0115             quoted = true;
0116         end
0117         tok = token(str);
0118     end
0119     
0120     tok.quoted = quoted;
0121 end
0122 
0123 function [tf] = is_op(str)
0124     if nargin == 0
0125         str = buf.val;
0126     end
0127     tf = ismember(str,all_ops);
0128 end
0129 
0130 end % function
```

---

Generated on Thu 11-Aug-2011 15:06:22 by **m2html** © 2005
