# Supplementary material for: TIGER: Toolbox for integrating genome-scale metabolic models, expression data, and transcriptional regulatory networks
Source: BMC Syst Biol. 2011 Sep 23;5:147. doi: 10.1186/1752-0509-5-147 (PMC3224351; doi:10.1186/1752-0509-5-147)
Supplement: Additional file 2 — TIGER source code. Source code, documentation, and tutorials are also available online at http://bme.virginia.edu/csbl/downloads/ or http://csbl.bitbucket.org/tiger. [file 1752-0509-5-147-S2.GZ › tiger/doc/m2html/tiger/parsing/make_rxnGeneMat.html]

Description of make\_rxnGeneMat


Home > tiger > parsing > make\_rxnGeneMat.m

# make\_rxnGeneMat

## PURPOSE

**Build a rxnGeneMat for cobra models**

## SYNOPSIS

**function [rgm] = make\_rxnGeneMat(cobra,exprs)**

## DESCRIPTION

```
 MAKE_RXNGENEMAT  Build a rxnGeneMat for cobra models

   [RGM] = MAKE_RXNGENEMAT(COBRA,EXPRS)

   Create a reaction-gene matrix (rxnGeneMat) for a COBRA toolbox model.
   The (i,j)th entry indicates whether the ith gene appears is the GPR
   for the jth reaction.

   Inputs
   COBRA   A COBRA toolbox model
   EXPRS   (optional) A set of expr objects parsed from the GPR.  This
           avoids the need to parse the GPR again.

   Outputs
   RGM     The rxnGeneMat in the form of the COBRA toolbox field of the
           same name.
```

## CROSS-REFERENCE INFORMATION

This function calls:

- parse\_string Parse a rule string into an EXPR object

This function is called by:

- cobra\_model 2 3 4 5 6 7 8 9

## SOURCE CODE

```
0001 function [rgm] = make_rxnGeneMat(cobra,exprs)
0002 % MAKE_RXNGENEMAT  Build a rxnGeneMat for cobra models
0003 %
0004 %   [RGM] = MAKE_RXNGENEMAT(COBRA,EXPRS)
0005 %
0006 %   Create a reaction-gene matrix (rxnGeneMat) for a COBRA toolbox model.
0007 %   The (i,j)th entry indicates whether the ith gene appears is the GPR
0008 %   for the jth reaction.
0009 %
0010 %   Inputs
0011 %   COBRA   A COBRA toolbox model
0012 %   EXPRS   (optional) A set of expr objects parsed from the GPR.  This
0013 %           avoids the need to parse the GPR again.
0014 %
0015 %   Outputs
0016 %   RGM     The rxnGeneMat in the form of the COBRA toolbox field of the
0017 %           same name.
0018 
0019 nrxns = size(cobra.S,2);
0020 ngenes = length(cobra.genes);
0021 
0022 has_gpr = cellfun(@(x) ~isempty(x), cobra.grRules);
0023 
0024 if nargin < 2
0025     exprs = cell(1,nrxns);
0026     for i = 1 : nrxns
0027         if has_gpr(i)
0028             exprs{i} = parse_string(cobra.grRules{i});
0029         else
0030             exprs{i} = [];
0031         end
0032     end
0033 end
0034 
0035 rgm = false(nrxns,ngenes);
0036 for i = 1 : nrxns
0037     if has_gpr(i)
0038         rgm(i,:) = ismember(cobra.genes, ...
0039                             exprs{i}.atoms);
0040     end
0041 end
0042
```

---

Generated on Wed 27-Apr-2011 15:36:13 by **m2html** © 2005
