# Supplementary material for: TIGER: Toolbox for integrating genome-scale metabolic models, expression data, and transcriptional regulatory networks
Source: BMC Syst Biol. 2011 Sep 23;5:147. doi: 10.1186/1752-0509-5-147 (PMC3224351; doi:10.1186/1752-0509-5-147)
Supplement: Additional file 2 — TIGER source code. Source code, documentation, and tutorials are also available online at http://bme.virginia.edu/csbl/downloads/ or http://csbl.bitbucket.org/tiger. [file 1752-0509-5-147-S2.GZ › tiger/doc/m2html/tiger/parsing/make_expr.html]

Description of make\_expr


Home > tiger > parsing > make\_expr.m

# make\_expr

## PURPOSE

**Make an EXPR cons object from tokens**

## SYNOPSIS

**function [ex] = make\_expr(e1,op,e2)**

## DESCRIPTION

```
 MAKE_EXPR  Make an EXPR cons object from tokens

   [EX] = MAKE_EXPR(E1)
   [EX] = MAKE_EXPR(E1,OP,E2)

   If one object (E1) is given, an atom is created and returned.  If E1
   is already an expression, it is returned unchanged.

   If two tokens (E1 and E2) are given, they are cons-ed with the
   operator string OP; the resulting compound expression is returned.
```

## CROSS-REFERENCE INFORMATION

This function calls:

- expr
- make\_expr Make an EXPR cons object from tokens

This function is called by:

- make\_expr Make an EXPR cons object from tokens
- parse Parse a sequence of TOKEN objects

## SOURCE CODE

```
0001 function [ex] = make_expr(e1,op,e2)
0002 % MAKE_EXPR  Make an EXPR cons object from tokens
0003 %
0004 %   [EX] = MAKE_EXPR(E1)
0005 %   [EX] = MAKE_EXPR(E1,OP,E2)
0006 %
0007 %   If one object (E1) is given, an atom is created and returned.  If E1
0008 %   is already an expression, it is returned unchanged.
0009 %
0010 %   If two tokens (E1 and E2) are given, they are cons-ed with the
0011 %   operator string OP; the resulting compound expression is returned.
0012 
0013 if nargin == 1
0014     if isa(e1,'token')
0015         ex = expr();
0016         ex.id = e1.value;
0017         ex.was_quoted = e1.quoted;
0018     elseif isa(e1,'expr')
0019         ex = e1;
0020     else
0021         error('Expr cannot be formed from class ''%s''',class(e1));
0022     end
0023 else
0024     % make operator cons
0025     ex = expr();
0026     ex.lexpr = make_expr(e1);
0027     ex.rexpr = make_expr(e2);
0028     switch op
0029         case 'and'
0030             ex.AND = true;
0031         case 'or'
0032             ex.OR = true;
0033         case 'iff'
0034             ex.IFF = true;
0035         case 'if'
0036             ex.IF = true;
0037         otherwise
0038             ex.cond_op = op;
0039     end
0040 end
```

---

Generated on Thu 11-Aug-2011 15:06:22 by **m2html** © 2005
