# Supplementary material for: TIGER: Toolbox for integrating genome-scale metabolic models, expression data, and transcriptional regulatory networks
Source: BMC Syst Biol. 2011 Sep 23;5:147. doi: 10.1186/1752-0509-5-147 (PMC3224351; doi:10.1186/1752-0509-5-147)
Supplement: Additional file 2 — TIGER source code. Source code, documentation, and tutorials are also available online at http://bme.virginia.edu/csbl/downloads/ or http://csbl.bitbucket.org/tiger. [file 1752-0509-5-147-S2.GZ › tiger/doc/m2html/tiger/parsing/make_dnf.html]

Description of make\_dnf


Home > tiger > parsing > make\_dnf.m

# make\_dnf

## PURPOSE

**Make lists of atoms in the Disjunctive Normal Form**

## SYNOPSIS

**function [and\_lists] = make\_dnf(ex,names\_only)**

## DESCRIPTION

```
 MAKE_DNF  Make lists of atoms in the Disjunctive Normal Form

   [AND_LISTS] = MAKE_DNF(EX)
   [AND_LISTS] = MAKE_DNF(EX,NAMES_ONLY)

   Splits an expression EX into a series of ATOM lists.  Each list
   corresponds to a disjunction in the form
       EX <=> (list1) or (list2) or (list3) or ...
   where
       list1 <=> AND_LISTS{1}{1} & AND_LISTS{1}{2} & ...

   AND_LISTS is a cell array of ATOM cells.  If NAMES_ONLY is true
   (default = false), then only the names of the atoms are returned, not
   the entire ATOM object.
```

## CROSS-REFERENCE INFORMATION

This function calls:

- map Generate a new list by applying a function

This function is called by:

- cobra\_to\_elf Create an ELF model from a COBRA structure

## SUBFUNCTIONS

- function [tf] = has\_or(ex)
- function [sub\_ex] = split\_by\_or(ex,dir)
- function [atoms] = get\_atoms(ex)

## SOURCE CODE

```
0001 function [and_lists] = make_dnf(ex,names_only)
0002 % MAKE_DNF  Make lists of atoms in the Disjunctive Normal Form
0003 %
0004 %   [AND_LISTS] = MAKE_DNF(EX)
0005 %   [AND_LISTS] = MAKE_DNF(EX,NAMES_ONLY)
0006 %
0007 %   Splits an expression EX into a series of ATOM lists.  Each list
0008 %   corresponds to a disjunction in the form
0009 %       EX <=> (list1) or (list2) or (list3) or ...
0010 %   where
0011 %       list1 <=> AND_LISTS{1}{1} & AND_LISTS{1}{2} & ...
0012 %
0013 %   AND_LISTS is a cell array of ATOM cells.  If NAMES_ONLY is true
0014 %   (default = false), then only the names of the atoms are returned, not
0015 %   the entire ATOM object.
0016 
0017 if nargin < 2
0018     names_only = false;
0019 end
0020 
0021 ex_list = {ex.copy};
0022 
0023 while any(cellfun(@has_or,ex_list))
0024     for i = 1 : length(ex_list)
0025         if has_or(ex_list{i})
0026             ex_list{end+1} = split_by_or(ex_list{i},'right');
0027             ex_list{i}     = split_by_or(ex_list{i},'left');
0028         end
0029     end
0030 end
0031 
0032 and_lists = map(@get_atoms,ex_list);
0033 
0034 if names_only
0035     and_lists = map(@(x) map(@(e) e.id,x),and_lists);
0036 end
0037 
0038 
0039 function [tf] = has_or(ex)
0040     tf = ex.is_junc ...
0041             && (ex.OR || has_or(ex.lexpr) || has_or(ex.rexpr));
0042         
0043 function [sub_ex] = split_by_or(ex,dir)
0044     if ~has_or(ex)
0045         sub_ex = ex.copy;
0046     elseif ex.OR
0047         switch dir
0048             case 'left'
0049                 sub_ex = ex.lexpr.copy;
0050             case 'right'
0051                 sub_ex = ex.rexpr.copy;
0052         end
0053     else % AND
0054         sub_ex = ex.copy;
0055         if has_or(ex.lexpr)
0056             sub_ex.lexpr = split_by_or(ex.lexpr,dir);
0057         else
0058             sub_ex.rexpr = split_by_or(ex.rexpr,dir);
0059         end
0060     end
0061 
0062 function [atoms] = get_atoms(ex)
0063     if ~ex.is_junc
0064         atoms = {ex.copy};
0065     else
0066         atoms = [get_atoms(ex.lexpr), get_atoms(ex.rexpr)];
0067     end
0068
```

---

Generated on Thu 11-Aug-2011 15:06:22 by **m2html** © 2005
