# Supplementary material for: TIGER: Toolbox for integrating genome-scale metabolic models, expression data, and transcriptional regulatory networks
Source: BMC Syst Biol. 2011 Sep 23;5:147. doi: 10.1186/1752-0509-5-147 (PMC3224351; doi:10.1186/1752-0509-5-147)
Supplement: Additional file 2 — TIGER source code. Source code, documentation, and tutorials are also available online at http://bme.virginia.edu/csbl/downloads/ or http://csbl.bitbucket.org/tiger. [file 1752-0509-5-147-S2.GZ › tiger/doc/m2html/tiger/parsing/convert_grRules.html]

Description of convert\_grRules


Home > tiger > parsing > convert\_grRules.m

# convert\_grRules

## PURPOSE

## SYNOPSIS

**function [rules] = convert\_grRules(cobra)**

## DESCRIPTION

## CROSS-REFERENCE INFORMATION

This function calls:

- parse\_string Parse a rule string into an EXPR object
- map

This function is called by:

- cobra\_model 2 3 4 5 6 7 8 9

## SUBFUNCTIONS

- function [rule] = convert\_aux(str)
- function switch\_atom(e)

## SOURCE CODE

```
0001 function [rules] = convert_grRules(cobra)
0002 
0003 rules = map(@convert_aux,cobra.grRules);
0004 
0005 function [rule] = convert_aux(str)
0006     e = parse_string(str);
0007     e.iterif(@(x) x.is_atom,@(x) switch_atom(x));
0008     rule = e.to_string();
0009 end
0010 
0011 function switch_atom(e)
0012     [~,loc] = ismember(e.id,cobra.genes);
0013     e.id = sprintf('x(%i)',loc);
0014 end
0015 
0016 end
```

---

Generated on Wed 27-Apr-2011 15:36:13 by **m2html** © 2005
