# Supplementary material for: TIGER: Toolbox for integrating genome-scale metabolic models, expression data, and transcriptional regulatory networks
Source: BMC Syst Biol. 2011 Sep 23;5:147. doi: 10.1186/1752-0509-5-147 (PMC3224351; doi:10.1186/1752-0509-5-147)
Supplement: Additional file 2 — TIGER source code. Source code, documentation, and tutorials are also available online at http://bme.virginia.edu/csbl/downloads/ or http://csbl.bitbucket.org/tiger. [file 1752-0509-5-147-S2.GZ › tiger/doc/m2html/tiger/parsing/parse_string.html]

Description of parse\_string


Home > tiger > parsing > parse\_string.m

# parse\_string

## PURPOSE

**Parse a rule string into an EXPR object**

## SYNOPSIS

**function [exp] = parse\_string(str,numeric)**

## DESCRIPTION

```
 PARSE_STRING  Parse a rule string into an EXPR object

   [EXP] = PARSE_STRING(STR)
   [EXP] = PARSE_STRING(STR,NUMERIC)

   Parse a string and return the corresponding EXPR object.  If STR is a
   cell array of strings, EXP will be a cell array of EXPR objects.

   If any element of STR is already an EXPR object, a copy of the object 
   is returned.

   The NUMERIC option determines if conditional statements can contain
   numeric constants.  If true (the default), atoms in conditionals are
   parsed as constants.  If false, they are treated as variable names.
```

## CROSS-REFERENCE INFORMATION

This function calls:

- expr
- lex Return a list of TOKENS from a string
- parse Parse a sequence of TOKEN objects
- assert\_cell Assert that variable is a cell array.
- map Generate a new list by applying a function

This function is called by:

- add\_rule Add rules to a TIGER model
- convert\_grRules Parse grRules into rules for the COBRA toolbox
- make\_rxnGeneMat Build a rxnGeneMat for cobra models
- cobra\_to\_elf Create an ELF model from a COBRA structure
- find\_infeasible\_rules Determine which rules make a model infeasible.
- remove\_rule Remove rule(s) previously added to a TIGER model
- convert\_rules
- create\_yeast\_trn\_model
- load\_rules

## SUBFUNCTIONS

- function [e] = parse\_single(s)
- function parse\_numerics(cond)
- function parse\_aux(e)

## SOURCE CODE

```
0001 function [exp] = parse_string(str,numeric)
0002 % PARSE_STRING  Parse a rule string into an EXPR object
0003 %
0004 %   [EXP] = PARSE_STRING(STR)
0005 %   [EXP] = PARSE_STRING(STR,NUMERIC)
0006 %
0007 %   Parse a string and return the corresponding EXPR object.  If STR is a
0008 %   cell array of strings, EXP will be a cell array of EXPR objects.
0009 %
0010 %   If any element of STR is already an EXPR object, a copy of the object
0011 %   is returned.
0012 %
0013 %   The NUMERIC option determines if conditional statements can contain
0014 %   numeric constants.  If true (the default), atoms in conditionals are
0015 %   parsed as constants.  If false, they are treated as variable names.
0016 
0017 if nargin < 2
0018     numeric = true;
0019 end
0020 
0021 % default regular expression for numeric values
0022 NUMERIC_REGEXP = '^[+-]?\d+\.?\d*([eE]+[+-]?\d+)?$';
0023 
0024 levels = { {'not'}, ...
0025            {'>=','>','=','<','<=','~='}, ...
0026            {'and','or'}, ...
0027            {'iff','if'} };
0028 
0029 unary = {'not'};
0030 
0031 strs = assert_cell(str);
0032 exp = map(@parse_single,strs);
0033 if numeric
0034     cellfun(@(x) x.iterif(@(e) e.is_cond,@parse_numerics),exp);
0035 end
0036 
0037 if ~isa(str,'cell') && ~isempty(exp)
0038     exp = exp{1};
0039 end
0040 
0041 function [e] = parse_single(s)
0042     if isa(s,'expr')
0043         e = s.copy();
0044     elseif isempty(s)
0045         e = expr();
0046         e.NULL = true;
0047     else
0048         assert(isa(s,'char'),'Objects of class %s cannot be parsed.', ...
0049                              class(s));
0050         e = parse(lex(s),levels,unary);
0051     end
0052 end
0053 
0054 function parse_numerics(cond)
0055     parse_aux(cond.lexpr);
0056     parse_aux(cond.rexpr);
0057 
0058     function parse_aux(e)
0059         if ~e.was_quoted
0060             m = regexp(e.id,NUMERIC_REGEXP,'once');
0061             if ~isempty(m)
0062                 e.is_numeric = true;
0063             end
0064         end
0065     end
0066 end
0067 
0068 end
```

---

Generated on Thu 11-Aug-2011 15:06:22 by **m2html** © 2005
