# Supplementary material for: TIGER: Toolbox for integrating genome-scale metabolic models, expression data, and transcriptional regulatory networks
Source: BMC Syst Biol. 2011 Sep 23;5:147. doi: 10.1186/1752-0509-5-147 (PMC3224351; doi:10.1186/1752-0509-5-147)
Supplement: Additional file 2 — TIGER source code. Source code, documentation, and tutorials are also available online at http://bme.virginia.edu/csbl/downloads/ or http://csbl.bitbucket.org/tiger. [file 1752-0509-5-147-S2.GZ › tiger/doc/m2html/tiger/parsing/expr.html]

Description of expr


Home > tiger > parsing > expr.m

# expr

## PURPOSE

## SYNOPSIS

**This is a script file.**

## DESCRIPTION

## CROSS-REFERENCE INFORMATION

This function calls:

- expr
- textframe

This function is called by:

- add\_rule Add rules to a TIGER model
- find\_infeasible\_rules Determine which rules make a model infeasible.
- expr
- make\_expr Make an EXPR cons object from tokens
- parse\_string Parse a rule string into an EXPR object
- remove\_rule Remove rule(s) previously added to a TIGER model

## SUBFUNCTIONS

- function [tf] = get.is\_junc(obj)
- function [tf] = get.is\_cond(obj)
- function [tf] = get.is\_rule(obj)
- function [tf] = get.is\_atom(obj)
- function [atoms] = get.atoms(obj)
- function [tf] = get.is\_simple(obj)
- function [new] = copy(obj)
- function iterif(e,test,f)
- function [new\_e] = mapif(e,test,f)
- function iter(e,f)
- function [new\_e] = map(e,f)
- function demorgan(obj)
- function [str] = get.display\_id(obj)
- function [str] = to\_string(obj)
- function [s] = make\_cons(sep)
- function [str] = cond\_to\_str(obj)
- function [frame] = make\_textframe(obj,indent,pipe)
- function display(obj)

## SOURCE CODE

```
0001 classdef expr < handle
0002 % EXPR  Expression objects for describing TIGER rules.
0003     
0004 properties (Dependent)
0005     is_junc     % true if EXPR is a junction (AND or OR)
0006     is_cond     % true if EXPR is a conditional (<=, <, etc.)
0007     is_rule     % true if EXPR is a rule (=> or <=>)
0008     is_atom     % true if EXPR is an atom (variable name)
0009     
0010     is_simple   % is a simple rule; does not require substitution
0011     
0012     atoms       % list of all atoms appearing in a rule
0013 end
0014 
0015 properties
0016     AND = false   % is an AND junction
0017     OR = false    % is an OR junction
0018     cond_op = ''  % operator for conditional expressions
0019     IFF = false   % is an IFF rule
0020     IF  = false   % is an IF rule
0021     lexpr         % left-hand-side expression
0022     rexpr         % right-hand-side expression
0023 
0024     negated = false  % true if actual rule preceeded by a NOT
0025     
0026     is_numeric = false  % true if EXPR is a numeric constant
0027     was_quoted = false  % true if original id was quoted
0028 
0029     id = ''       % atom name
0030     display_id    % atom name prefaced by '~' if negated
0031 
0032     NULL = false  % not an EXPR (returned by parsing empty strings)
0033 end
0034 
0035 methods
0036     function [tf] = get.is_junc(obj)
0037         tf = (obj.AND || obj.OR);
0038     end
0039     
0040     function [tf] = get.is_cond(obj)
0041         tf = ~isempty(obj.cond_op);
0042     end
0043     
0044     function [tf] = get.is_rule(obj)
0045         tf = (obj.IFF || obj.IF);
0046     end
0047     
0048     function [tf] = get.is_atom(obj)
0049         tf = ~isempty(obj.id);
0050     end
0051 
0052     function [atoms] = get.atoms(obj)
0053         if ~isempty(obj.lexpr) || ~isempty(obj.rexpr)
0054             latoms = subsref(obj.lexpr,substruct('.','atoms'));
0055             ratoms = subsref(obj.rexpr,substruct('.','atoms'));
0056             atoms = unique([latoms ratoms]);
0057         else
0058             if obj.is_numeric
0059                 atoms = {};
0060             else
0061                 atoms = {obj.id};
0062             end
0063         end
0064     end
0065     
0066     function [tf] = get.is_simple(obj)
0067         tf =    obj.is_atom ...
0068              || (obj.is_cond && ~obj.negated) ...
0069              || (obj.is_junc && obj.lexpr.is_atom ...
0070                              && obj.rexpr.is_atom);
0071     end  
0072     
0073     function [new] = copy(obj)
0074         % perform a deep copy of the handle
0075         new = expr();
0076         props = properties(obj);
0077         for p = 1 : length(props)
0078             p_meta = findprop(expr,props{p});
0079             if ~p_meta.Dependent
0080                 new.(props{p}) = obj.(props{p});
0081             end
0082         end
0083         
0084         % deep copy
0085         if ~isempty(obj.lexpr)
0086             new.lexpr = obj.lexpr.copy;
0087         end
0088         if ~isempty(obj.rexpr)
0089             new.rexpr = obj.rexpr.copy;
0090         end
0091     end
0092     
0093     % ------------ manipulation ------------
0094     
0095     function iterif(e,test,f)
0096         % Iterate over every part of the expression, applying function
0097         % handle F if function handle TEST is true.  Modifies the
0098         % expression in place.
0099         if test(e)
0100             f(e);
0101         end
0102         if ~isempty(e.lexpr)
0103             e.lexpr.iterif(test,f);
0104         end
0105         if ~isempty(e.rexpr)
0106             e.rexpr.iterif(test,f);
0107         end
0108     end
0109     
0110     function [new_e] = mapif(e,test,f)
0111         % Map function F over each part of the expression where function
0112         % handle TEST is true.  Returns a new copy of the expression.
0113         new_e = e.copy;
0114         new_e.iterif(test,f);
0115     end
0116     
0117     function iter(e,f)
0118         % Apply function handle F to every part of the expression,
0119         % modifying the expression in place.
0120         e.iterif(@(x) true,f);
0121     end
0122     
0123     function [new_e] = map(e,f)
0124         % Map function F over each part of the expression, returning a new
0125         % copy of the expression.
0126         new_e = e.mapif(@(x) true,f);
0127     end
0128     
0129     function demorgan(obj)
0130         % Apply DeMorgan's rule to the expression:
0131         %       NOT (x AND y) -> (NOT x) OR (NOT y)
0132         %       NOT (x OR y)  -> (NOT x) AND (NOT y)
0133         %       NOT (NOT x)   -> x
0134         if obj.negated && obj.is_junc
0135             obj.AND = ~obj.AND;
0136             obj.OR  = ~obj.OR;
0137             obj.negated = false;
0138             
0139             obj.lexpr.negated = ~obj.lexpr.negated;
0140             obj.rexpr.negated = ~obj.rexpr.negated;
0141         elseif obj.negated && obj.is_cond
0142             obj.negated = false;
0143             switch obj.cond_op
0144                 case '='
0145                     obj.cond_op = '~=';
0146                 case '~='
0147                     obj.cond_op = '=';
0148                 case '>='
0149                     obj.cond_op = '<';
0150                 case '<'
0151                     obj.cond_op = '>=';
0152                 case '<='
0153                     obj.cond_op = '>';
0154                 case '>'
0155                     obj.cond_op = '<=';
0156             end
0157         end
0158         
0159         if obj.lexpr.is_junc || obj.lexpr.is_cond
0160             obj.lexpr.demorgan();
0161         end
0162         if obj.rexpr.is_junc || obj.rexpr.is_cond
0163             obj.rexpr.demorgan();
0164         end
0165     end
0166     
0167     % ------------ display routines ------------
0168     
0169     function [str] = get.display_id(obj)
0170         if obj.negated
0171             str = ['~' obj.id];
0172         else
0173             str = obj.id;
0174         end
0175     end
0176     
0177     function [str] = to_string(obj)
0178         % Format an expression as a one-line string.
0179         if obj.is_atom
0180             str = obj.id;
0181         elseif obj.AND
0182             str = make_cons(' & ');
0183         elseif obj.OR
0184             str = make_cons(' | ');
0185         elseif obj.IF
0186             str = make_cons(' => ');
0187         elseif obj.IFF
0188             str = make_cons(' <=> ');
0189         elseif obj.is_cond
0190             str = obj.cond_to_str();
0191         else
0192             str = '';
0193         end
0194         
0195         if obj.negated
0196             str = ['~' str];
0197         end
0198         
0199         function [s] = make_cons(sep)
0200             s = ['(' obj.lexpr.to_string() sep obj.rexpr.to_string() ')'];
0201         end
0202     end
0203     
0204     function [str] = cond_to_str(obj)
0205         % Format a conditional as a one-line string.
0206         str = [obj.lexpr.display_id ' ' ...
0207                obj.cond_op ' ' ...
0208                obj.rexpr.display_id];
0209         if obj.negated
0210             str = ['(' str ')'];
0211         end
0212     end
0213     
0214     function [frame] = make_textframe(obj,indent,pipe)
0215         % Converts an expression (not a rule) into a textframe containing
0216         % a tree structure.
0217         frame = textframe();
0218         if obj.NULL
0219             return
0220         end
0221         
0222         TOPLEVEL_INDENT = '   ';
0223         TIGHT_DISPLAY = false;
0224 
0225         if TIGHT_DISPLAY
0226             BASE_INDENT = '     ';
0227             EXTRA_SPACER = '';
0228         else
0229             BASE_INDENT = '      ';
0230             EXTRA_SPACER = '-';
0231         end
0232 
0233         toplevel = (nargin < 2);
0234 
0235         if obj.AND
0236             name = 'AND';
0237         elseif obj.OR
0238             name = 'OR';
0239         elseif obj.is_atom
0240             name = obj.id;
0241         elseif obj.is_cond
0242             name = obj.cond_to_str;
0243         else % rule?
0244             name = '';
0245         end
0246 
0247         if obj.negated
0248             spacer = [EXTRA_SPACER '- ~'];
0249         else
0250             spacer = [EXTRA_SPACER '-- '];
0251         end
0252 
0253         if toplevel
0254             indent = TOPLEVEL_INDENT;
0255             if obj.negated
0256                 frame.add_line('%s~%s',indent(1:end-1),name);
0257             else
0258                 frame.add_line('%s%s',indent,name);
0259             end
0260         else
0261             if pipe
0262                 frame.add_line('%s |%s%s',indent,spacer,name);
0263                 indent = [indent ' |' BASE_INDENT(3:end)];
0264             else
0265                 frame.add_line('%s +%s%s',indent,spacer,name);
0266                 indent = [indent BASE_INDENT];
0267             end
0268         end
0269         if ~isempty(obj.lexpr) && ~obj.is_cond
0270             frame = frame.vcat(obj.lexpr.make_textframe(indent,true));
0271             if ~TIGHT_DISPLAY
0272                 frame.add_line('%s |',indent);
0273             end
0274             frame = frame.vcat(obj.rexpr.make_textframe(indent,false));
0275         end
0276     end
0277     
0278     function display(obj)
0279         % Show a rule or expression as a true structure, overriding the
0280         % builtin display as a structure.
0281         if obj.is_rule
0282             l = obj.lexpr.make_textframe();
0283             r = obj.rexpr.make_textframe();
0284             sign = textframe();
0285             if obj.IFF
0286                 sign.add_line('   <==> ');
0287             else
0288                 sign.add_line('    ==> ');
0289             end
0290             l.hcat(sign,r,'valign','middle').display();
0291         else
0292             obj.make_textframe().display();
0293         end
0294     end
0295 end
0296 
0297 end % classdef
0298 
0299
```

---

Generated on Thu 11-Aug-2011 15:06:22 by **m2html** © 2005
