# Supplementary material for: TIGER: Toolbox for integrating genome-scale metabolic models, expression data, and transcriptional regulatory networks
Source: BMC Syst Biol. 2011 Sep 23;5:147. doi: 10.1186/1752-0509-5-147 (PMC3224351; doi:10.1186/1752-0509-5-147)
Supplement: Additional file 2 — TIGER source code. Source code, documentation, and tutorials are also available online at http://bme.virginia.edu/csbl/downloads/ or http://csbl.bitbucket.org/tiger. [file 1752-0509-5-147-S2.GZ › tiger/doc/m2html/tiger/fba.html]

Description of fba


Home > tiger > fba.m

# fba

## PURPOSE

**Run Flux Balance Analysis on a TIGER model.**

## SYNOPSIS

**function [sol] = fba(tiger,fluxnorm,obj\_frac)**

## DESCRIPTION

```
 FBA  Run Flux Balance Analysis on a TIGER model.

   [SOL] = FBA(TIGER)
   [SOL] = FBA(TIGER,FLUXNORM,OBJ_FRAC)

   Run Flux Balance Analysis on a TIGER model, returning a CMPI solution
   structure.  If given, FLUXNORM specifies the norm to be minimized;
   choices include 
       'none' (default):
           maximize obj*v
           s.t. Sv = 0
                lb <= v <= ub
       'two' (Euclidian)
           minimize v'*v
           s.t. Sv = 0
                lb <= v <= ub
                obj*v >= OBJ_FRAC*v_max

   Unless specified, OBJ_FRAC defaults to 1.0.
```

## CROSS-REFERENCE INFORMATION

This function calls:

- add\_growth\_constraint Add minimum growth constraint to a model.

This function is called by:

- add\_growth\_constraint Add minimum growth constraint to a model.
- minimal\_genome Calculate a minimal genome
- single\_gene\_ko Perform single gene knockout simulations
- test\_\_add\_diff
- test\_\_fba
- test\_\_gimme
- test\_\_indicators
- test\_\_multilevel
- test\_\_tile\_mip
- gal\_test
- run\_new\_rules
- run\_trn\_comparison
- diffadj Formulate and solve the differential adjustment problem

## SOURCE CODE

```
0001 function [sol] = fba(tiger,fluxnorm,obj_frac)
0002 % FBA  Run Flux Balance Analysis on a TIGER model.
0003 %
0004 %   [SOL] = FBA(TIGER)
0005 %   [SOL] = FBA(TIGER,FLUXNORM,OBJ_FRAC)
0006 %
0007 %   Run Flux Balance Analysis on a TIGER model, returning a CMPI solution
0008 %   structure.  If given, FLUXNORM specifies the norm to be minimized;
0009 %   choices include
0010 %       'none' (default):
0011 %           maximize obj*v
0012 %           s.t. Sv = 0
0013 %                lb <= v <= ub
0014 %       'two' (Euclidian)
0015 %           minimize v'*v
0016 %           s.t. Sv = 0
0017 %                lb <= v <= ub
0018 %                obj*v >= OBJ_FRAC*v_max
0019 %
0020 %   Unless specified, OBJ_FRAC defaults to 1.0.
0021 
0022 if nargin < 3
0023     obj_frac = 1.0;
0024 end
0025 
0026 if nargin < 2
0027     fluxnorm = 'none';
0028 end
0029 
0030 switch fluxnorm
0031     case {'none','fba'}
0032         % standard FBA
0033         tiger.sense = -1;
0034         sol = cmpi.solve_mip(tiger);
0035     case {'euclid','two','quad'}
0036         % Euclidian norm
0037         tiger = add_growth_constraint(tiger,obj_frac);
0038         nS = size(tiger.S,2);
0039         nA = size(tiger.A,2);
0040         tiger.Q = spalloc(nA,nA,nS);
0041         tiger.Q(1:nS,1:nS) = eye(nS);
0042         sol = cmpi.solve_mip(tiger);
0043 end
0044
```

---

Generated on Thu 11-Aug-2011 15:06:22 by **m2html** © 2005
