# Supplementary material for: TIGER: Toolbox for integrating genome-scale metabolic models, expression data, and transcriptional regulatory networks
Source: BMC Syst Biol. 2011 Sep 23;5:147. doi: 10.1186/1752-0509-5-147 (PMC3224351; doi:10.1186/1752-0509-5-147)
Supplement: Additional file 2 — TIGER source code. Source code, documentation, and tutorials are also available online at http://bme.virginia.edu/csbl/downloads/ or http://csbl.bitbucket.org/tiger. [file 1752-0509-5-147-S2.GZ › tiger/doc/m2html/tiger/show_exchange_rxns.html]

Description of show\_exchange\_rxns


Home > tiger > show\_exchange\_rxns.m

# show\_exchange\_rxns

## PURPOSE

**Show exchange reactions (and fluxes)**

## SYNOPSIS

**function show\_exchange\_rxns(model,sol)**

## DESCRIPTION

```
 SHOW_EXCHANGE_RXNS  Show exchange reactions (and fluxes)

   SHOW_EXCHANGE_RXNS(MODEL)
   SHOW_EXCHANGE_RXNS(MODEL,SOL)

   Shows the metabolite name and index of exchange reactions in MODEL.
   If a solution structure or flux vector SOL is given, only nonzero
   fluxes are displayed (with the corresponding flux value).
```

## CROSS-REFERENCE INFORMATION

This function calls:

- find\_exchange\_rxns Find locations of exchange reactions
- map Generate a new list by applying a function

This function is called by:


## SOURCE CODE

```
0001 function show_exchange_rxns(model,sol)
0002 % SHOW_EXCHANGE_RXNS  Show exchange reactions (and fluxes)
0003 %
0004 %   SHOW_EXCHANGE_RXNS(MODEL)
0005 %   SHOW_EXCHANGE_RXNS(MODEL,SOL)
0006 %
0007 %   Shows the metabolite name and index of exchange reactions in MODEL.
0008 %   If a solution structure or flux vector SOL is given, only nonzero
0009 %   fluxes are displayed (with the corresponding flux value).
0010 
0011 show_flux = nargin >= 2;
0012 
0013 if show_flux && isa(sol,'struct')
0014     sol = sol.x;
0015 end
0016 
0017 ex_rxns = find_exchange_rxns(model);
0018 
0019 % find corresponding metabolite
0020 if isfield(model,'metNames')
0021     mets = model.metNames;
0022 elseif isfield(model,'mets')
0023     mets = model.mets;
0024 else
0025     mets = model.varnames;
0026 end
0027     
0028 metnamef = @(x) mets{model.S(:,x) ~= 0};
0029 metnames = map(metnamef,ex_rxns);
0030 
0031 max_length = max(cellfun(@length,metnames));
0032 
0033 if show_flux
0034     fmt = '%*s (%4i):  %+08f\n';
0035 else
0036     fmt = '%*s (%4i)\n';
0037 end
0038 
0039 fprintf('\n\n');
0040 for i = 1 : length(ex_rxns)
0041     if show_flux && sol(ex_rxns(i)) ~= 0
0042         fprintf(fmt,max_length,metnames{i},ex_rxns(i),sol(ex_rxns(i)));
0043     elseif ~show_flux
0044         fprintf(fmt,max_length,metnames{i},ex_rxns(i));
0045     end
0046 end
0047 fprintf('\n\n');
```

---

Generated on Thu 11-Aug-2011 15:06:22 by **m2html** © 2005
