# Supplementary material for: TIGER: Toolbox for integrating genome-scale metabolic models, expression data, and transcriptional regulatory networks
Source: BMC Syst Biol. 2011 Sep 23;5:147. doi: 10.1186/1752-0509-5-147 (PMC3224351; doi:10.1186/1752-0509-5-147)
Supplement: Additional file 2 — TIGER source code. Source code, documentation, and tutorials are also available online at http://bme.virginia.edu/csbl/downloads/ or http://csbl.bitbucket.org/tiger. [file 1752-0509-5-147-S2.GZ › tiger/doc/m2html/tiger/extract_cobra.html]

Description of extract\_cobra


Home > tiger > extract\_cobra.m

# extract\_cobra

## PURPOSE

**Extract the original COBRA model from a TIGER structure.**

## SYNOPSIS

**function [cobra] = extract\_cobra(tiger)**

## DESCRIPTION

```
 EXTRACT_COBRA  Extract the original COBRA model from a TIGER structure.

   [COBRA] = EXTRACT_COBRA(TIGER)

   Returns the original COBRA model structure from a TIGER model.
```

## CROSS-REFERENCE INFORMATION

This function calls:


This function is called by:

- imat Integrative Metabolic Analysis Tool

## SOURCE CODE

```
0001 function [cobra] = extract_cobra(tiger)
0002 % EXTRACT_COBRA  Extract the original COBRA model from a TIGER structure.
0003 %
0004 %   [COBRA] = EXTRACT_COBRA(TIGER)
0005 %
0006 %   Returns the original COBRA model structure from a TIGER model.
0007 
0008 tiger_fields = {'A','obj','ctypes','vartypes','gpr','ind','indtypes', ...
0009                 'varnames','rownames','param'};
0010 
0011 to_remove = tiger_fields(isfield(tiger,tiger_fields));
0012 cobra = rmfield(tiger,to_remove);
0013 
0014 [m,n] = size(cobra.S);
0015 cobra.b = cobra.b(1:m);
0016 cobra.lb = cobra.lb(1:n);
0017 cobra.ub = cobra.ub(1:n);
0018 
0019 if ~isfield(cobra,'c')
0020     cobra.c = tiger.obj(1:n);
0021 end
```

---

Generated on Thu 11-Aug-2011 15:06:22 by **m2html** © 2005
