# Supplementary material for: TIGER: Toolbox for integrating genome-scale metabolic models, expression data, and transcriptional regulatory networks
Source: BMC Syst Biol. 2011 Sep 23;5:147. doi: 10.1186/1752-0509-5-147 (PMC3224351; doi:10.1186/1752-0509-5-147)
Supplement: Additional file 2 — TIGER source code. Source code, documentation, and tutorials are also available online at http://bme.virginia.edu/csbl/downloads/ or http://csbl.bitbucket.org/tiger. [file 1752-0509-5-147-S2.GZ › tiger/doc/m2html/tiger/about_tiger.html]

Description of about\_tiger


Home > tiger > about\_tiger.m

# about\_tiger

## PURPOSE

## SYNOPSIS

**function about\_tiger()**

## DESCRIPTION

## CROSS-REFERENCE INFORMATION

This function calls:


This function is called by:


## SOURCE CODE

```
0001 function about_tiger()
0002 
0003 VERSION = '0.9.1 (08/05/2011)';
0004 
0005 fprintf('\n\n');
0006 fprintf('TIGER:  Toolbox for Integrating Genome-scale metabolism,\n');
0007 fprintf('        Expression, and Regulation\n\n');
0008 fprintf('Version:  %s\n\n',VERSION);
0009 fprintf('TIGER is maintained by the \n');
0010 fprintf('        Computational Systems Biology Laboratory\n');
0011 fprintf('          Department of Biomedical Engineering\n');
0012 fprintf('                 University of Virginia\n\n');
0013 fprintf('Questions, comments, and suggestions can be sent to \n');
0014 fprintf('tigerproject@email.virginia.edu\n\n');
0015 fprintf('Source code and documentation are available online at\n');
0016 fprintf('http://csbl.bitbucket.org/tiger\n\n');
```

---

Generated on Thu 11-Aug-2011 15:06:22 by **m2html** © 2005
