# Supplementary material for: TIGER: Toolbox for integrating genome-scale metabolic models, expression data, and transcriptional regulatory networks
Source: BMC Syst Biol. 2011 Sep 23;5:147. doi: 10.1186/1752-0509-5-147 (PMC3224351; doi:10.1186/1752-0509-5-147)
Supplement: Additional file 2 — TIGER source code. Source code, documentation, and tutorials are also available online at http://bme.virginia.edu/csbl/downloads/ or http://csbl.bitbucket.org/tiger. [file 1752-0509-5-147-S2.GZ › tiger/doc/m2html/tiger/set_var.html]

Description of set\_var


Home > tiger > set\_var.m

# set\_var

## PURPOSE

**Set bounds on a variable**

## SYNOPSIS

**function [tiger] = set\_var(tiger,id,lb,ub)**

## DESCRIPTION

```
 SET_VAR  Set bounds on a variable

   [TIGER] = SET_VAR(TIGER,ID,VAL)
   [TIGER] = SET_VAR(TIGER,ID,LB,UB)

   Sets the bounds on variables in a TIGER model, returning the modified
   model structure.  ID can be any ids accepted by CONVERT_IDS.  The
   following calling formats are allowed:

       SET_VAR(TIGER,ID,VAL)
           Sets upper and lower bounds to VAL.
       SET_VAR(TIGER,ID,LB,UB)
           Sets lower bound to LB, upper bound to UB.
       SET_VAR(TIGER,ID,[],UB)
           Sets upper bound to UB; lower bound is unchanged.
       SET_VAR(TIGER,ID,LB,[])
           Sets lower bound to LB; upper bound is unchanged.

   If multiple IDS are given but only a single value is specified for LB
   or UB, this value is repeated.
```

## CROSS-REFERENCE INFORMATION

This function calls:

- set\_fieldval Set values in a TIGER structure field

This function is called by:

- test\_\_add\_diff
- test\_\_fba
- test\_\_multilevel
- test\_\_tile\_mip
- diffadj Formulate and solve the differential adjustment problem
- gimme Gene Inactivity Moderated by Metabolism and Expression
- imat Integrative Metabolic Analysis Tool

## SOURCE CODE

```
0001 function [tiger] = set_var(tiger,id,lb,ub)
0002 % SET_VAR  Set bounds on a variable
0003 %
0004 %   [TIGER] = SET_VAR(TIGER,ID,VAL)
0005 %   [TIGER] = SET_VAR(TIGER,ID,LB,UB)
0006 %
0007 %   Sets the bounds on variables in a TIGER model, returning the modified
0008 %   model structure.  ID can be any ids accepted by CONVERT_IDS.  The
0009 %   following calling formats are allowed:
0010 %
0011 %       SET_VAR(TIGER,ID,VAL)
0012 %           Sets upper and lower bounds to VAL.
0013 %       SET_VAR(TIGER,ID,LB,UB)
0014 %           Sets lower bound to LB, upper bound to UB.
0015 %       SET_VAR(TIGER,ID,[],UB)
0016 %           Sets upper bound to UB; lower bound is unchanged.
0017 %       SET_VAR(TIGER,ID,LB,[])
0018 %           Sets lower bound to LB; upper bound is unchanged.
0019 %
0020 %   If multiple IDS are given but only a single value is specified for LB
0021 %   or UB, this value is repeated.
0022 
0023 assert(nargin > 2, 'At least three arguments required.');
0024 
0025 if nargin == 3
0026     ub = lb;
0027 end
0028 
0029 if ~isempty(lb)
0030     tiger = set_fieldval(tiger,'lb',id,lb);
0031 end
0032 if ~isempty(ub)
0033     tiger = set_fieldval(tiger,'ub',id,ub);
0034 end
```

---

Generated on Thu 11-Aug-2011 15:06:22 by **m2html** © 2005
