# Supplementary material for: TIGER: Toolbox for integrating genome-scale metabolic models, expression data, and transcriptional regulatory networks
Source: BMC Syst Biol. 2011 Sep 23;5:147. doi: 10.1186/1752-0509-5-147 (PMC3224351; doi:10.1186/1752-0509-5-147)
Supplement: Additional file 2 — TIGER source code. Source code, documentation, and tutorials are also available online at http://bme.virginia.edu/csbl/downloads/ or http://csbl.bitbucket.org/tiger. [file 1752-0509-5-147-S2.GZ › tiger/doc/m2html/tiger/remove_rule.html]

Description of remove\_rule


Home > tiger > remove\_rule.m

# remove\_rule

## PURPOSE

**Remove rule(s) previously added to a TIGER model**

## SYNOPSIS

**function [tiger] = remove\_rule(tiger,rule,varargin)**

## DESCRIPTION

```
 REMOVE_RULE  Remove rule(s) previously added to a TIGER model

   [TIGER] = REMOVE_RULE(TIGER,RULE,...params...)

   Remove the constraints generated by a list of rules.  RULE can be
   either a string, EXPR object, or a cell.  Removed constriants are
   zeroed, and the rules is removed from TIGER.param.rules.

   Parameters given to the function are passed to REMOVE_NULL_RULES.
```

## CROSS-REFERENCE INFORMATION

This function calls:

- expr
- parse\_string Parse a rule string into an EXPR object
- remove\_null\_rules Remove NULL rules from a TIGER model
- assert\_cell Assert that variable is a cell array.
- map Generate a new list by applying a function

This function is called by:

- test\_\_remove\_rule
- update\_rule Re-compile rule(s) previously added to a TIGER model

## SOURCE CODE

```
0001 function [tiger] = remove_rule(tiger,rule,varargin)
0002 % REMOVE_RULE  Remove rule(s) previously added to a TIGER model
0003 %
0004 %   [TIGER] = REMOVE_RULE(TIGER,RULE,...params...)
0005 %
0006 %   Remove the constraints generated by a list of rules.  RULE can be
0007 %   either a string, EXPR object, or a cell.  Removed constriants are
0008 %   zeroed, and the rules is removed from TIGER.param.rules.
0009 %
0010 %   Parameters given to the function are passed to REMOVE_NULL_RULES.
0011 
0012 if isa(rule,'double')
0013     % indices given
0014     exprs = tiger.param.rules(rule);
0015 else
0016     exprs = assert_cell(parse_string(rule));
0017 end
0018 
0019 null_expr = expr();
0020 null_expr.NULL = true;
0021 
0022 N = length(exprs);
0023 rule_strs = map(@(x) x.to_string,tiger.param.rules);
0024 for i = 1 : N
0025     % find matching rules
0026     matched = find(cellfun(@(x) strcmp(x,exprs{i}.to_string),rule_strs));
0027     % find corresponding rows
0028     to_zero = ismember(tiger.param.rule_id,matched);
0029     % zero the rows
0030     tiger.A(to_zero,:) = 0;
0031     tiger.b(to_zero) = 0;
0032     tiger.ind(to_zero) = 0;
0033     tiger.indtypes(to_zero) = ' ';
0034     % replace the removed rule with the null expression
0035     for j = 1 : length(matched)
0036         tiger.param.rules{matched(j)} = null_expr.copy;
0037     end
0038 end
0039 
0040 tiger = remove_null_rules(tiger,varargin{:});
```

---

Generated on Thu 11-Aug-2011 15:06:22 by **m2html** © 2005
